# Expression complementation of gene presence/absence polymorphisms in hybrids contributes importantly to heterosis in sunflower

**DOI:** 10.1016/j.jare.2022.04.008

**Published:** 2022-04-22

**Authors:** Joon Seon Lee, Mojtaba Jahani, Kaichi Huang, Jennifer R. Mandel, Laura F. Marek, John M. Burke, Nicolas B. Langlade, Gregory L. Owens, Loren H. Rieseberg

**Affiliations:** a Department of Botany and Biodiversity Research Centre, University of British Columbia, Vancouver, BC V6T 1Z4, Canada; b Department of Biological Sciences and Center for Biodiversity, University of Memphis, Memphis, TN 38152, USA; c Department of Agronomy, Iowa State University, Ames, IA 50011, USA; d Department of Plant Biology, Miller Plant Sciences, University of Georgia, Athens 30602, Georgia; e LIPM, Université de Toulouse, INRA, CNRS, Castanet-Tolosan, France; f Department of Biology, University of Victoria, Victoria, BC V8P 5C2, Canada

**Keywords:** Drought stress, Expression complementation, Heterosis, Presence absence variation, Sunflower

## Abstract

**Introduction:**

Numerous crops have transitioned to hybrid seed production to increase yields and yield stability through heterosis. However, the molecular mechanisms underlying heterosis and its stability across environments are not yet fully understood.

**Objectives:**

This study aimed to (1) elucidate the genetic and molecular mechanisms underlying heterosis in sunflower, and (2) determine how heterosis is maintained under different environments.

**Methods:**

Genome-wide association (GWA) analyses were employed to assess the effects of presence/absence variants (PAVs) and stop codons on 16 traits phenotyped in the sunflower association mapping population at three locations. To link the GWA results to transcriptomic variation, we sequenced the transcriptomes of two sunflower cultivars and their F_1_ hybrid (INEDI) under both control and drought conditions and analyzed patterns of gene expression and alternative splicing.

**Results:**

Thousands of PAVs were found to affect phenotypic variation using a relaxed significance threshold, and at most such loci the “absence” allele reduced values of heterotic traits, but not those of non-heterotic traits. This pattern was strengthened for PAVs that showed expression complementation in INEDI. Stop codons were much rarer than PAVs and less likely to reduce heterotic trait values. Hybrid expression patterns were enriched for the GO category, sensitivity to stimulus, but all genotypes responded to drought similarily – by up-regulating water stress response pathways and down-regulating metabolic pathways. Changes in alternative splicing were strongly negatively correlated with expression variation, implying that alternative splicing in this system largely acts to reinforce expression responses.

**Conclusion:**

Our results imply that complementation of expression of PAVs in hybrids is a major contributor to heterosis in sunflower, consistent with the dominance model of heterosis. This mechanism can account for yield stability across different environments. Moreover, given the much larger numbers of PAVs in plant vs. animal genomes, it also offers an explanation for the stronger heterotic responses seen in the former.

## Highlights

• For most PAVs in sunflower, the absence allele reduces heterotic trait values.
• This pattern was strongest for PAVs with expression complementation in hybrids.
• Stop codons were rarer than PAVs and less likely to reduce heterotic trait values.
• Expression complementation seen under both control and drought conditions.
• Complementation of expression of PAVs is major contributor to heterosis.
• This mechanism can account for yield stability across different environments.

## Graphical abstract

**Figure:**
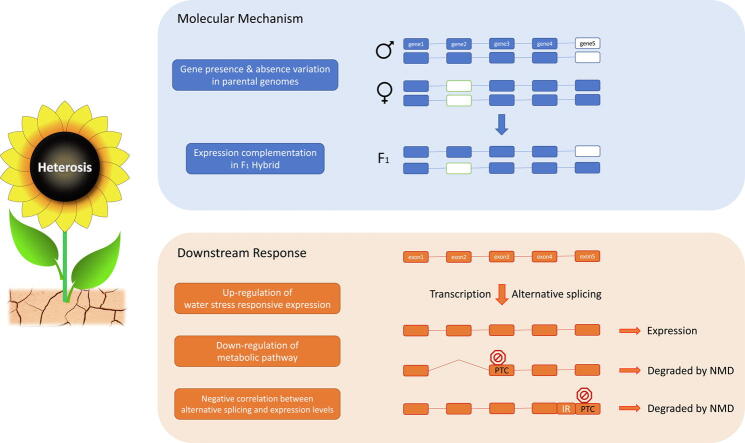

## Introduction

Over the past century, a number of the world’s most important crops have transitioned from open-pollinated varieties to hybrid production. This transition has been driven in large part by the immediate yield increase offered by heterosis or hybrid vigor, as well as by the greater biological and legal protection afforded by hybrid crops. A less widely recognized benefit has been the impact of heterosis on environmental yield stability, especially in response to various abiotic stresses [1], [2]. Examples include increased tolerance to low nutrients and drought in maize [1], heat tolerance in canola [3] and rice [4], and drought tolerance in sunflower [5]. That heterosis sometimes has a positive effect on drought tolerance is especially surprising, because the higher growth rates and biomass accumulation resulting from heterosis are expected to require greater water usage [6].

## Mechanisms of heterosis

Despite its importance in plant breeding and evolution, the genetic, physiological, and molecular bases of heterosis are not fully understood (reviewed in [7], [8]). Until recently, there were three main genetic models: (1) the dominance model, in which deleterious recessive alleles found in one parent are masked by superior dominant alleles from the other parent [7], [9]; (2) the over-dominance model, in which the alleles at individual loci interact to increase performance [8], [10], [11]; and (3) epistasis, in which favorable interactions among alleles at different genes are the cause of heterosis [12], [13]. Quantitative trait locus (QTL) and genome wide association (GWA) studies have offered support for all three models but imply that the dominance model is most common [14], [15], [16].

Genomic and functional studies have attempted to understand the molecular mechanisms responsible for heterosis and relate them to the genetic models (reviewed in [7], [8]). Support has been found for the dominance model. In maize, numerous genes with missing expression in one or the other parental genotype are expressed in hybrids, and hybrids express many more genes overall than either parent [17]. Although not directly linked to heterotic phenotypes, such a pattern of expression complementation in hybrids is consistent with the dominance model, and likely results in part from copy number variation (CNV) and presence/absence variation (PAV) in underlying genes [18]. Evidence for the overdominance model has also been reported, but less frequently. For example, in tomato, allelic interactions at the *SFT* (*SINGLE FLOWER TRUSS*) gene lead to single locus overdominance via a change in floral architecture [19].

Transcriptomic studies often suggest a more complex and idiosyncratic genetic architecture underlying heterosis (reviewed in [20]). Numerous studies have documented non-additive gene expression patterns in hybrids [21], which often appear to arise from epigenetic perturbations at key regulatory loci (reviewed in [7]). However, determining whether such patterns are a cause or consequence of heterosis can be challenging. Moreover, in wide crosses, non-additive expression may result, in part, from transcriptome shock rather than heterosis [22]. Nonetheless, changes in hybrid gene expression can offer clues regarding the genes and genetic networks contributing to heterotic phenotypes, especially in intraspecific crosses. For example, in *Arabidopsis*, expression changes in genes underlying circadian rhythms such as *CIRCADIAN CLOCK-ASSOCIATED 1* (*CCA1*) have been shown to increase photosynthesis and starch metabolism, leading to increased biomass [23]. Interestingly, the increase in biomass in *Arabidopsis* comes at the cost of decreased resistance to both biotic [24] and abiotic stress [16], [21]. Although up-regulation of photosynthesis and carbon metabolism is commonly observed in hybrids (e.g., [25], [26]), outside of *Arabidopsis* it often is accompanied by an increase rather than decrease in stress tolerance (reviewed in [2]). How this is achieved remains an open question.

### Mechanisms of drought tolerance

While a universal mechanism for heterosis remains outside of our reach, arguably greater progress has been made towards understanding the genetic, physiological and molecular bases of responses to drought. Drought stress tolerance/resistance can be achieved by drought escape, dehydration avoidance or dehydration tolerance (reviewed in [27]). Drought escape is typically accomplished by rapid growth and early flowering [28]. Such an adaptive drought response can be triggered by photoperiod or other environmental cues [29]. Drought avoidance is achieved by reducing water loss and enhancing water capture from the soil. Stomatal regulation [30] and higher ratios of root to shoot growth [31] represent common mechanisms for increasing water-use efficiency (WUE), thereby minimizing the impacts of drought. Lastly, dehydration tolerance can be enhanced by regulating the phytohormone abscisic acid (ABA) response [32] and via osmotic adjustment [33].

Transcriptome analyses have been especially effective for elucidating the genetic networks underlying drought responses in different crop plants. For example, Zheng et al. [34] showed that the ABA signaling pathway was significantly upregulated in response to drought stress in maize and suggested that differential expression of cell wall-related genes (e.g., subunits of cellulose synthase, pectinesterase, and expansin), as well as transporter genes (e.g., ion and sugar transporters) may account for the different responses of the two genotypes included in the experiment. Lenka et al. [35] compared transcriptomes of drought-tolerant and drought-susceptible genotypes of rice; drought tolerance was found to be associated with up-regulation of carbon metabolism similar to that frequently seen in transcriptome analyses of heterosis. The studies above suggest that the regulation of drought responsive genes (e.g., zinc finger proteins shown to confer drought stress tolerance – [35]) are critically influenced by genotypic variation.

Likewise, a comprehensive comparative transcriptome study in chickpea under drought and/or salinity stress found that genes associated with metabolic pathways were up-regulated under drought, as well as a variety of transcription factors and enzymes that are known from other studies to contribute to drought tolerance [36]. In canola, drought resistance was found to be associated with differential expression of root developmental genes [37]. In sunflower, several fatty acid desaturase genes, ABA-responsive genes such as *ABI2*, as well as a helix-loop-helix transcription factor (*HabHLH024*) were found to be up-regulated under drought stress [38], [39], [40]. Overall, these findings are largely consistent with the different phenotypic and physiological mechanisms known to underlie drought tolerance and drought avoidance.

### Regulation of heterotic responses to drought by alternative splicing

Expression responses to drought have also been shown to be further regulated by alternative splicing (AS), a post-transcriptional mechanism (reviewed in [41], [42]). The major types of AS are: i) intron retention, ii) alternative donor, iii) alternative acceptor, iv) alternative position, and v) exon skipping [41]. AS can affect gene function in two major ways: some transcript forms are translated to produce alternative protein isoforms [43], and other transcripts are degraded by non-sense mediated RNA decay (NMD) to reduce the level of gene expression [44]. AS can have downstream effects on drought stress response and tolerance. For example, a truncated isoform of the *Arabidopsis* zinc-induced facilitator-like 1 (*ZIFL1*) transporter is targeted to the plasma membrane of leaf stomatal guard cells instead of the tonoplast of root cells (full length isoform) and mediates drought tolerance [45]. Interestingly, AS of the key circadian rhythm gene *CCA1* has been shown to mediate drought responses in maize [46], similar to its role in heterosis (above). However, it is currently largely unknown how AS contributes to heterotic responses to drought.

### Approach taken by present study

A major challenge to studying the dominance model of heterosis is that deleterious mutations tend to be numerous, to occur at low frequencies within populations, and to have relatively small phenotypic effects. As a result, genome wide association (GWA) studies have little power to detect them. Here, we take a novel GWA approach to this problem based on the hypothesis that the absence allele in PAVs is more likely to be deleterious than the presence allele, as well as our understanding of the categories of traits (i.e., growth rate, biomass, and yield-related traits) that are expected to exhibit heterotic phenotypes and those that are not (i.e., quality traits). We also employed ridge regression best linear unbiased prediction (RR-BLUP) to train a genomic prediction model and estimate the size and direction of effects of PAVs. Genomic prediction uses all marker effects across the entire genome to train a predictive model, which makes it suitable for polygenic quantitative traits, especially those with low heritability [47]. Transcriptomic analyses are subsequently employed to link the GWA results to patterns of gene expression and AS, explore how heterosis is maintained under both well-watered and drought conditions, and identify genes and gene networks underlying heterotic responses.

Our focus is on sunflower, *Helianthus annuus* L., which is a global oilseed crop that moved from an open-pollinated production system to hybrid production in the 1970s. It currently ranks as the second most important hybrid crop after maize [48]. While considered to be a drought tolerant crop, yield in many parts of the world is limited by drought stress [49]. Also, cultivated lines are far less drought tolerant than compatible wild relatives [50]. We make use of the cultivated sunflower association mapping (SAM) population for the GWA analyses [51] and a well-characterized F_1_ hybrid (INEDI) and its maternal (XRQ) and paternal (PSC8) parental lines for the gene expression and AS analyses. High quality reference sequences are available for XRQ and PSC8 (https://www.heliagene.org/) and the former genotype is included in the SAM population.

We find that heterosis in sunflower is mainly caused by expression complementation in hybrids of genes that are missing in one or the other parental lines. This mechanism can account for the maintenance of heterosis in different environments, likely accounting for the greater environmental yield stability of hybrid crops.

## Materials and Methods

We report on two main experiments. First, we describe the phenotyping, genotyping, and GWAS analyses of the SAM population across three environments. This allows us to examine the genetic bases of heterosis and to show that the results are repeatable across a broad range of environments. We then describe a smaller scale greenhouse experiment to generate transcriptome data and to observe phenotypic responses in XRQ and PSC8, as well as their hybrid INEDI. We chose these three genotypes for two reasons. Most importantly, chromosome level reference genomes have been generated for XRQ and PSC8 (https://www.heliagene.org/), which permitted us to characterize PAVs and to know which PAVs are expected to be complemented in INEDI. In addition, a previous study [38] showed that INEDI exhibits heterosis under both control and drought conditions.

### Phenotyping and genotyping of the SAM population

The SAM population includes 288 sunflower cultivars that represent circa 90% of the allelic diversity in the cultivated sunflower gene pool [51], [52]. The population includes a mix of inbred and open-pollinated lines, as well as oilseed and confectionary varieties. Here we analyze phenotypic data that were collected for the population in the summer of 2010; experimental design and GWA analyses of two traits (branching and days to flower) have been described previously [51], [53]. Phenotyping was conducted at three locations: Watkinsville, GA and Ames, IA in the USA and Vancouver, BC, in Canada using 271 lines that were available at the time. A total of 19,512 seeds were planted across the three sites using an alpha lattice design (12 seeds per plot × 271 lines × 2 replicate sites × 3 locales). Between two and four plants per plot were phenotyped for 16 traits (Table 1), 14 of which are presented here for the first time. In addition, we surveyed the literature for papers that report on phenotypic data for hybrid and parental sunflower lines in order to determine which traits are likely to display heterotic responses (e.g., [50]), as well as the relative strength of such responses (Table 1).

**Table 1:** Phenotypic traits assayed and predicted phenotypic expression in hybrids. Traits phenotyped in XRQ, PSC8, and INEDI by [38] are in italics.

**Trait**	**Phenotyping assay**	**Stage**^a^	**Heterosis?**^b^
*Leaf area*	First fully expanded leaf scanned and area calculated with ImageJ	R4/R5	weak
Leaf weight	Dry weight of first fully expanded leaf	R4/R5	weak
*Specific leaf area*	Ratio of leaf area/dry weight	R4/R5	no
Days to flower	Calculated from the planting date	R5.1	no
*Height*	Distance from soil line to point on stem connecting to terminal flower head	R5.1	strong
Head diameter	Diameter of terminal flowering head averaged across N/S orientation and E/W orientation	R5.1	weak
Anthocyanins in disk florets	Intensity scored on a scale of 1 (yellow) to 9 (darkly pigmented)	R5.1	no
Anthocyanins in stigmas	Intensity scored on a scale of 1 (yellow) to 9 (red)	R5.1	no
Biomass	Dry weight of all above ground plant material	R9	moderate
Stem weight	Dry weight of stem	R9	moderate
Head weight	Dry weight of terminal flowering head	R9	strong
*Stem diameter*	Diameter at base of dry stem averaged across N/S orientation and E/W orientation	R9	moderate
Seed weight	Weight of 100 seeds in grams	R9	weak
Seed size	Length × width averaged over 5 seeds	R9	weak
Oil percentage	Oil concentrations measured by pulsed nuclear magnetic resonance (NMR) analysis	R9	no
Branching	See Nambeesan et al. (2015)	All	no

a Developmental stages from [55].

b Traits were classified as heterotic or not based on reports from the literature (e.g., [54]).

The SAM population was previously sequenced (5-25x depth) as described in Hübner et al. [56]. The sequence data were aligned against the HA412-HOv2 reference genome for variant calling using the pipeline described in Todesco et al. [57]. Briefly, we trimmed the reads using Trimmomatic v0.36 (usadellab.org/cms/?page = trimmomatic) to remove Illumina adapters and poor quality sequences, and the filtered reads were then aligned to the HA412-HOv2 reference genome using NextGenMap v0.5.3 (github.com/Cibiv/NextGenMap). Variant calling was performed following the Genome Analysis Tool Kit best practices (https://gatk.broadinstitute.org/hc/en-us) on non-repetitive regions on 17 chromosomes of the reference genome. In order to remove low-quality variants, we conducted Variant Quality Score Recalibration (VQSR) using the 20 samples with the highest sequencing depth in the SAM population as a “gold set”. The variant data set was filtered to retain only bi-allelic SNPs in the 90% tranche with minor allele frequency > 0.05 and genotyping rate > 50% for GWA analyses.

Detection of gene PAVs in the SAM population used read depth values from Illumina reads aligned to the HA412-HOv2 reference genome. For each 100 bp non-overlapping window across the genome, we measured the read depth in each sample, ignoring mapping quality. We then classified each window as either being present (depth > 0), or absent (depth = 0). We recognize that given modest read depth in some samples, some regions are not sequenced due to chance, as well that some regions are classified as absent due to sequence divergence preventing accurate alignment, but accept this as noise in our genotyping. Our initial PAV table included all 100 bp windows, including invariable windows present or absent in all samples. We therefore treated the PAVs as haploid genotypes, and filtered the table to retain only windows with minor allele frequency ≥ 5%.

### GWA analyses

We initially conducted a standard GWA analysis using EMMAX (https://genome.sph.umich.edu/wiki/EMMAX), in which we searched for associations between PAVs, and phenotypic data for the 16 traits in Table 1. To identify relatedness between samples, we used a SNP table for the SAM population filtered to include only biallelic sites sequenced in ≥ 50% of samples with a minor allele frequency ≥ 5%, and further pruned for linkage disequilibrium (LD < 0.2 in 500 kb windows) using bcftools (https://samtools.github.io/bcftools/bcftools.html) and SNPrelate (https://github.com/samtools). This SNP dataset was used to create a PCA of samples using SNPrelate, as well as a kinship matrix using EMMAX. The first two principal components were used as covariates in EMMAX, as well as the kinship matrix to control for relatedness between samples. Additionally, we included average gene read depth for each sample as a covariate to control for the difference in read depth between samples. Trait values were manually inspected and extreme outliers, likely from data recording errors, were removed. Trait values were averaged per line, so each line was only represented once in the GWA. To run PAVs within EMMAX, we treated each sample as a diploid homozygote for either the present or absent allele. We recognize that this approach lacks power to detect PAVs with only mildly deleterious effects on phenotype, and PAVs with strong deleterious effects are likely to have been purged from the cultivated gene pool. Therefore, we examined the direction of effects of all quantitative trait loci (QTLs) detected at a less conservative significance threshold (p < 0.05). While some QTLs detected using such an approach are likely to be false positives, we can ask whether the proportion of PAVs with positive or negative effects differs from the random expectation of 0.5. We predicted that “absence” alleles at PAVs are more likely to reduce values of heterosis-related traits than “presence” alleles. Likewise, we expect such a pattern to be observed most strongly for those traits that exhibit moderate to strong heterosis. We also applied the same approach to the subset of genes that showed expression complementation in INEDI (see below), with the goal of further linking transcriptomic patterns to heterotic phenotypes.

Under the dominance model, other kinds of deleterious mutations are predicted to contribute to heterosis as well, but effect sizes are likely to be small. Therefore, we surveyed the genome for stop mutations in protein-coding genes, since we expected such mutations to be most similar to PAVs in effect sizes. Based on a recently-refined annotation of the reference genome (https://sunflowergenome.org/), we removed RNA genes, putative pseudogenes (including those without suitable ORF sequences and all genes without introns) and genes with an ORF<75 amino acids, leaving a total of 36,856 genes. Using the filtered set of protein-coding genes, we annotated the SNPs with the program snpEff (https://pcingola.github.io/SnpEff/) and extracted “stop_gained” and “stop_lost” mutations. Only stop codon mutations with MAF > 20% were chosen for further analyses because sites with skewed allele frequencies are likely to generate unreliable GWAS results. We then searched for associations between stop condons and the 16 phenotypic traits listed in Table 1. As with the PAVs, we examined the direction of effects of all stop codon quantitative trait loci (QTLs) detected at p < 0.05 and asked whether the proportion of stop codons with positive or negative effects differs from 0.5. Our hypothesis was the stop codons would disproportionately reduce heterotic trait values, but lack a directional effect on other non-heterotic traits.

### Genomic prediction

The following genomic prediction model was trained with mixed.solve function in R package rrBLUP version 4.6.1 (https://CRAN.R-project.org/package = rrBLUP) for the 16 traits in Table 1 and PAV markers:

y=1β+Zg+ε

where y is a vector of the phenotypic trait (in this case Z-scores of the phenotypic trait); Z is a 286*7541945 incidence matrix containing the allelic states of the markers (Z = {-1, 1}); −1 and 1 represent the absent and present allele, respectively; β is a vector of fixed effects; g is the vector of marker effects; and ε is a vector of residuals.

To evaluate the effects of deleterious mutations, the value of each phenotypic trait (Z-scores) was predicted by the genomic prediction model, in which the direction of PAV loci was allow to vary from 0 to 100 percent random present alleles at 1% intervals for a total of 100 predictions.

To further clarify the effects of deleterious PAVs on phenotype, a linear model (Y ∼ X) was fit, where X is the percentage of present alleles and Y is the corresponding predicted phenotype for each trait. The beta coefficient of X can therefore represent the contribution and direction of deleterious effects of PAVs to heterosis.

A replicated k-fold cross-validation approach with k = 2, 5, and 10 was used to assess the accuracy of the genomic prediction models. Each individual was randomly assigned into k bins. Individuals in k-1 bins (training population) were used to train a prediction model to predict the phenotype in the remaining bin (validation population), and this process was repeated so that individuals in each bin were predicted once. The Pearson correlation between predicted and observed phenotype values in the validation population in each run was calculated. The procedure of defining bins and predicting the value of individuals in each bin was repeated 20 times resulting in k*20 different cross-validation runs for each k scenario. The mean and standard deviation of the Pearson correlation across all runs was used to measure the prediction model's accuracy.

*R*-squared was also calculated in a linear model (observed phenotypes ∼ predicted phenotypes) in each cross-validation run for all k scenarios. The overall average value shows the proportion of the variation in phenotypic data that PAVs can explain in the prediction model.

### Plant material preparation for transcriptomic analyses

Two drought stress experiments were conducted on the Heliaphen phenotyping platform (https://www6.inrae.fr/phenotoul_eng/WHO-we-are/PhenoToul/HeliaPhen) in 2012 and 2013, respectively, using the sunflower inbred lines XRQ (maternal), PSC8 (paternal) and their F_1_ hybrid (INEDI). For both experiments, seeds of the three different genotypes were sterilized with bleach and were germinated on Petri dishes with Apron XL and Celeste solutions (Syngenta, Basel, Switzerland) for three days. Each seedling was transplanted into an individual 20-liter pot. The pots were filled with 10% sand, 40% P.A.M.2 potting soil (Proveen distributed by Soprimex, Chateaurenard, Bouches-du-Rhône, France) and 50% clay, and covered with a 3-mm-thick polystyrene sheet to prevent evaporation. Plants were fertilized with 500 ml of Peter's Professionnal 17–07-27 (0.6 g/l) and Hortilon (0.46 g/l) solution. Twenty-five days after sowing, irrigation on drought-treated plants was stopped. Soil water content was monitored according to the fraction of transpirable soil water (FTSW), which was calculated as described in Marchand et al. [58]. Seedlings were harvested at different time points based on soil FTSW values.

For the 2012 experiment (12S01 – the year of 2012, serre, the 1st trial [59]), leaf tissue was sampled from seven week-old seedlings across a drought stress gradient from least to most stressed: FTSW values of 0.7, 0.55, 0.40, 0.25, 0.12, 0.1). One sample for each genotype was collected at each of the six FTSW levels (3 genotypes × 6 FTSW levels = 18 samples total). For the 2013 experiment (13HP02 – the year of 2013, Heliaphen, the 2nd trial [59]), leaves from three seven week-old seedlings of each genotype were sampled from an extreme drought stress treatment (FTSW = 0.1) and a well-watered control (FTSW = 1.0) treatment (3 genotypes × 3 replicates × 2 treatments = 18 samples total). In both experiments, leaves were removed without their petiole and immediately frozen in liquid nitrogen. Samples were ground using a ZM200 grinder (Retsch, Haan, Germany) with a 0.5-mm sieve. Total RNA was extracted using QIAzol Lysis Reagent following the manufacturer’s instructions (Qiagen, Dusseldorf, Germany). The quantity of RNA was estimated using a ND-1000 spectrophotometer (Nanodrop, Wilmington, DE, USA). RNA quality was checked by electrophoresis on an agarose gel and Bioanalyzer 2100 (Agilent Technologies, Santa Clara, CA, USA).

### Illumina RNA-Seq library preparation

We generated 100 bp paired-end RNA sequencing libraries of each sample (36 libraries total from the two experiments). The libraries were prepared using the TruSeq sample preparation kit (Illumina San Diego, CA, USA) following the manufacturer’s instructions. RNA sequencing was performed on the Illumina HiSeq 2000 by DNAVision (Charleroi, Belgium). FASTQ files from the Illumina sequencing have been deposited in the National Center for Biotechnology Information Sequence Read Archive under BioProject number of PRJNA345532.

### Analyses of gene expression and alternative splicing

**Fig. 1:**
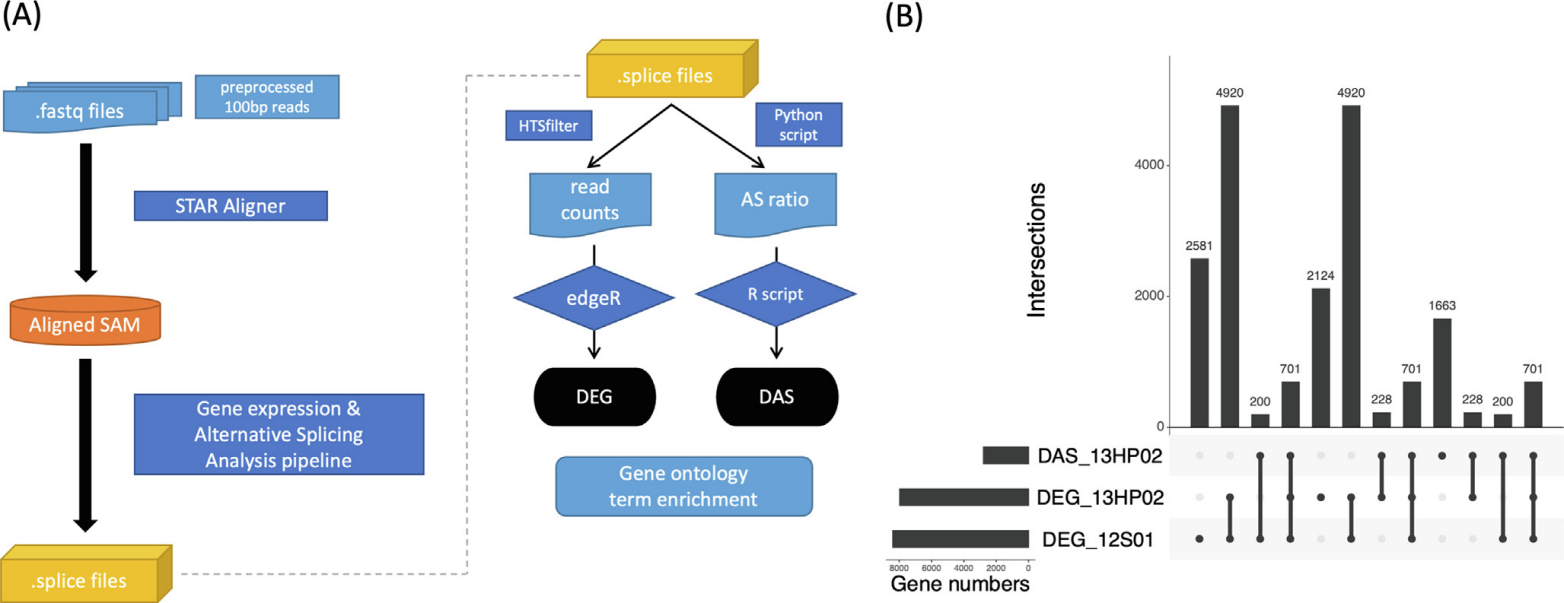
An overview of the short-read sequence analyses for sunflower (*Helianthus annuus*) transcriptomic responses to drought in two experiments. (A) Transcriptome analysis pipeline for short-read sequencing (RNA-Seq). DEG: differentially expressed genes. DAS: differentially spliced genes. (B) An UpSet plot of DEG in 13HP02 (extreme drought vs. well-watered control), DAS (13HP02) and DEG in 12S01 (stress gradient), with false discovery rate (FDR) cutoff 0.01, logFC > 1 or < -1.

The 100 bp paired-end raw reads from the 36 RNA-Seq libraries were assessed for quality using FastQC (bioinformatics.babraham.ac.uk/projects/fastqc), filtered and trimmed using Trimmomatic v0.36 (usadellab.org/cms/?page = trimmomatic), and checked using FastQC again to confirm read quality. Processed reads were then mapped against the XRQ sunflower reference genome [60] using STAR v2.4.2 (github.com/alexdobin/STAR) with non-default parameters (--alignIntronMax 10,000). Analyses of expression levels and alternative splicing (AS) employed a custom pipeline (Fig. 1A) following the approach of Lee and Adams [61]. Read count metrics and AS events were determined by quantifying reads mapped against constitutive and alternatively spliced forms using reference genome gene models [60]. HTSFilter v1.12.0 (https://bioconductor.org/packages/release/bioc/html/HTSFilter.html) was used to remove the weakly expressed genes across all the conditions. Differentially expressed genes (DEGs) were identified using a cutoff set at a false discovery rate (FDR) < 0.01 via edgeR v3.18.1(https://bioconductor.org/packages/release/bioc/html/edgeR.html).

For the AS analysis, in addition to the HTSFiltering (above), we excluded genes with fewer than three reads mapped against alternative isoforms. Alternative splicing events were categorized according to the major classes of alternative splicing events: intron retention (IR), alternative donor (ALTD), alternative acceptor (ALTA), alternative position (ALTP), exon skipping (SKIP), cryptic intron (CRIN), and cryptic exon (CREX). Based on the presence or absence of splice junctions, reads mapping to alternative vs. constitutive isoforms were counted, and their relative abundance or percent splicing index (PSI) was calculated for each sample. Logistic regression using a custom R script (https://github.com/dejonggr/differential_as) was employed to identify differentially spliced genes (DAS) (FDR < 0.01, log_2_FC > 0.25 or log_2_FC < -0.25). UpSetR v1.4.0 (https://cran.r-project.org/web/packages/UpSetR/index.html) was used to visualize the extent of overlap in differentially expressed and differentially spliced genes across the two experiments (Fig. 1B). Because the AS data is in a binomial distribution, a higher log2 fold change underestimates DAS detection.

### Detection of genes exhibiting expression complementation due to PAV

We have newly sequenced reference genomes of both the XRQ (version 2) and PSC8 genotypes (https://www.heliagene.org/). In order to identify genes exhibiting expression complementation due to PAV, we re-mapped the RNA-Seq data of all three genotypes (13HP02) against both reference genomes. We classified genes as expressed if Transcripts Per Million (TPM) > 2 and not expressed if TPM < 0.1. We used the thresholds based on the even distribution of reads on Integrative Genomics Viewer (https://software.broadinstitute.org/software/igv/). Genes expressed in one parent and hybrid, but not the other parent, were blasted (BLASTN, E-value < 1e − 100, sequence identity > 95%, skipping shorter than 200 bp, minimum bit-score of 200) against the reference genome of the latter to ensure they were truly missing. We considered this filtered set of genes as exhibiting expression complementation in hybrids.

### Classification of inheritance patterns in the F_1_ hybrids from the parents

**Fig. 2:**
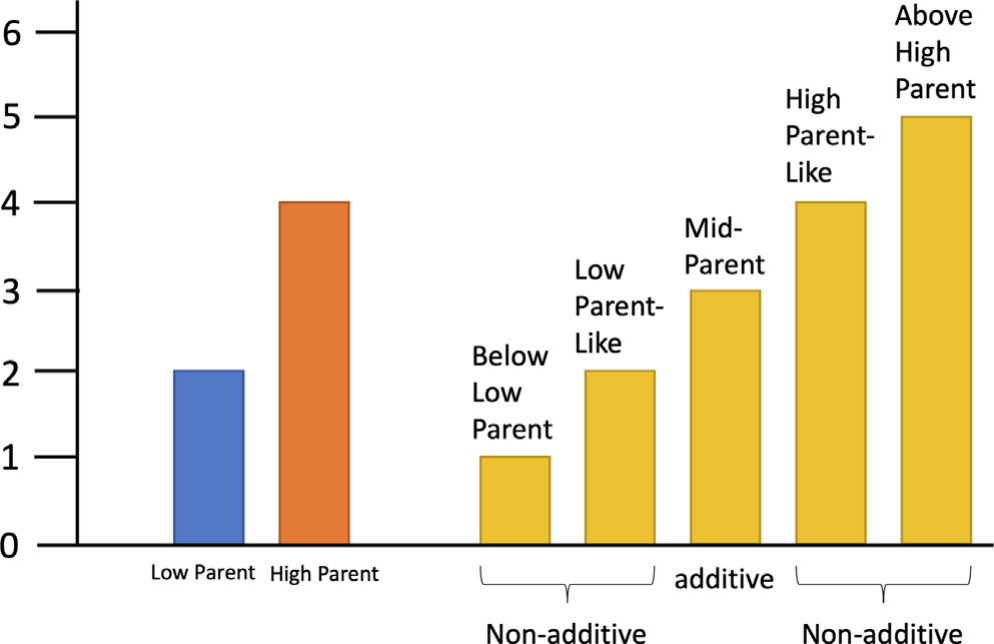
Illustration of non-additivity model. Non-additivity was further categorized based on significant differences (FDR < 0.05) from each parent. The Y-axis represents arbitrary phenotypic levels.

XRQ, PSC8, and INEDI were previously phenotyped by Rengel et al. [38]. Here we re-analyzed the subset of traits that overlapped with those phenotyped for the SAM population (Table 1) to confirm previous observations of heterosis. To identify genes and AS events that may regulate the non-additivity of vegetative performance under control and drought stress, we used the model of hierarchical additive/nonadditive effects on heterosis described by [24] and shown in Fig. 2. First, mid-parent levels of expression and PSI values of alternative splicing were calculated. Then INEDI values were compared to the mid parent values using edgeR for expression and logistic regression analysis for AS. Genes or AS events with FDR p-value < 0.05 were regarded as non-additive and FDR p-values of 0.05 or higher were considered as additive. The genes or AS events that showed non-additivity were further categorized (Fig. 2) as either like the high or low parent (dominance) or as significantly above the high-parent (over-dominance) or significantly below the low parent (under-dominance). We focused on over-dominance and under-dominance of expression and alternative splicing patterns when considering their possible contribution to heterosis, except for genes that were missing in one or the other parent (see below). For AS, we use DAS up-regulation (DASU) to describe over-dominance like AS frequencies in hybrids and DAS down-regulation (DASD) for under-dominance like AS frequencies in hybrids.

### Predicting potential functions of differentially expressed and/or spliced genes

To identify potential functions of genes that were differentially expressed or differentially spliced, we conducted gene ontology (GO) enrichment analysis via ShinyGO v0.61 (http://bioinformatics.sdstate.edu/go61/) (false discovery rate < 0.1). The results are summarized and shown as networks that cluster closely related enriched GO terms. To identify biological pathways or networks of the differentially expressed and spliced genes, the genes were mapped to the pathways in the Kyoto Encyclopedia of Genes and Genomes (KEGG; https://www.genome.jp/kegg/), which is implemented in ShinyGO. The top 10 enriched GO terms and KEGG pathways were selected for further analyses and discussion.

## Results

### GWA analyses in SAM population

**Fig. 3:**
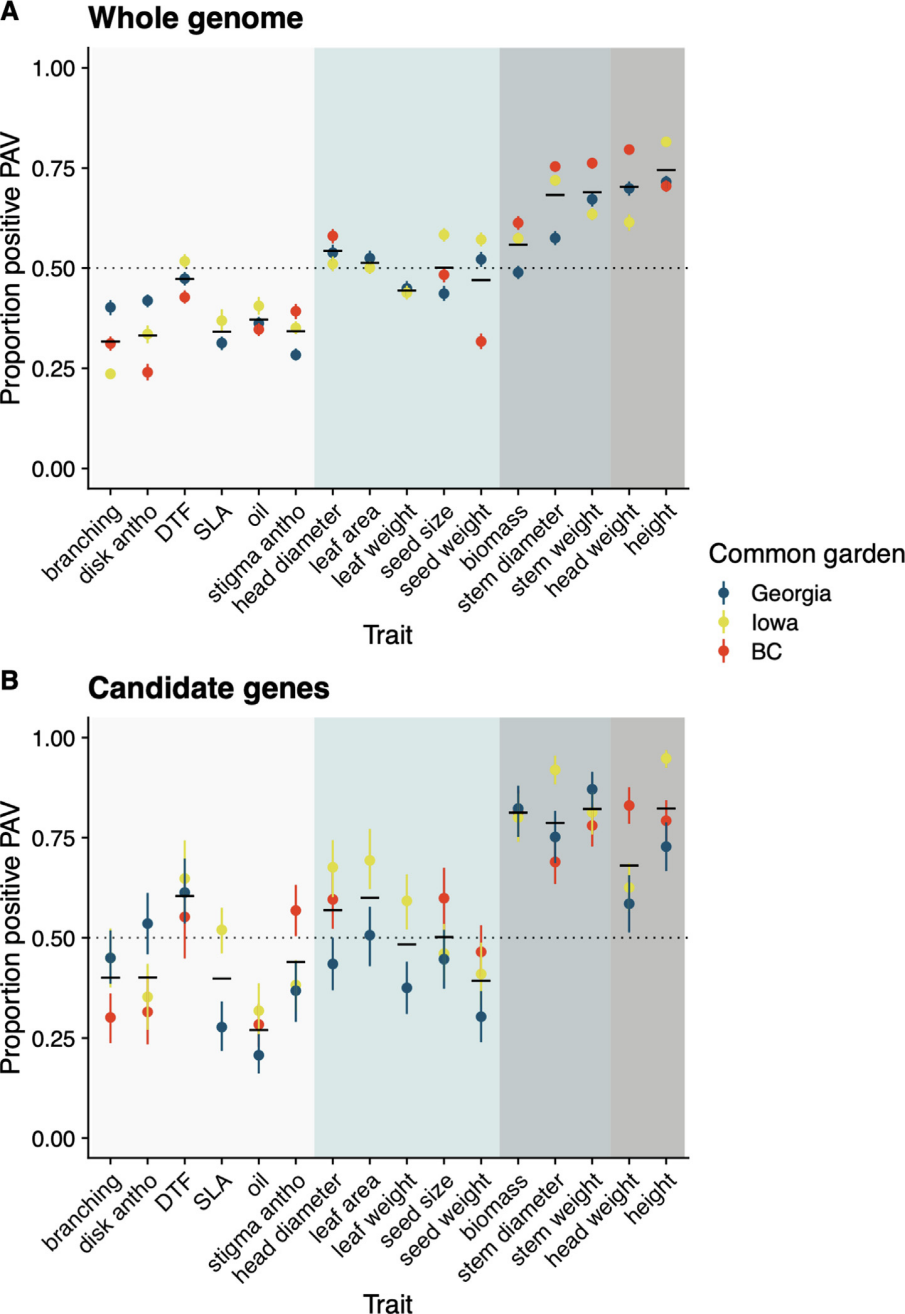
The proportion of presence/absence variants (p < 0.05) with a positive or negative effect on phenotypic traits. (A) All PAVs. (B) Only PAVs that differ between XRQ and PSC8 and show expression complementation in INEDI. 95% confidence intervals from 2000 1 Mb block bootstrap permutations (A) or 2000 individual bootstraps (B). Black horizontal bars indicate mean value for all gardens. Background color indicates no (white), weak (light blue), moderate (blue-grey), or strong (grey) evidence of heterosis.

A total of 7,541,945 PAVs (variable 100 bp windows) was detected at a ≥ 5% minor allele frequency cut off. For many traits, GWA analyses failed to identify significant associations with PAVs when using a q-value correction with an FDR of 10% (Supplementary Fig. S1-3). However, we can detect numerous QTLs (∼170 k to 470 k per trait) at a less stringent threshold (p < 0.05). While we recognize that many of these are likely to be false positives, analysis of the direction of QTL effects at these candidate QTLs indicate that PAVs likely contribute importantly to heterotic phenotypic effects (Fig. 3A). As predicted, the “presence” allele was significantly more likely to increase values of heterosis-related traits than the “absence” allele, and this bias was most extreme in traits with the most evidence of heterosis. For example, plant height and head weight (or yield) are consistently found to be the most strongly heterotic traits in sunflower [54], and they have the largest proportion of “presence” alleles increasing trait values. In contrast, heterosis is generally not reported for traits such as branching, levels of anthocyanin pigmentation, specific leaf area, and oil percentage, and allelic proportions were in the opposite direction. The latter might be a consequence of introgressions from wild species, since we have previously shown that gene losses cluster in introgressed regions [62]. Also, branching maps to such introgressions [63], consistent with this explanation. When we confined our analyses to the subset of PAVs that differ between XRQ and PSC8 and show expression complementation in INEDI, the proportion of PAVs with effects in the predicted direction was even larger (Fig. 3B).

**Fig. 4:**
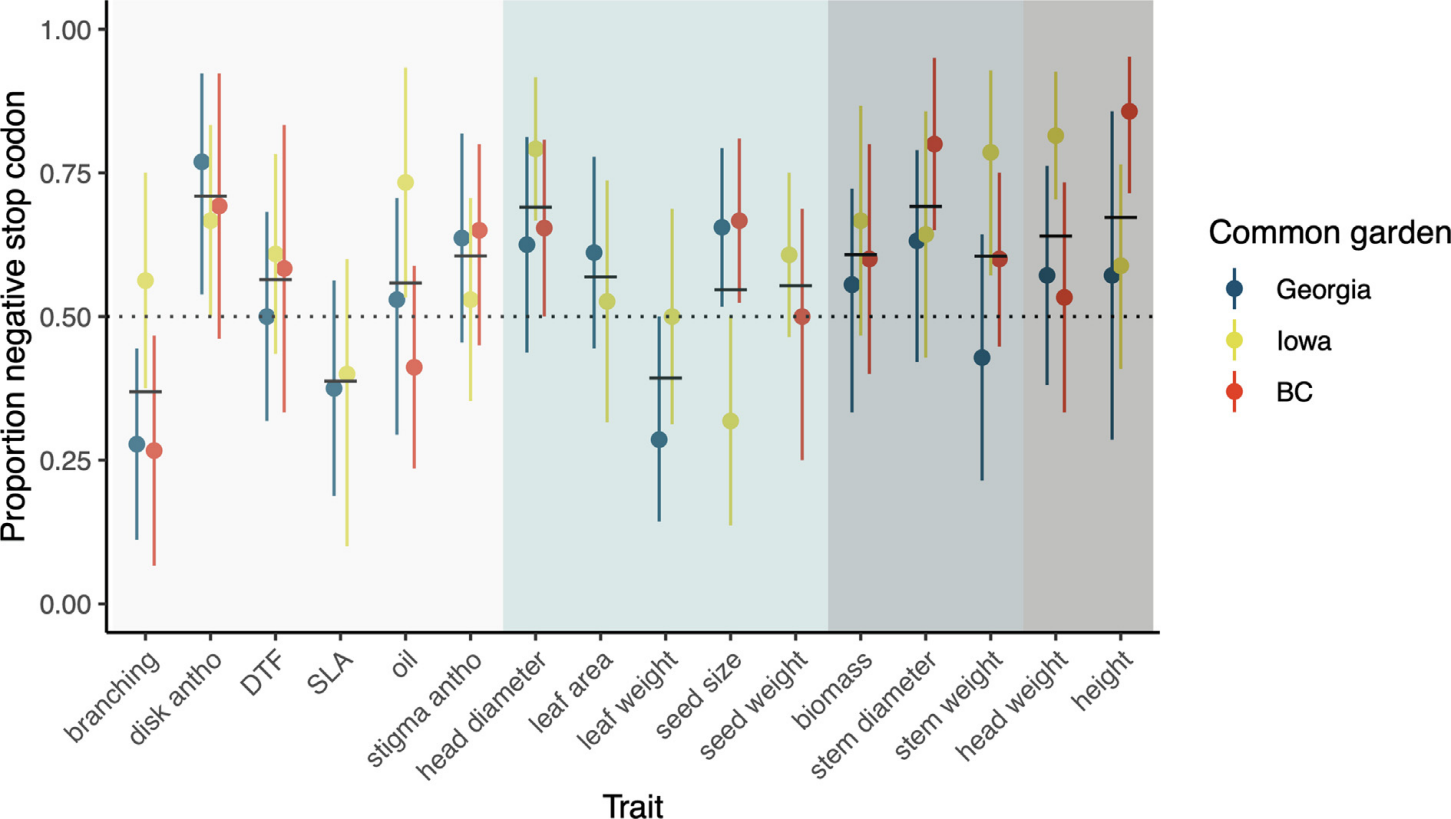
The proportion of stop codon mutations (p < 0.05) with a positive or negative effect on phenotypic traits. Background color indicates no (white), weak (light blue), moderate (blue-grey), or strong (grey) evidence of heterosis.

The impact of stop codons on heterosis is less clear (Fig. 4). In total, we identified 361 stop codon mutations in the SAM population. Between 2% and 8% of these have detectable effect sizes at p < 0.05 in each GWA analyses. In general, heterosis-related traits have a higher proportion of stop codons with negative effects than traits predicted to show weak or no heterosis. However, the excess of negative stop codons is significant in only a portion of the trials, and some traits that exhibit weak or no heterosis (e.g., head diameter and disk anthocyanin content, respectively) also have an excess of stop codons with negative effects. The lack of a clear signal probably stems in part from the relatively small number of stop codons segregating in the SAM population, as well as from the low sequence coverage for some genotypes, which makes it difficult to distinguish between homozygous and heterozygous genotypes. Note that this should not be not an issue for PAVs, because the “presence” allele is expected to be dominant, whereas for stop codons, the wild type allele is generally expected to be dominant.

### Genomic prediction

**Fig. 5:**
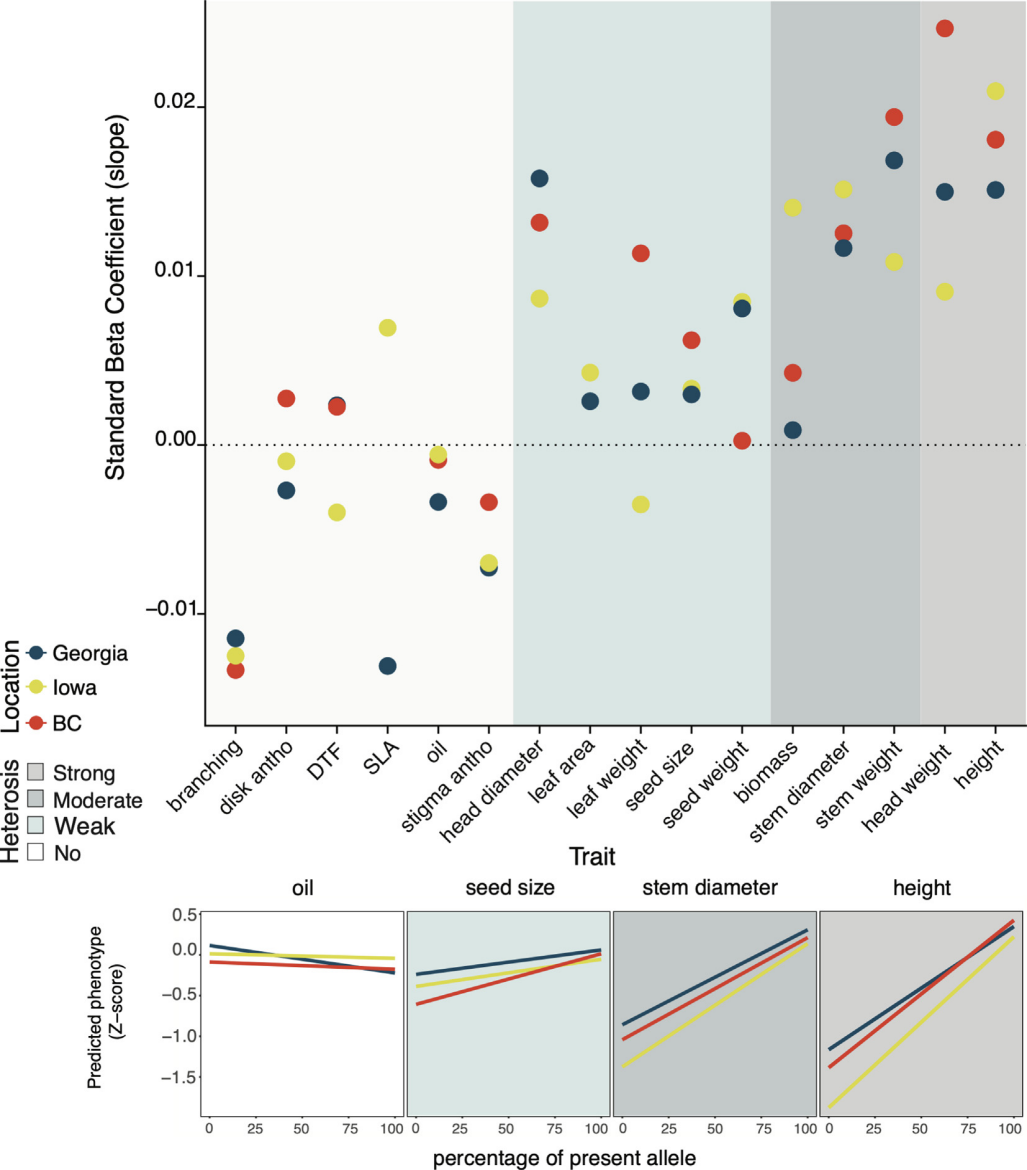
Results from linear regression model where X = the percentage of the present allele and Y = predicted phenotype. (A) The standard beta coefficient of all traits in three common garden experiments. (B) A fitted linear regression line for height, stem diameter, seed size, and oil representing strongly heterotic, moderately heterotic, weakly heterotic, and non-heterotic trait groups, respectively. Background color indicates no (white), weak (light blue), moderate (blue-grey), or strong (grey) evidence of heterosis.

A genomic prediction model was used to simulate the effects of presence/absence variation on trait values. To permit comparisons across traits, prediction models were fitted to Z-score normalized trait values. We found that presence alleles were much more likely than absence alleles to increase values of heterosis-related traits. Therefore, the standard beta coefficient of the linear model (predicted phenotypes ∼ the percentage of present allele) was used to predict levels of heterosis (Fig. 5, Supplementary Figure S4). For this, a positive beta value indicates that presence alleles tend to increase trait values. In general, strongly heterotic traits such as plant height and head weight, and well as the moderately heterotic trait group, show the largest beta coefficients, while the beta coefficients are much smaller for weakly heterotic and non-heterotic traits. One notable exception is head diameter, which was classified from the literature scan as a weakly heterotic trait, but shows a fairly large beta coefficient, suggesting that it probably should have been classified as a moderate to strong heterotic trait. Variation in beta coefficients across different traits is highly consistent across sites, similar to that seen for PAV ratios above (Fig. 3).

Cross-validation was performed for various k-fold scenarios, resulting in different sizes of training sets with each value of k. However, prediction accuracy was relatively stable for different k scenarios at each location (Table 2), ranging from 0.878 (branching-BC) to 0.097 (biomass-Georgia).

**Table 2:** Genomic prediction model accuracy based on a replicated k-fold cross-validation approach.

	k = 2			k = 5			k = 10			Average			
phenotype	**GA**^a^	**IA**^b^	**BC**^c^	**GA**^a^	**IA**^b^	**BC**^c^	**GA**^a^	**IA**^b^	**BC**^c^	**GA**^a^	**IA**^b^	**BC**^c^	**All**
biomass	0.097	0.301	0.318	0.115	0.352	0.343	0.115	0.37	0.361	0.109	0.341	0.341	0.264
branching	0.823	0.841	0.858	0.837	0.858	0.872	0.846	0.864	0.878	0.835	0.854	0.869	0.853
disk_antho	0.244	0.261	0.141	0.395	0.341	0.181	0.446	0.344	0.238	0.362	0.315	0.187	0.288
dtf	0.489	0.406	0.226	0.55	0.443	0.264	0.555	0.462	0.264	0.531	0.437	0.251	0.407
head_diameter	0.688	0.649	0.497	0.698	0.67	0.514	0.71	0.684	0.529	0.698	0.668	0.513	0.626
head_weight	0.751	0.51	0.75	0.773	0.53	0.762	0.773	0.532	0.77	0.766	0.524	0.761	0.684
height	0.499	0.453	0.44	0.547	0.484	0.478	0.549	0.494	0.499	0.532	0.477	0.473	0.494
leaf_area	0.475	0.565	–	0.5	0.586	–	0.504	0.598	–	0.493	0.583	–	0.538
leaf_sla	0.473	0.217	–	0.49	0.278	–	0.502	0.314	–	0.489	0.27	–	0.379
leaf_weight	0.498	0.569	0.675	0.509	0.606	0.697	0.51	0.61	0.705	0.506	0.595	0.692	0.598
oil	0.739	0.616	0.726	0.763	0.649	0.751	0.768	0.656	0.754	0.757	0.64	0.744	0.714
seed_size	0.803	0.84	0.819	0.825	0.855	0.838	0.833	0.854	0.846	0.82	0.85	0.834	0.835
seed_weight	0.72	0.733	0.637	0.746	0.751	0.666	0.747	0.767	0.689	0.738	0.75	0.664	0.717
stem_diameter	0.484	0.506	0.568	0.494	0.539	0.578	0.506	0.55	0.586	0.495	0.532	0.577	0.535
stem_weight	0.513	0.399	0.501	0.528	0.442	0.518	0.539	0.456	0.529	0.527	0.432	0.516	0.492
stigma_antho	0.335	0.356	0.323	0.416	0.411	0.381	0.432	0.42	0.424	0.394	0.396	0.376	0.389

a Georgia.

b Iowa.

c British Columbia.

Model accuracy can be affected by trait heritability, size of training population, and marker density [64]. Since there were no differences in marker density or training population size, variation in prediction model accuracy most likely results from differences in trait heritability and in the proportion of trait variation explained by PAVs (Table 3).

**Table 3:** Proportion of phenotype variation explained by presence absence variation in the genomic prediction model.

phenotype	GA^a^	IA^b^	BC^c^	Average
biomass	0.109	0.354	0.348	0.27
branching	0.842	0.86	0.873	0.858
disk_antho	0.408	0.35	0.21	0.323
dtf	0.548	0.451	0.262	0.42
head_diameter	0.702	0.675	0.518	0.632
head_weight	0.77	0.528	0.767	0.688
height	0.542	0.484	0.484	0.503
leaf_area	0.497	0.588	–	0.543
leaf_sla	0.496	0.29	–	0.393
leaf_weight	0.506	0.603	0.699	0.603
oil	0.762	0.647	0.75	0.72
seed_size	0.827	0.852	0.839	0.839
seed_weight	0.743	0.76	0.679	0.727
stem_diameter	0.5	0.544	0.58	0.541
stem_weight	0.531	0.447	0.527	0.502
stigma_antho	0.412	0.409	0.398	0.406

a Georgia.

b Iowa.

c British Columbia.

### RNA-Seq data

We generated ∼ 1.8 billion 100 bp paired-end reads from 36 RNA-Seq libraries from three genotypes (PSC8, XRQ, and their F_1_ hybrid INEDI). As detailed above, these libraries were developed from seedling leaf tissue in both control and drought treatments and across two independent experiments (12S01 and 13HP02). After filtering out low-quality reads and genes with low constant expression levels, 417 million clean Illumina mRNA reads were obtained for 35,278 genes (an average of 11.6 million reads per sample).

### Heterosis for morpho-physiological traits in INEDI

Re-analyses of the morpho-physiological trait data from Rengel et al. [38] confirmed previous observations [65]; INEDI exhibits heterosis in vegetative performance (plant height, stem diameter, third-leaf area, and total leaf area) under control and drought stress, but not for specific leaf area, when compared with the parental lines, PSC8 and XRQ (Suppplementary Figure S5). Notably, INEDI significantly out-performed the parental lines in almost every category under control conditions and was significantly taller than both parental lines under drought stress (Wilcoxon rank-sum test, p < 0.05). For the other categories under drought stress, INEDI was above the mid-parent mean, but not significantly larger than the high parent.

### Expression complementation of PAVs

The GWA analyses described above indicate that absence alleles typically reduce the performance of sunflower cultivars. Complementation of such PAVs therefore offers a simple explanation for heterosis. To confirm predicted expression complementation, we mapped the transcriptome data against both parental reference genomes and identified genes exhibiting presence absence variation in gene expression. Mapping on the XRQ genome, we found 170 XRQ genes that were expressed in XRQ and INEDI but not in PSC8 in the control treatment, and 57 XRQ genes that were expressed in XRQ and INEDI but not in PSC8 under drought stress. Mapping on the PSC8 genome, we found 180 PSC8 genes that were expressed in PSC8 and INEDI but not in XRQ under control conditions, as well as 200 PSC8 genes that were expressed in PSC8 and INEDI but not in XRQ in the drought treatment. We did BLAST searches of those genes against the parental genome lacking expression to see if the genes were truly missing from the genome (Supplementary Table S1). Forty-three genes identified from the control treatment and 12 from the drought treatment for XRQ (10 genes overlapped between treatments), and 43 genes identified from the control and 56 genes from the drought treatment for PSC8 (31 genes overlapped) were confirmed to be true presence/absence polymorphisms. Expression complementation of these genes in INEDI is consistent with the dominance model of heterosis. The overlap in genes exhibiting expression complementation between the control and drought conditions further suggests that the dominance model could account for the partial maintenance of heterosis under drought conditions.

Genes showing PAV and expression complementation were not significantly enriched in any GO terms, although marginally significant enrichment was observed for the “response to heat” (GO:0034605) category in both the control (FDR = 0.093) and drought treatment (FDR = 0.058).

### Differential expression in response to drought stress

Transcriptome data from the 13HP02 experiment, which consisted of an extreme drought stress treatment (FTSW = 0.1) and a well-watered control (FTSW = 1.0), resulted in detection of 7,973 differentially expressed genes (FDR < 0.01) responding to drought stress. Likewise, analyses of transcriptomic responses to the drought stress gradient in the 12S01 experiment identified 8,402 differentially expressed genes, of which approximately two thirds (5,621 genes) were also found on the 13HP02 DEG list (Fig. 1B). The genes in common between the two datasets were used for downstream analyses.

Of the genes responding to drought stress, 2,523 were up-regulated, and 3,098 were down-regulated. For the up-regulated DEGs (Supplementary Table S2A), the most significantly enriched GO terms were Response to chemical (GO:0042221, FDR = 4.31E-21) in biological process, Nuclear lumen (GO:0031981, FDR = 1.06E-14) in cellular component, and Cation binding (GO:0043169, FDR = 9.70E-09) in molecular function. These genes most significantly (FDR = 3.09E-4) mapped to Metabolic pathways (map01100) and Protein processing in endoplasmic reticulum (map04141), followed by Spliceosome (map03040, FDR = 3.58E-4).

Down-regulated DEGs (Supplementary Table S2B) were mostly enriched in GO terms of Photosynthesis (GO:0015979, FDR = 1.03E-24) in biological process, Chloroplast (GO:0009507, FDR = 2.44E-82) in cellular component, and Transferase activity (GO:0016740, FDR = 1.01E-14) in molecular function. Similar to the up-regulated genes, the most significantly mapped KEGG pathways for down-regulated genes included Metabolic pathways (map01100, FDR = 1.64E-37). In addition, a number of photosynthesis related pathways were significantly mapped for down-regulated genes.

Overall, our results suggest that major responses of sunflower to drought stress include up-regulation of genes involved in stimulus responses and down regulation of photosynthetic pathways, as well as some metabolic processes such as carbon metabolism.

### Differential AS in response to drought stress

Focusing on the 13HP02 data because of its greater replication, we employed a custom pipeline of Python (v2.7.3) scripts (including the filtering; [66]) to discover 33,186 alternative splicing events in 11,630 genes. The majority of these events were classified as intron retention (30,615 events), followed by alternative acceptor (859 events), alternative donor (743 events), alternative position (213 events), exon skipping (602 events), and other (153 events). The latter represent complex and less easily categorized AS events.

A total of 3,493 differential splicing events (2,980 up-regulated and 513 down-regulated) were found as responses to drought stress in sunflower cultivars. Up-regulated DAS (Supplementary Table S2C) were most significantly enriched in Photosynthesis (GO:0015979, FDR = 7.96E-42) in biological process, in Chloroplast (GO:0009507, FDR = 2.47E-167) in cellular process, and in Protein domain specific binding (GO:0019904, FDR = 5.23E-16) in molecular function. The Metabolic pathways category (map01100, FDR = 4.76E-47) was most significantly mapped from the KEGG pathway database.

On the other hand, down-regulated DAS (Supplementary Table S2D) were mostly enriched in Small molecule metabolic process (GO:0044281, FDR = 3.24E-07) in biological process, Chloroplast (GO:0009507, FDR = 1.07E-14) in cellular process, and Nucleotide binding (GO:0000166, FDR = 2.49E-4) in molecular function. These genes also mapped most significantly on Metabolic pathways (map01100, FDR = 2.94E-08).

Differential splicing appears to negatively correlate with differential expression, most notably for photosynthesis related genes. This implies that DAS acts to reinforce differential expression patterns. That is, up-regulation of AS for photosynthesis related genes increases the frequency of non-functional splice forms, resulting in fewer functional transcripts overall, consistent with down-regulation of the same genes seen in the expression data.

### Non-additive gene expression in the F_1_ under control conditions

Analyses of gene expression data from the 13HP02 experiment, which included a well-watered control treatment (FTSW = 1.0), resulted in detection of 3,003 genes (FDR < 0.05) in INEDI with significant expression differences from its parents. For analyses of gene expression patterns potentially associated with heterosis, we focused on the genes exhibiting over-dominant (550 genes) or under-dominant (89 genes) expression levels.

**Fig. 6:**
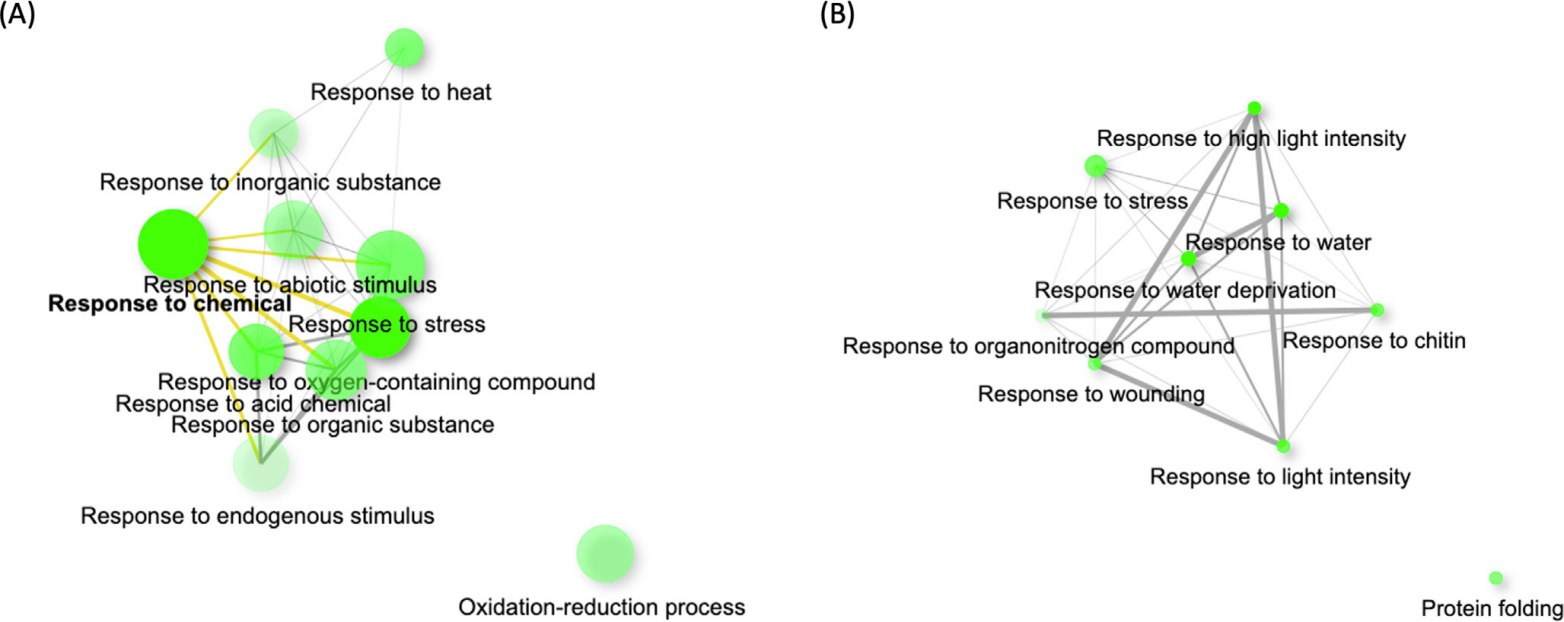
Gene ontology enrichment results using over-dominant genes. Each node represents an enriched GO term. Related GO terms are connected by a line. The thickness of a line displays percentage of overlapping genes. The size of green circle represents number of genes. (A) Over-dominant genes enriched in biological process GO terms under control condition. (B) Over-dominant genes enriched in biological process GO terms under drought condition.

The GO enrichment and KEGG pathway mapping results for these genes are listed in Supplementary Table S2E-F. Over-dominant genes were most significantly enriched in Response to chemical (GO:0042221, FDR = 1.03E-15) in biological process (Fig. 6A), Cell periphery (GO:0071944, FDR = 5.58E-3) in cellular component, and reversible chemical reaction related GO terms (Oxidoreductase activity, GO:0016491, FDR = 1.21E-09 was most significant) in molecular function. In terms of pathways, these genes most significantly mapped to Biosynthesis of secondary metabolites (map01110, FDR = 1.74E-09).

Under-dominant genes were also enriched in the GO term Response to acid chemical (GO:0001101, FDR = 1.92E-3) in biological process, chloroplast-related GO terms (Chloroplast thylakoid membrane, GO:0009535, FDR = 5.92E-08, was most significant) in cellular component, and Ion transmembrane transporter activity (GO:0015075, FDR = 2.63E-4) in molecular function. These genes most significantly mapped to Photosynthesis (map00195, FDR = 4.26E-3) and Metabolic pathways (map01100, FDR = 4.26E-3).

Taken together, both up-regulation and down-regulation of stimulus-related and metabolism-related genes may indicate that fine tuning of responses to environmental changes are associated with the heterosis. Alternatively, such changes might reflect neutral or slightly deleterious consequences of bringing two genomes together [22]. The down-regulation of photosynthesis activity was initially puzzling, but possibly relates to the age of the plants employed in the 13HP02 experiment (seven weeks). By this point, growth of the hybrid plants likely had begun to slow because of resource limitations. This possibility is further supported by analysis of the 12S01 experiment, in which plants were harvested after four weeks, and no such down-regulation of photosynthesis pathways was seen.

### Non-additive gene expression in the F_1_ under drought stress

For analyses of gene expression patterns potentially associated with heterosis, we focused on the genes exhibiting over-dominant (27 genes) or under-dominant (62 genes) expression levels. The GO enrichment analysis results and KEGG pathway mapping results are in Supplementary Table S2G-H.

Genes showing over-dominant expression under drought stress were enriched in GO terms of Response to water deprivation (GO:0009414, FDR = 3.90E-2) in biological process (Fig. 6B), Cytosol (GO:0005829, FDR = 1.23E-2) in cellular component, and Chaperone binding (GO:0051087, FDR = 5.44E-3) in molecular function. They did not significantly map to any of the KEGG pathways.

Genes with under-dominant expression under drought stress were enriched in metabolism and biosynthesis-related GO terms (thiamine biosynthetic process, GO:0009228, FDR = 1.91E-2, was most significant) in biological process, but they were not enriched in any other GO category. The genes were mainly significantly mapped on metabolism-related KEGG pathways (Thiamine metabolism, map00730, FDR = 2.63E-3, was most significant).

Up-regulating drought responsive processes and down-regulating unnecessary metabolic processes under drought stress appears to be associated with the maintenance of heterosis under drought.

### Differential splicing correlates with differential expression in hybrids

Under control conditions, we identified 546 DASU and 35 DASD in INEDI that were significantly different from its parents. DASU (Supplementary Table S2I) were mainly enriched in GO terms of response to stimulus (Response to abiotic stimulus, GO:0009628, FDR = 4.05E-16 and Photosynthesis, GO:0015979, FDR = 2.34E-15 were most significant) in biological process, and Chloroplast (GO:0009507, FDR = 1.96E-58) in cellular component, and Protein domain specific binding (GO:0019904, FDR = 1.38E-3) in molecular function. They were most significantly mapped on metabolic pathways (map01100, FDR = 1.81E-23).

DASD (Supplementary Table S2J) were enriched in nucleotide biosynthetic process related GO terms (e.g., ATP biosynthetic process related GO terms, FDR = 3.91E-2) in biological process, Chloroplast (GO:0009507, FDR = 1.40E-06) in cellular component, and Proton-transporting ATP synthase activity, rotational mechanism (GO:0046933, FDR = 1.61E-2) in molecular function. The DASD in the control treatment were not significantly mapped on any KEGG pathways.

We identified 3,493 DAS (2980 up-regulated AS, 513 down-regulated AS) that were significant (FDR < 0.01) in the drought treatment. Of these alternative splicing events, we found 141 DASU and 46 DASD in INEDI compared to its parents. DASU under drought (Supplementary Table S2K) were enriched in Response to abiotic stimulus (GO:0009628, FDR = 6.12E-12) in biological process, Chloroplast (GO:0009507, FDR = 2.64E-08) in cellular component, and Water transmembrane transporter activity (GO:0005372, FDR = 3.77E-3) in molecular function. In the KEGG pathway analysis, Metabolic pathways (map01100) was most significantly mapped (FDR = 8.44E-08).

DASD under drought (Supplementary Table S2L) were enriched in Oxylipin metabolic process (GO:0031407, FDR = 2.23E-2) in biological process, Chloroplast thylakoid (GO:0009534, FDR = 5.80E-05) in cellular component, and Oxidoreductase activity (GO:0016491, FDR = 4.63E-3) in molecular function. Among the KEGG pathway maps, Carbon metabolism (map01200) was most significantly mapped (FDR = 1.88E-3).

### Relationship between levels of gene expression and alternative splicing

**Fig. 7:**
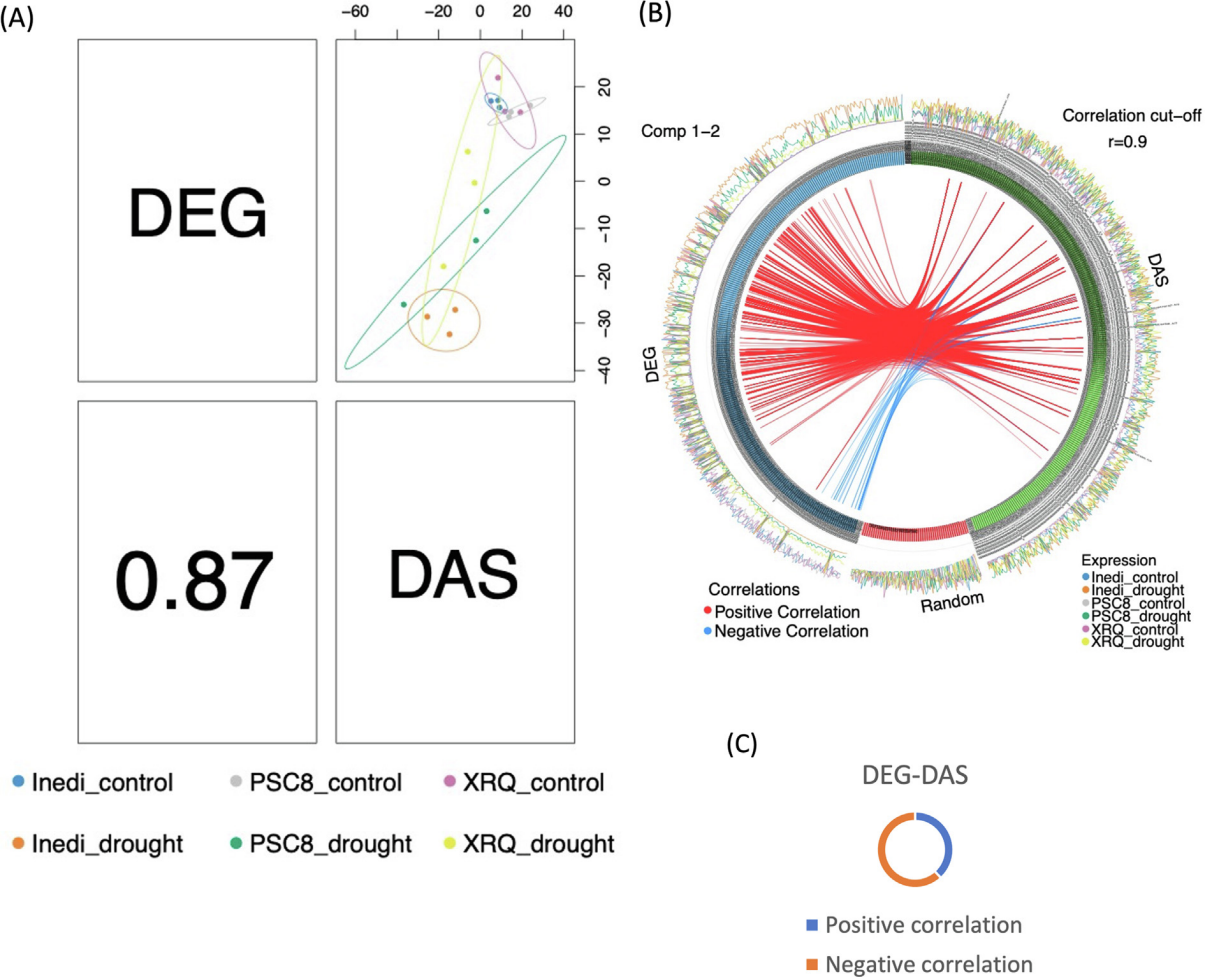
Sparse partial least squares (sPLS)-based classification of the different samples corresponding to the PSC8, XRQ, and INEDI genotypes, considering the changes in gene expression and alternative splicing. (A) A plot displaying the correlation between the DEG and DAS. The lower triangular panel indicates the Pearson's correlation coefficient, the upper triangular panel the scatter plot. (B) A circos plot between the top 500 DEG and top 500 DAS under drought stress. 100 randomly selected genes were included as a control. (C) A pie chart of direct correlations between DEG and DAS.

We employed sPLS regression to extract correlated information between the gene expression and AS data sets. Output from sPLS includes a set of components and loading vectors similar to that produced by principal components analysis. Using the top 500 DEG and DAS genes, we found a clear distinction between control and drought-stressed samples by sPLS components 1 and 2 with respect to expression and AS patterns, but not between genotypes (Supplementary Figure S6). Furthermore, as seen in Fig. 7A, the first component from DEG is highly correlated (Pearson's correlation coefficient = 0.87) to DAS. When we combine the two datasets, we see greater discrimination between genotypes within the drought treatment, particularly for INEDI (orange dots) (Fig. 7A). This suggests that a combination of transcriptional and post-transcriptional information may provide a better explanation of how INEDI’s unique responses are established than analyzing each dataset separately. We also tested for correlations between differential expression under drought and AS frequency, which are displayed by circosPlot in Fig. 7B. When we compare DEG to DAS with the same genes, the majority (65%) of correlations were negative (Fig. 7C). This indicates that stress responsive gene expression is largely negatively correlated with AS frequency, implying that AS often acts to reinforce expression responses (discussed above)**.**

## Discussion

### Gene expression complementation in hybrids contributes to heterosis in sunflower

Despite the importance of heterosis in plant and animal breeding, and the attention given to it by scientists, its mechanistic basis remains surprisingly murky. This is partly because there is no single, universal cause of heterosis [7], [8]. In addition, it can be difficult to reconcile the results from functional studies of heterotic phenotypic traits with classic quantitative genetic models. Lastly, many studies confound correlates of heterosis with causation. In this study we combined transcriptomic analyses with a GWA approach to explore both the underlying cause of heterosis and its consequences at the transcriptome level.

Our results suggest that expression complementation of PAVs is an important contributor to heterosis, consistent with the dominance model of heterosis. GWA analyses and genomic prediction models showed that for most PAVs, the “absence” allele reduced values of strongly heterotic traits, but the same pattern was not observed for traits with little or no evidence of heterosis. Furthermore, this pattern was strengthened in the specific PAVs that showed expression complementation in INEDI. Such PAVs are specifically in coding sequences, which presumably are more likely to have phenotypic effects than the rest of the genome.

In contrast, we failed to find strong evidence that stop codons contribute to inbreeding depression (the flip side of heterosis). This is partly due to the small number of stop codons segregating in the SAM population (a few hundred versus > 7 million PAVs). However, stop codons were also less likely than PAVs to have effects in the predicted direction.

There are limitations to our approach using reference aligned read depth as a proxy for PAVs. In this case, we are assaying absence compared with the HA412HOv2 reference genome, which means that the HA412HO sample has the highest proportion of present alleles. It’s important to note that although we find presence alleles are generally positive for heterotic traits, HA412HO has average values for heterotic traits despite its higher counts of presence alleles due to reference bias (Figure S7). This counters a possible explanation for our pattern that absence alleles represent divergence from a heterotically ideal reference sample, HA412HO.

The measured effect of PAVs is due to both the direct effect of PAVs themselves, and also to the underlying effects of linked alleles, as in all GWA. The SAM population includes samples with significant wild introgression bringing with it changes in gene content [56], [62]. For PAVs resulting from introgression, absence alleles are likely linked to larger blocks of wild alleles. This is particularly true for the branching phenotype, which is caused by a large wild introgression on chromosome 10 [63]. Using our PAV GWA, we see a large peak over the same region (Figures S1-S3). While the branching phenotype may, or may not, be caused by PAVs, the large peak is due to linkage between the causative QTL and PAVs found in the introgression. Because wild introgressions are enriched for missing genes [62], the association between PAVs and heterosis may be partially or primarily driven by linkage drag from wild introgressions. Follow up work should directly test the association between introgression and heterotic trait values in the SAM population.

Surprisingly, we were unable to find previous studies in the literature that directly linked expression complementation of PAVs to heterotic phenotypes. We suspect that there are several reasons for this. First, the vast majority of PAVs are likely weakly deleterious, so their impact on heterotic traits will not be detected in standard GWA experiments. Second, most studies have failed to distinguish between the predicted effects of the “absence” versus “presence” alleles on trait variation. Lastly, few studies have combined both GWA and transcriptome analyses. By employing a relaxed significance threshold for QTL discovery, and tabulating the direction of QTL effects, we were able show that numerous PAVs contribute to deleterious load and were complemented in an F_1_ hybrid.

Even in maize, where expression complementation has been observed, direct evidence that it is the cause of heterosis remains sparse [17], [67], [68]. For example, expression complementation in F_1_ hybrids of maize cultivars (B73 and Mo17) was reported by Paschold et al. [17], but no direct connection was made to heterotic phenotypic variation. Li et al. [69] conducted a more thorough analysis of expression complementation involving many inbred parental lines, their hybrids, and multiple tissue types. They also explored single parent expression from non-PAVs (differential expression between the parental alleles) and PAVs and showed that expression complementation of the latter was more common in hybrids, but no link was made with heterotic phenotypes. On the other hand, a large-scale genomic selection analysis, also in maize, did link putatively deleterious alleles (as identified by SNP variation in evolutionary constrained regions across the genome) to phenotypic variation and heterosis [70], but expression variation and PAVs were not assayed in that study.

We demonstrated that the majority of genes showing PAV and expression complementation overlap between two different conditions (control vs. drought) in sunflower and that these were enriched for heat-responsive genes. Marcon et al. [67] also found specific single parent expression (SPE) complementation in maize under control and drought conditions, and that the expression pattern of majority of SPE genes was consistent across environments. The stability of expression complementation under different water deficit conditions may account for the maintenance of heterosis under drought.

Our results imply that crosses designed to maximize complementation of PAVs should also maximize heterosis. We have previously shown that PAVs are clustered in introgressions from wild species [62], so complementing such introgressions should be considered as well. Lastly, we note that PAVs appear to be more common in plant than animal pan-genomes [71], perhaps accounting for the stronger heterosis typical of plant relative to animal hybrids [72].

### General responses to drought stress

Using a large-scale transcriptome data set from three different genotypes, we found that DEGs up-regulated in response to drought stress were most strongly enriched for GO categories related to stimulus response, whereas down-regulated DEGs were enriched for genes associated with photosynthesis and metabolic processes. Among the up-regulated genes responding to drought stress in our study is *XERICO*, an E3 ubiquitin-protein ligase that regulates abscisic acid biosynthesis in *Arabidopsis* via ubiquitin-mediated protein degradation [73]. The protein is up-regulated by salt and drought stress in *Arabidopsis* [73]. The *XERICO* homolog in sunflower (HanXRQChr10g0306801) is known to be up-regulated by drought as well [38], an observation that was confirmed in the present study across all three genotypes and both experiments. In another example, HanXRQChr01g0016781 is homologous to AT5G22580 (stress responsive A/B barrel domain family) in *Arabidopsis*. The A/B barrel domain is found in a group of stress response proteins in plants (e.g., *Oryza* and *Populus*; [74]) and makes a stable dimer that is localized in the chloroplast. In the present study, HanXRQChr01g0016781 was up-regulated significantly in all three genotypes in response to drought. Previously, some drought responsive genes in sunflower were analyzed by quantitative reverse transcriptase polymerase chain reaction (RT‐PCR), and they were suggested to be related to response to osmotic stress including ABA responsive pathways [75]. Liang et al. [76] also found that water stress responsive genes were significantly up-regulated by drought stress. Using a sunflower microarray, Roche et al. [77] identified 82 organ-specific differentially expressed genes in response to water stress across genotypes, and notably metabolism related genes were repressed in leaf tissue, also in congruence with our results.

The up-regulation of stimulus/stress responsive genes and down-regulation of unnecessary metabolic processes and photosynthesis were predicted based on results from other crops (i.e., rice [35]). In the future, a gene co-expression network analysis could build on our results to offer additional insights into how sunflowers respond to drought stress [78]) through, for example, the identification of hub genes (i.e., genes with the most connectivity in a network) that underlie drought responses (e.g., cotton [79]).

### Differentially expressed genes in the F_1_ hybrid relative to its parents

In addition to evidence of gene expression complementation consistent with the dominance model for heterosis [9], we also observed transgressive expression patterns (over- and under-dominance) in the F_1_ hybrid relative to its parents in both control and drought conditions. Under control conditions, both under- and over-dominant DEGs were enriched in stimulus responses and metabolic processes. Drought responsive processes were up-regulated in the F_1_ under drought stress and metabolic processes were down-regulated. This is the same general pattern seen for all three genotypes under drought stress, implying that drought stress responses are strengthened in the hybrid as opposed to the development of a novel response. Similar patterns have been reported in other hybrids and have sometimes been interpreted as support for the over-dominance model of heterosis (reviewed in [80]). However, we argue that such patterns should be viewed as a correlate or downstream mediator of heterosis rather than its source, at least in the absence of stronger evidence of causation.

### Differential splicing negatively correlated with expression variation, reinforcing hybrid expression responses

Alternative splicing can contribute importantly to stress responses by influencing the quantity, developmental stage, and location of transcript and protein variant production [41], [42]. Although AS has been studied in multicellular organisms for decades [81], genome-wide differential splicing patterns have not been well-studied in the context of heterosis. Zhao et al. [82] identified DEGs that are related to heat stress tolerance in maize hybrids, and some of the DEGs had significantly higher levels of AS, suggesting up-regulating AS plays an important role in response to heat stress. Wang et al. [83] found that *cis*-regulated AS divergence may contribute to environmental stress response in hybrids of *Arabidopsis thaliana* ecotypes. More recently, a large-scale study of AS effects on heterosis was conducted in maize by Hu et al. [68]. They found numerous DAS events in the hybrid compared to its parents [68]. Those DAS were involved in regulating expression of genes associated with both carbon and nitrogen metabolism, thereby potentially contributing to the development of maize ear heterosis [68]. We found an inverse relationship between DAS and DEG in our study, suggesting that DAS could regulate/reinforce gene expression responses to drought in all three genotypes, as well as the transgressive expression patterns we observed in hybrids. However, keep in mind that some fraction of such changes may be a consequence of transcriptome shock and be unrelated to heterosis [22]. Future studies will employ long read sequencing data (i.e., Iso-Seq) and RT-PCR to confirm the AS events reported here, as well as to provide a more complete catalog of the AS events produced by abiotic stress and hybridization.

### Caveats and future directions

There are several caveats with the analyses and interpretations presented above. Most importantly, our study design confounds the effects of inbreeding depression and heterosis. The SAM population includes a mix of inbred and open-pollinated lines [52]. The latter were advanced via single-seed descent for one or two generations to reduce heterozygosity, but some residual heterozygosity remains. As a consequence, the positive effects of the “presence” allele likely comes both from minimizing inbreeding depression in homozygous genotypes and from masking deleterious “absence” alleles in heterozygous genotypes. As mentioned above, this residual heterozygosity also makes it more difficult to elucidate the effects of stop codons or other classes of deleterious mutations.

Another issue is that we may be over-estimating the importance of expression complementation. The majority of PAVs occur outside of expressed regions and thus are not subject to expression complementation. However, it has been shown that conserved non-coding sequences are frequently associated with gene expression levels and that their absence leads to gene expression loss [84]. Thus, even when outside of genic regions, PAVs may affect phenotypes through the loss or gain of expression.

Followup studies should increase the sequencing depth for the SAM population so that all classes of putatively deleterious mutations can be detected and analyzed. In addition, we recommend that crosses be made between SAM inbred lines and multiple tester lines, thereby permitting direct assessment of gene expression complementation and heterosis in hundreds of F_1_ combinations as opposed to the single F_1_ analyzed here.

## Conclusions

We studied heterosis and drought responses in cultivated sunflower using a combination of GWA and transcriptomic (expression and alternative splicing) analyses under control and drought conditions. We showed that “absence” alleles at PAVs were disproportionately associated with reduced values of heterosis-related traits, but not other kinds of traits. Furthermore, we identified gene PAVs differentiating the parental lines that were complemented in their F_1_ hybrid, consistent with the dominance model of heterosis. Many of the PAVs were expressed consistently in both control and drought conditions, possibly accounting for the maintenance of heterosis under drought.

We also identified transgressively expressed and differentially spliced genes in the F_1_ hybrid compared with its parents. All three genotypes responded similarly to drought stress by up-regulating stress response genes and down-regulating metabolic processes. However, these responses were further strengthened in the F_1_ hybrid. Alternative splicing changes were negatively correlated with expression changes, implying that AS acts to reinforce expression differences.

Our results offer a straightforward mechanism for heterosis in sunflower and its maintenance under drought stress. Under this mechanism, heterosis should be greatest in hybrids that complement the highest proportion of PAVs. This could be determined bioinformatically, permitting breeders to choose parental lines that are likely to maximize heterosis. More generally, our approach, which combines GWA of PAVs and expression analyses, could be fruitfully applied to other taxa to establish whether expression complementation is a widespread cause of heterosis.

## Declaration of Competing Interest

The authors declare that they have no known competing financial interests or personal relationships that could have appeared to influence the work reported in this paper.
