# Supplementary material for: Expression complementation of gene presence/absence polymorphisms in hybrids contributes importantly to heterosis in sunflower
Source: J Adv Res. 2022 Apr 22;42:83–98. doi: 10.1016/j.jare.2022.04.008 (PMC9788961; doi:10.1016/j.jare.2022.04.008)
Supplement: Supplementary data 1 [file mmc1.docx]

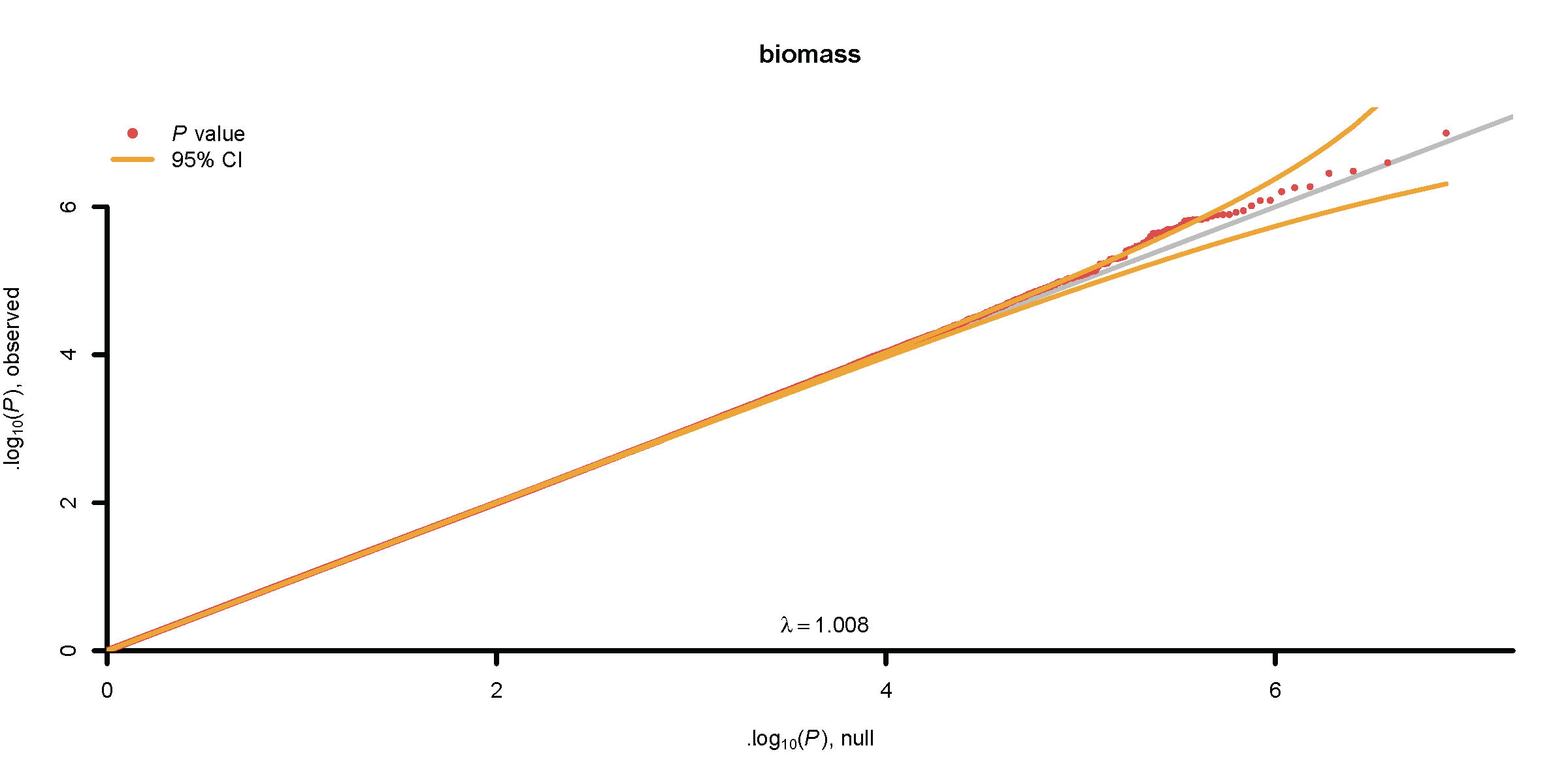

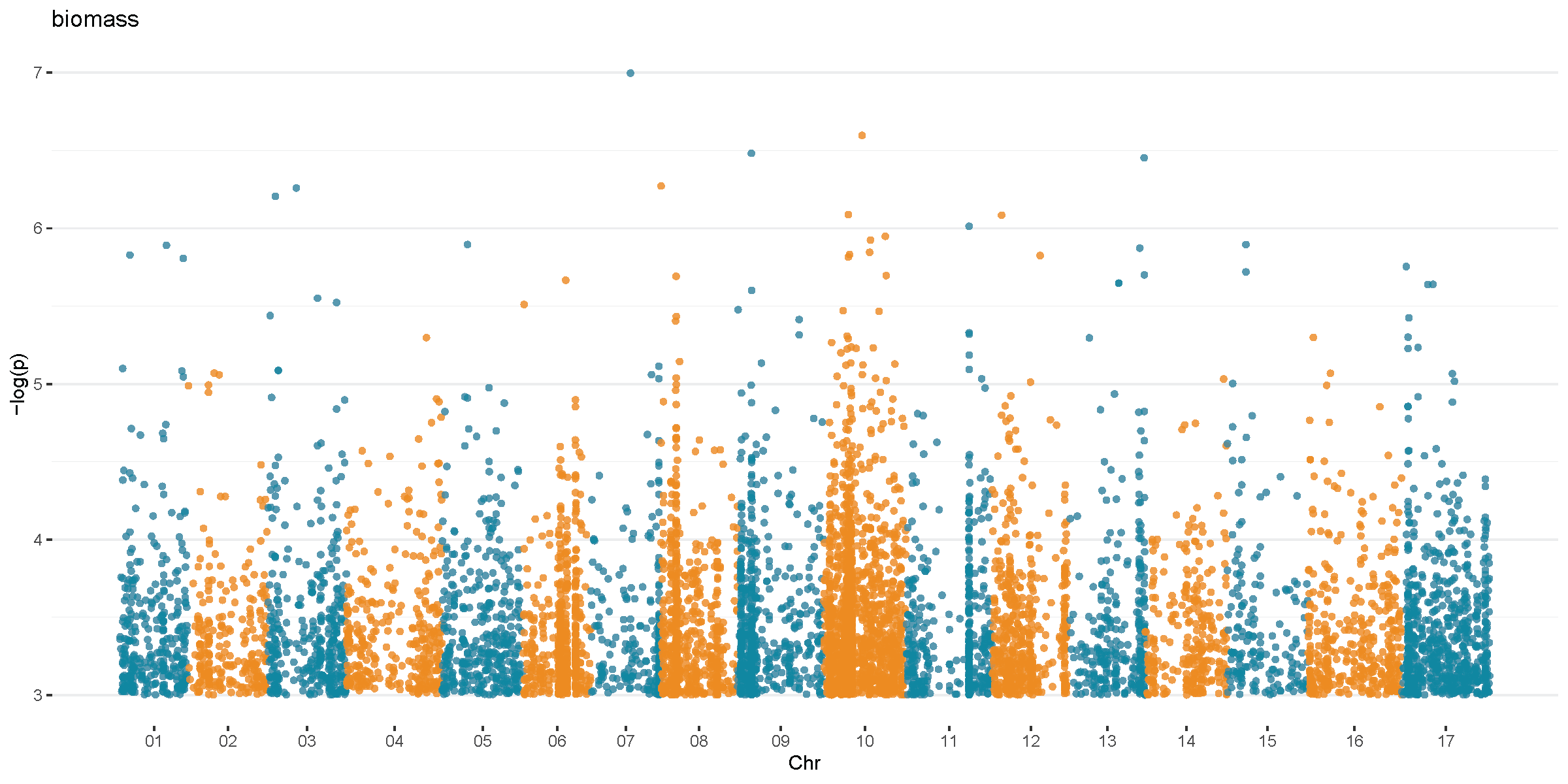

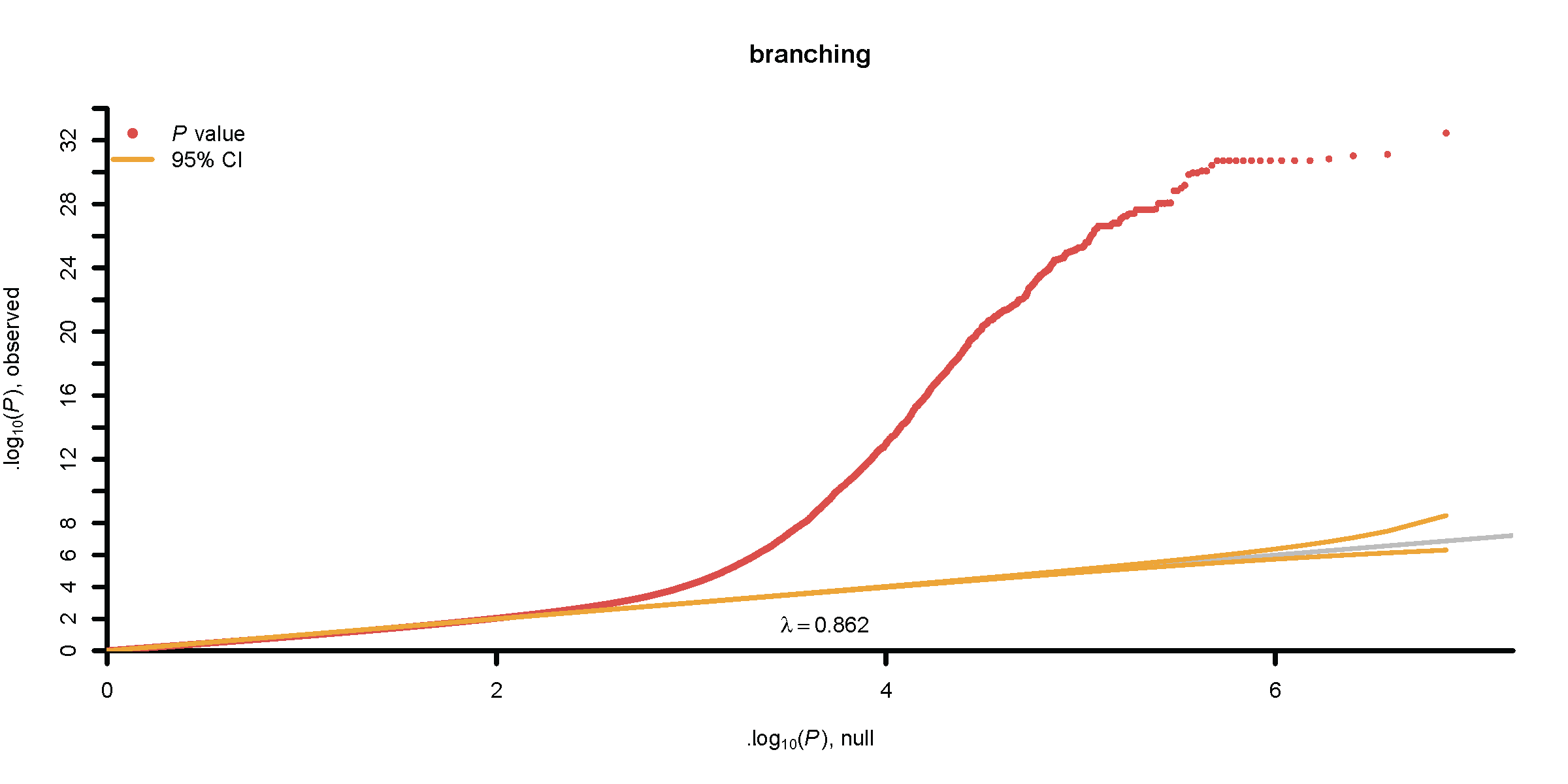

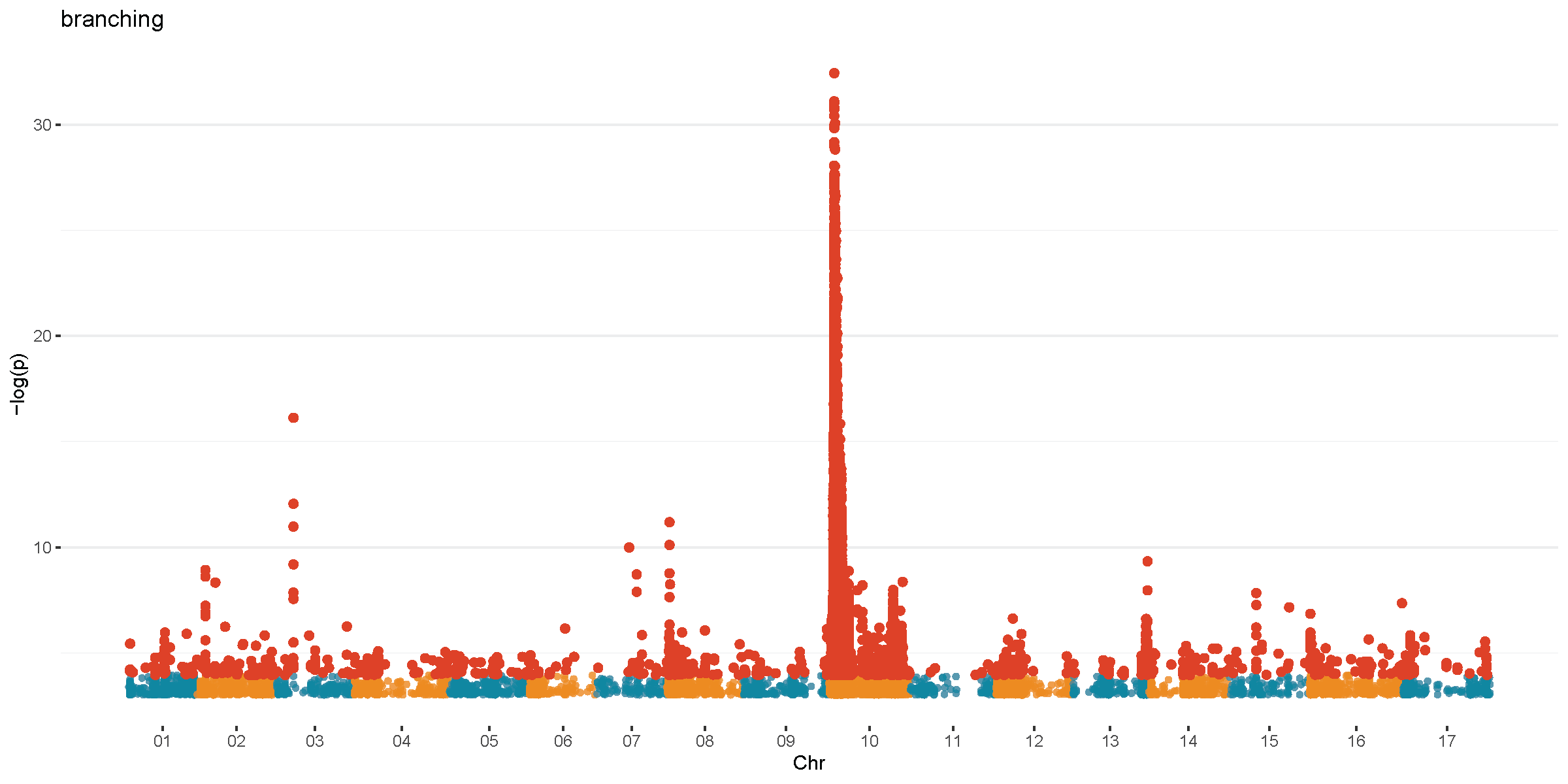

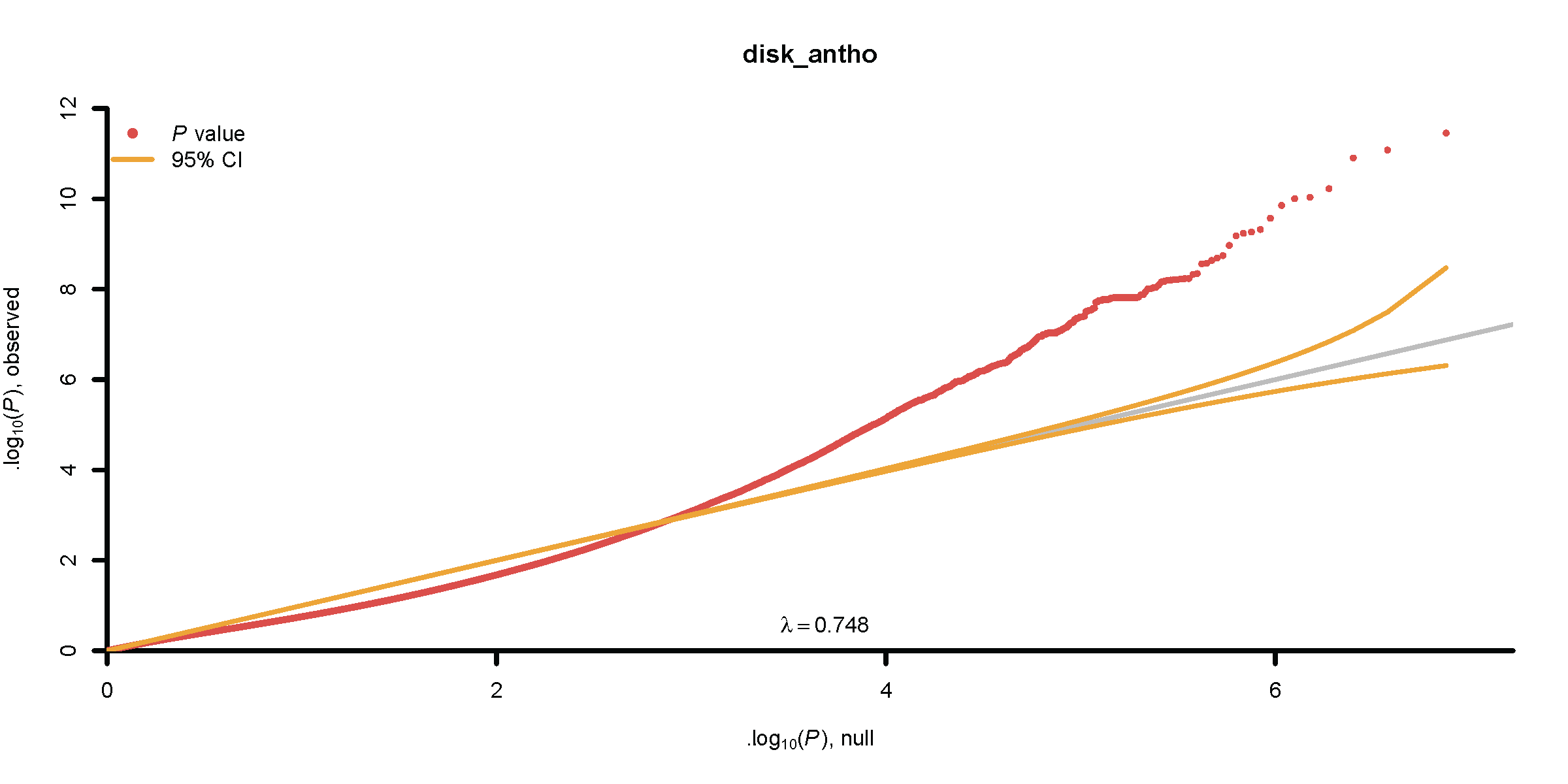

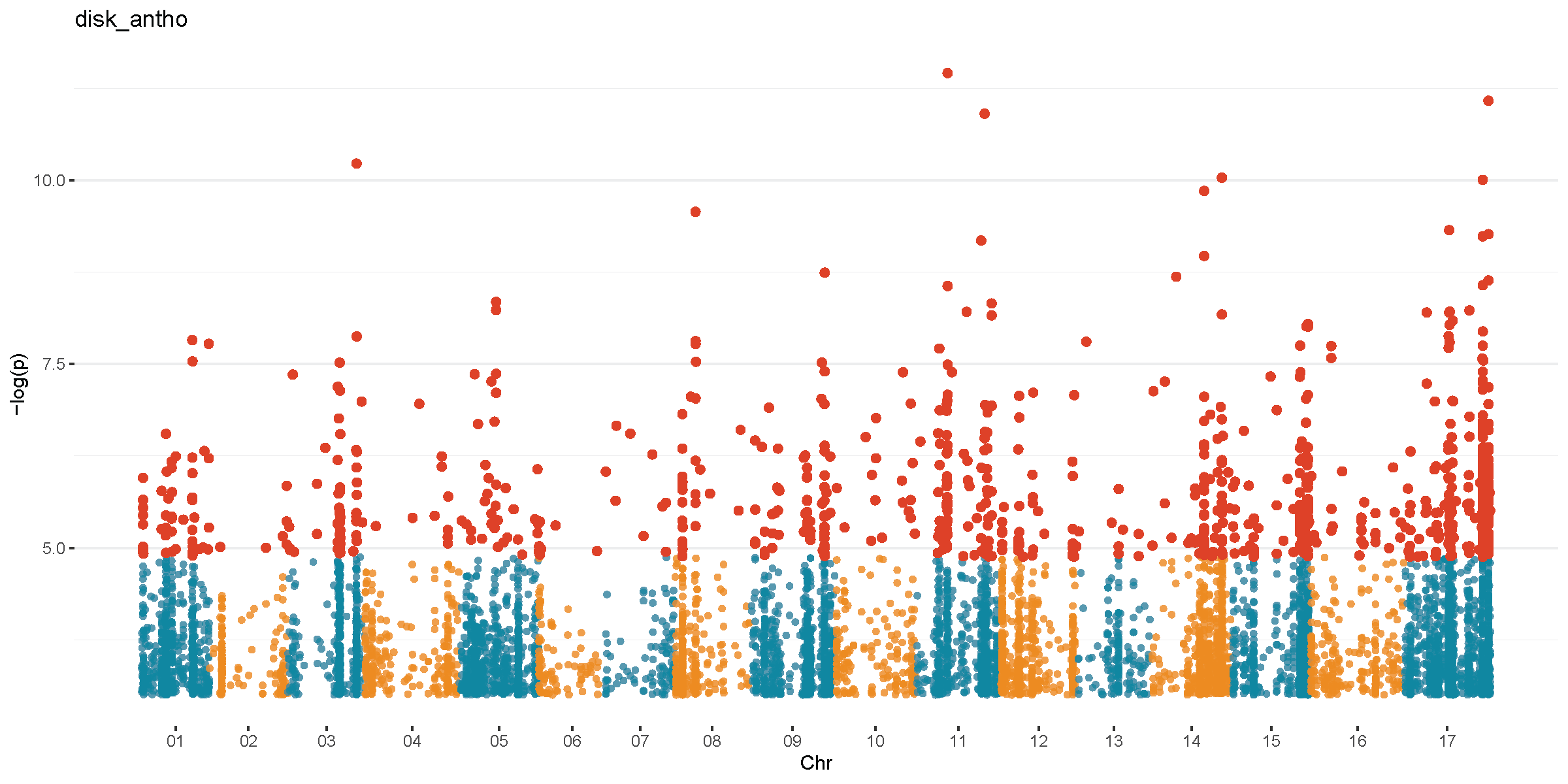

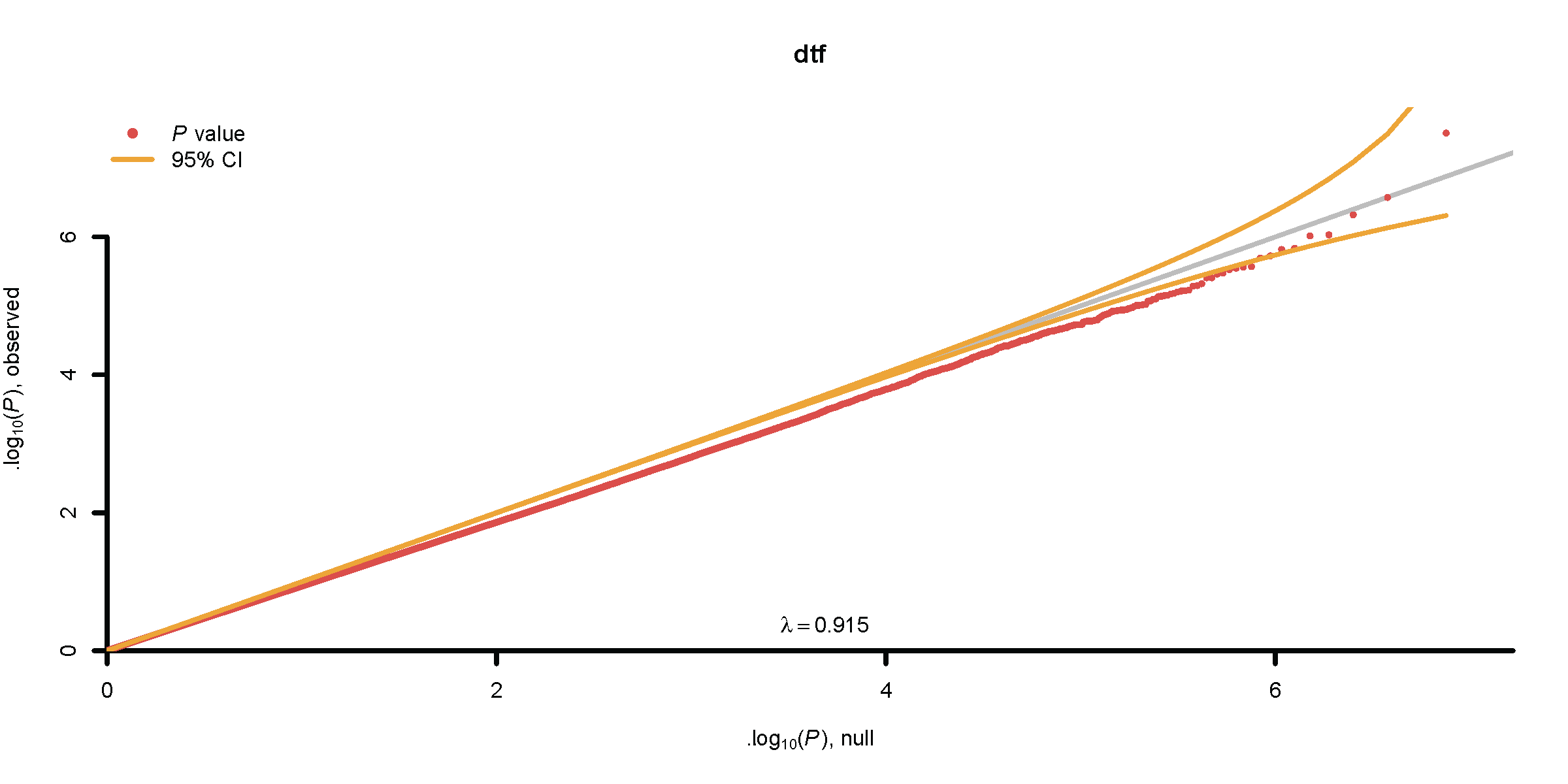

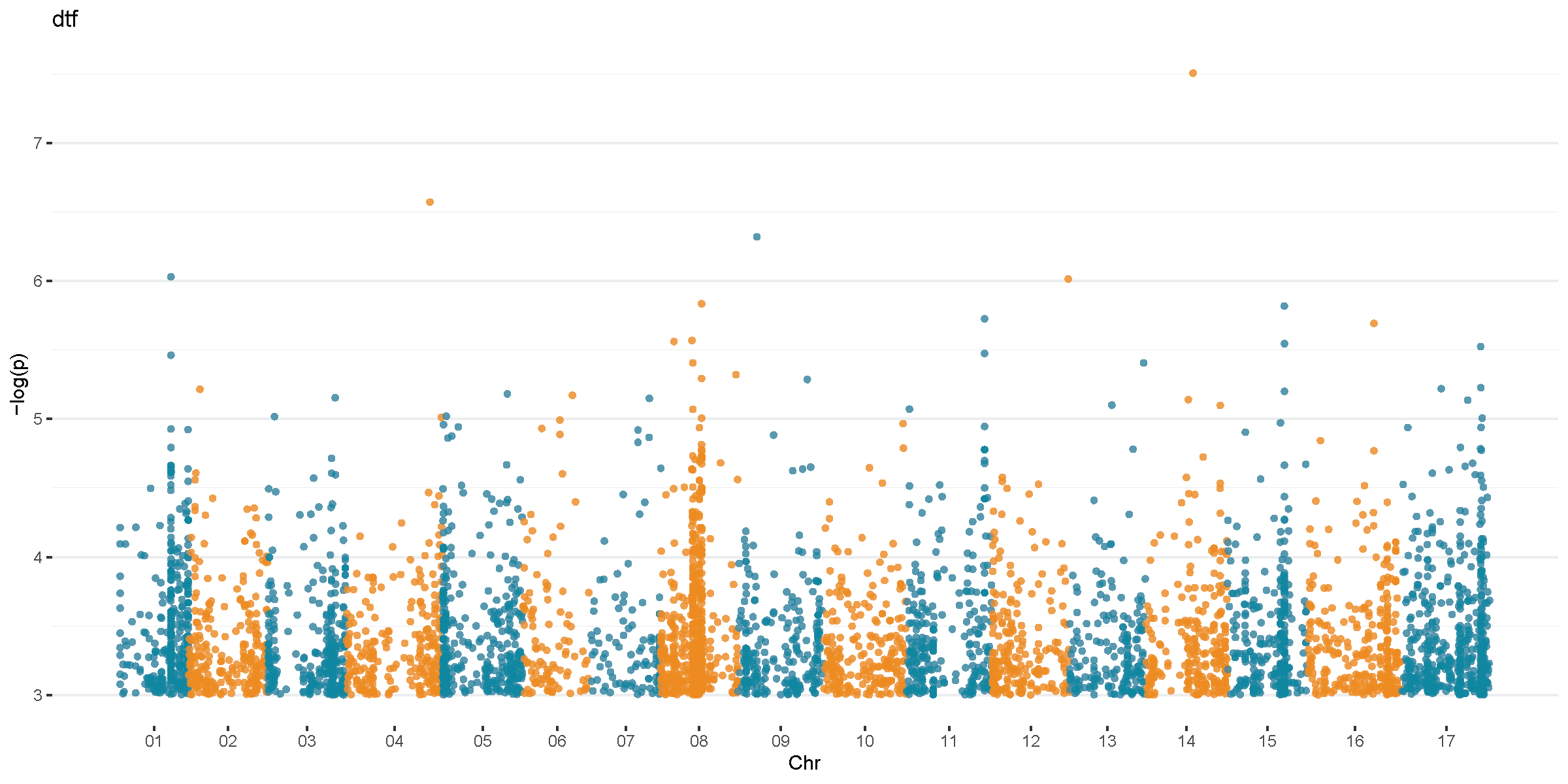

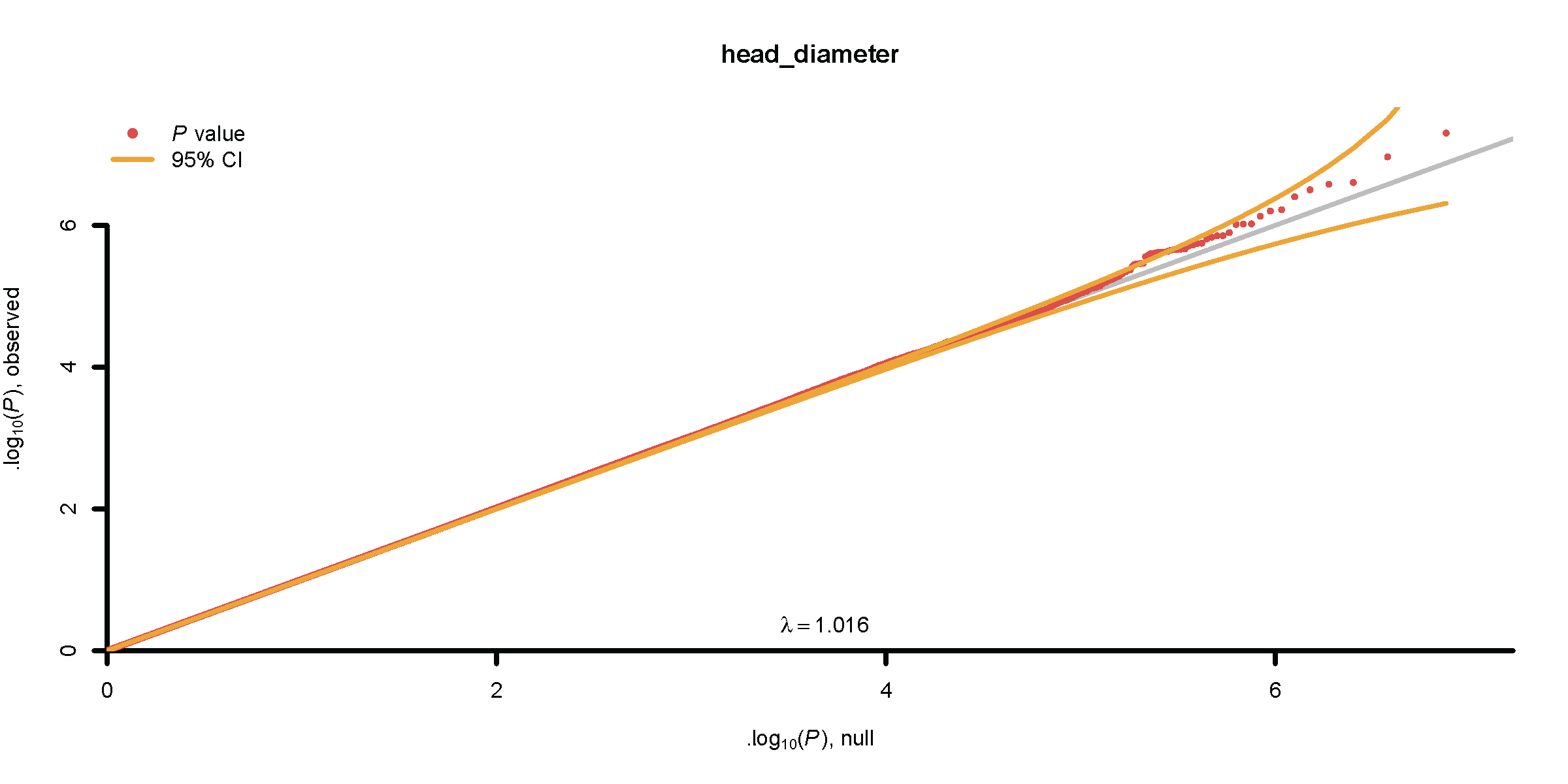

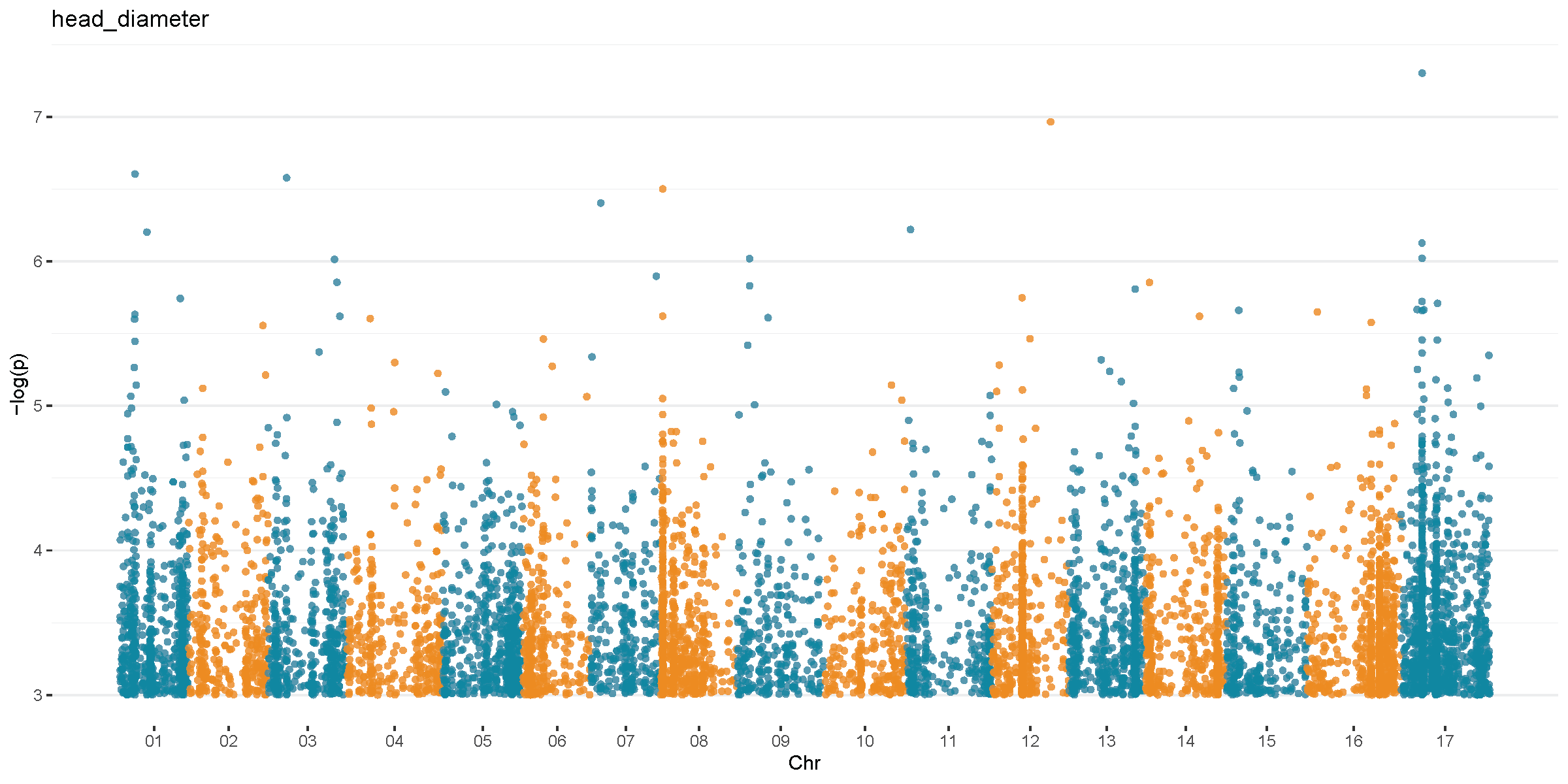

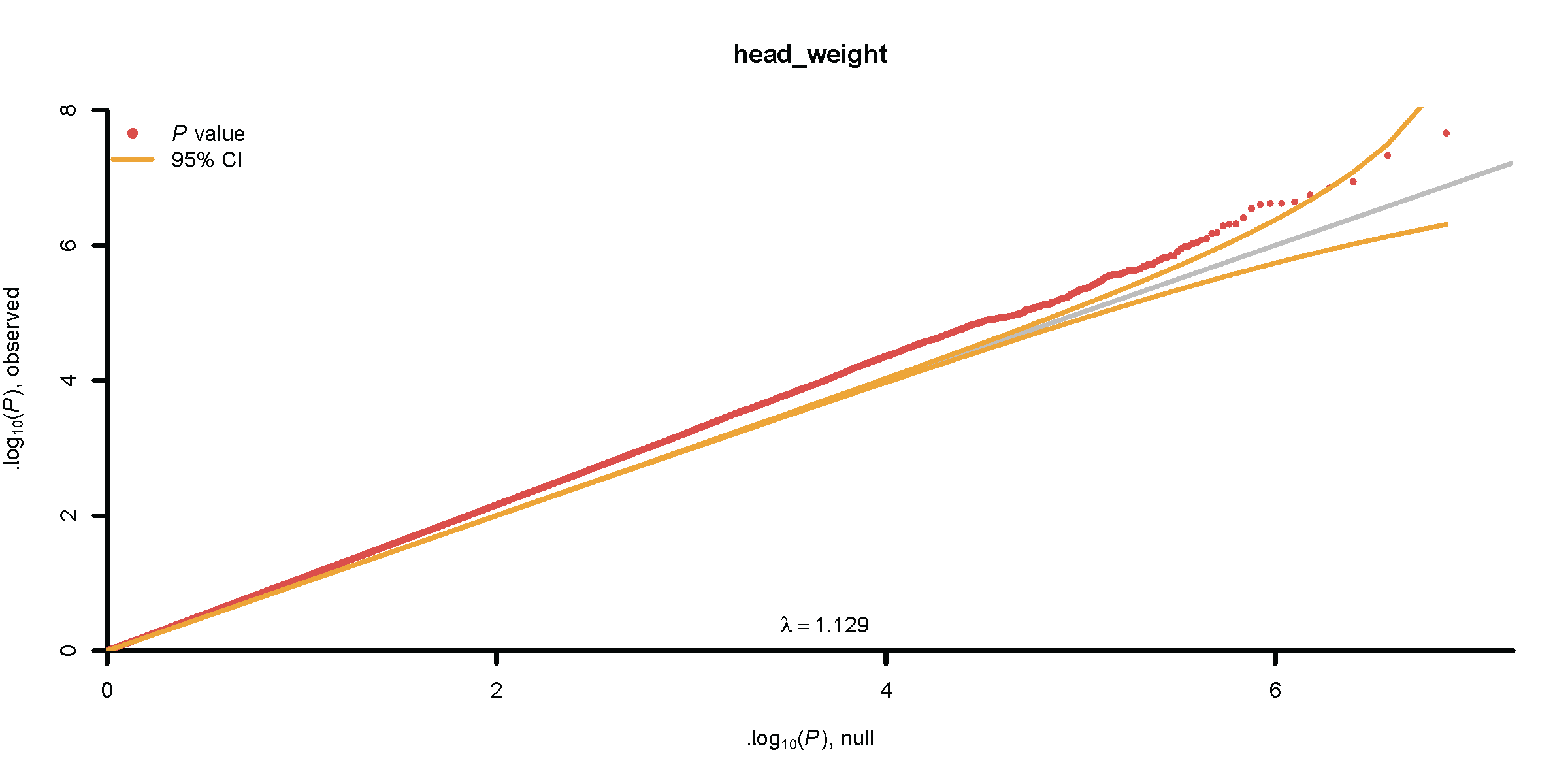

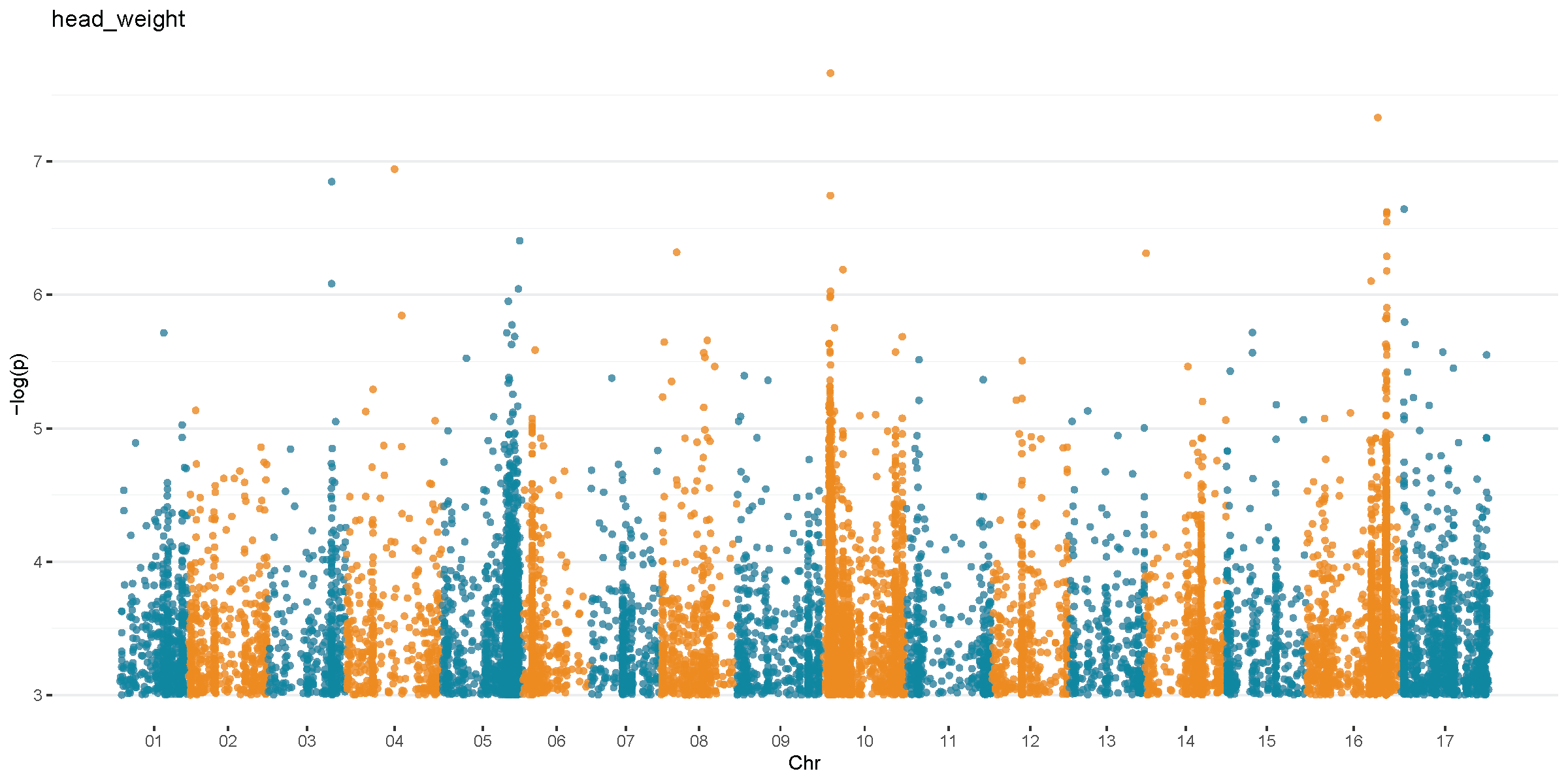

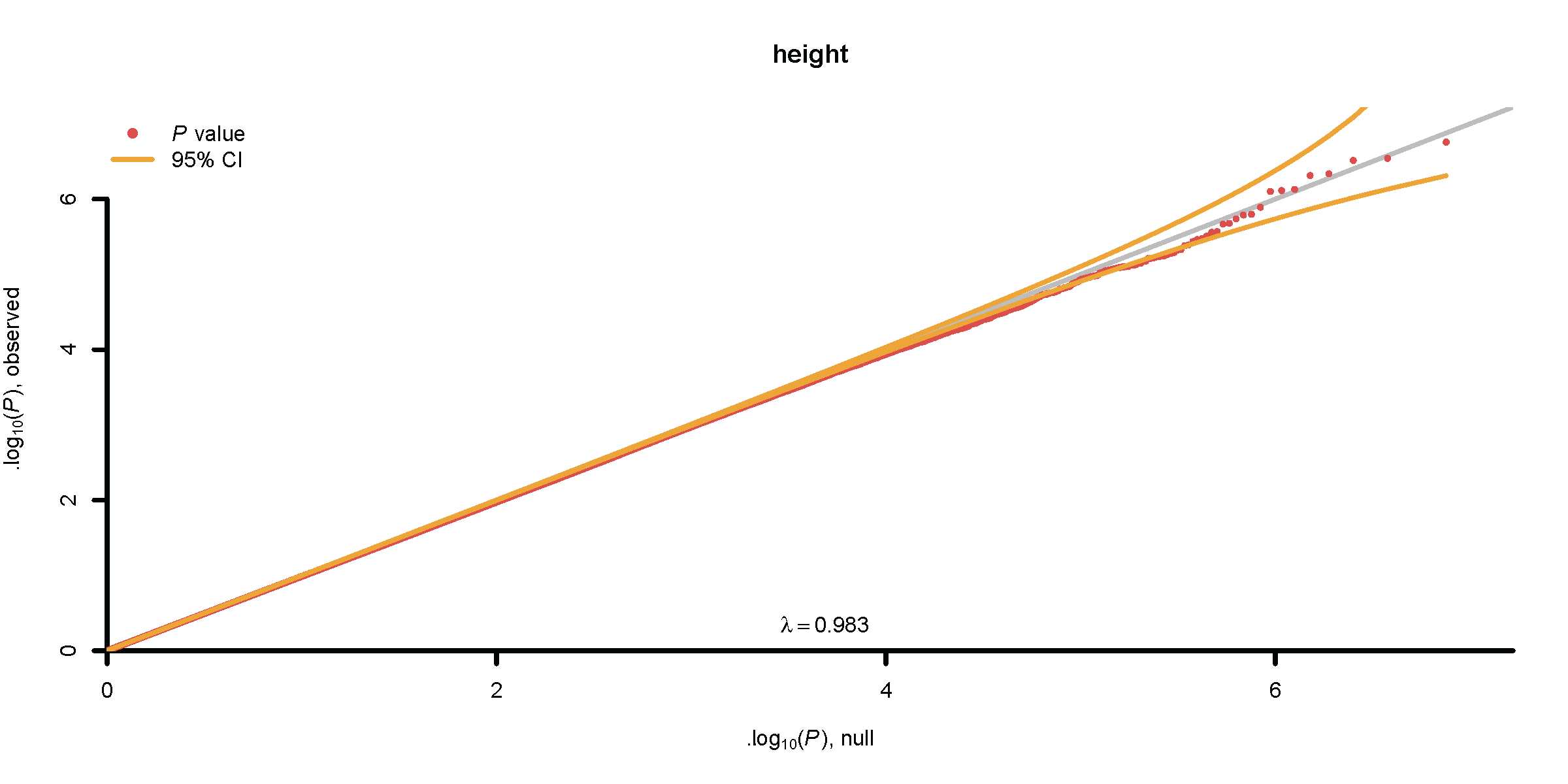

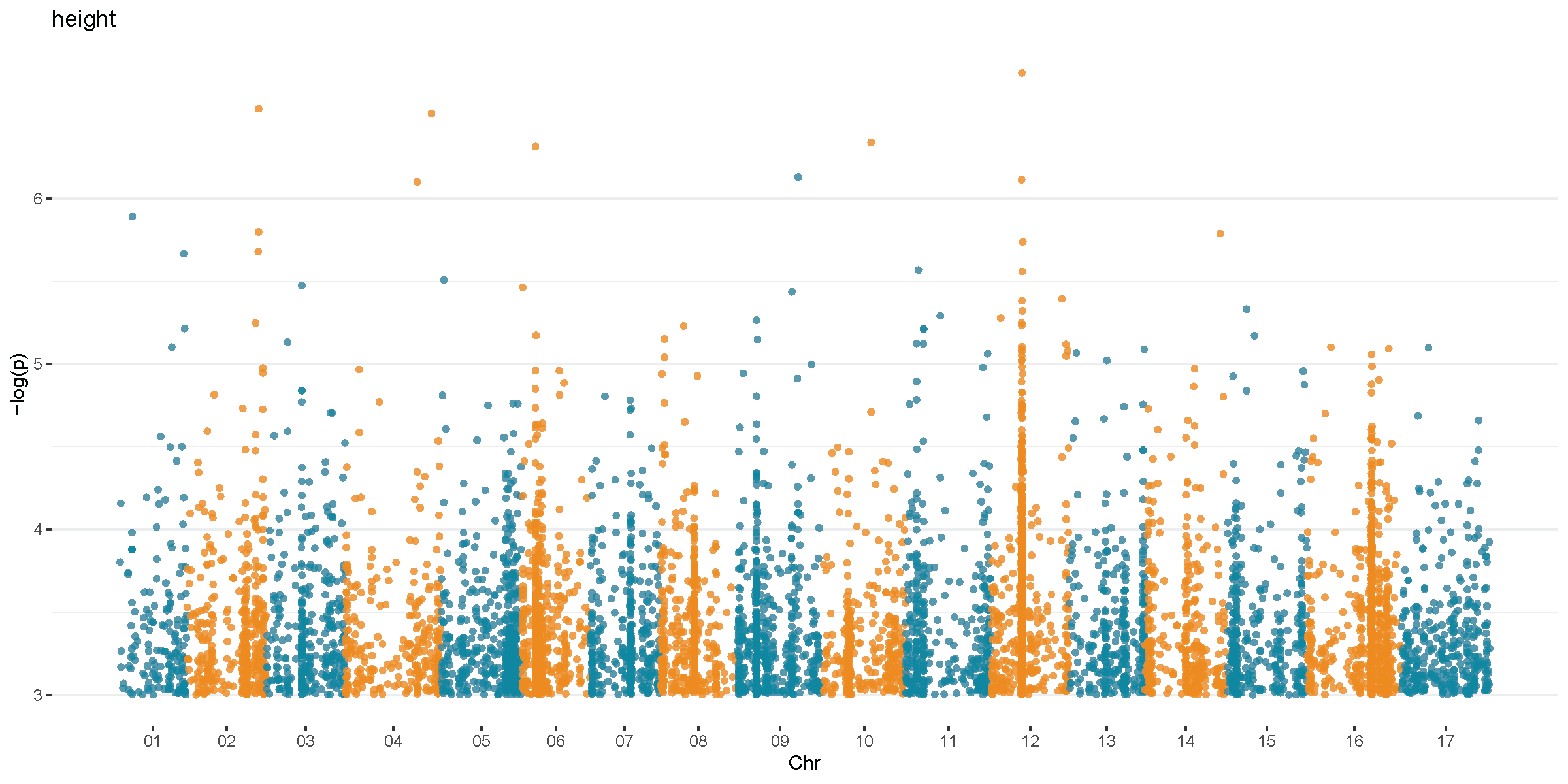

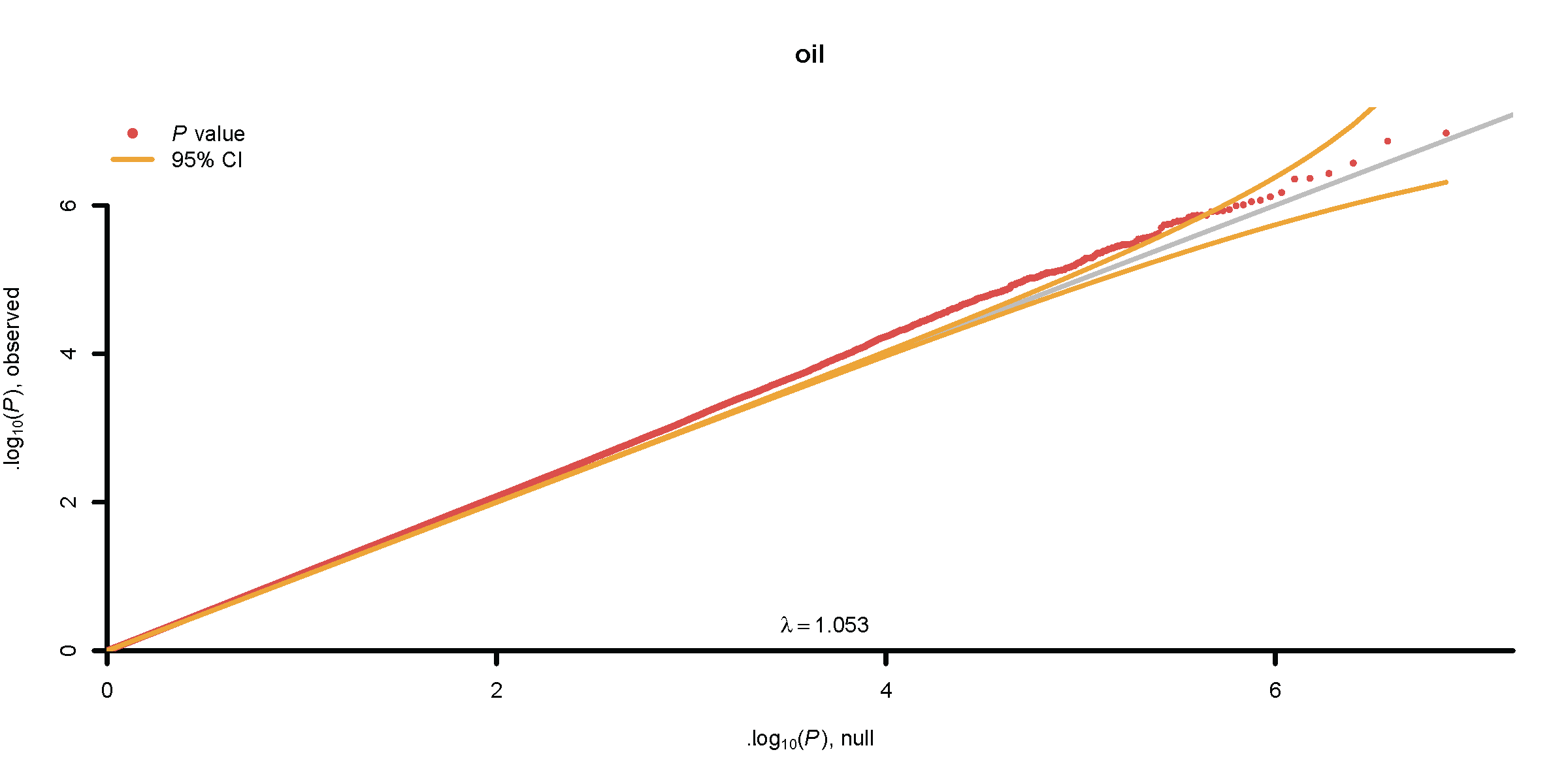

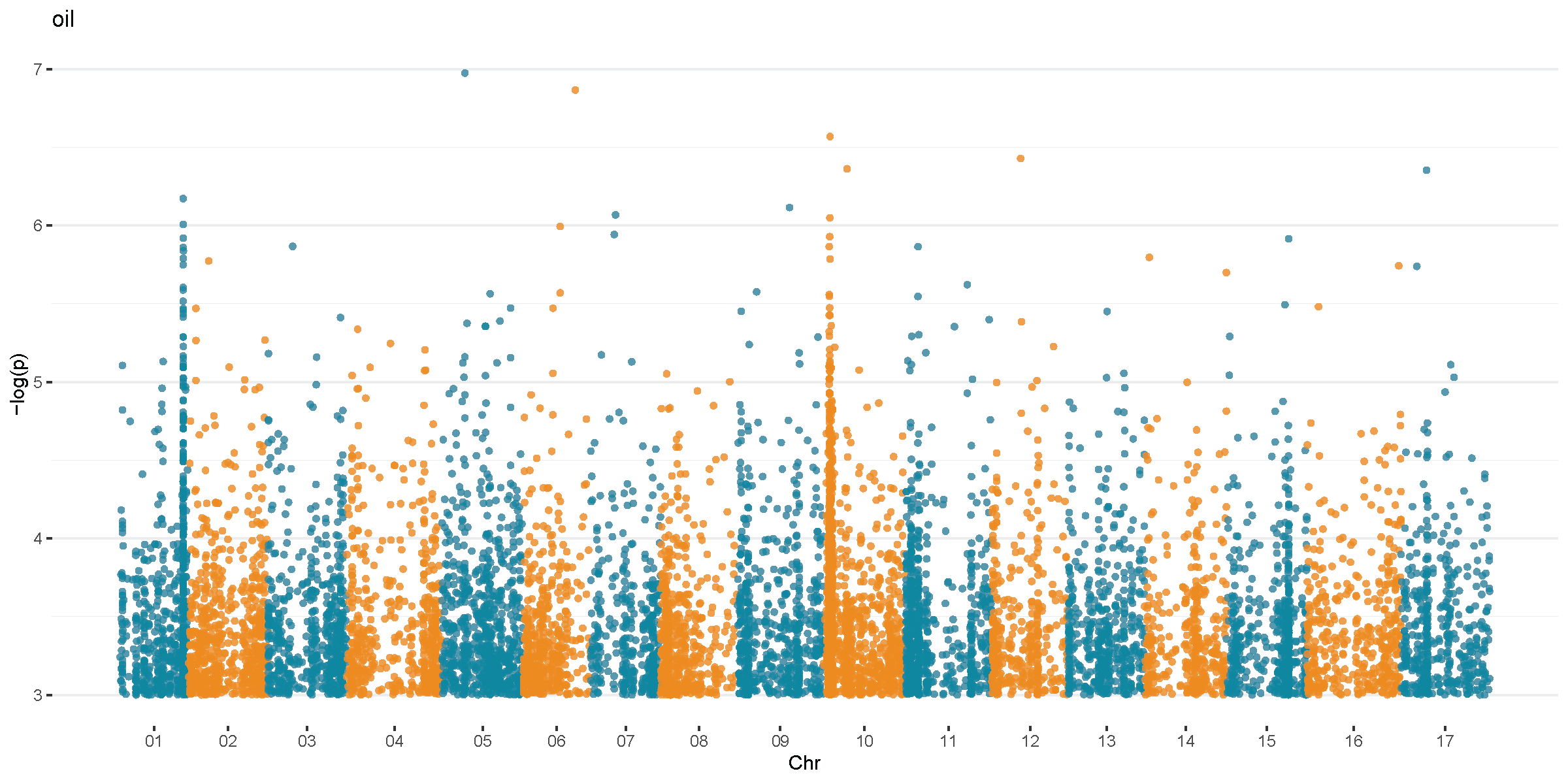


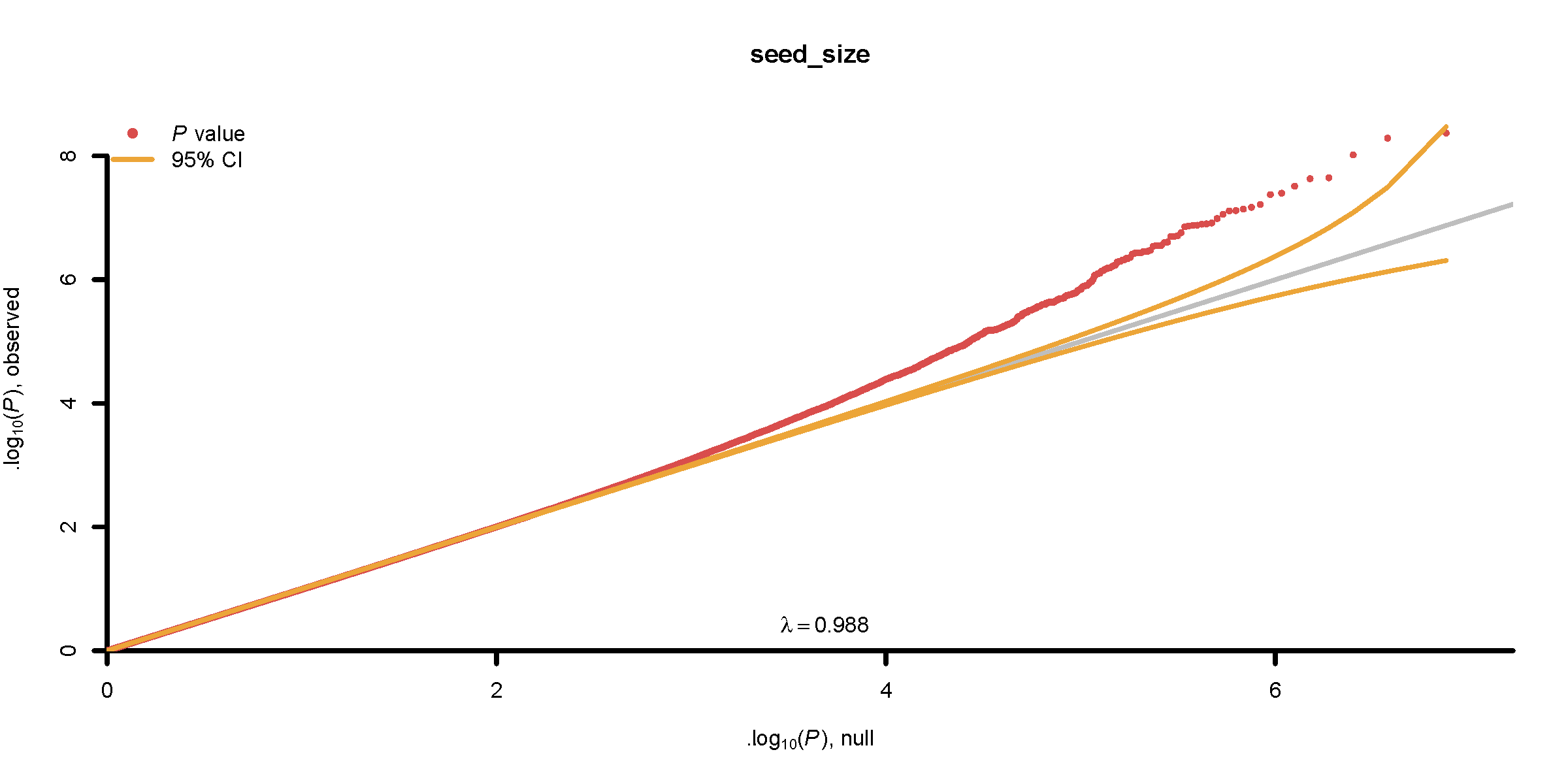


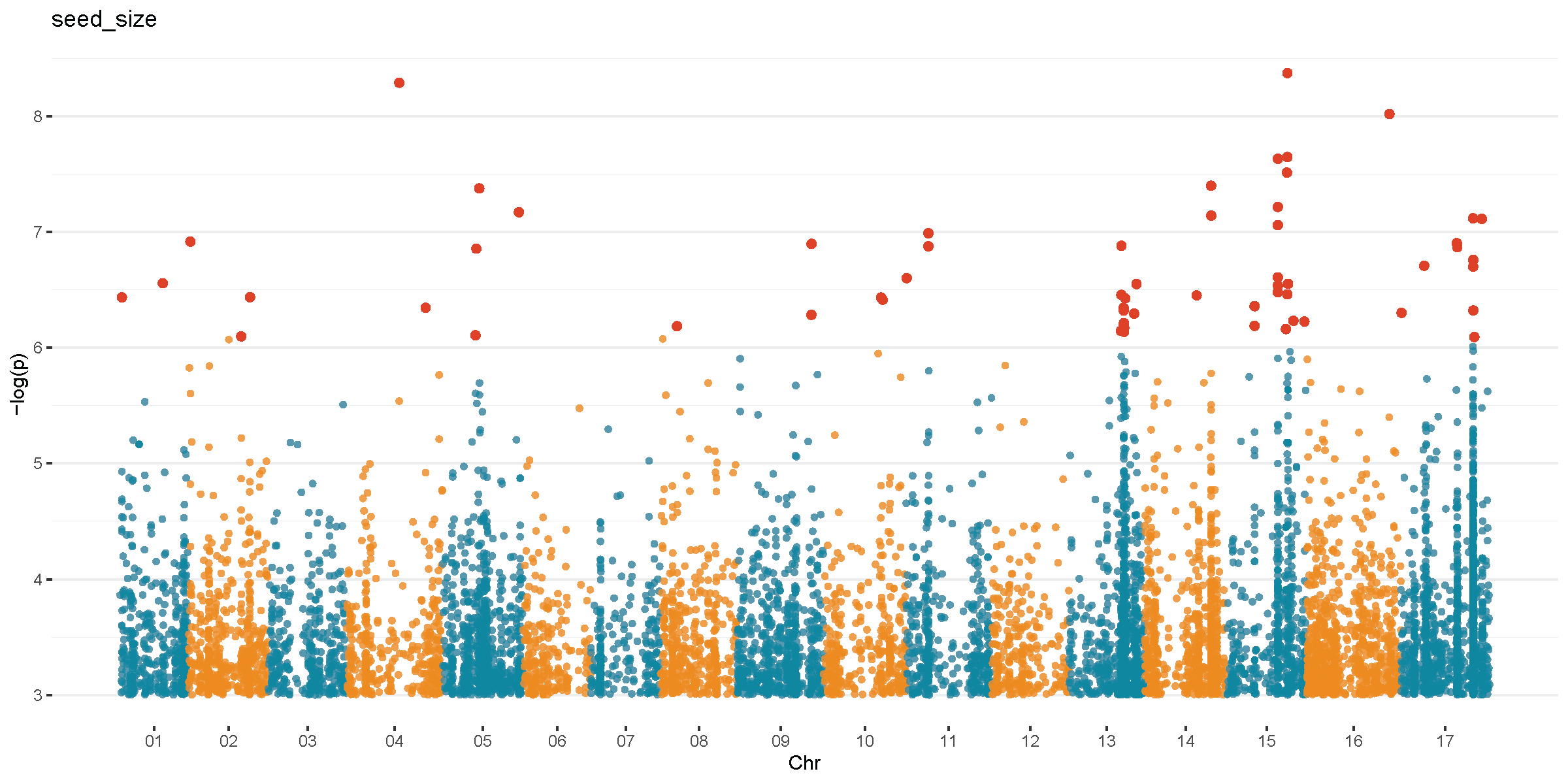


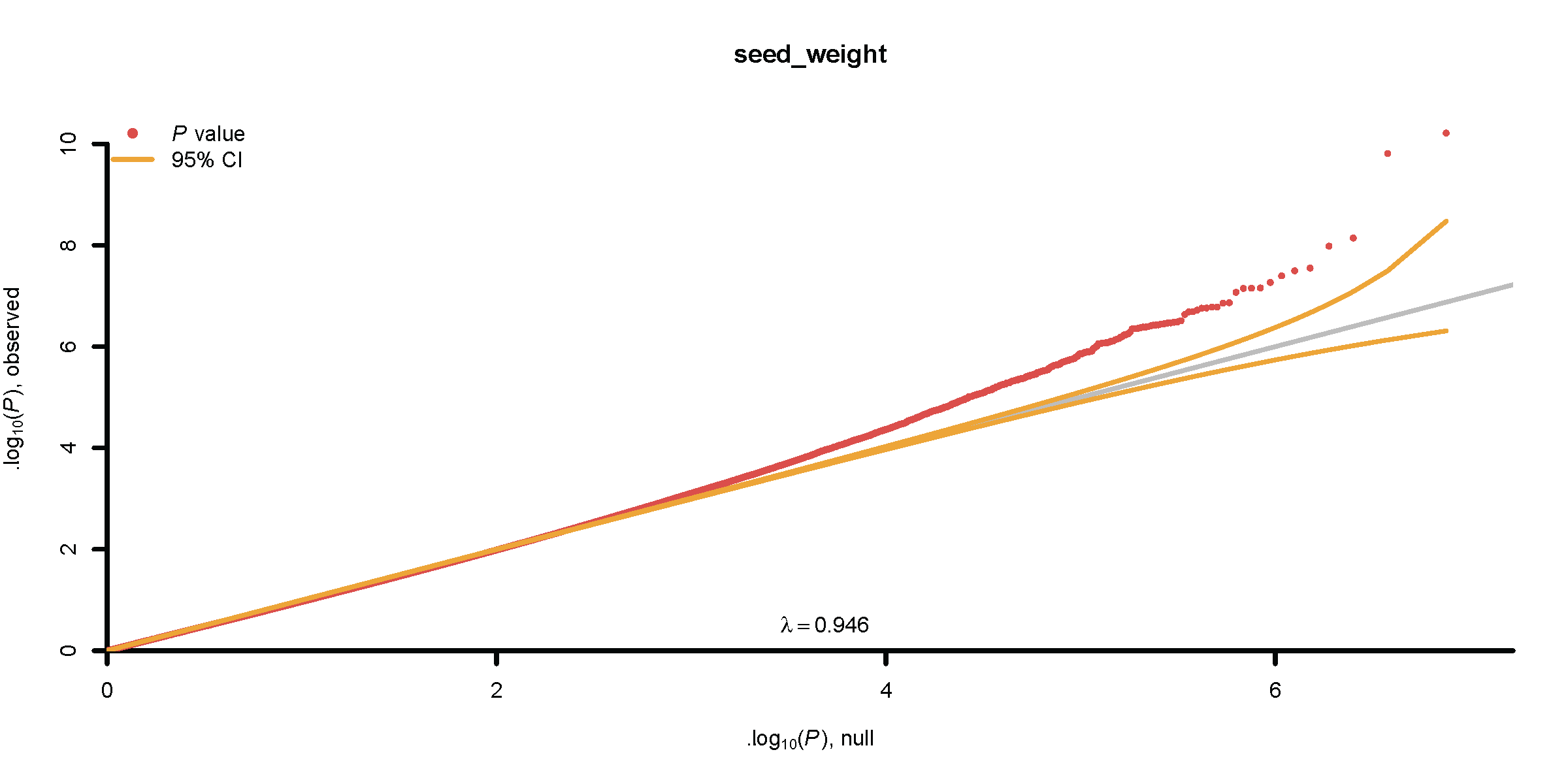

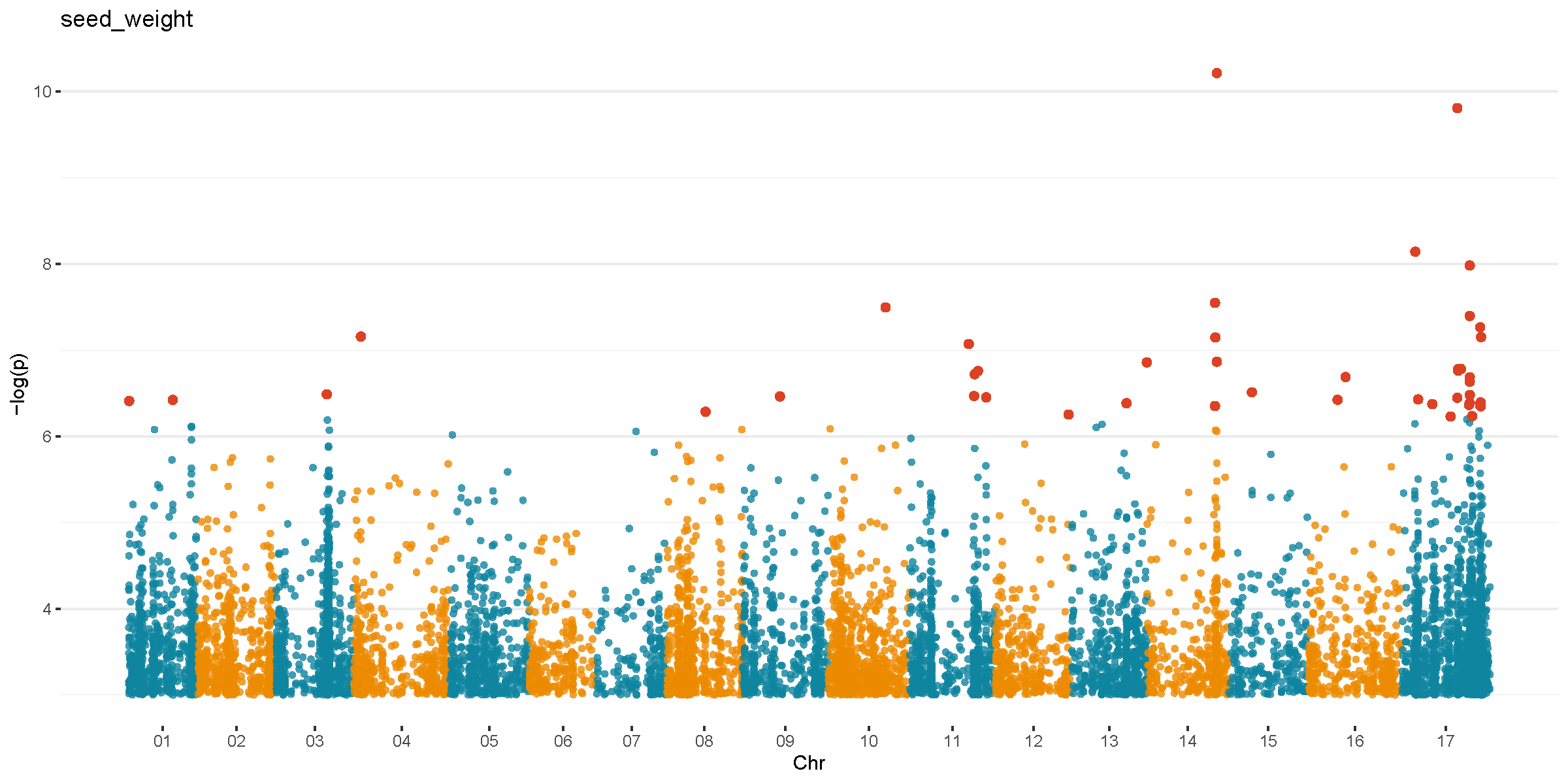

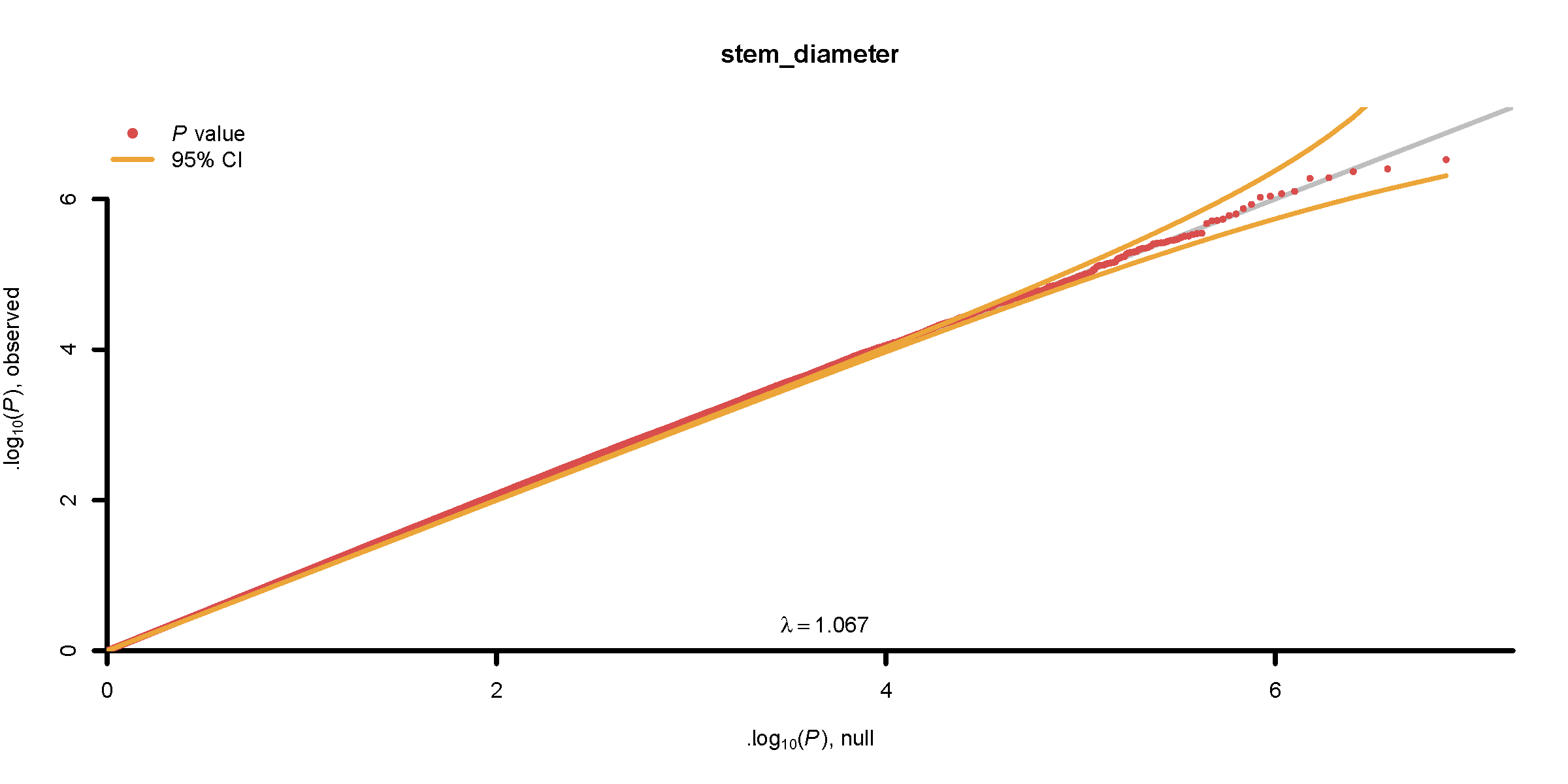

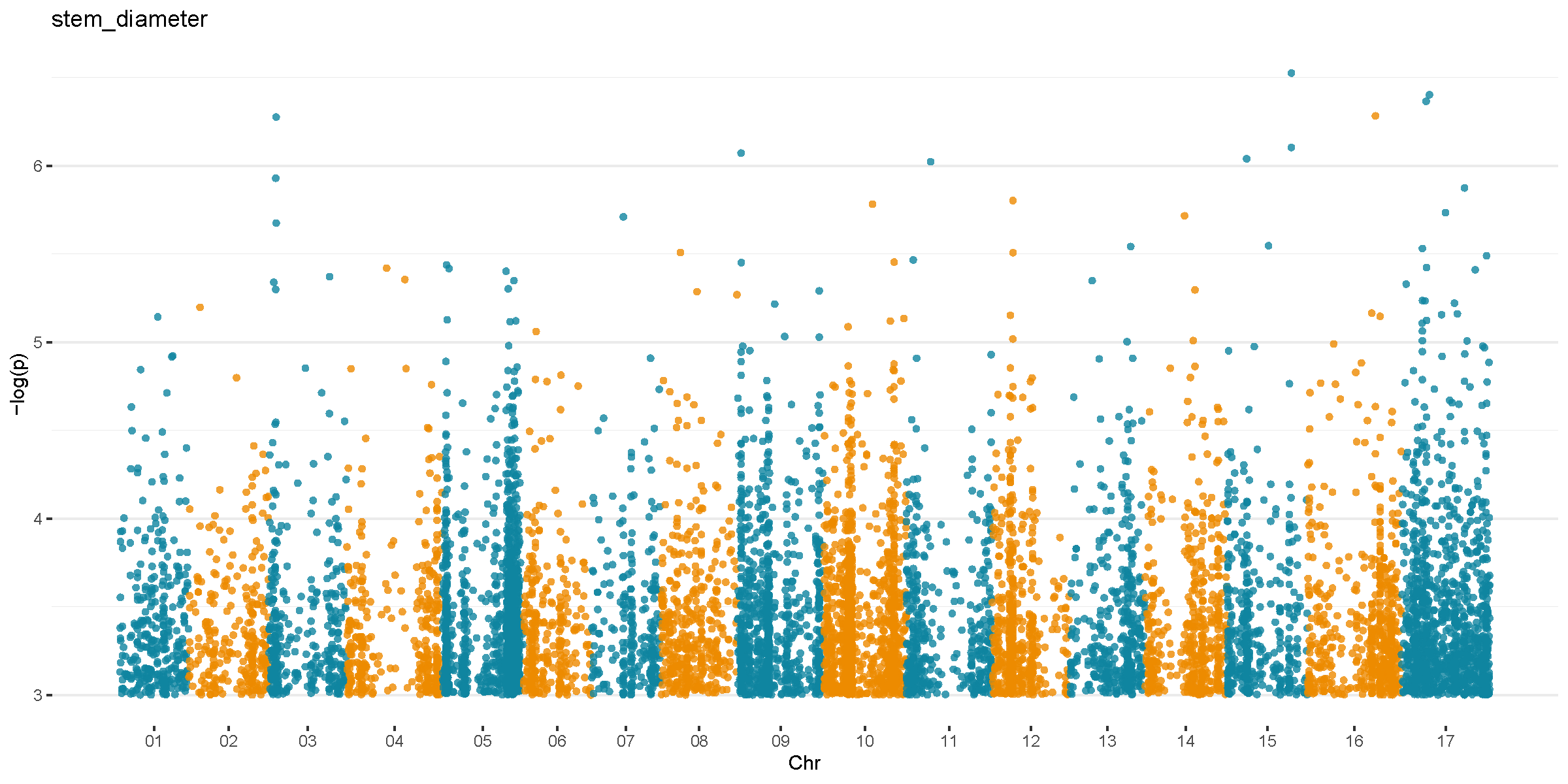

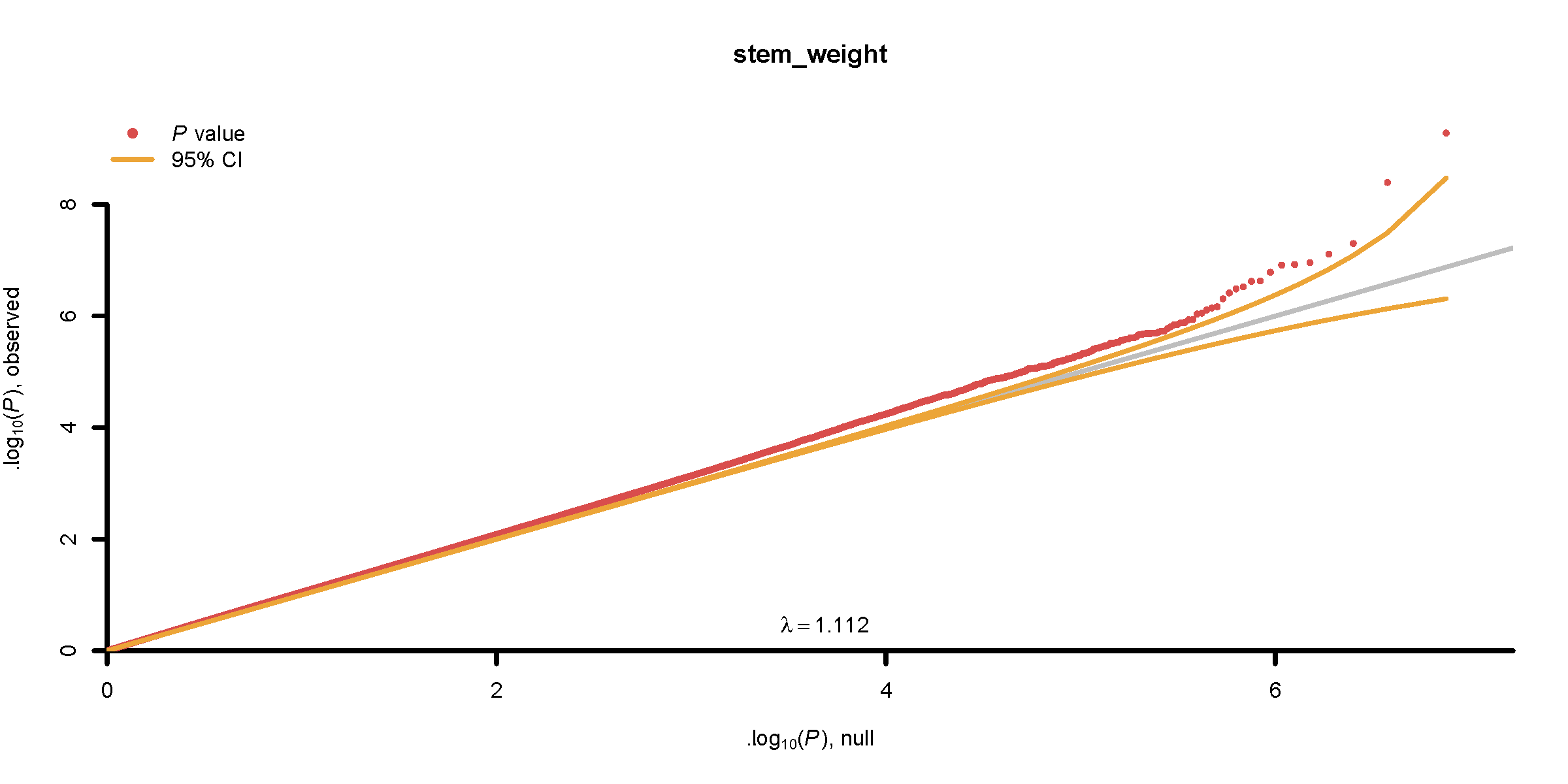

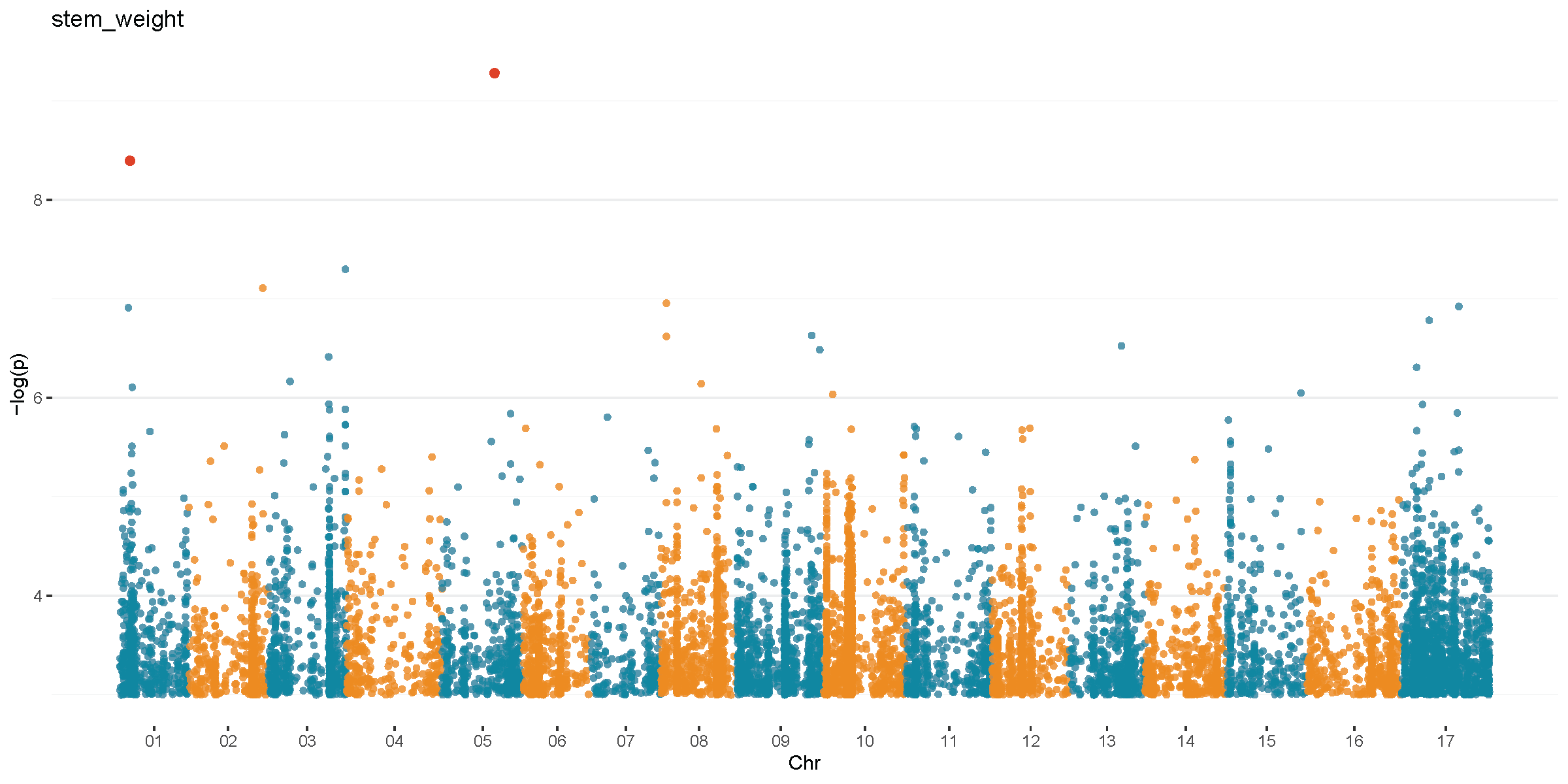

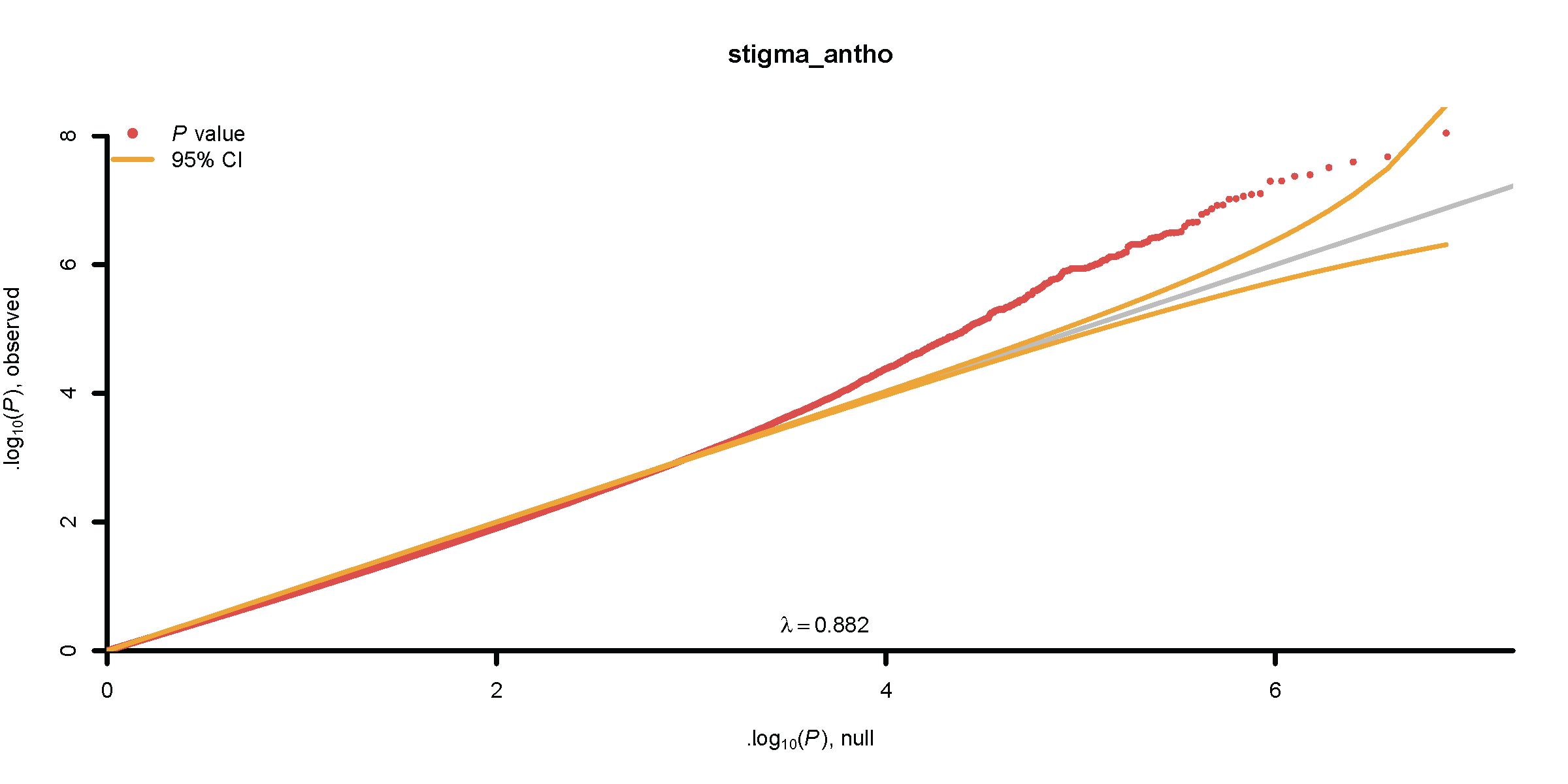

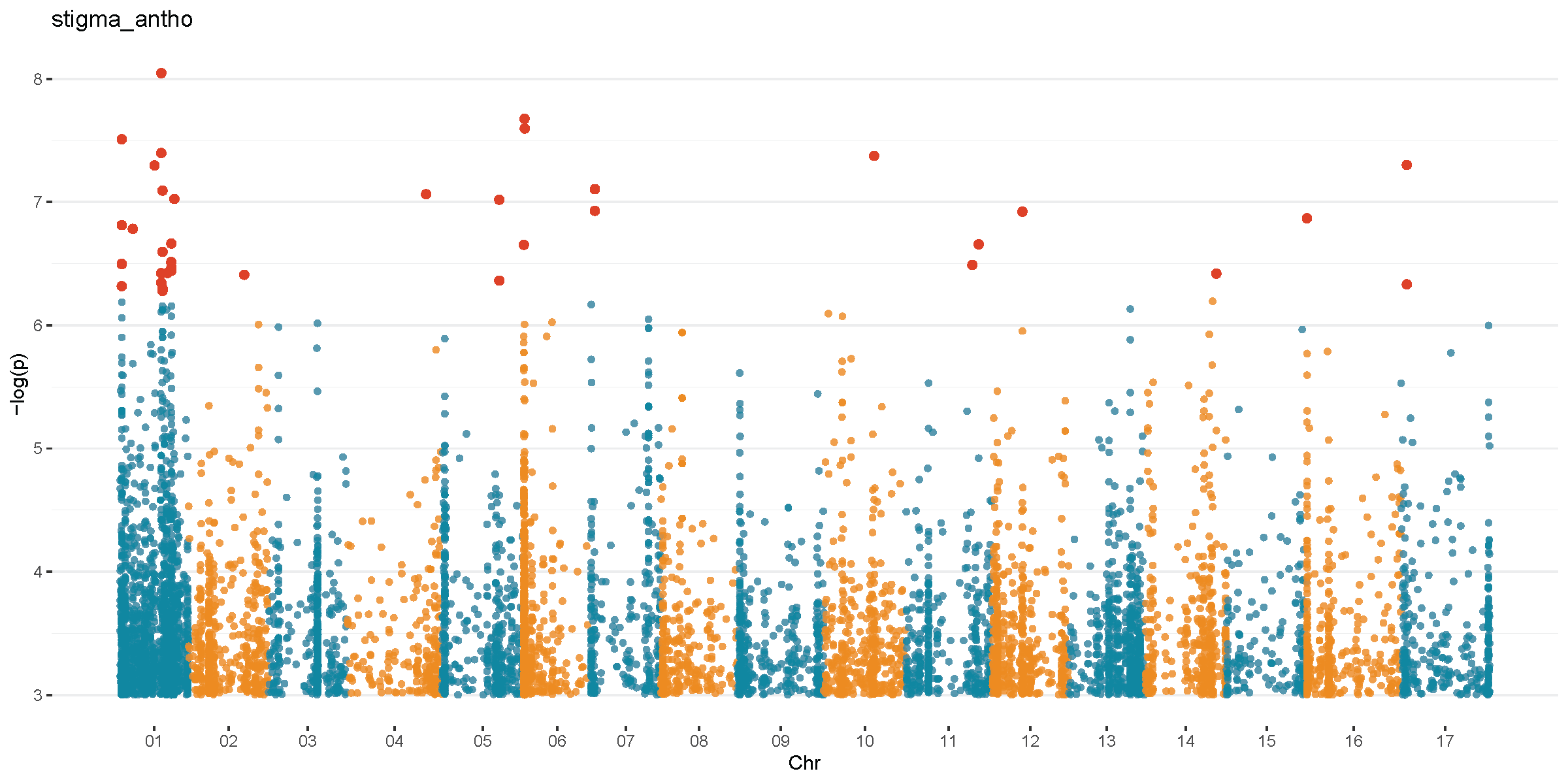


Supplementary Figure S1. Genome-wide association analyses with presence/absence variation. For each trait, panel A represents a quantile-quantile plot of p-values for tested PAV regions using qqPlotFast in ramwas. Values above the diagonal line indicate higher significance than expected based on a normal distribution. Panel B is a manhattan plot of -log10(p-values) for PAV regions. Significance was determined using a q-value correction with a false discovery rate of 0.1. Significant regions are highlighted in red. using a q-value correction with a FDR of 10%. Data collected at two field sites in Vancouver, Canada.


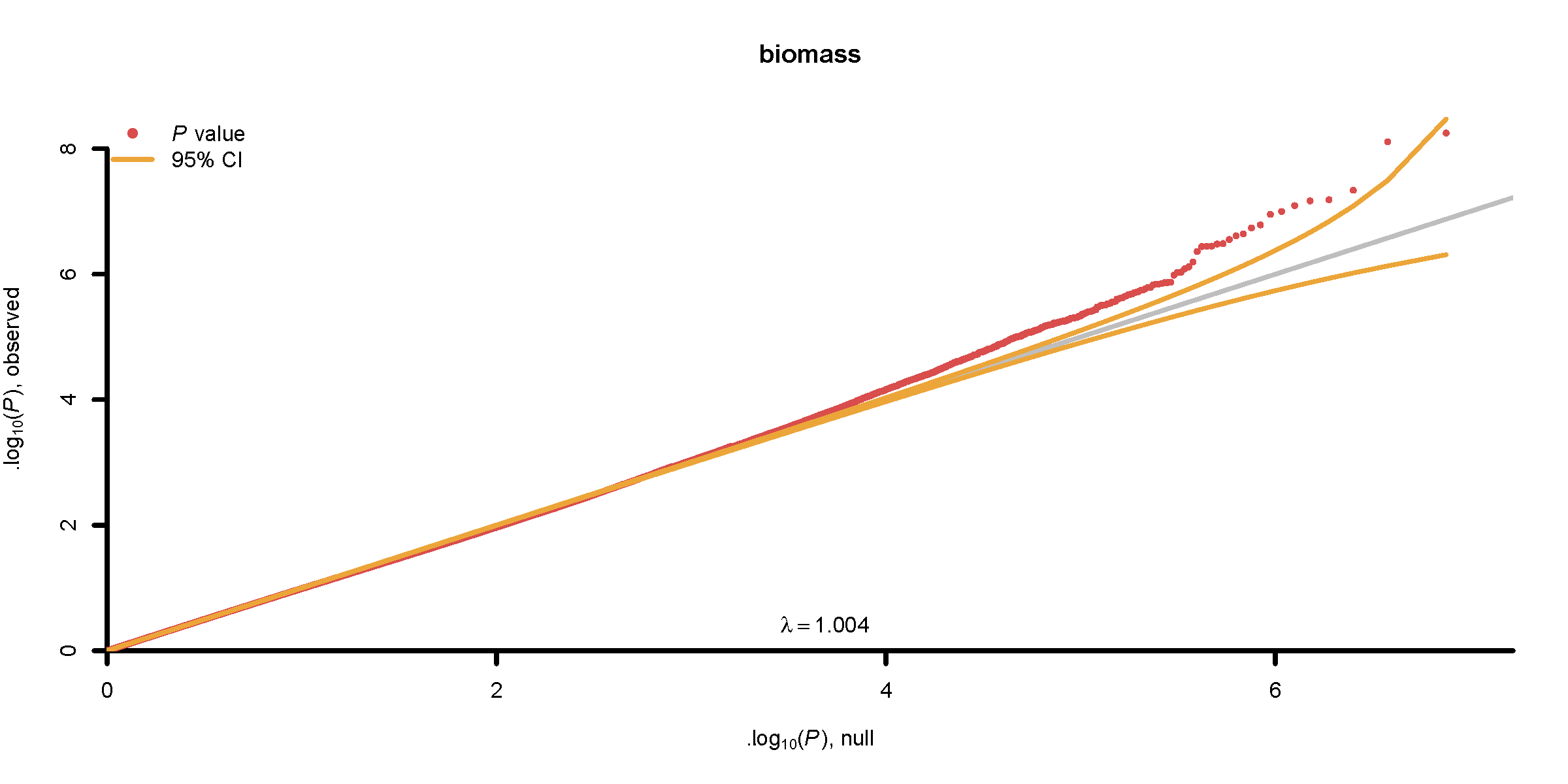

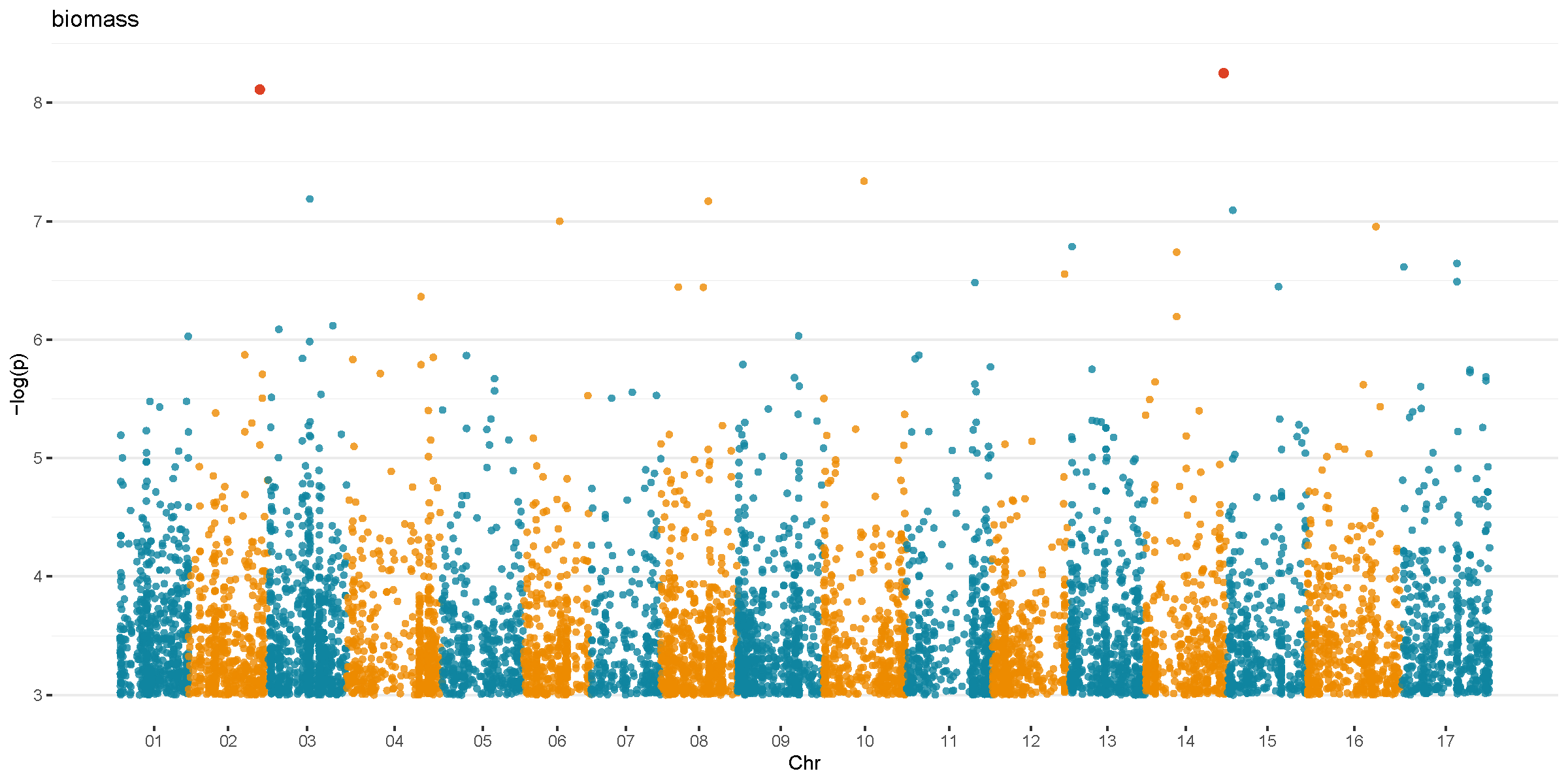

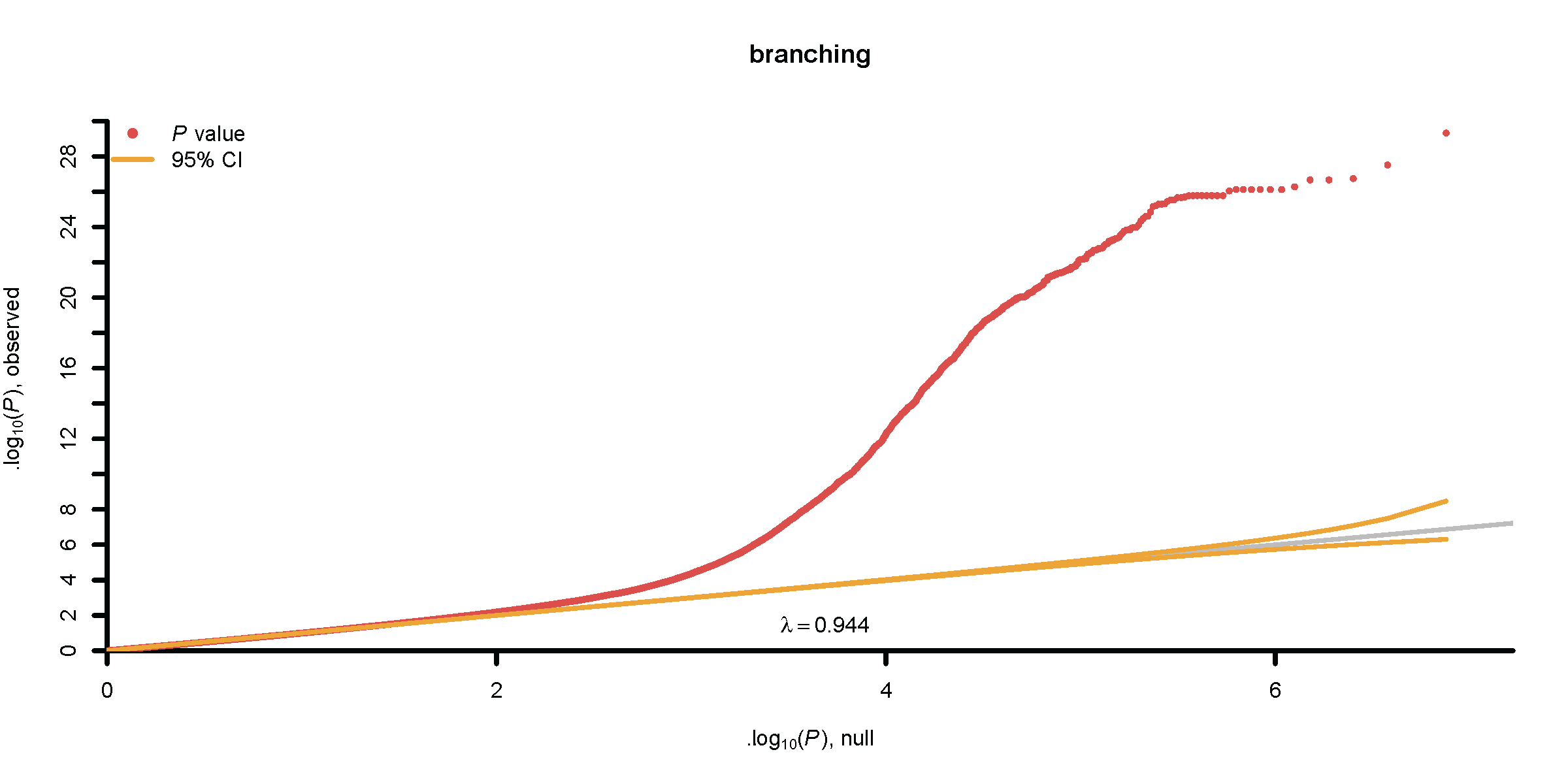

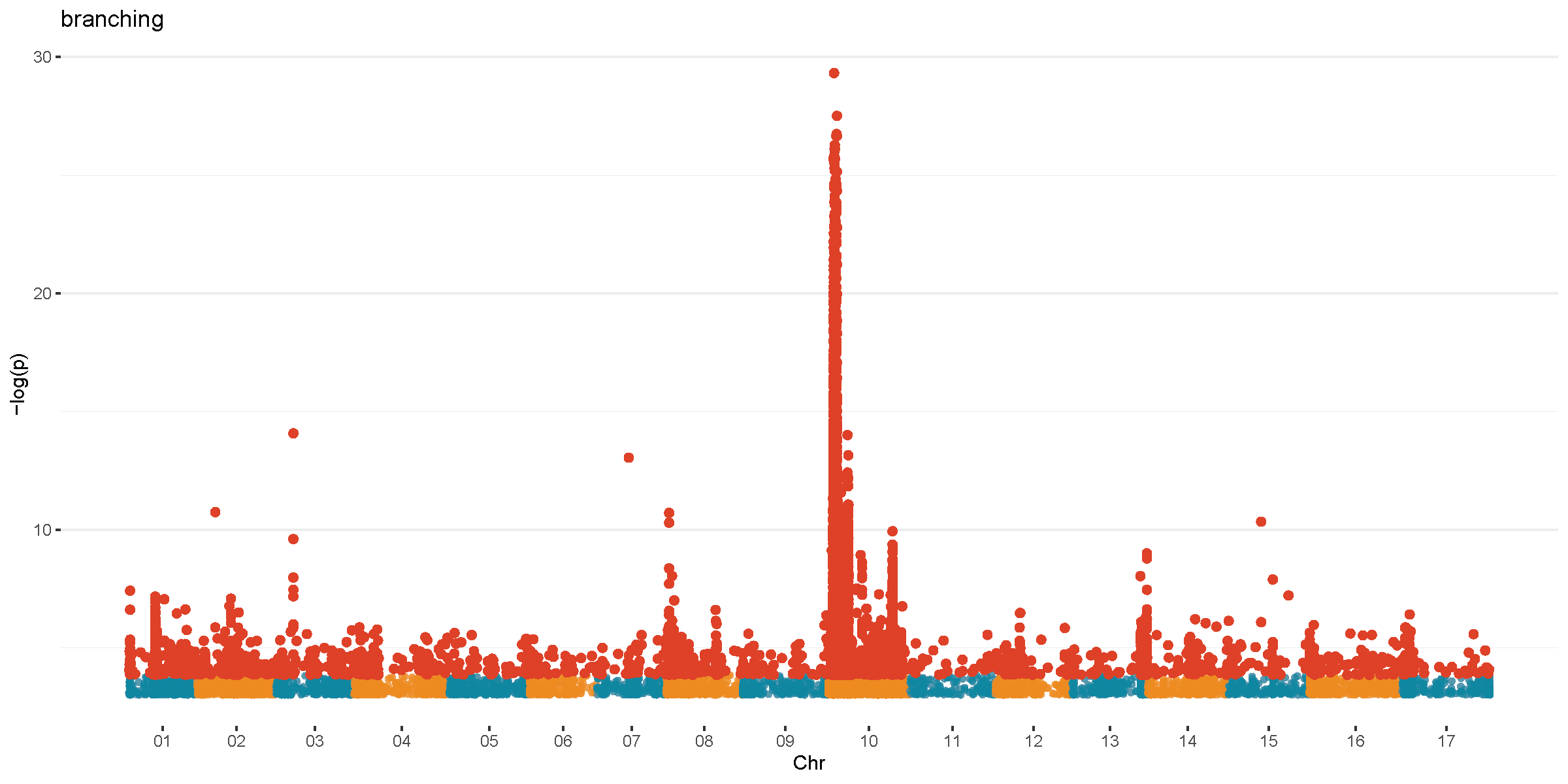

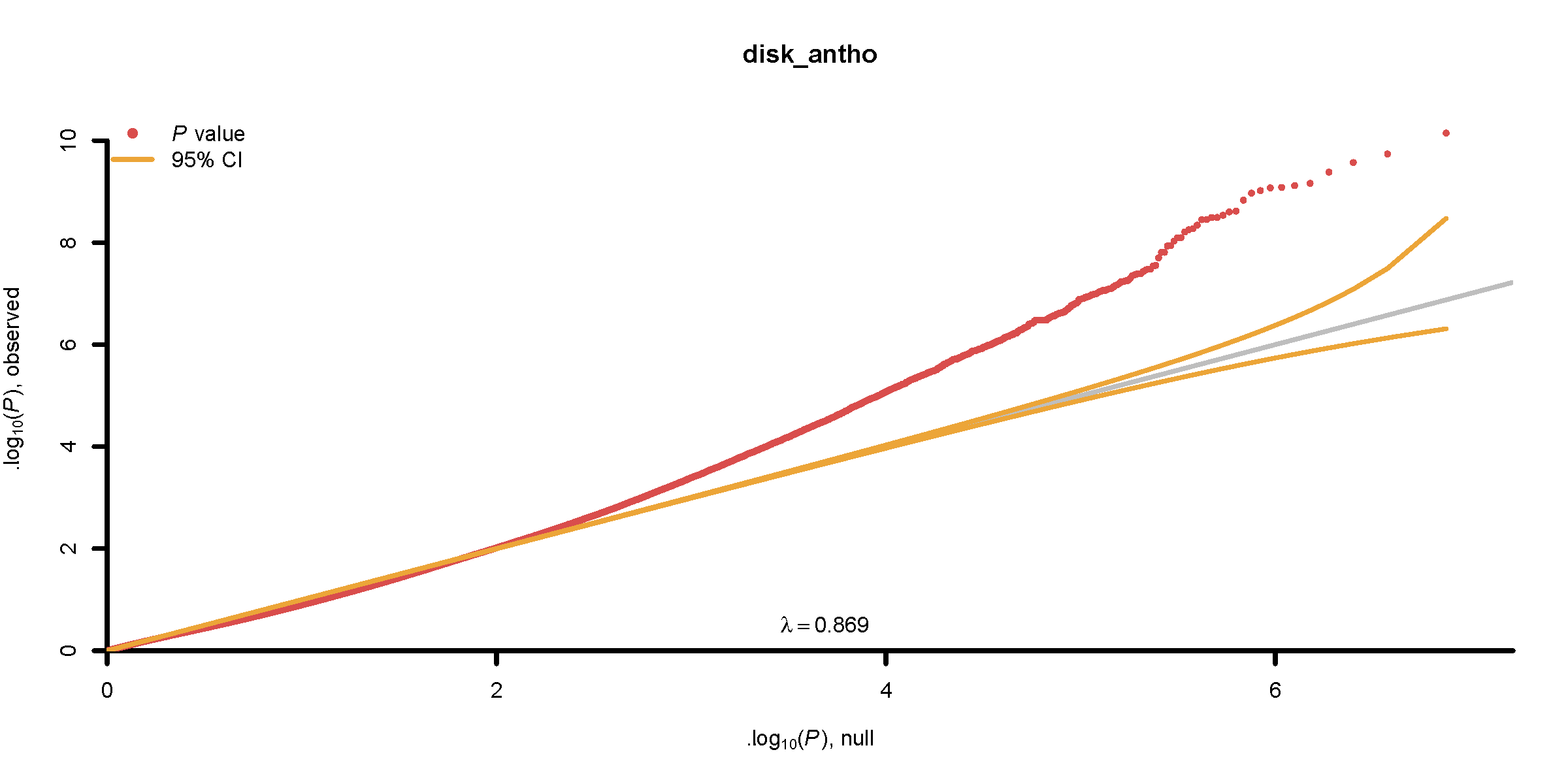

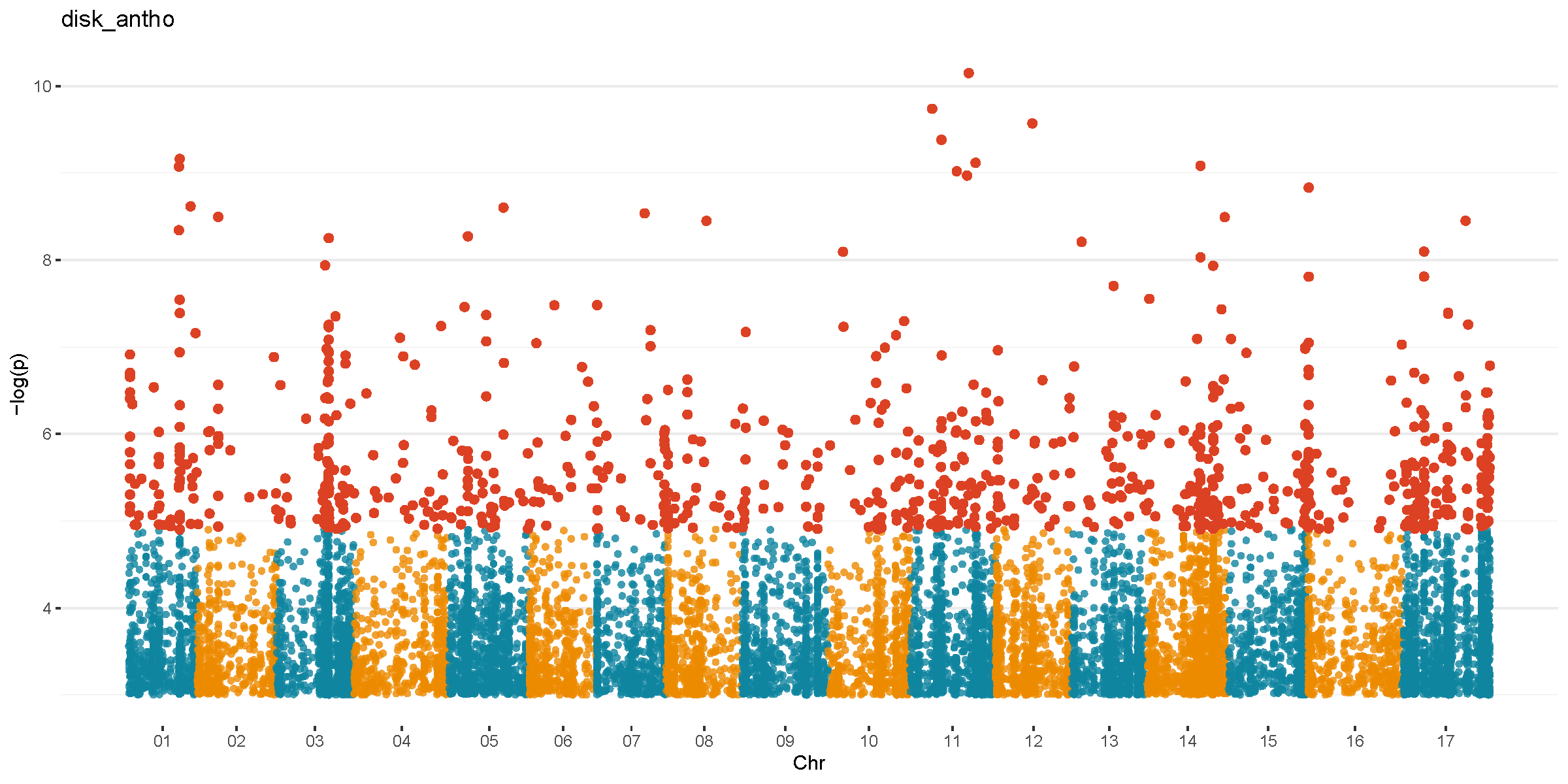

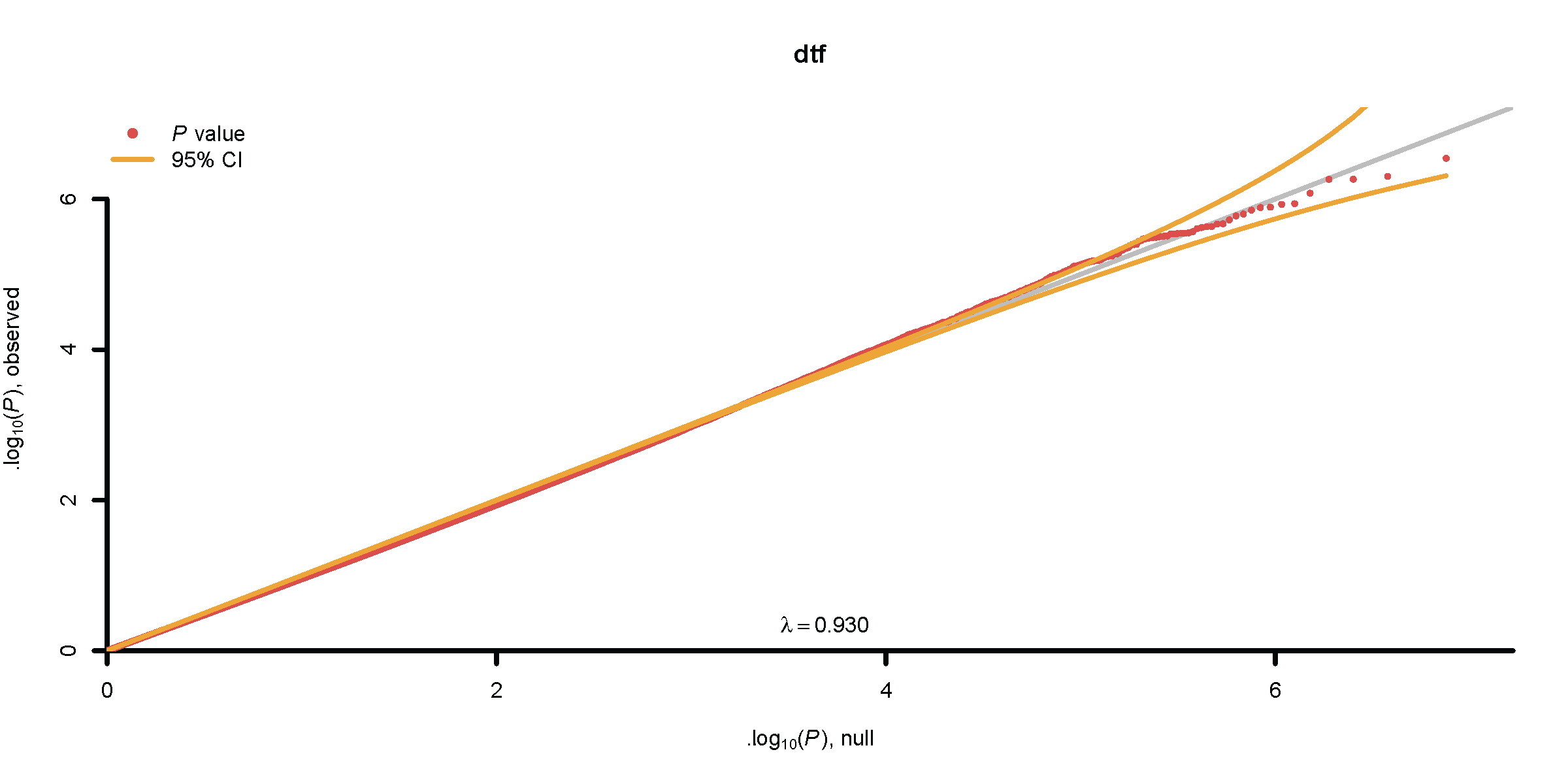

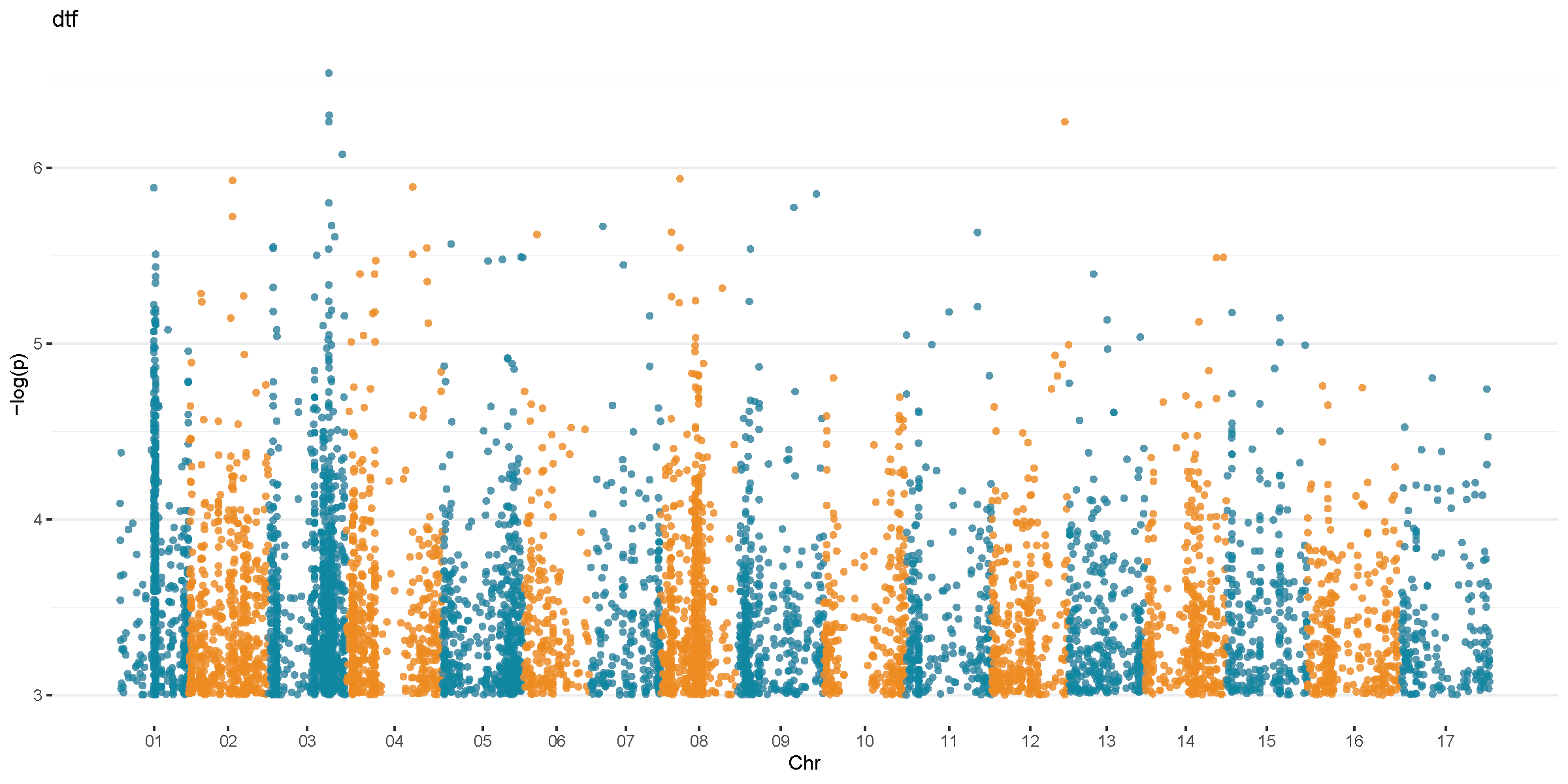

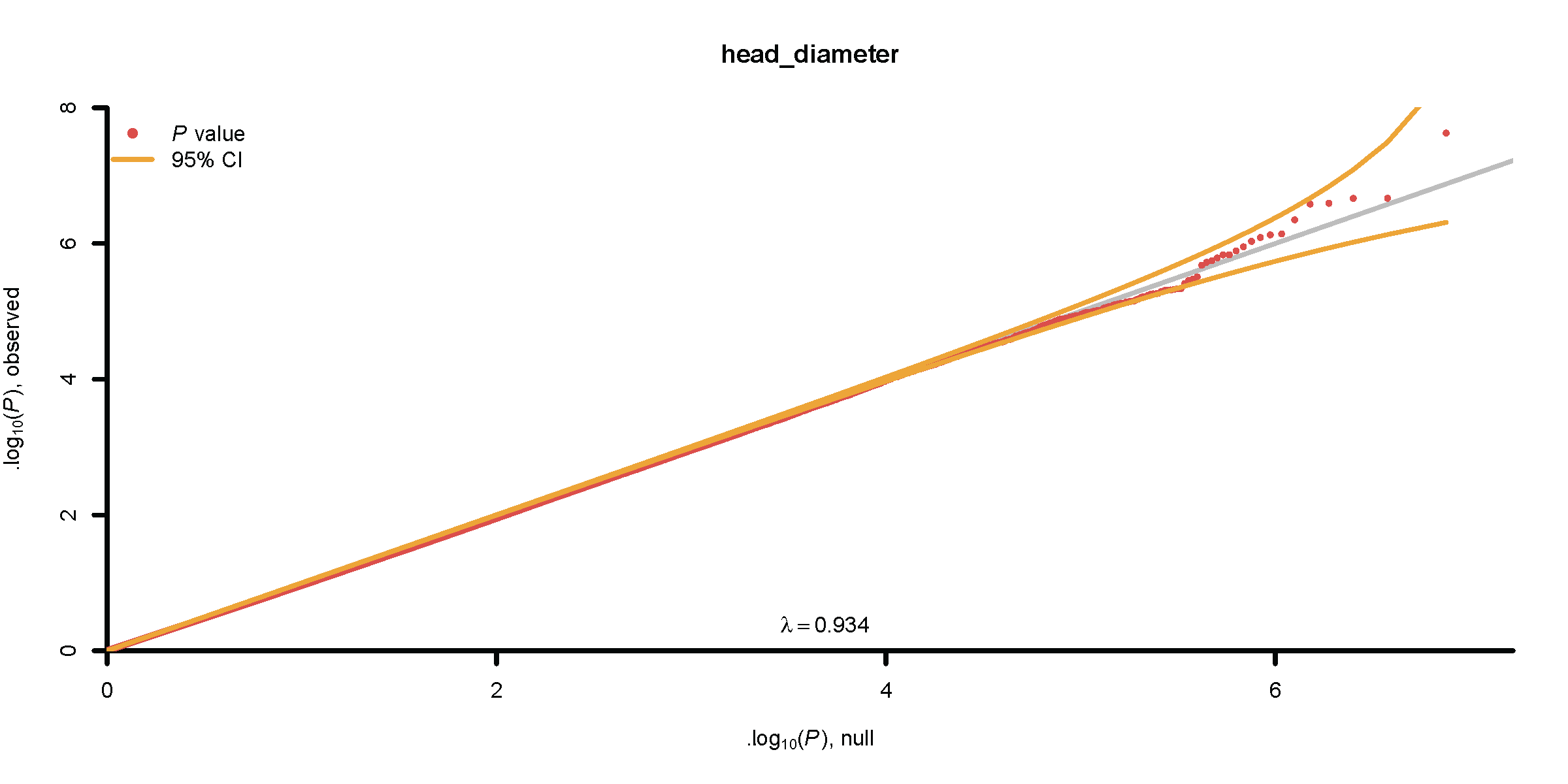

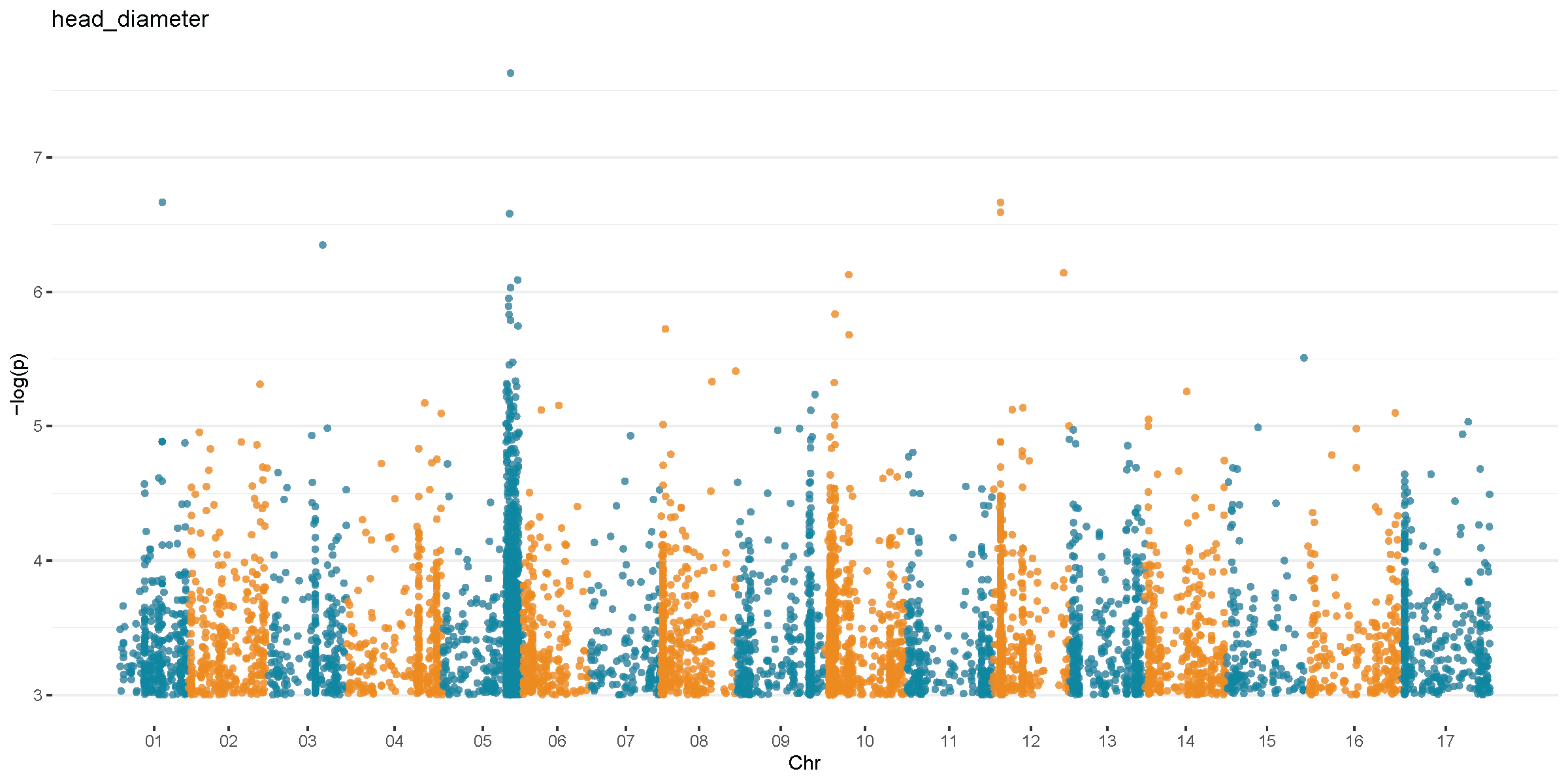

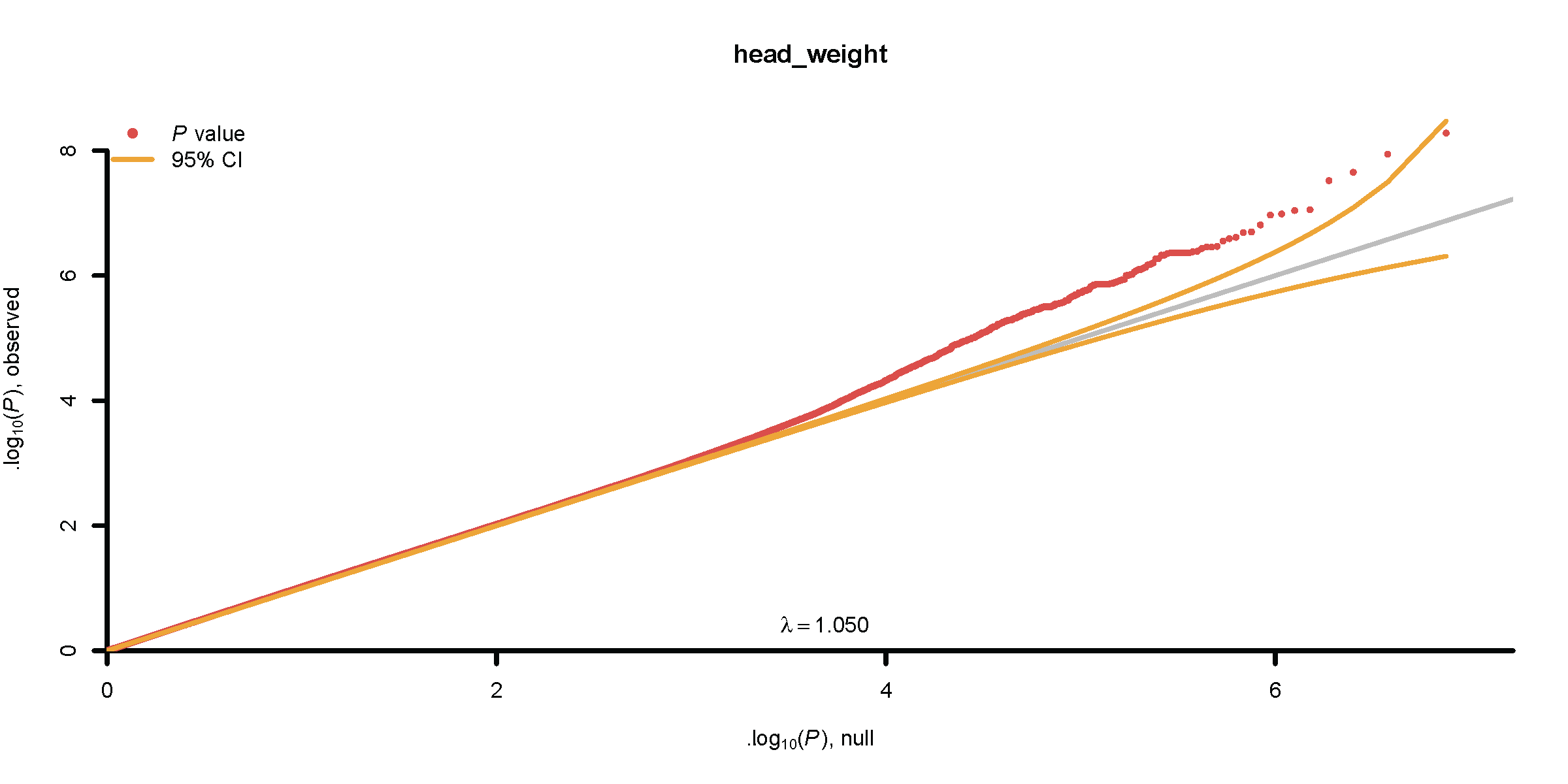

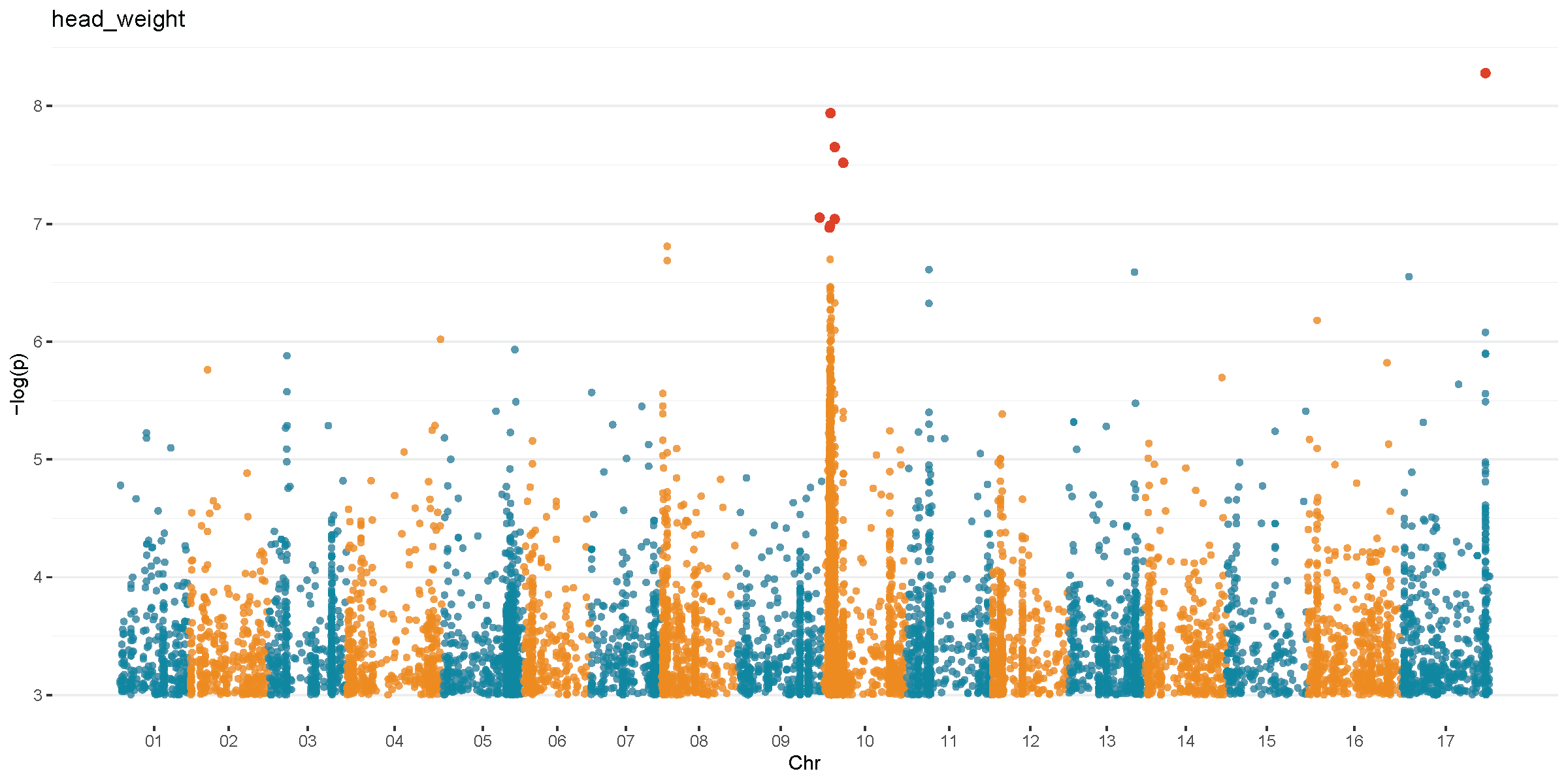

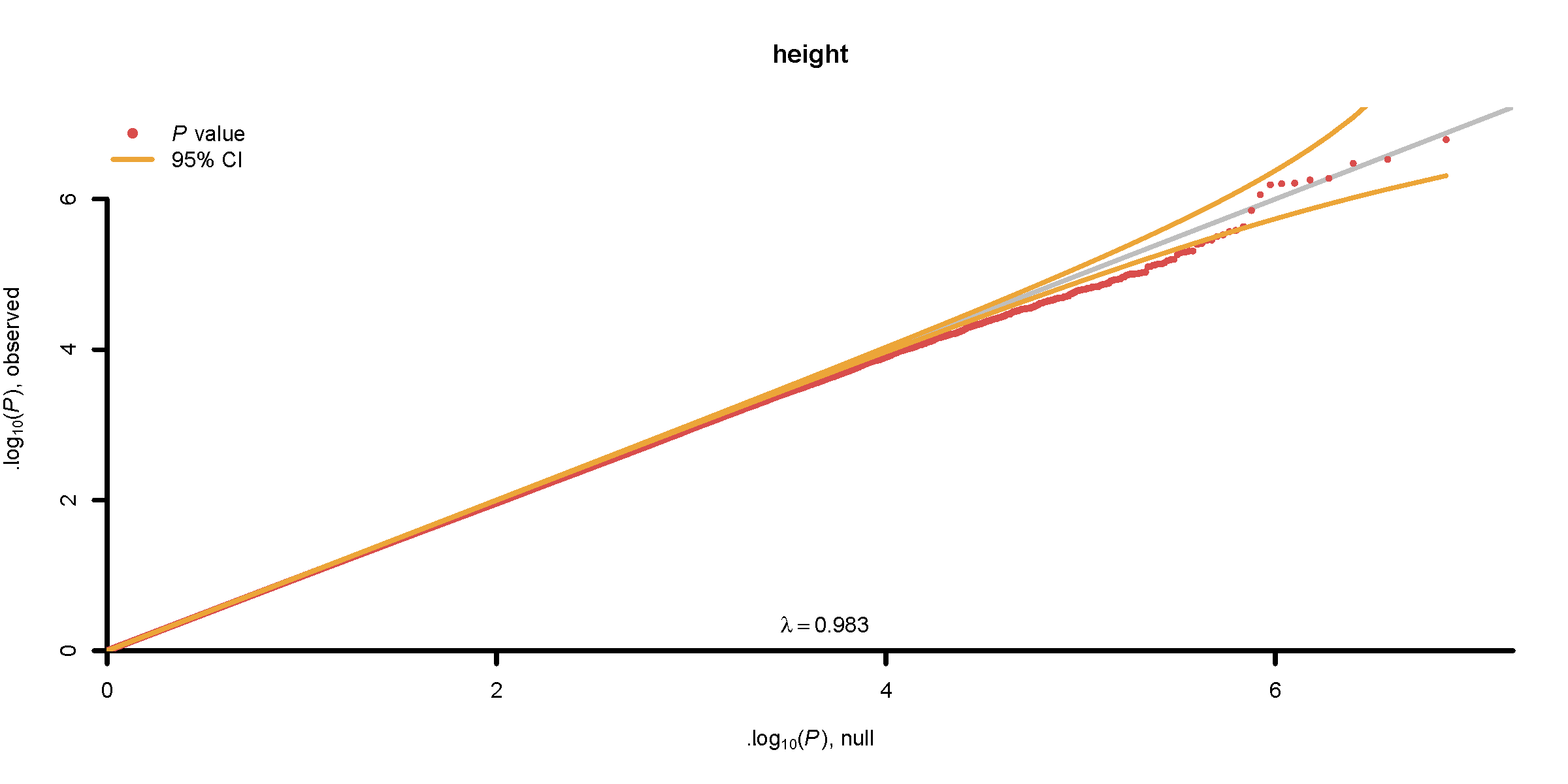

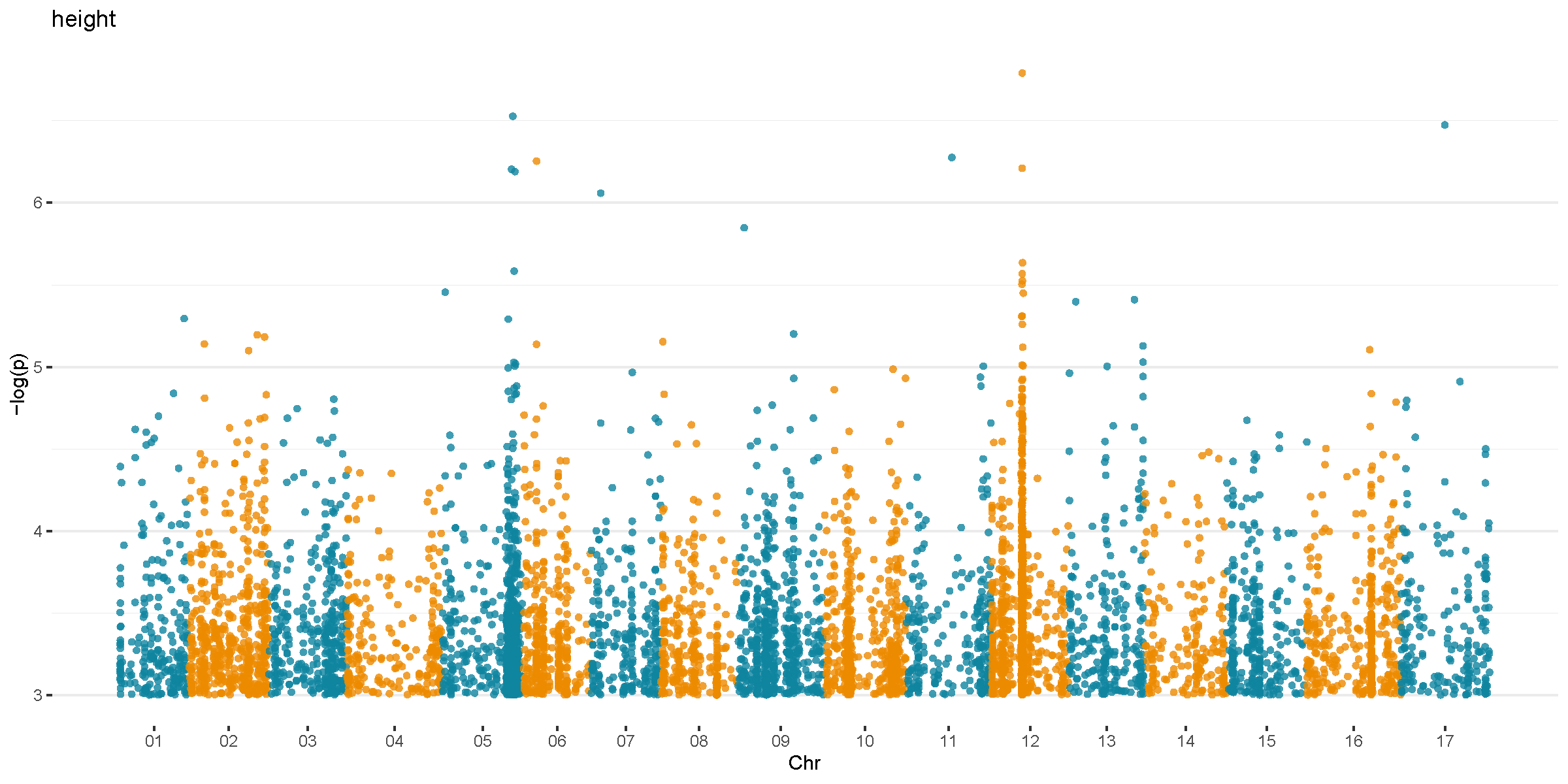

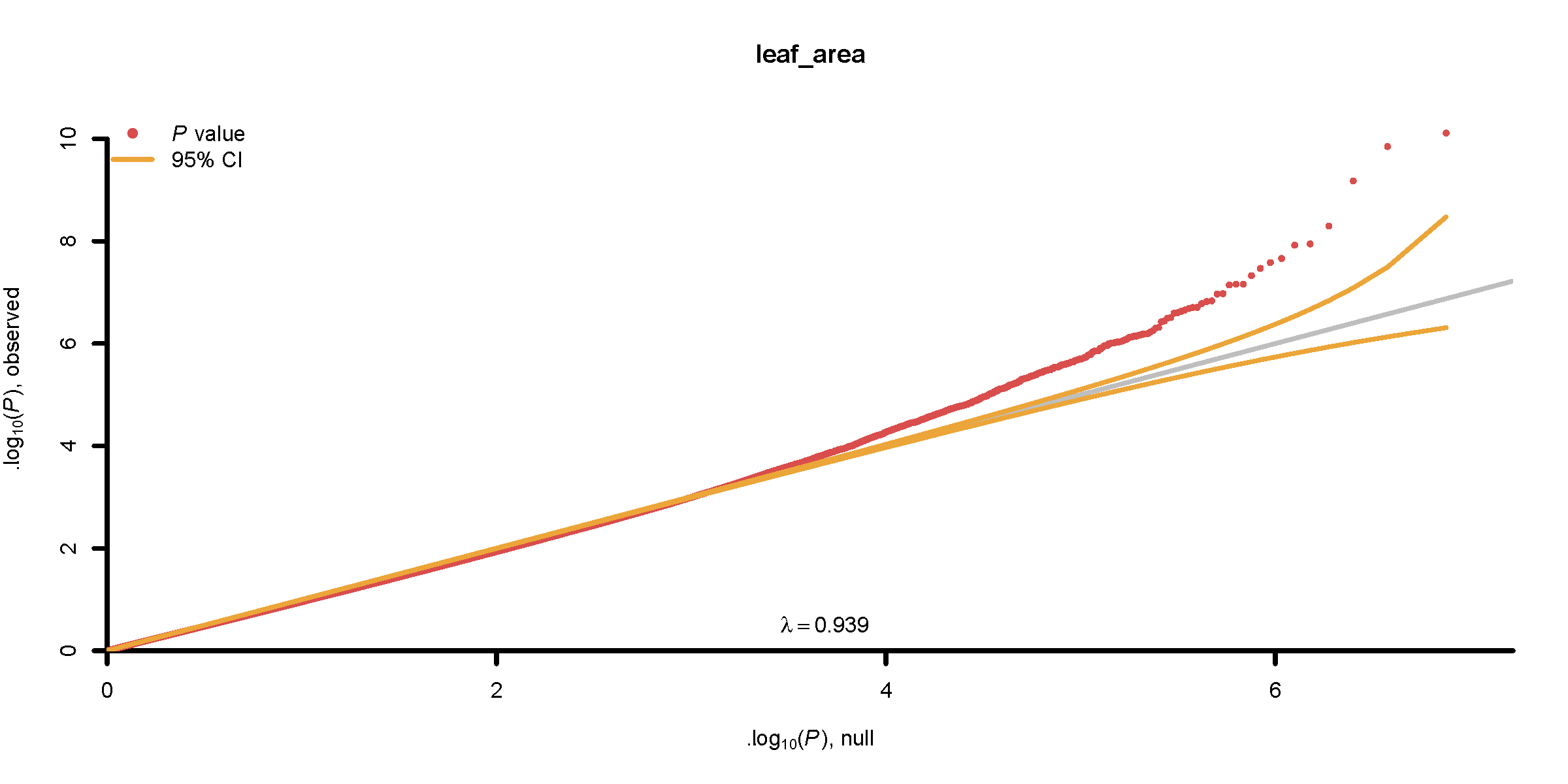

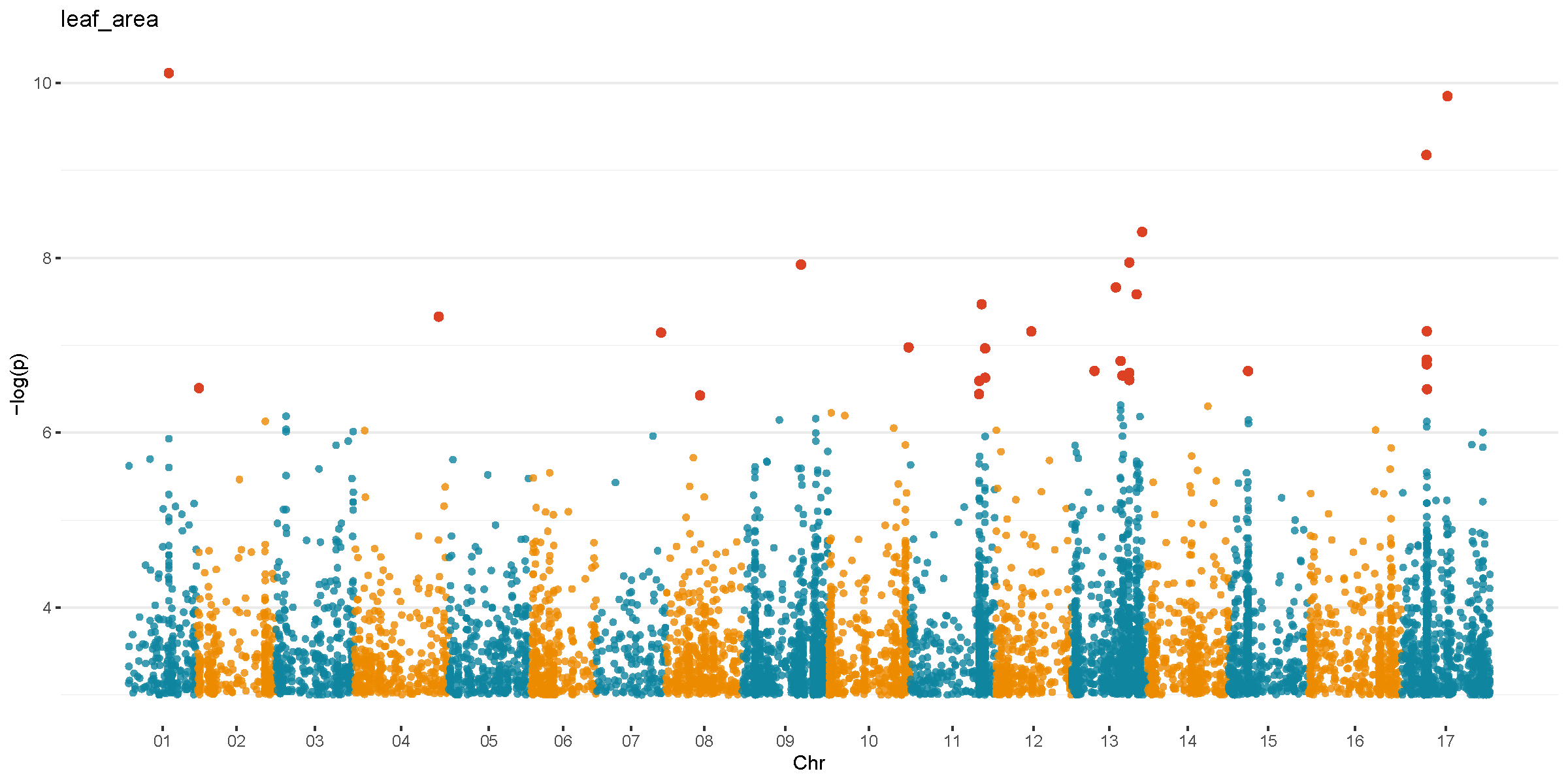

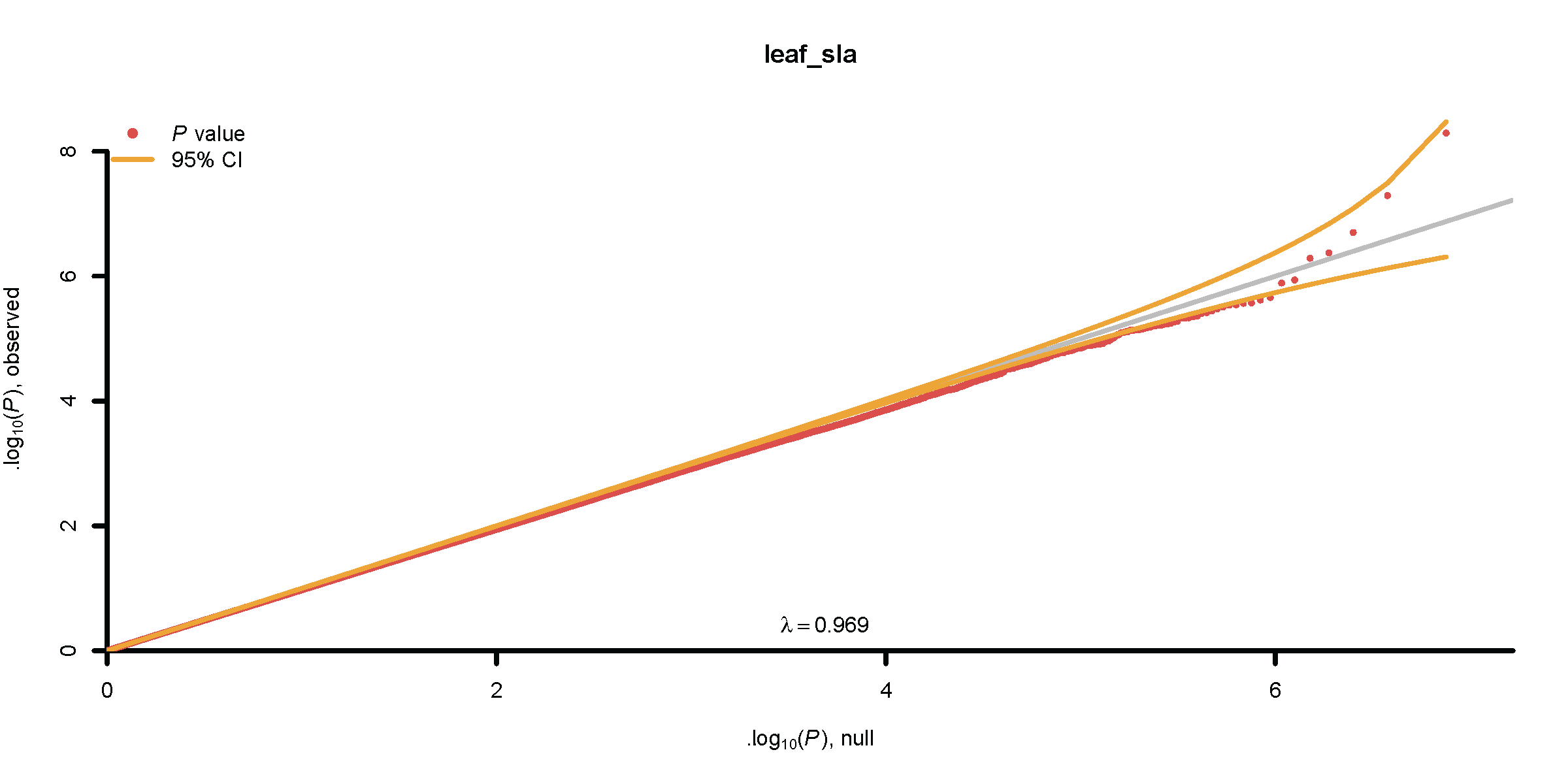

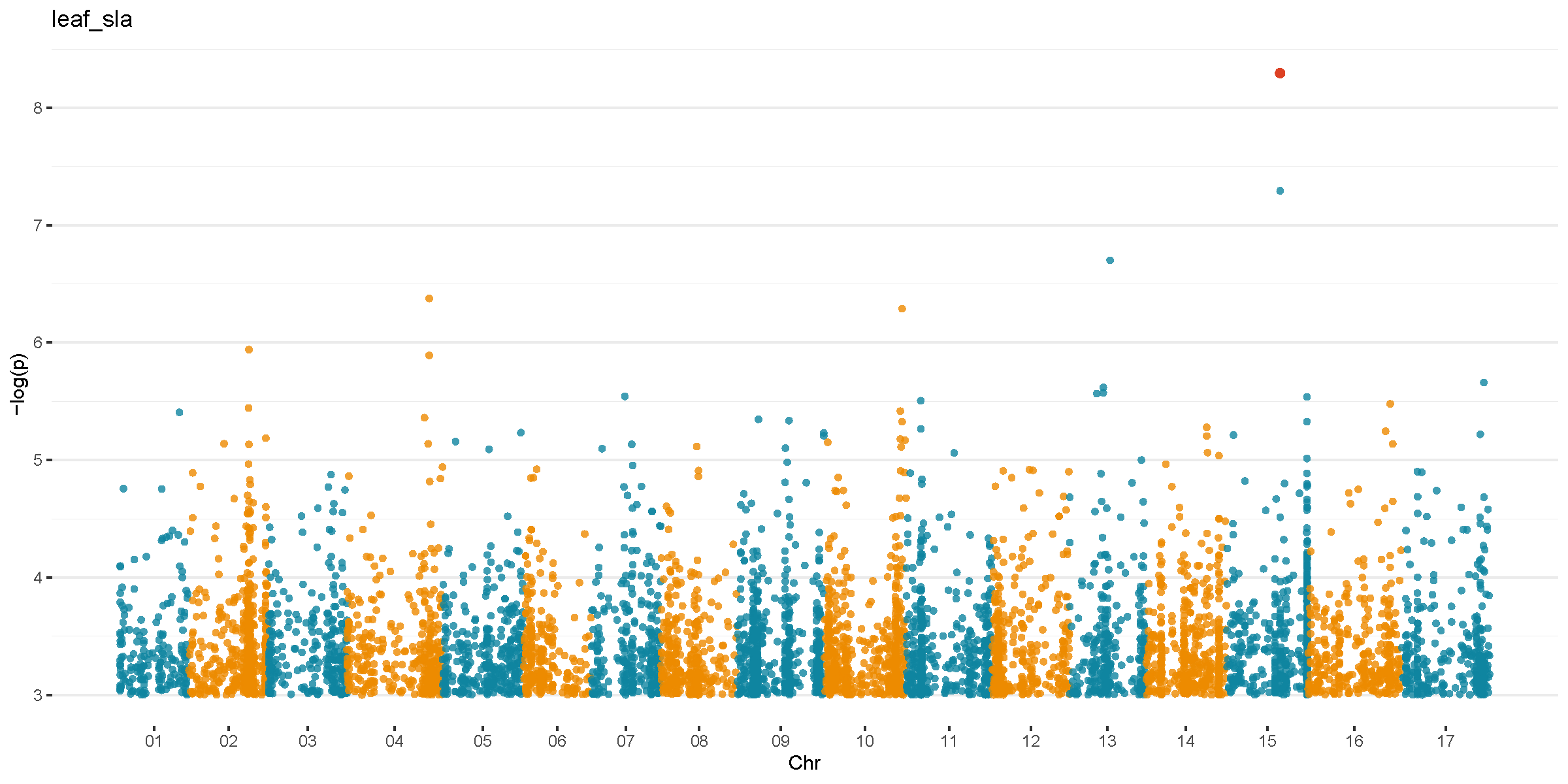

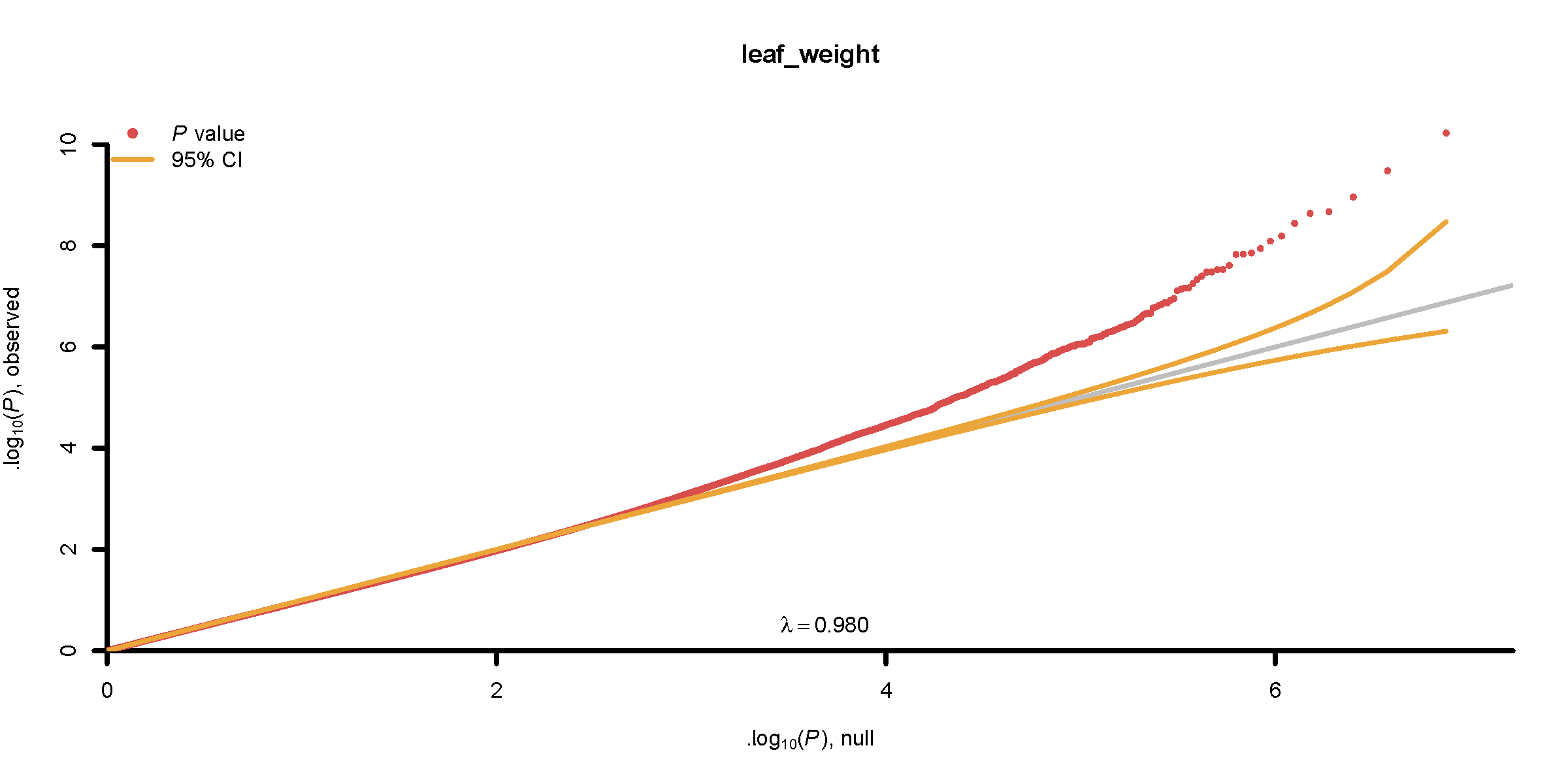

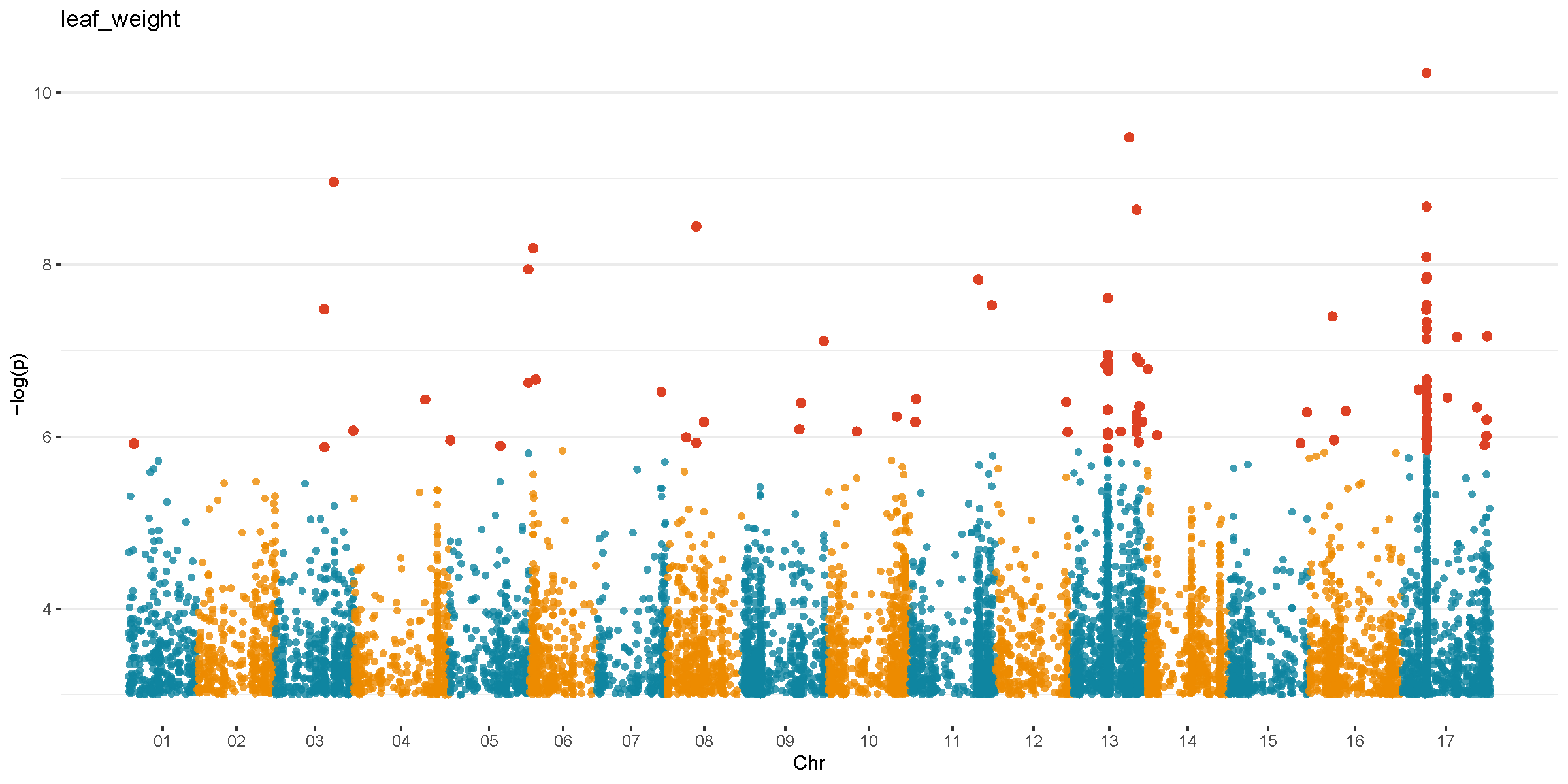

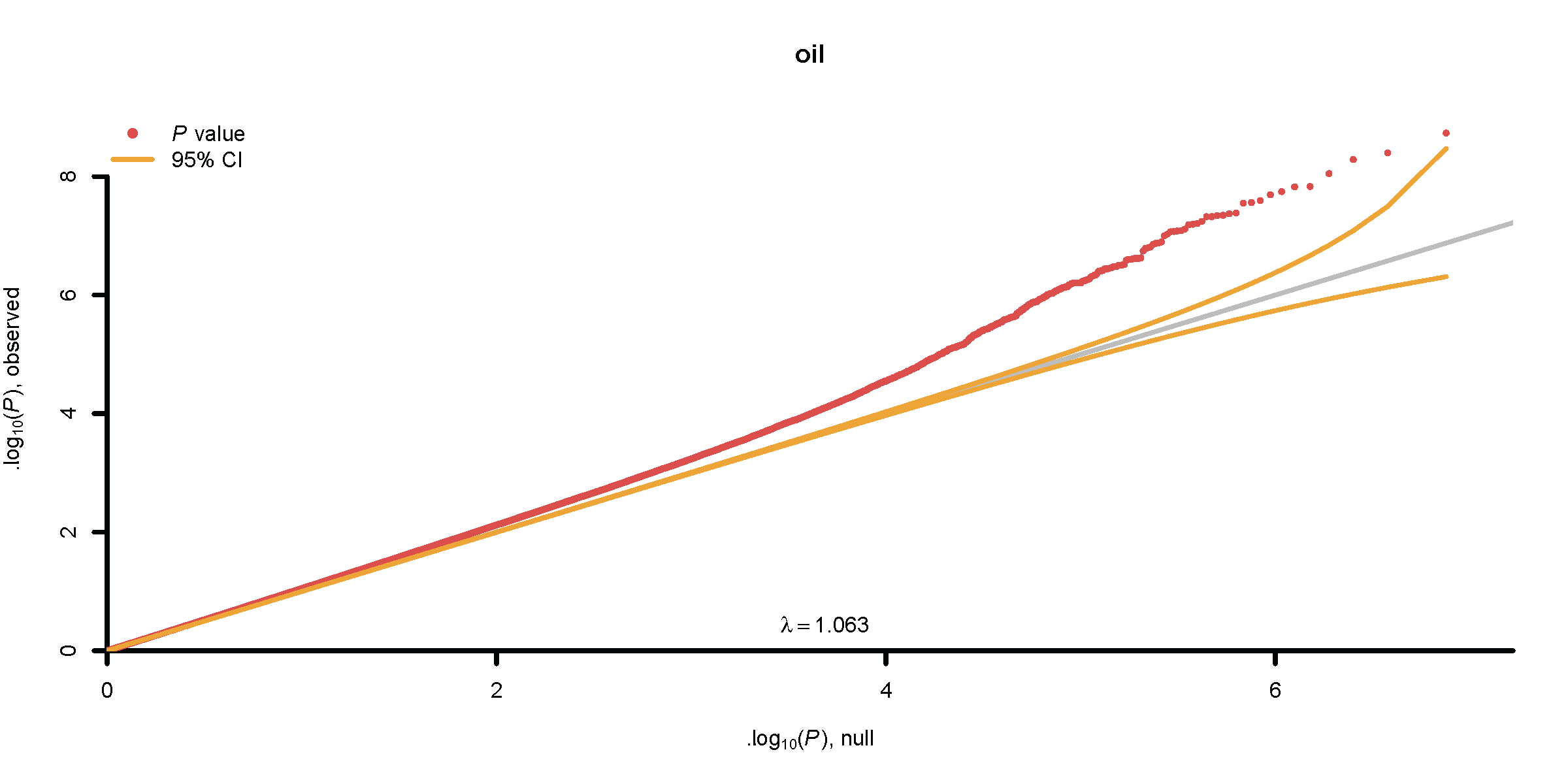

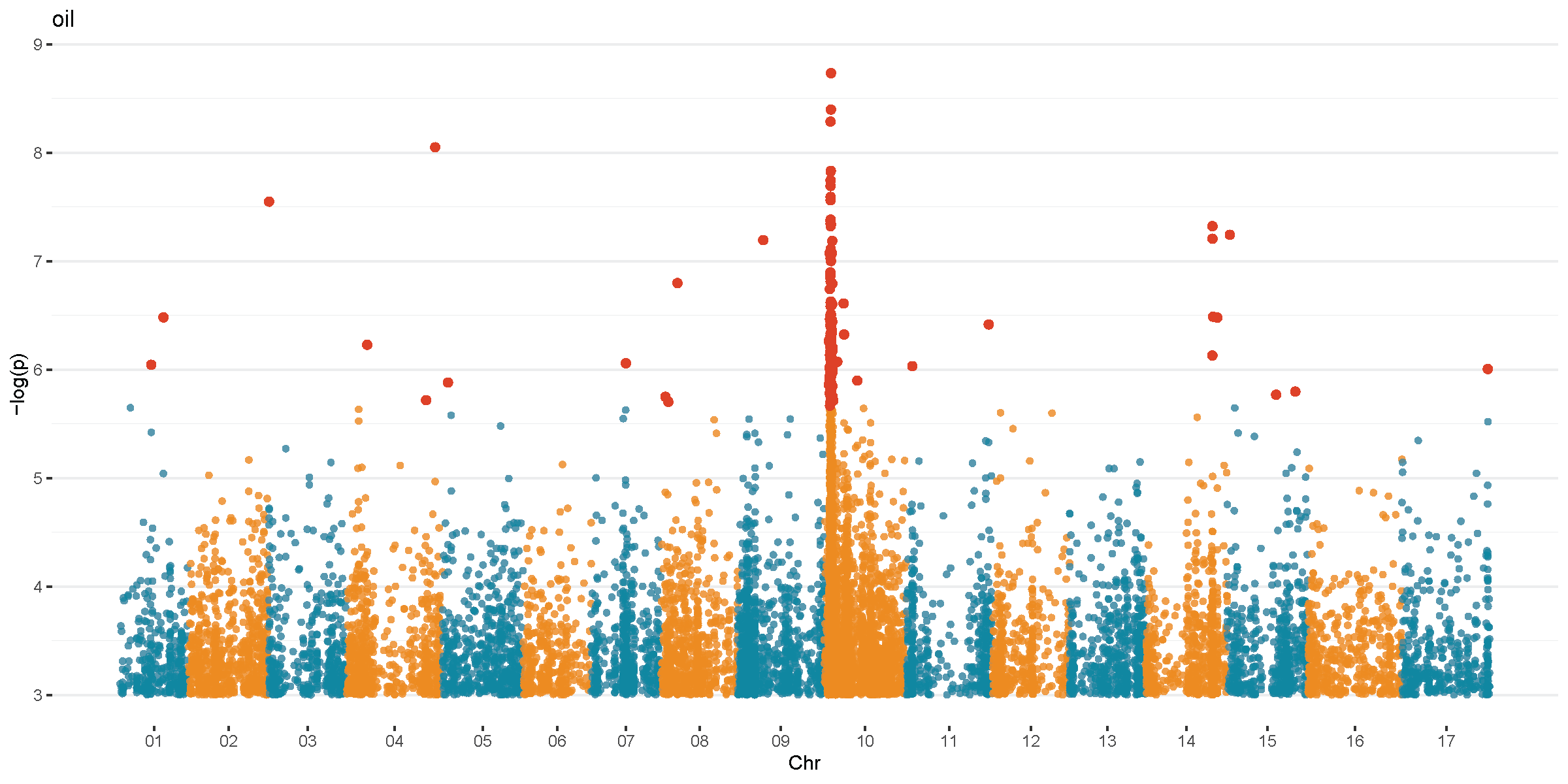

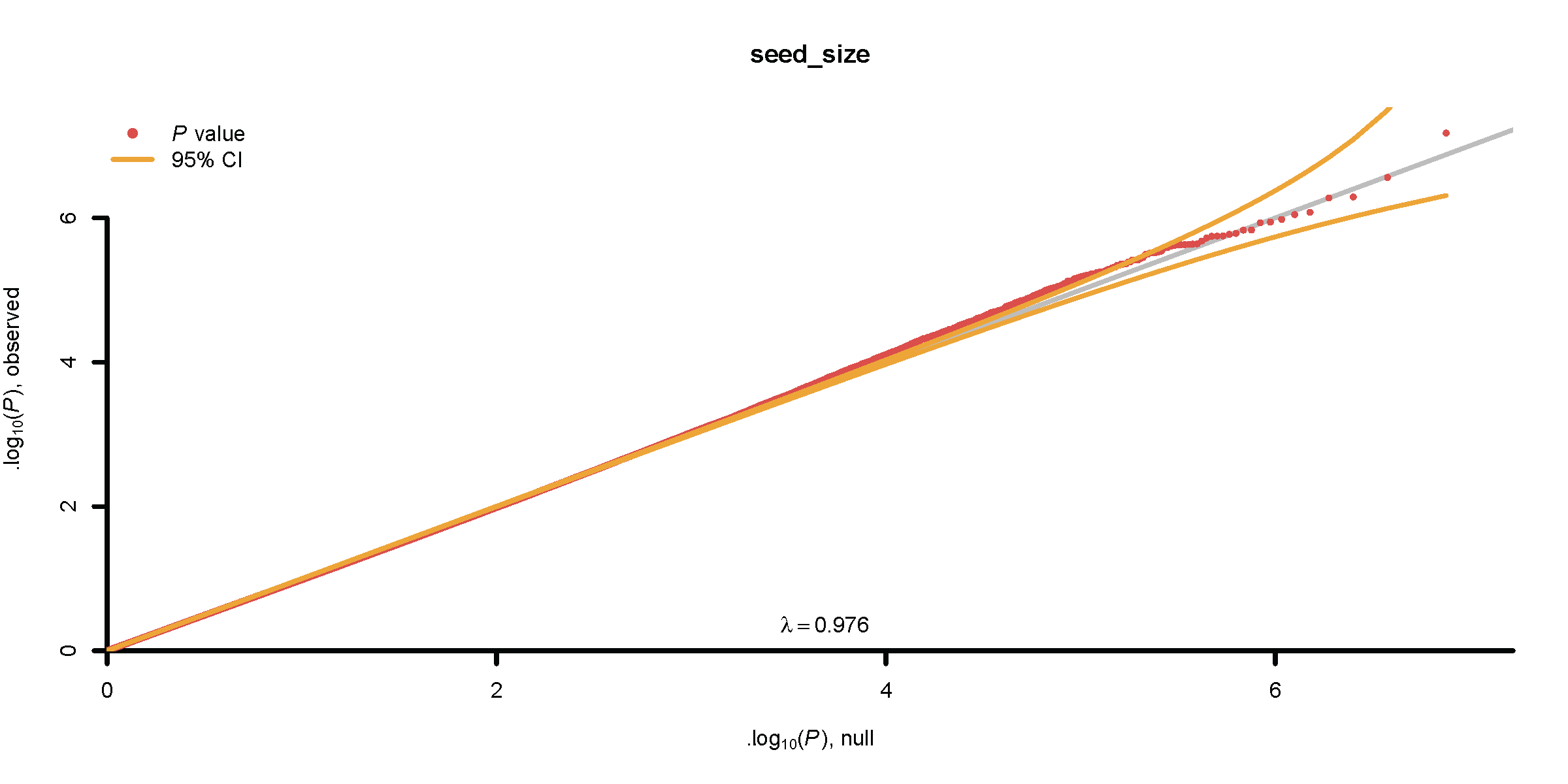

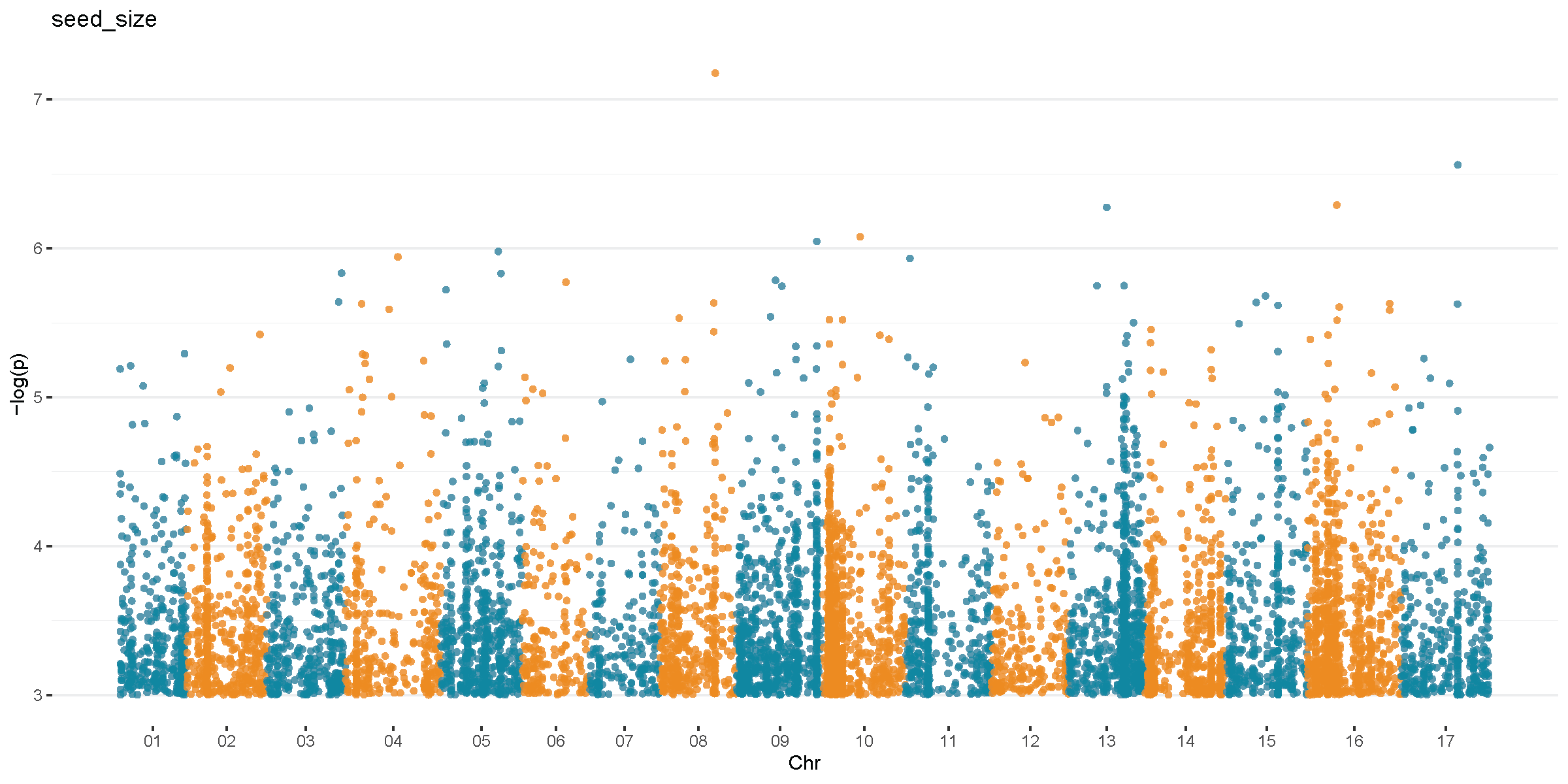

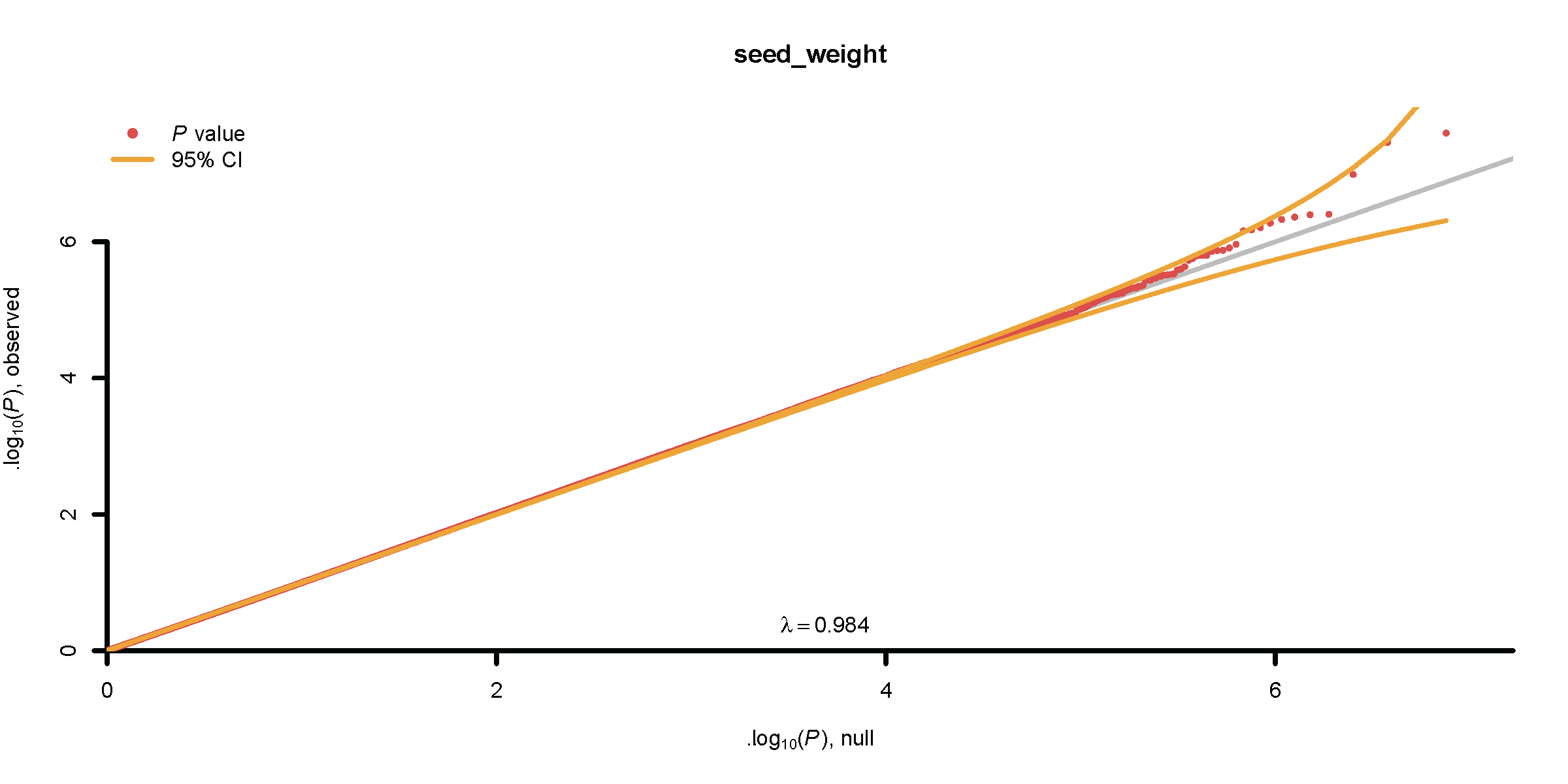

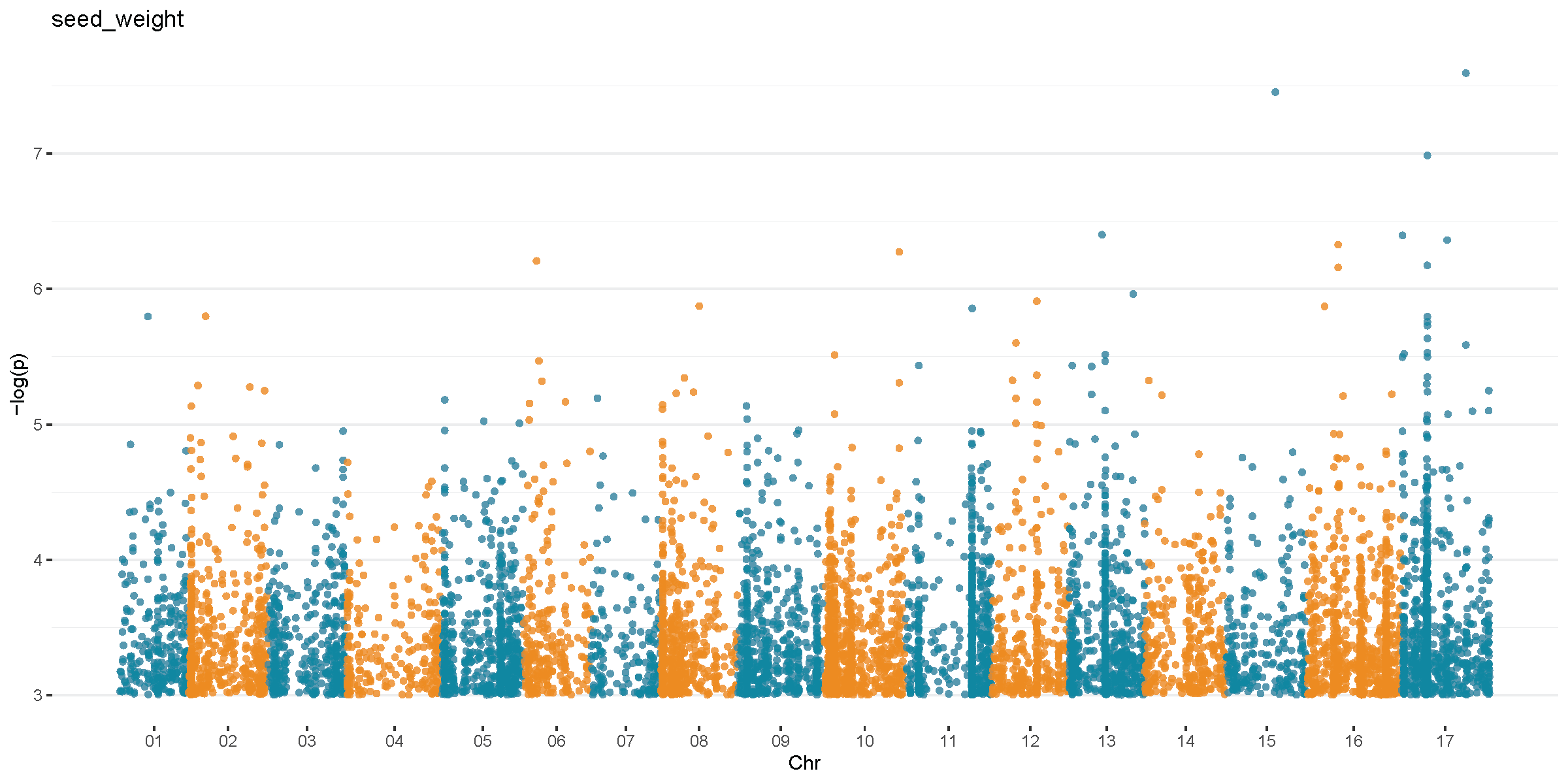

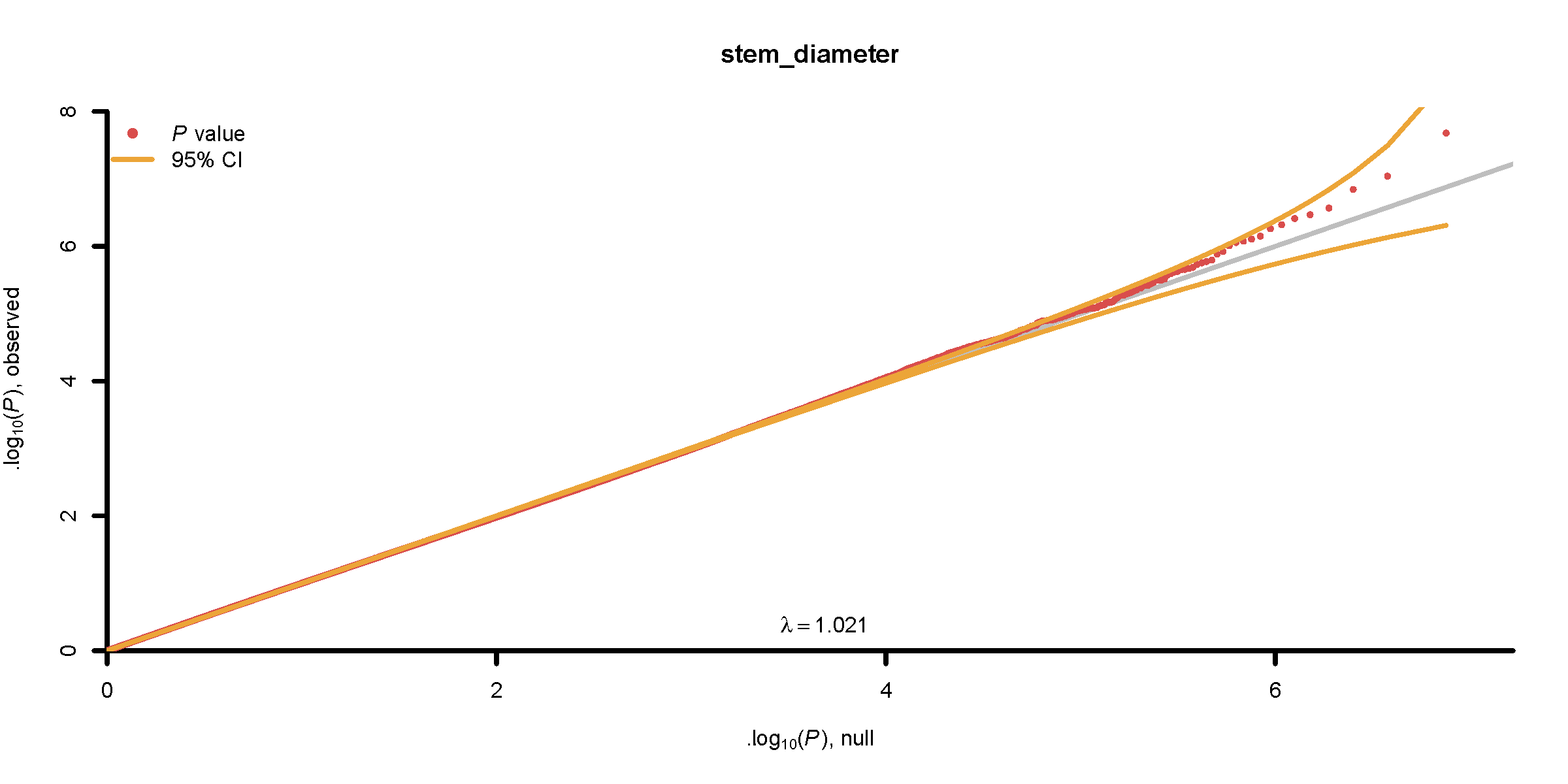

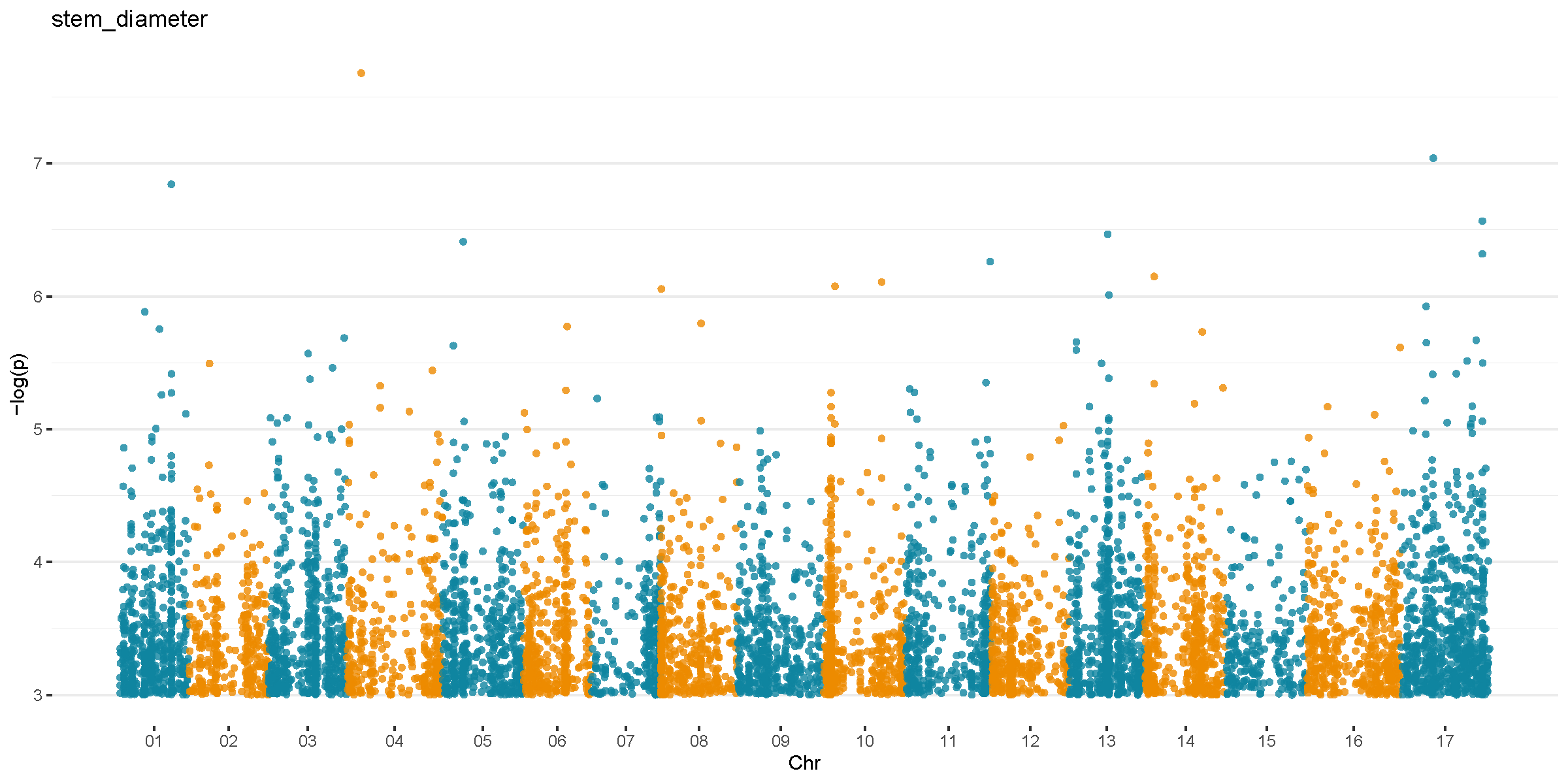

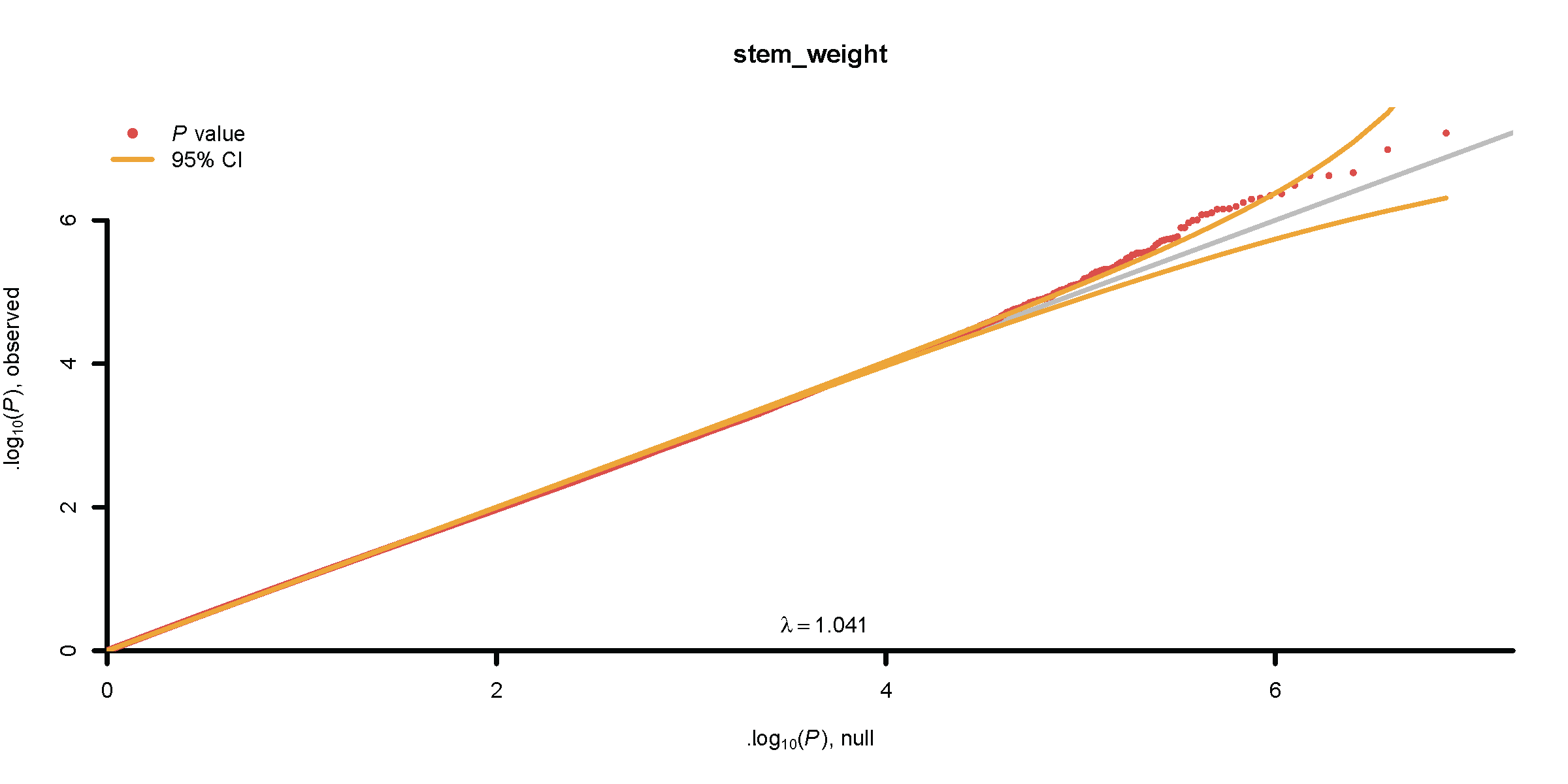

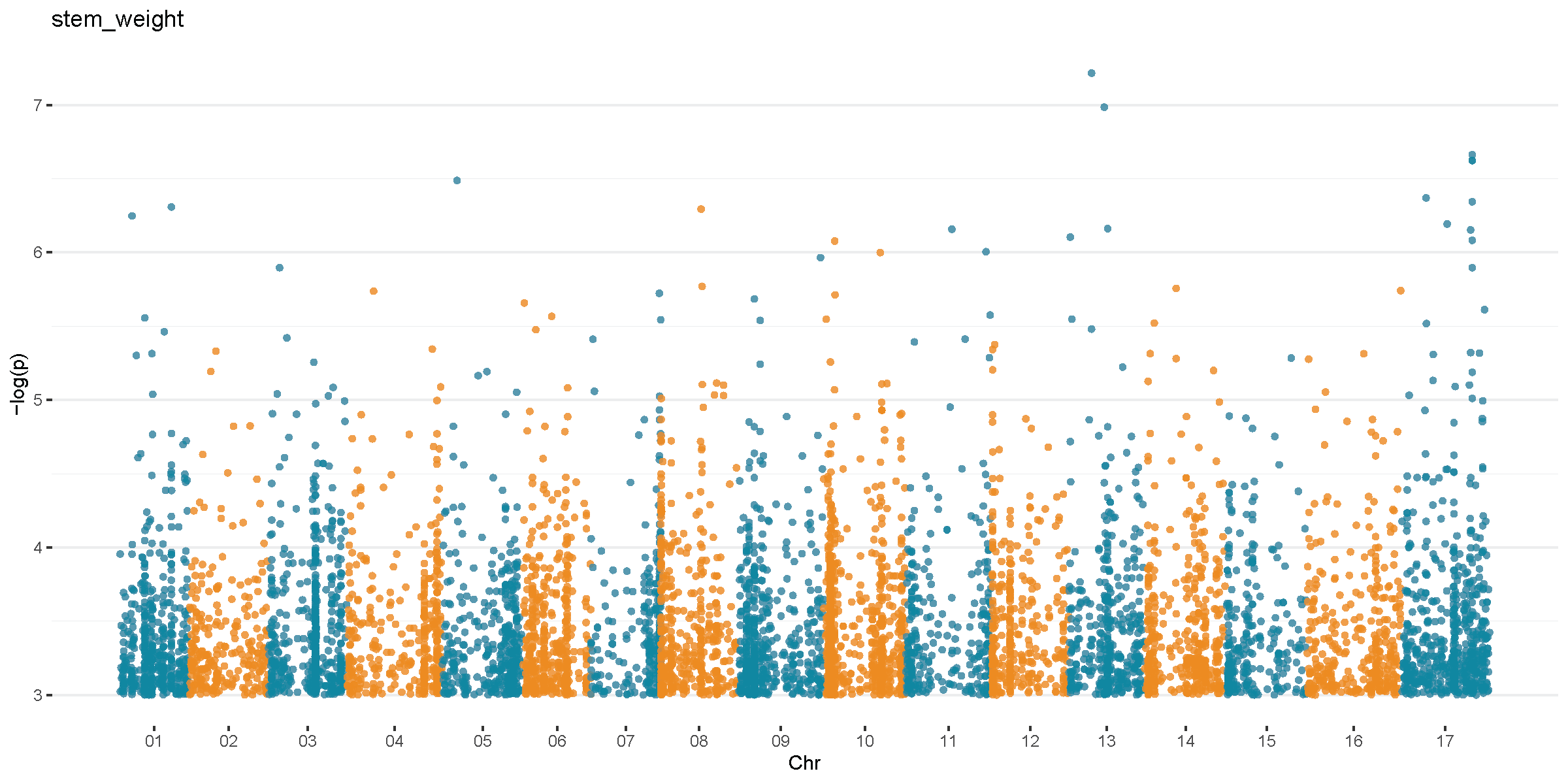

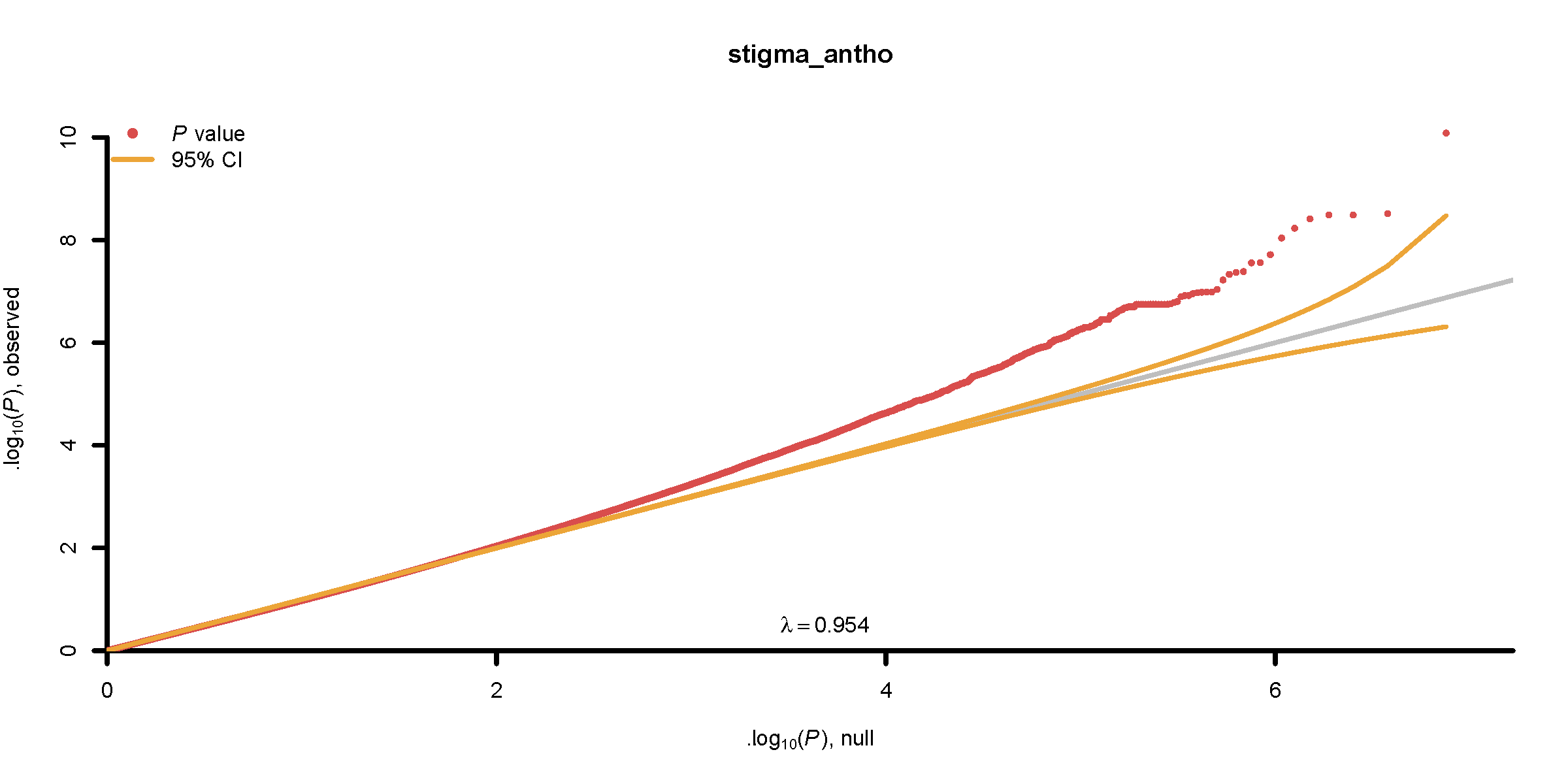

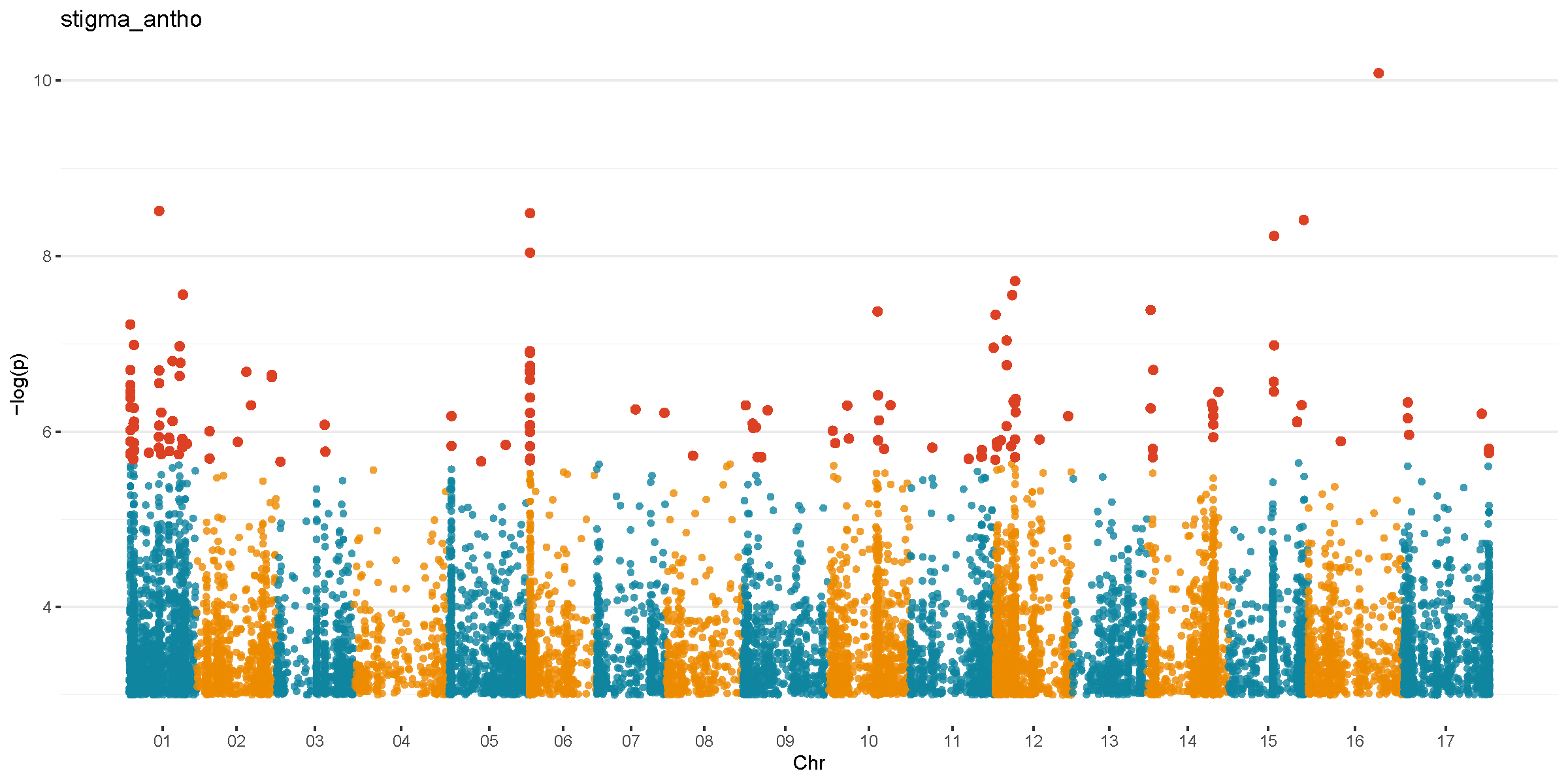


Supplementary Figure S2. Genome-wide association analyses with presence/absence variation. For each trait, panel A represents a quantile-quantile plot of p-values for tested PAV regions using qqPlotFast in ramwas. Values above the diagonal line indicate higher significance than expected based on a normal distribution. Panel B is a manhattan plot of -log10(p-values) for PAV regions. Significance was determined using a q-value correction with a false discovery rate of 0.1. Significant regions are highlighted in red. using a q-value correction with a FDR of 10%. Data collected at two field sites near Watkinsville, GA, USA.


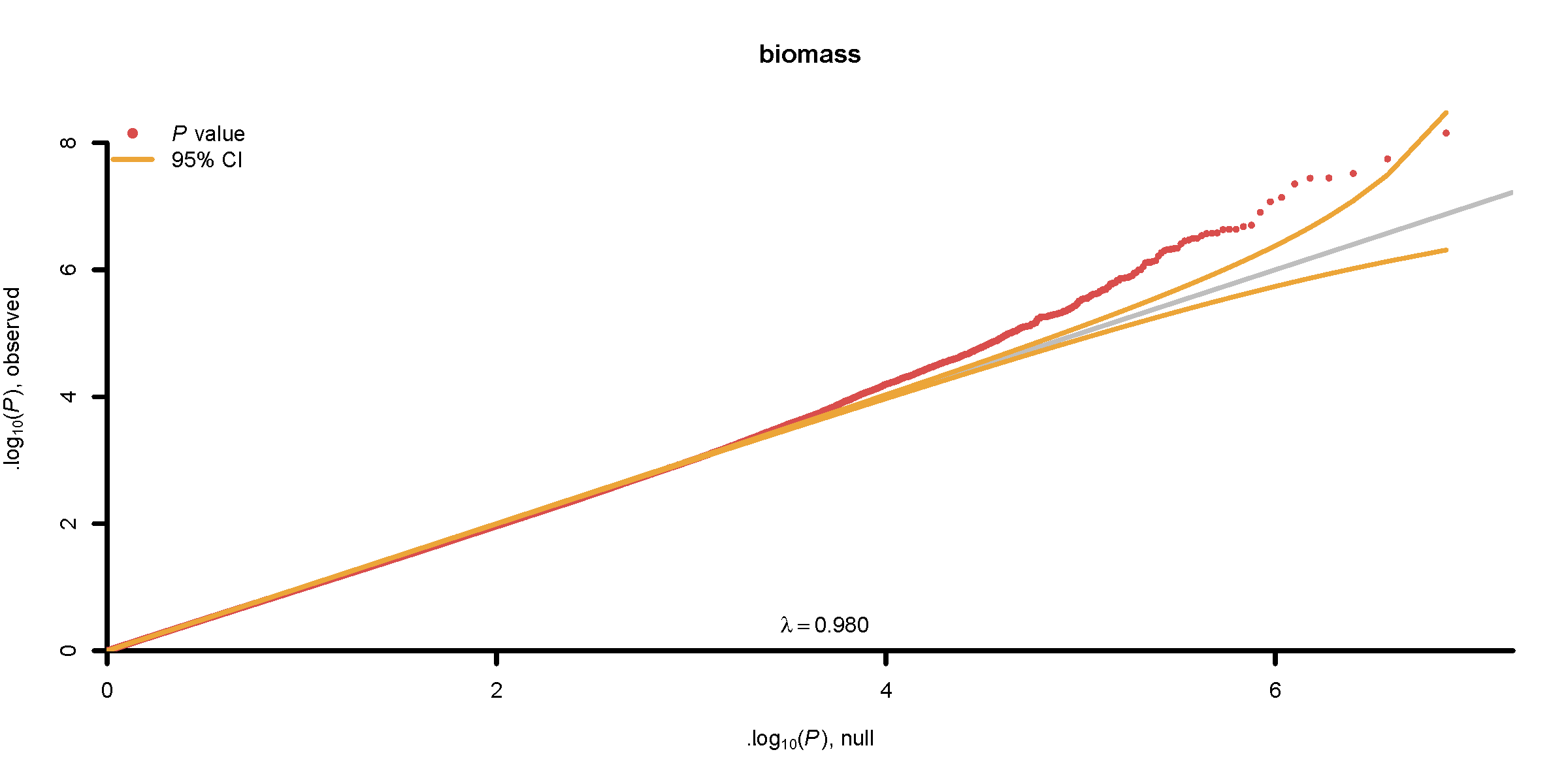

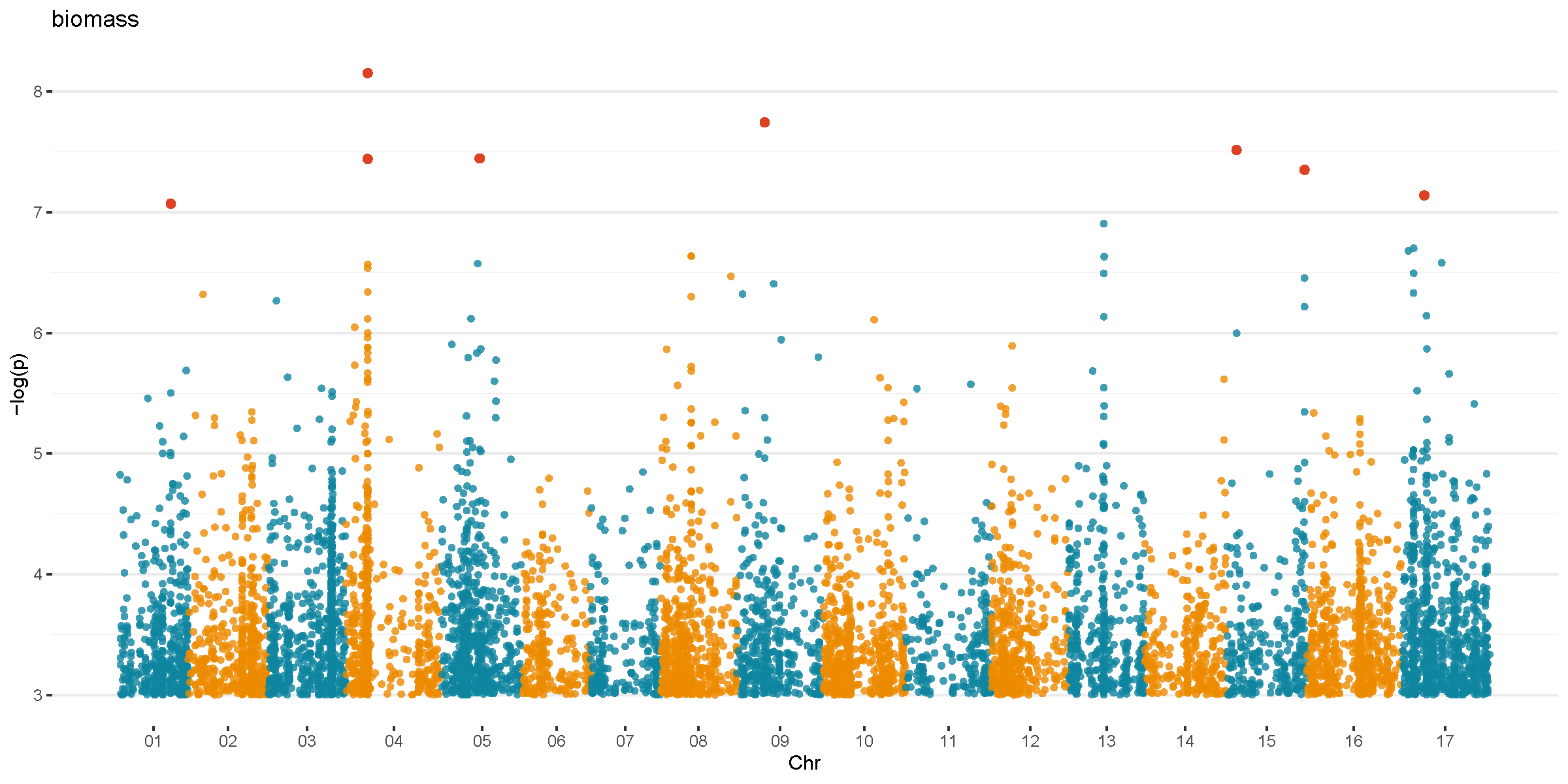

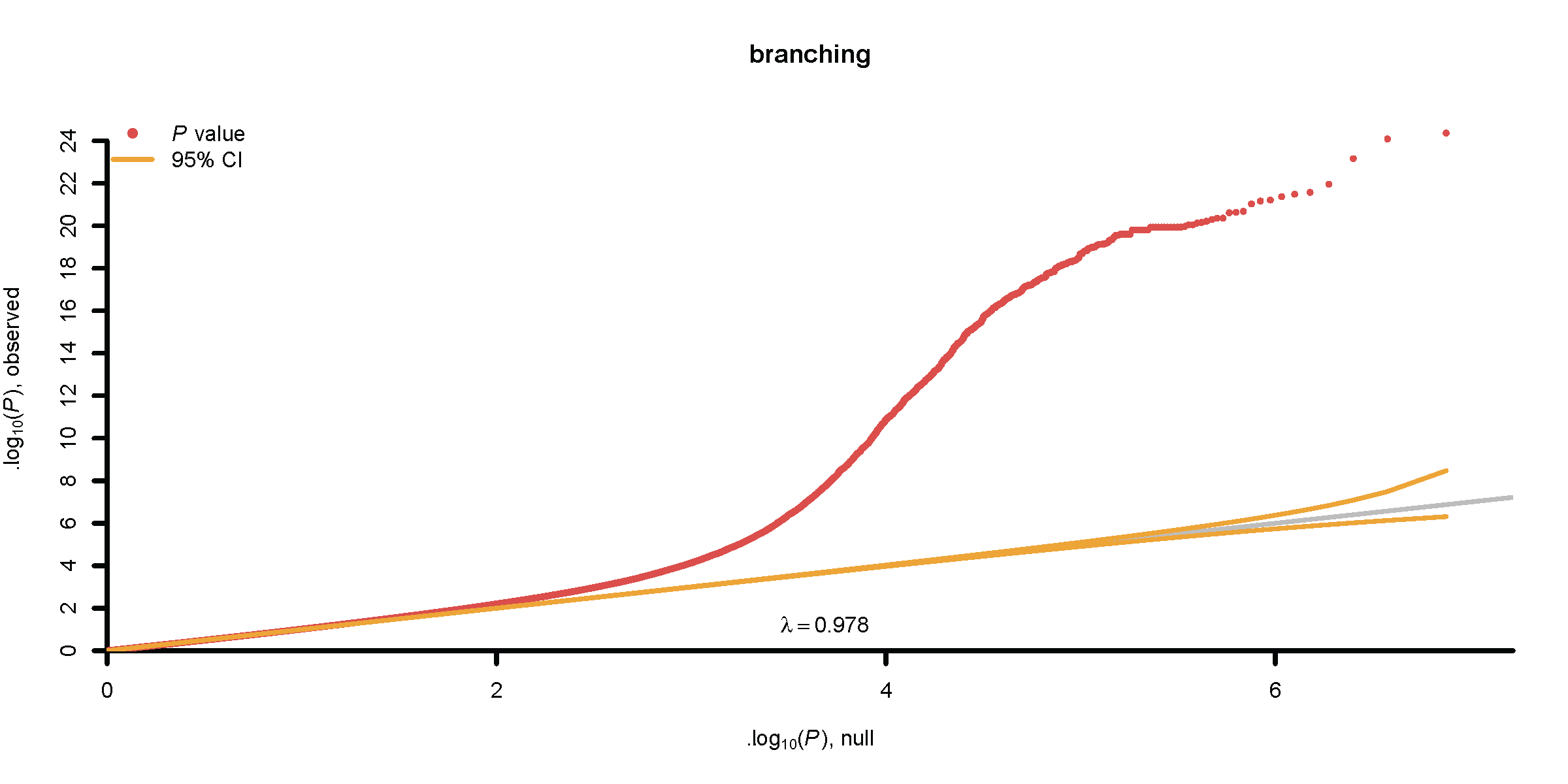

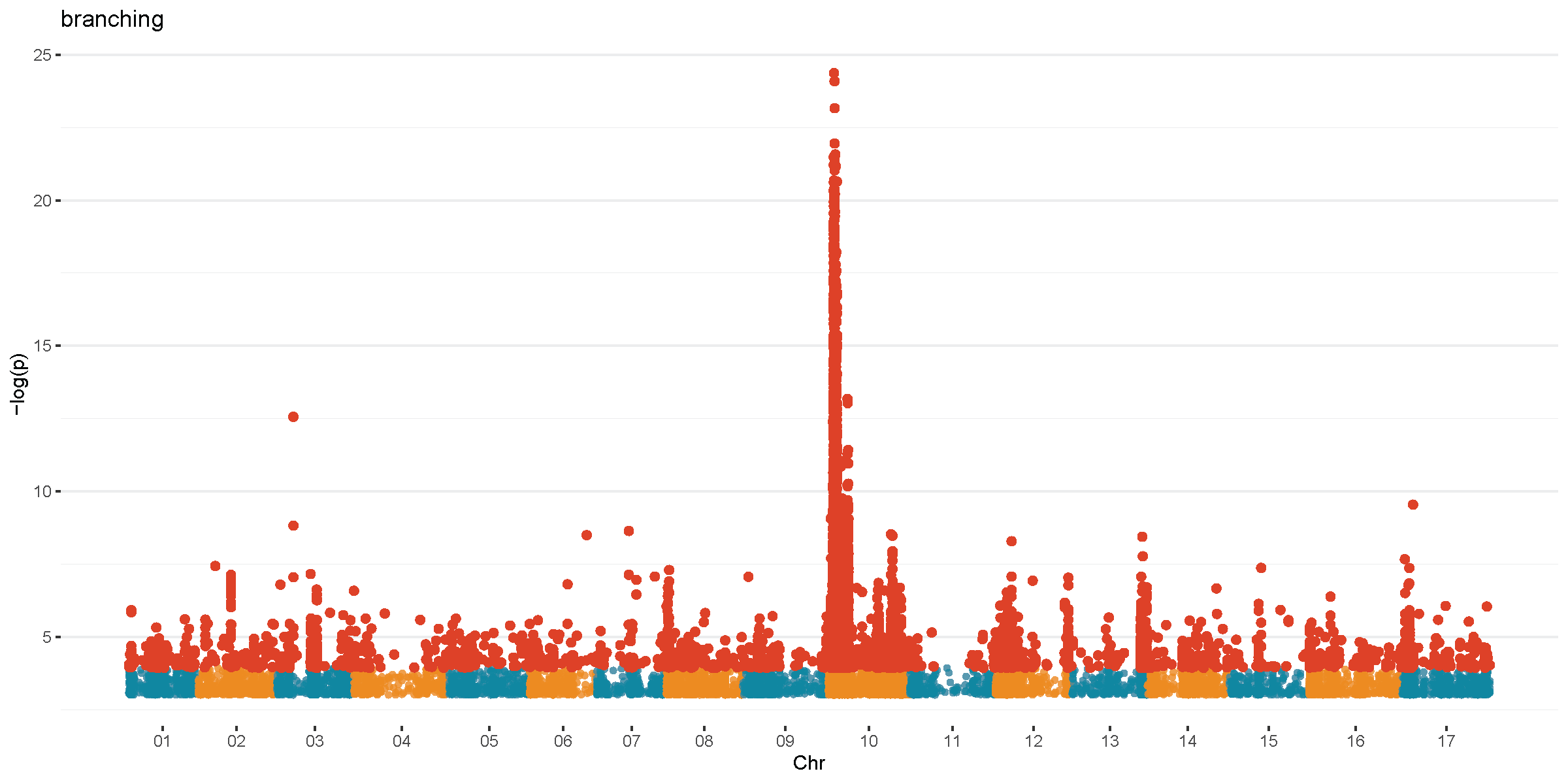

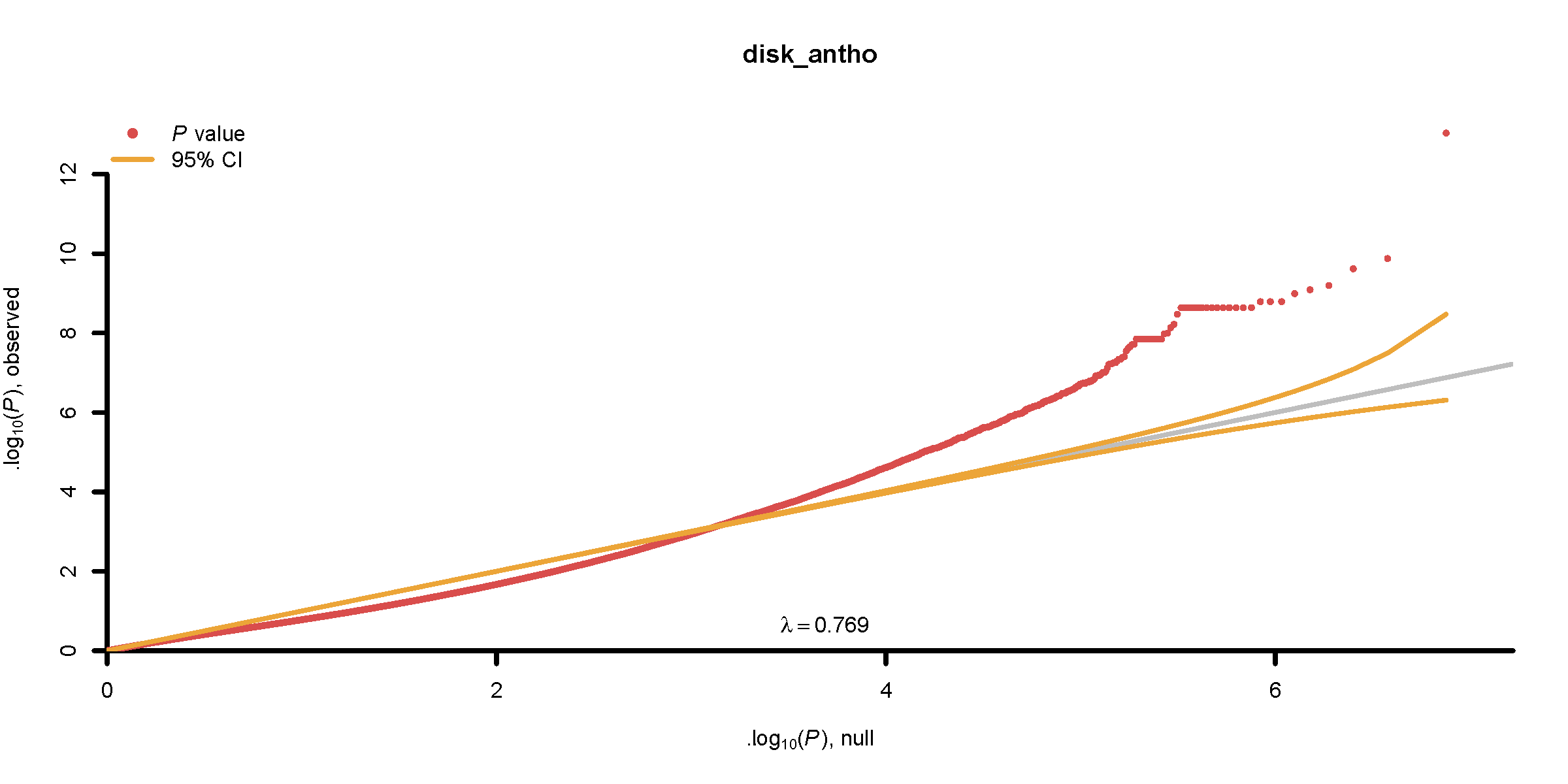

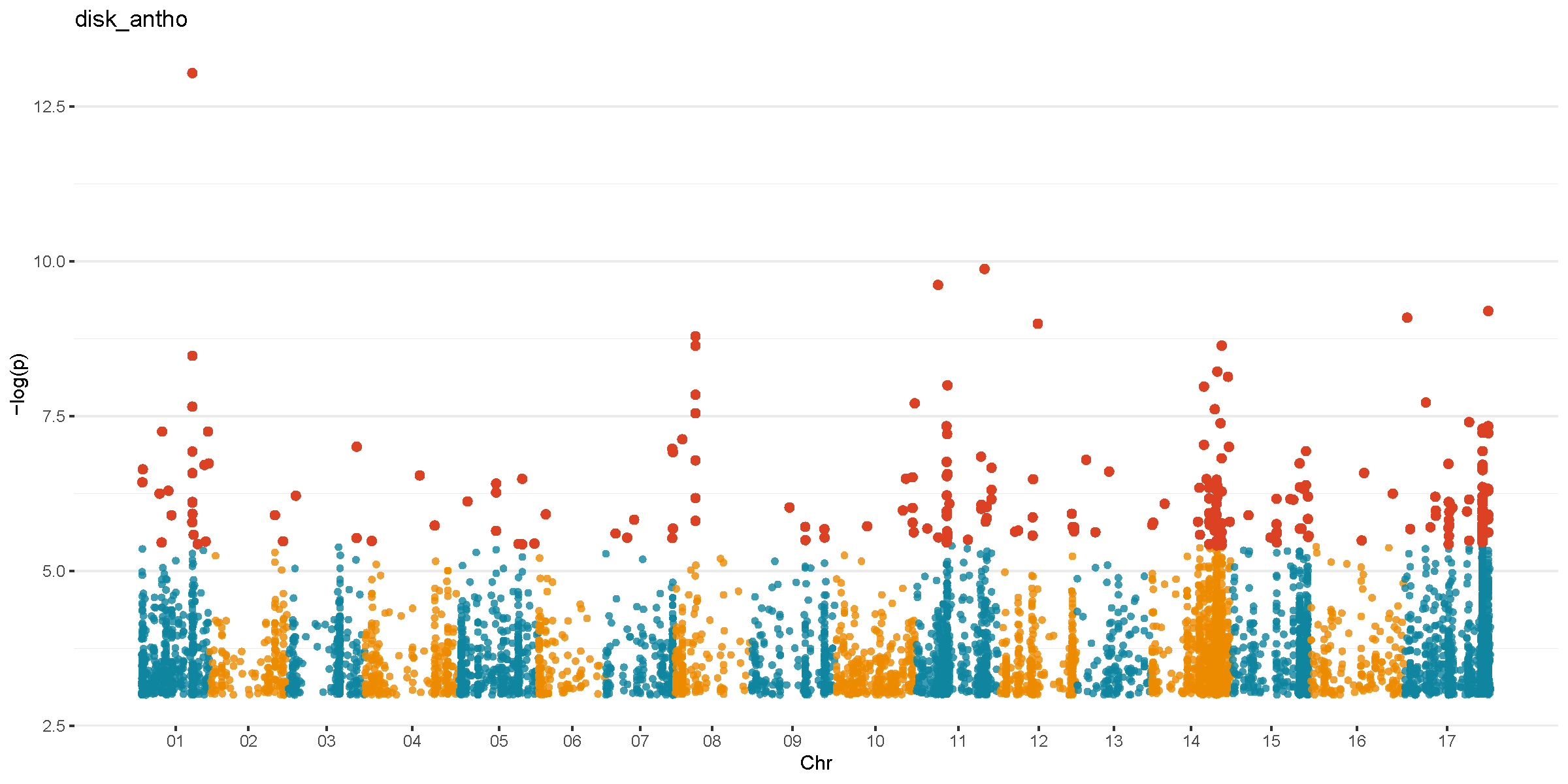

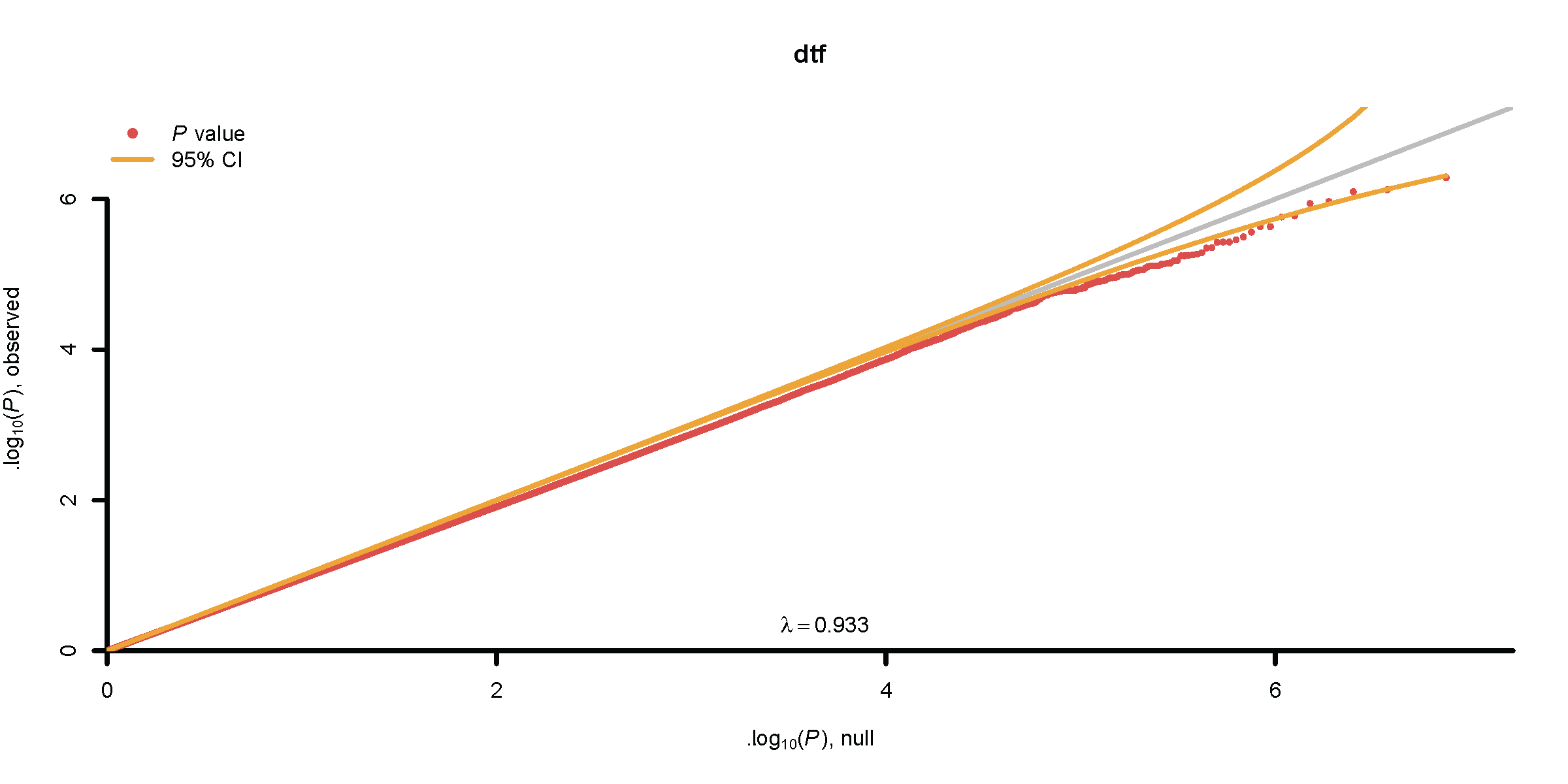

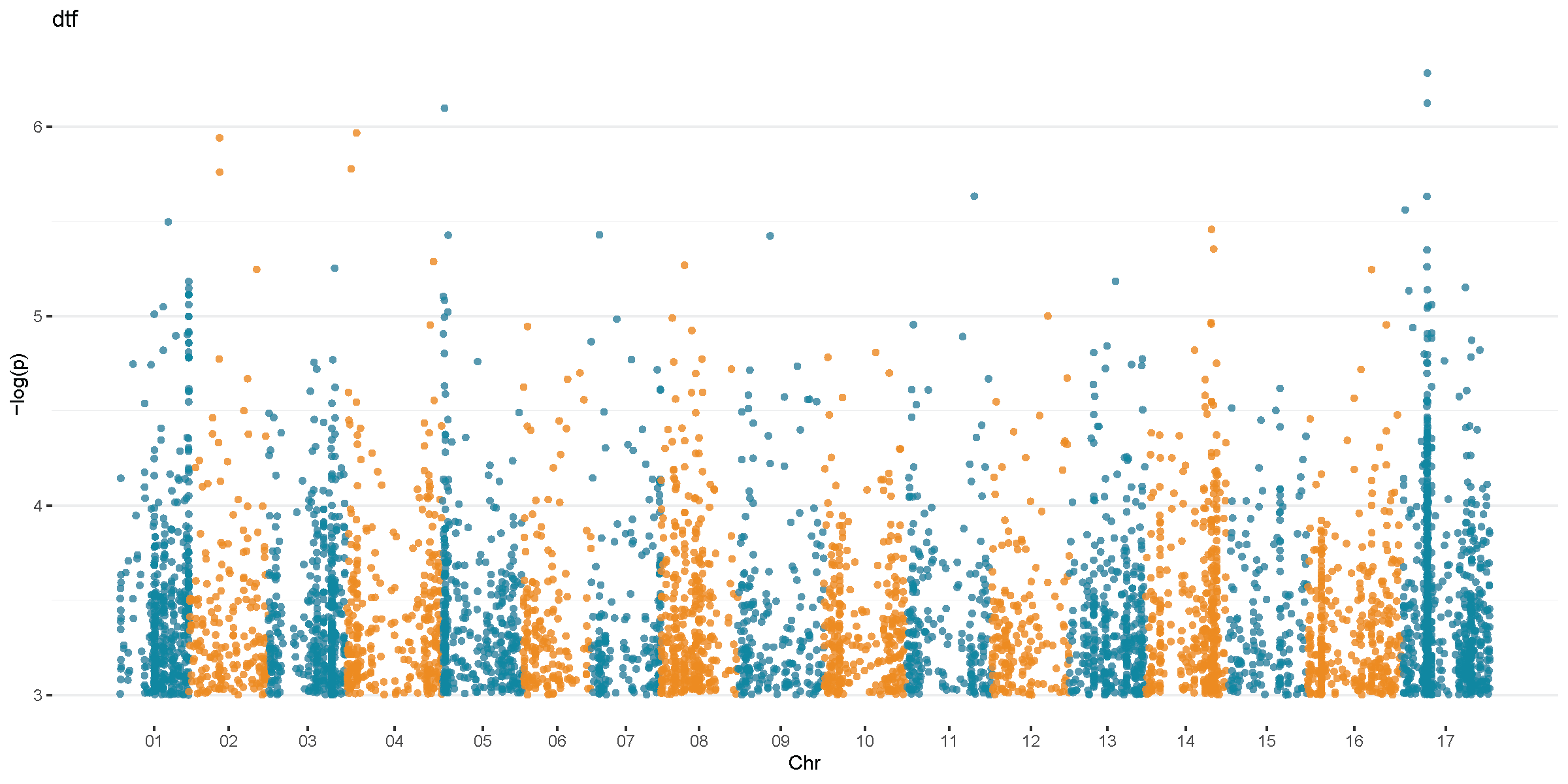

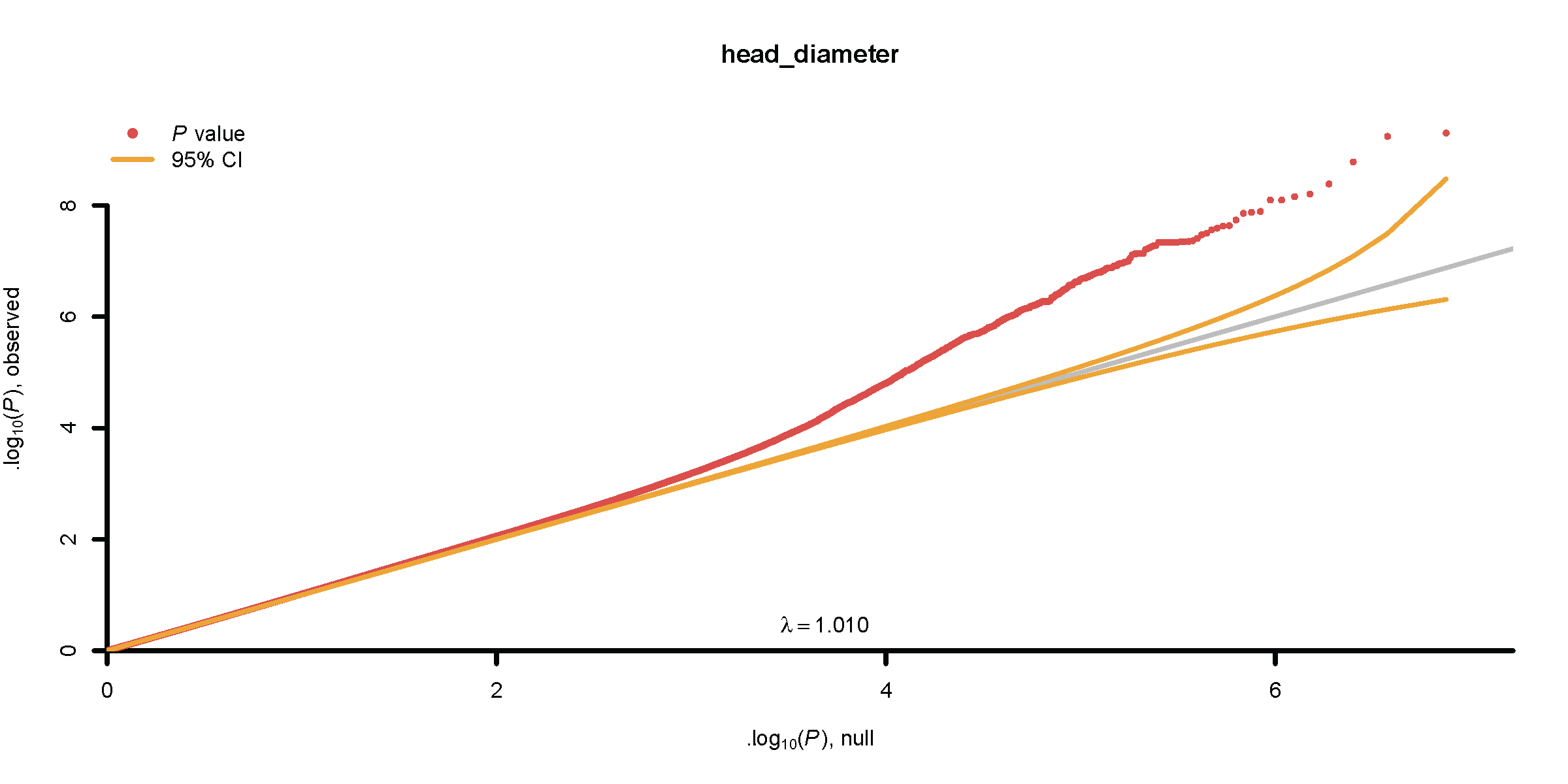

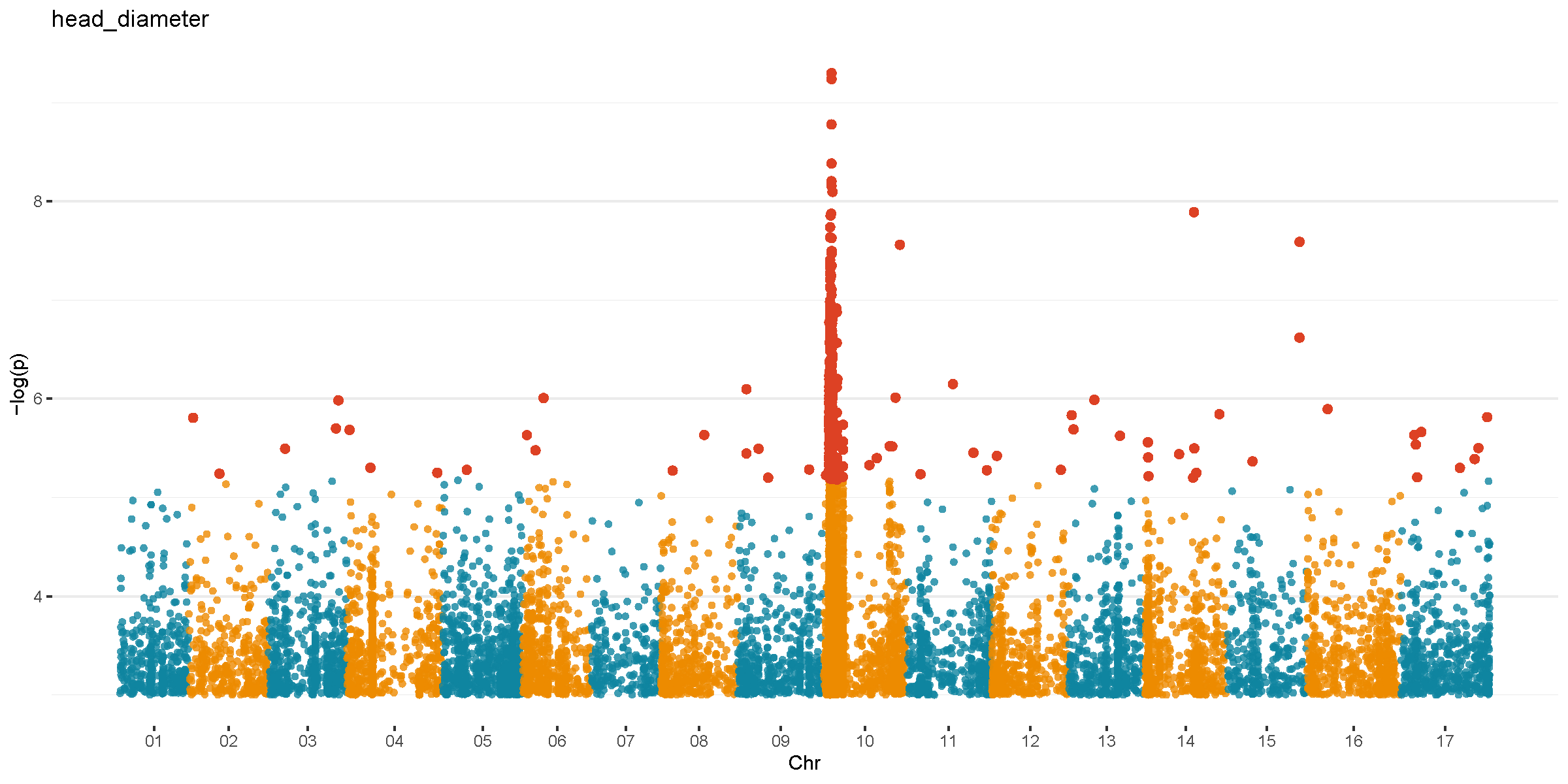

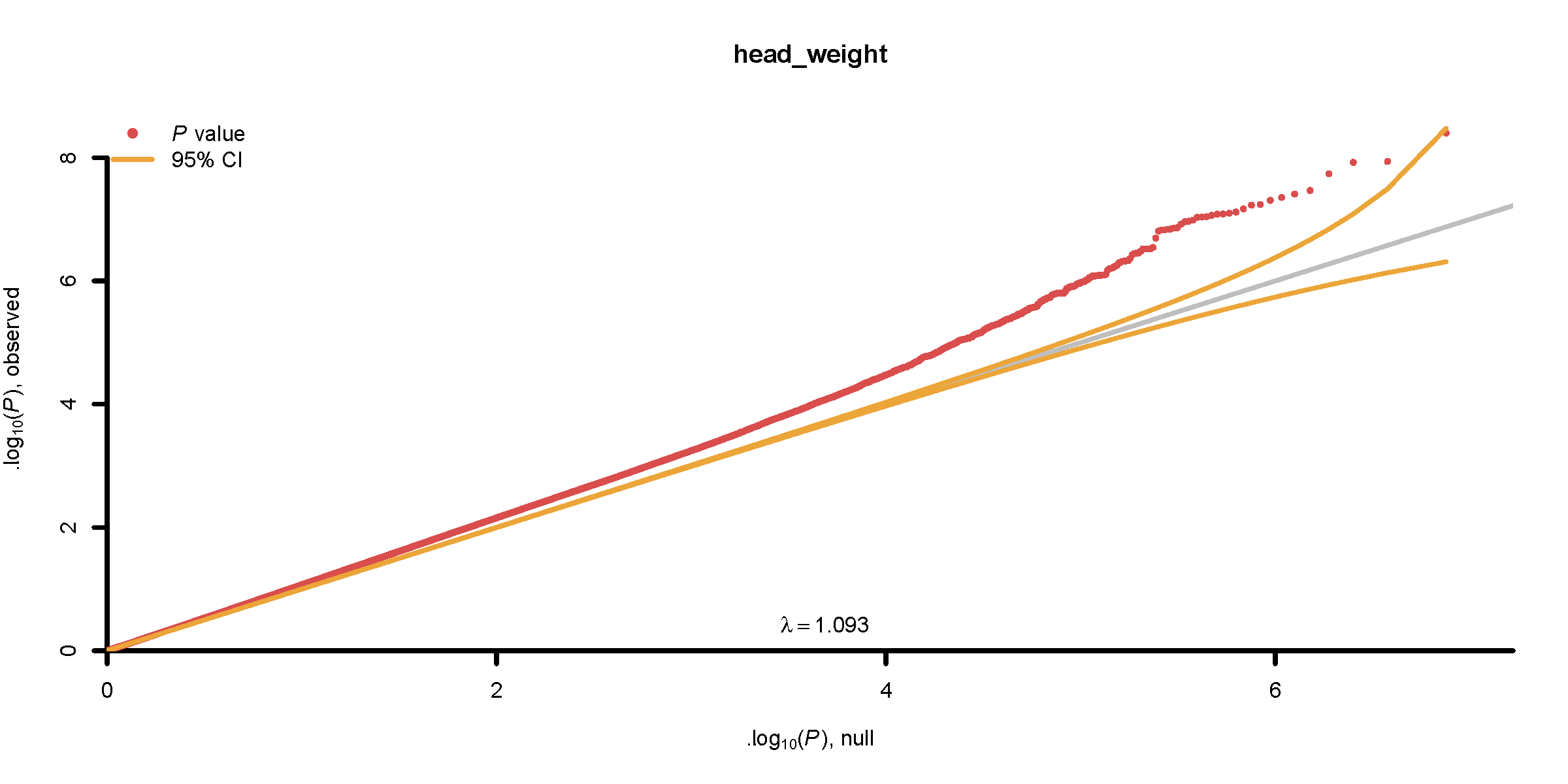

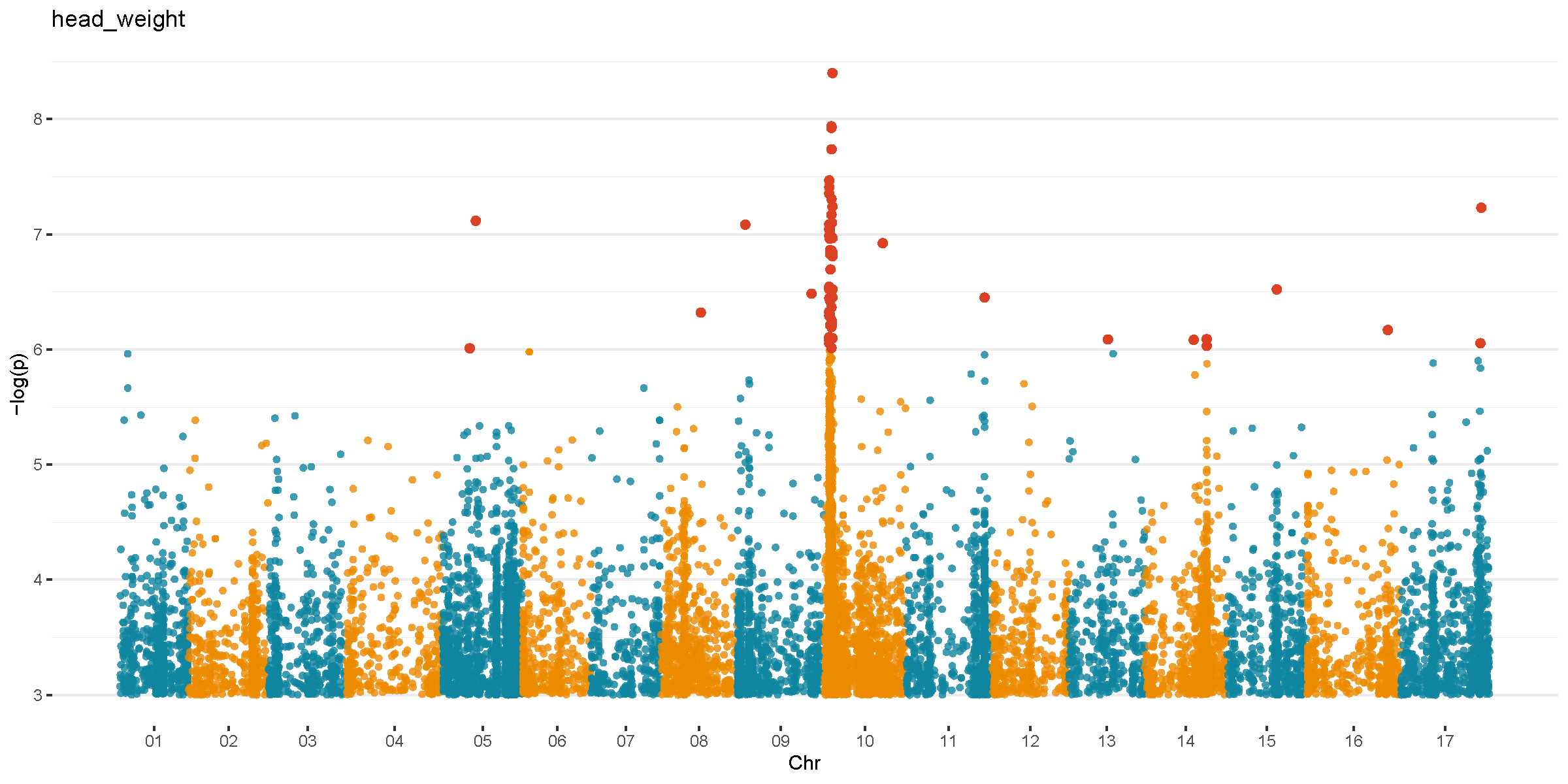

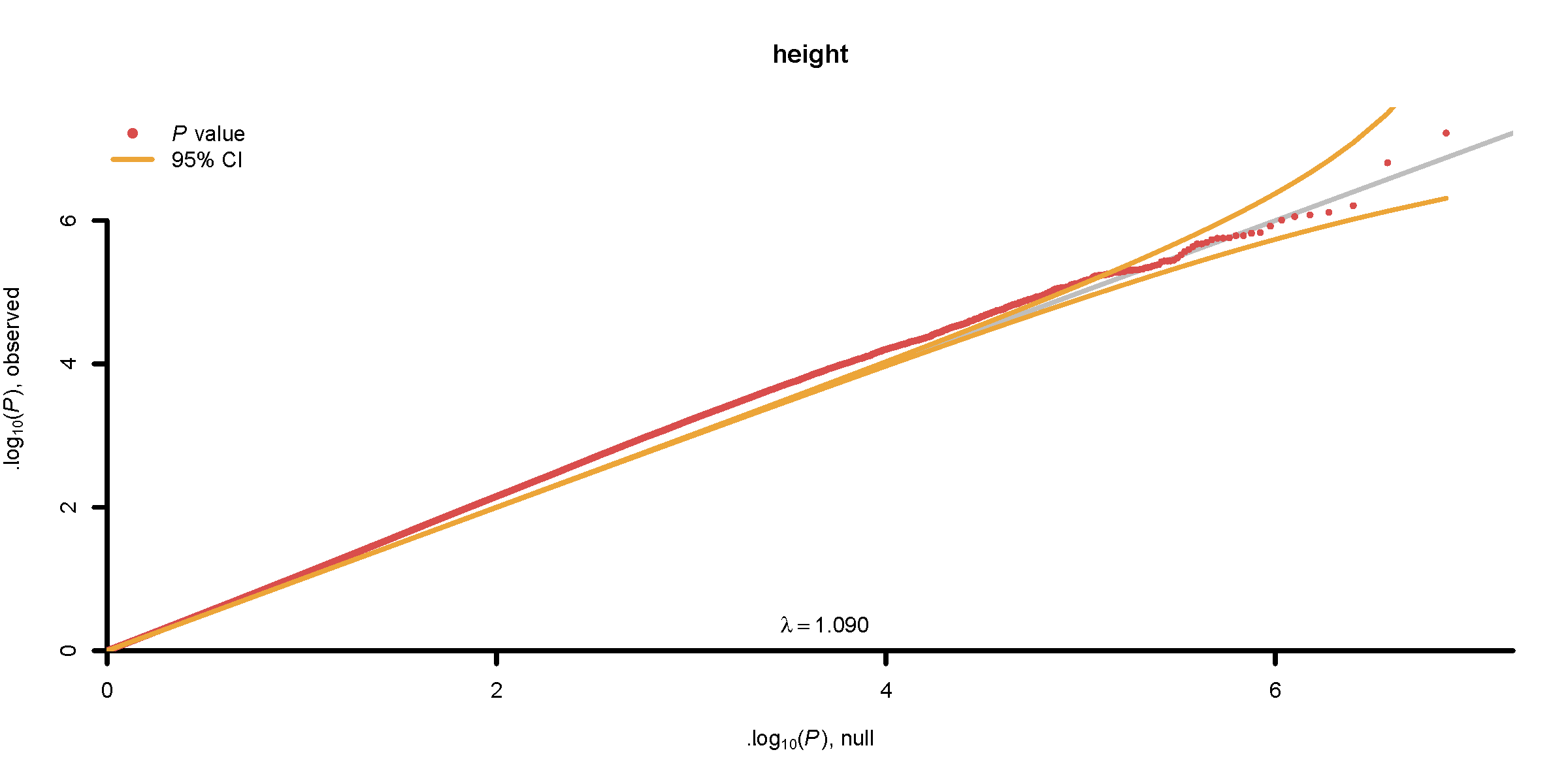

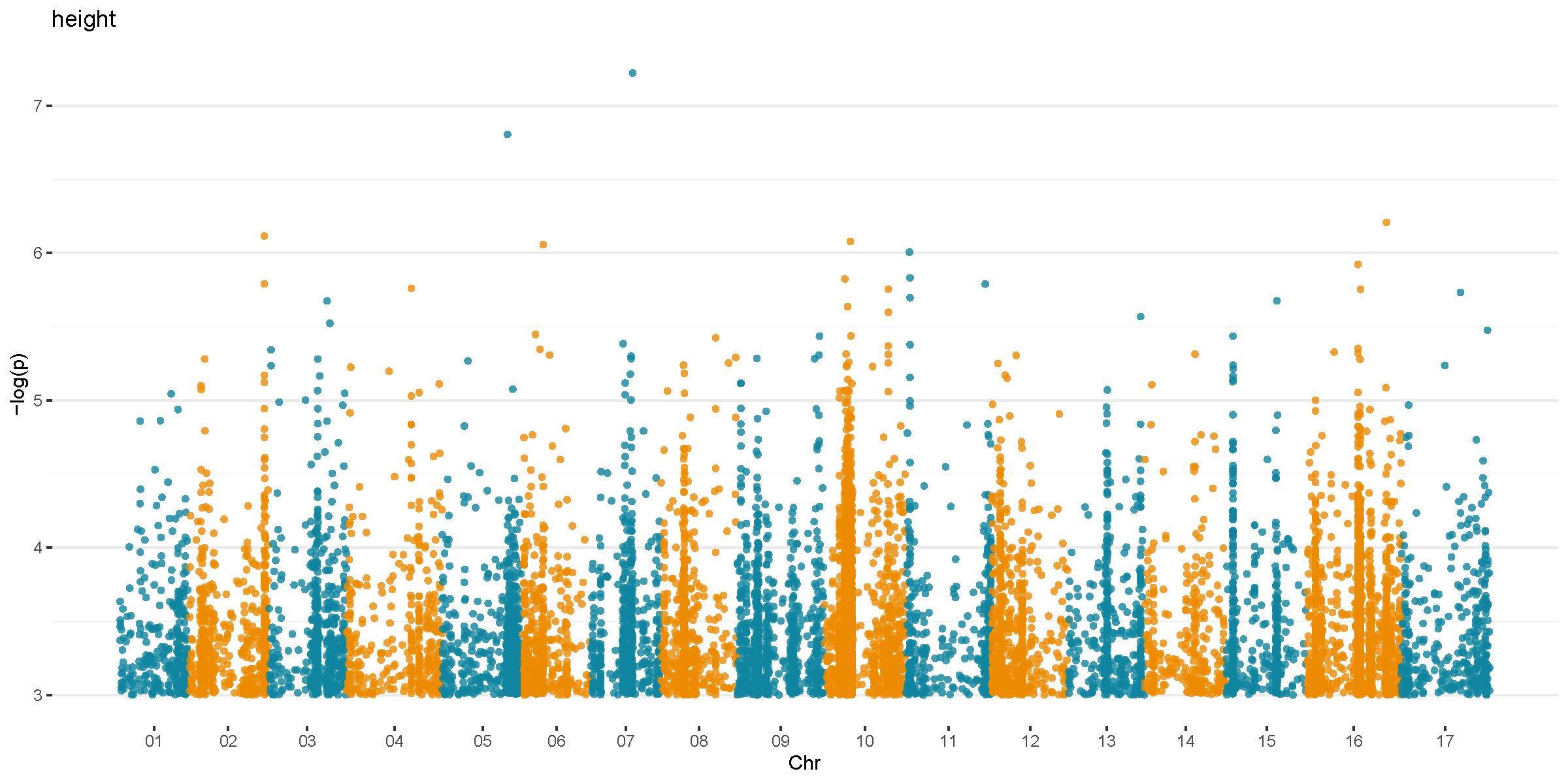

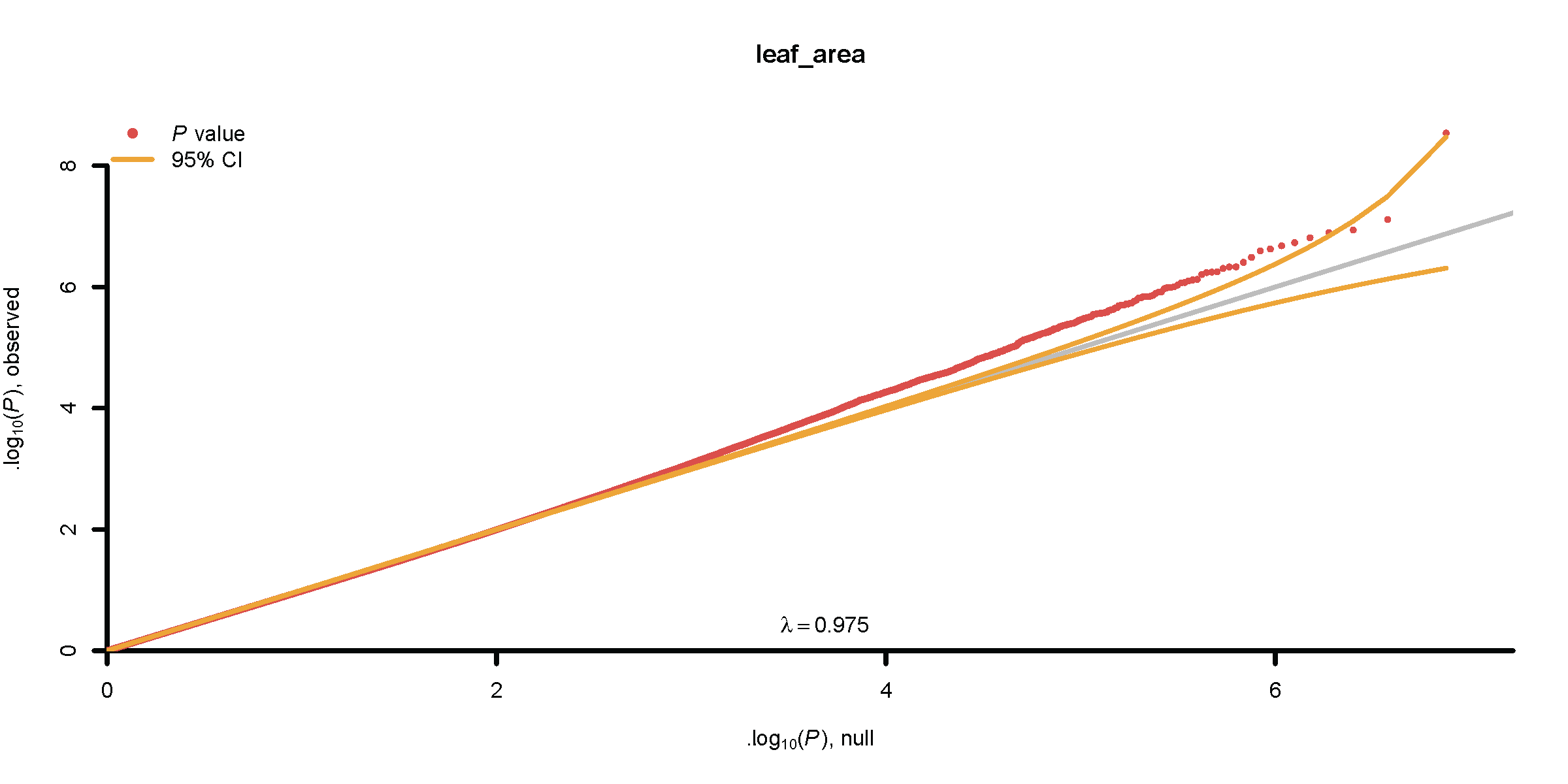

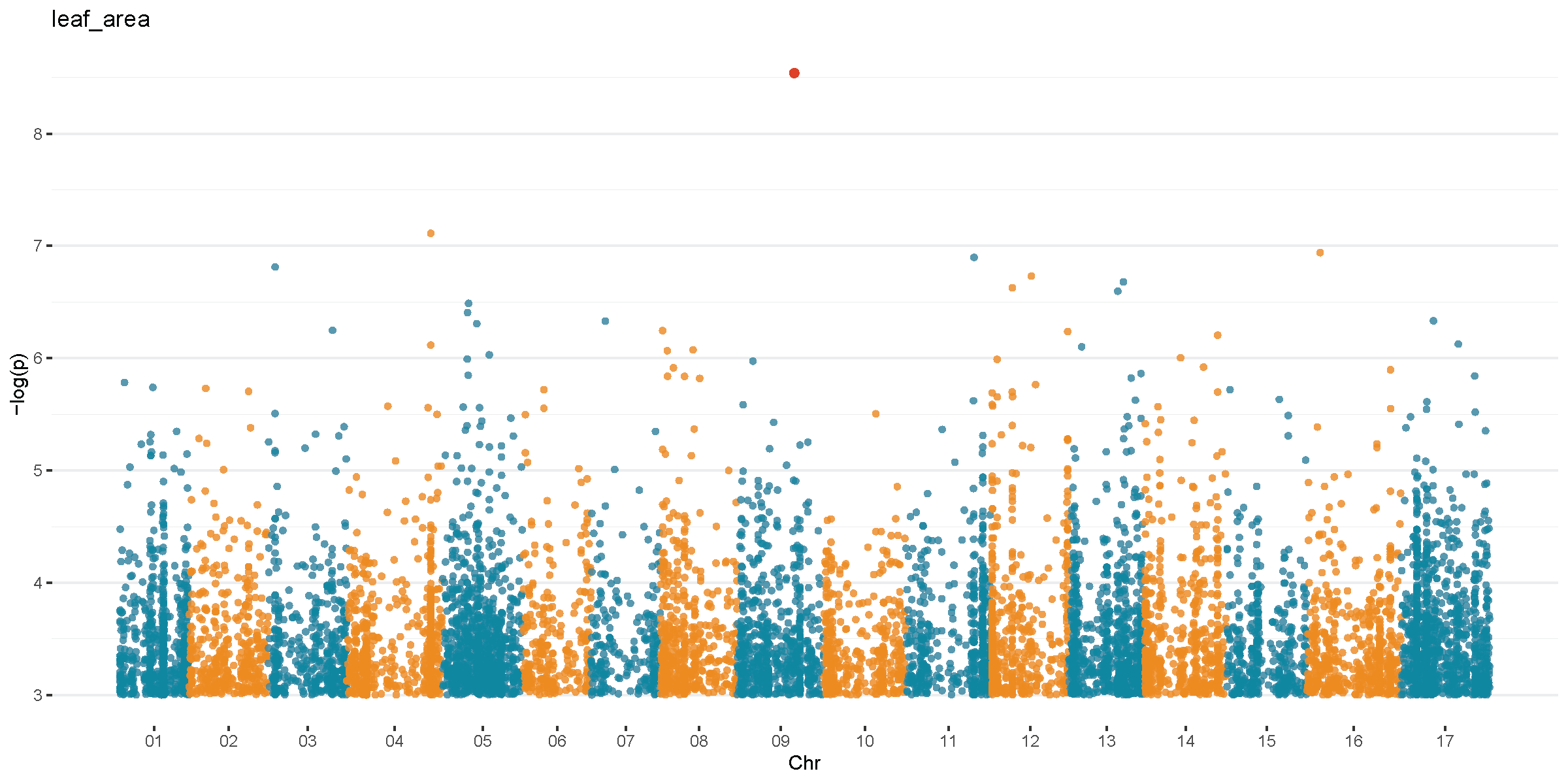

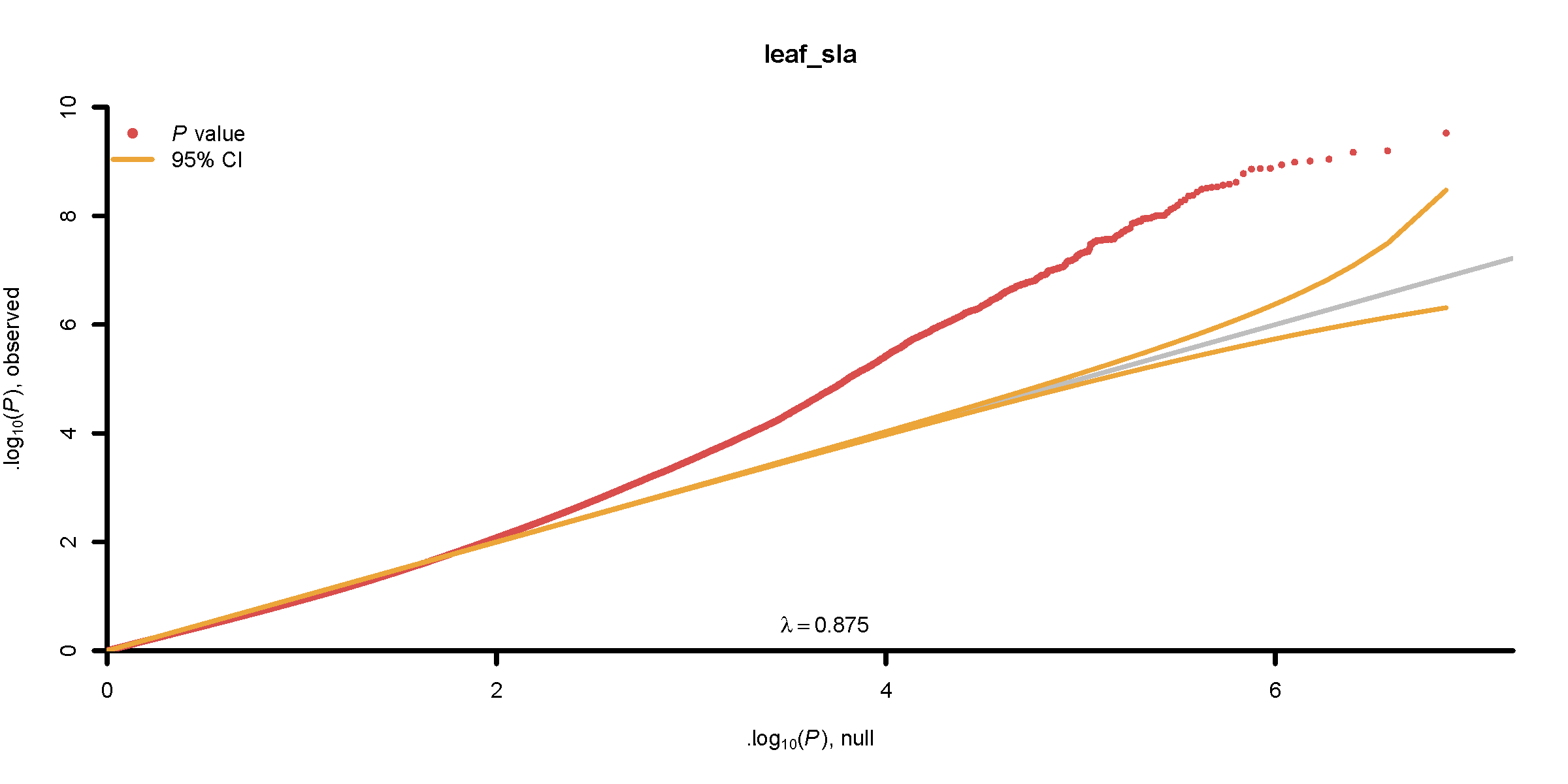

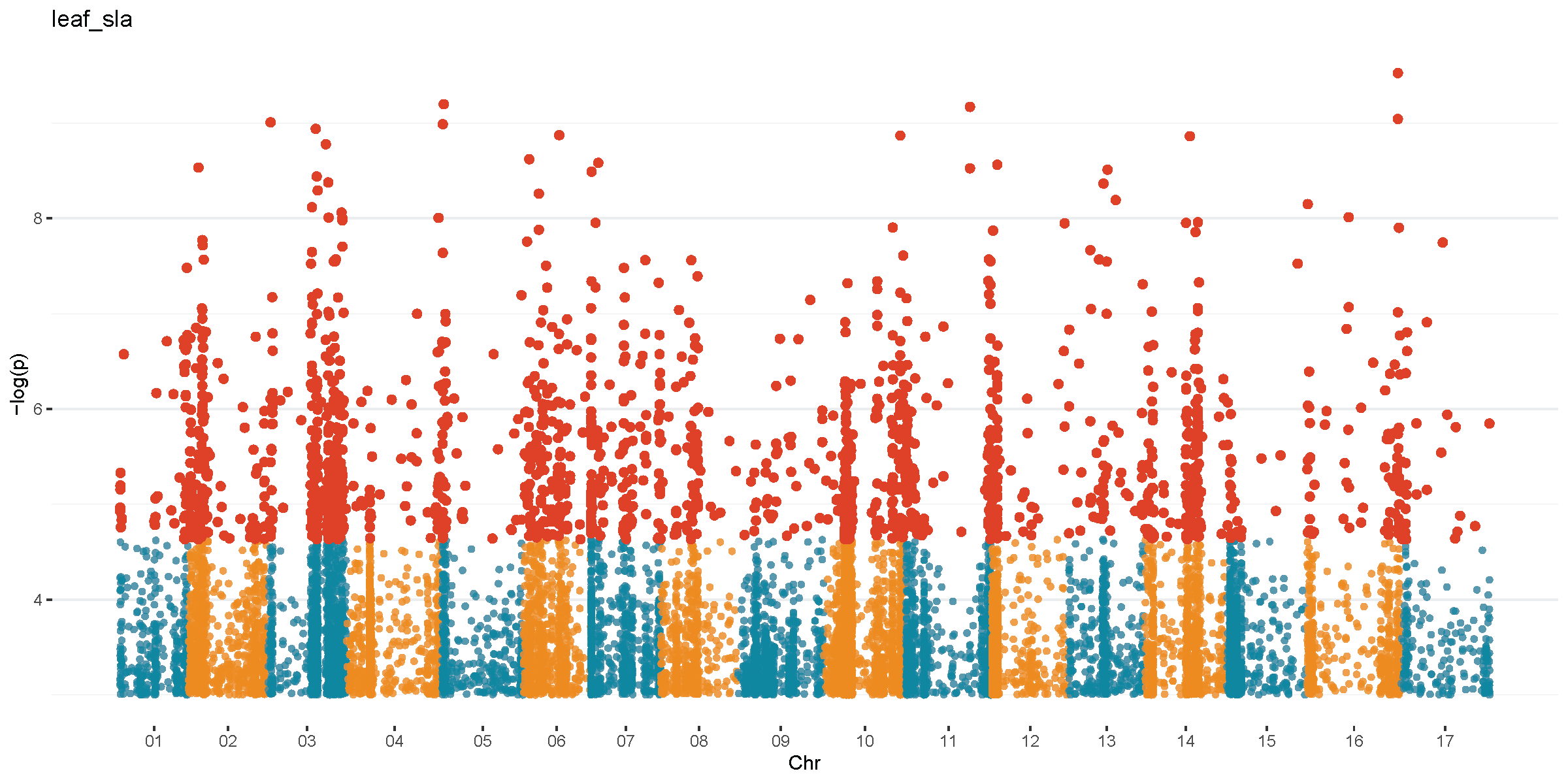

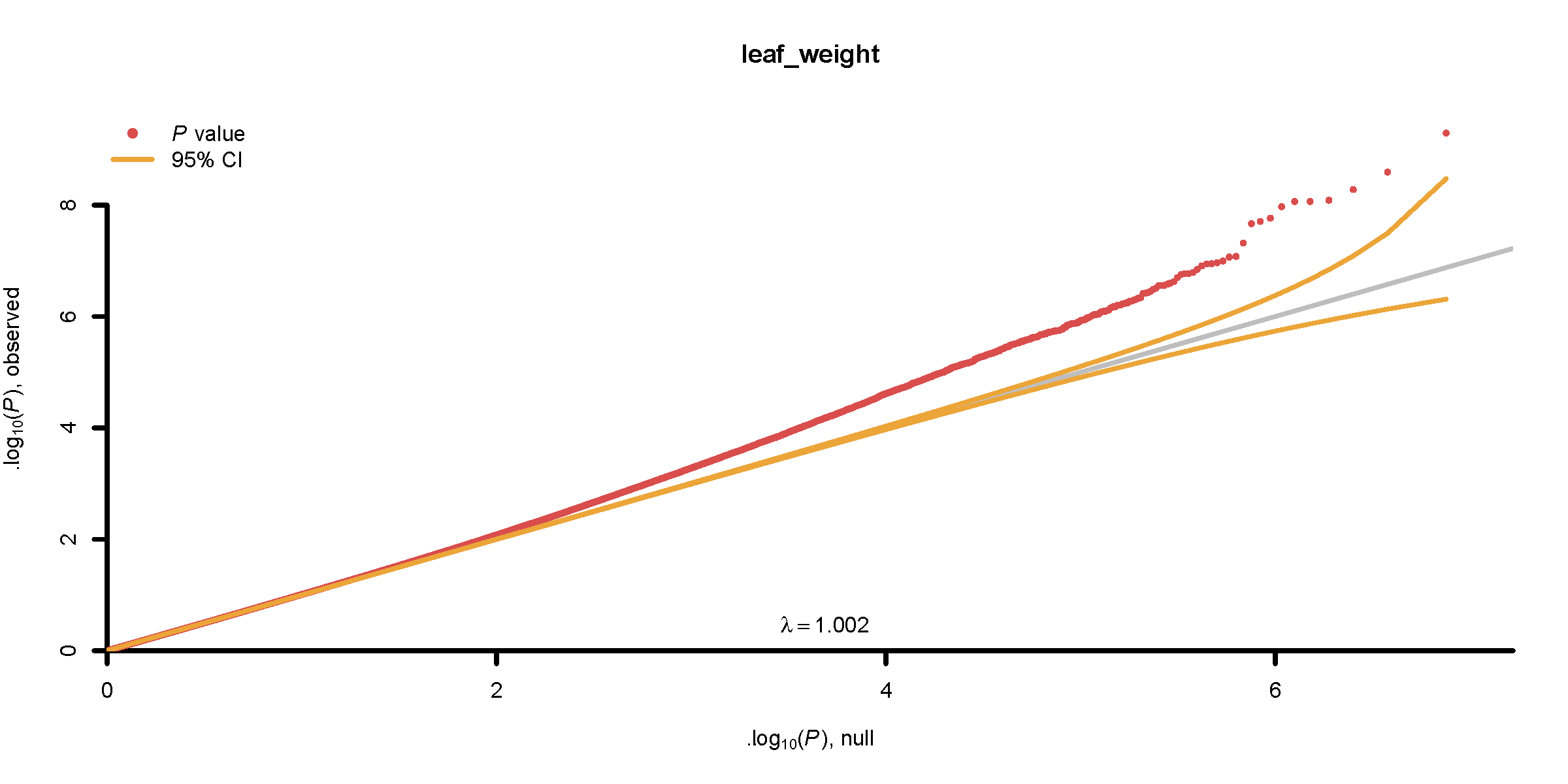

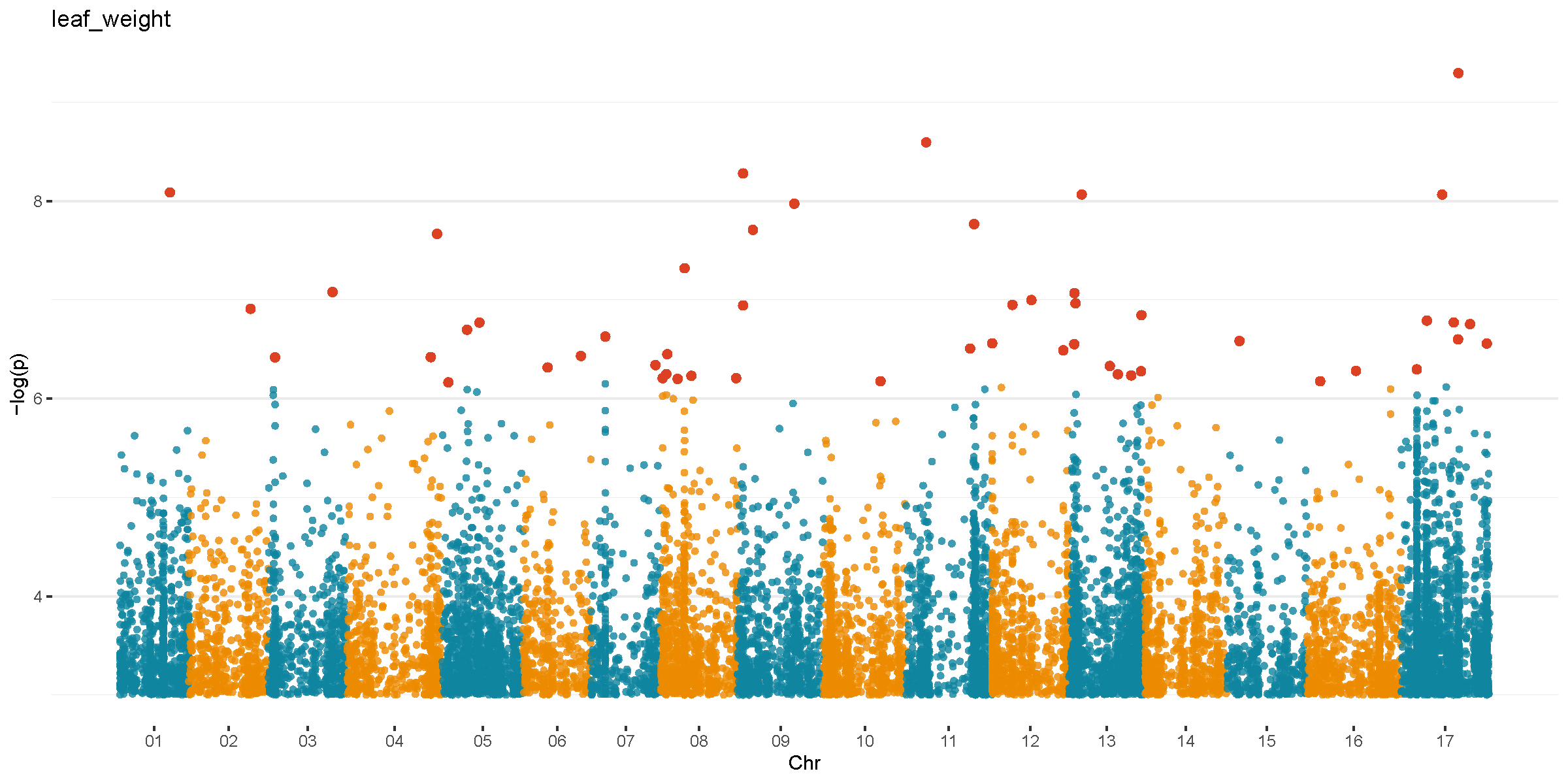

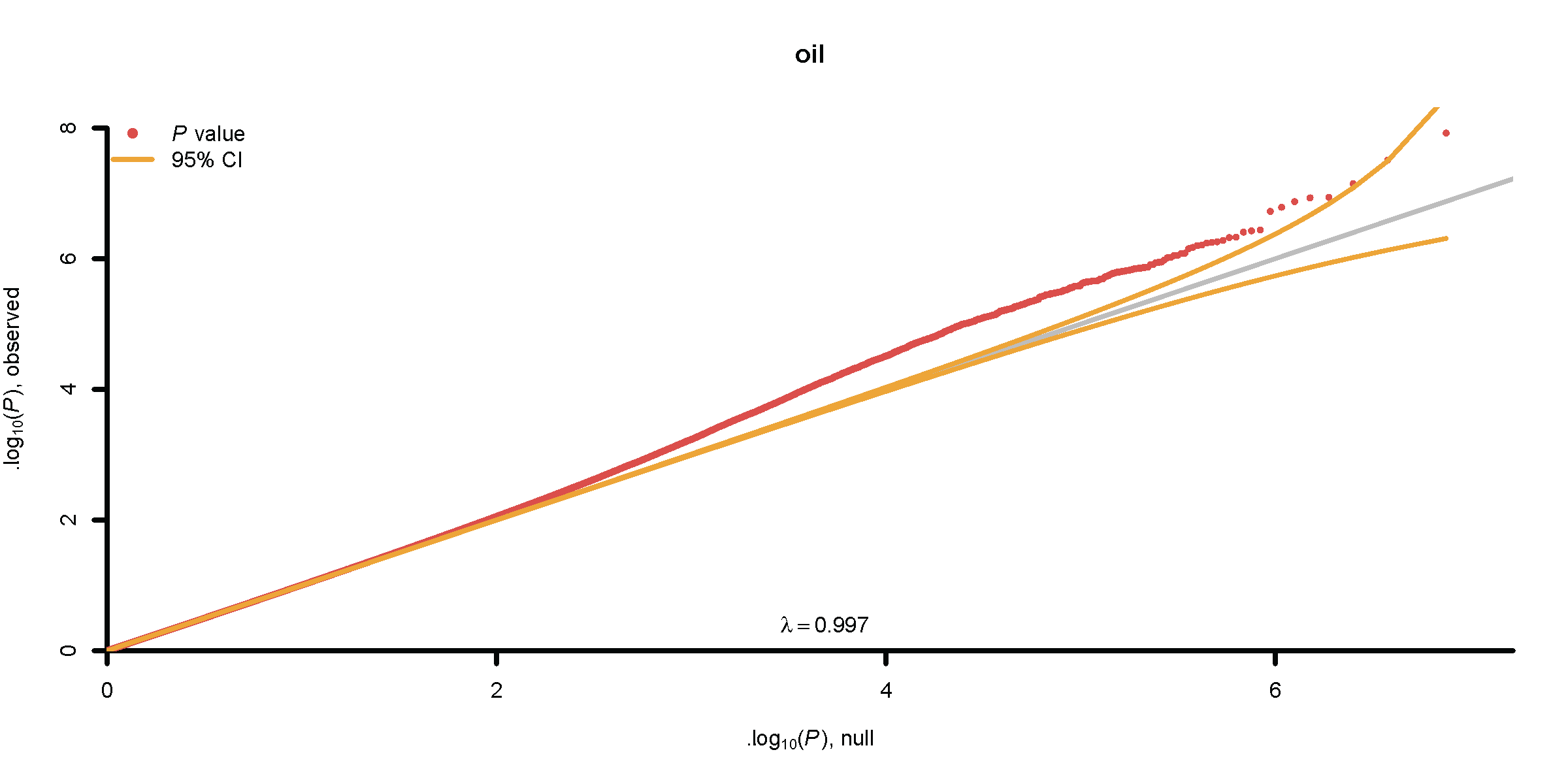

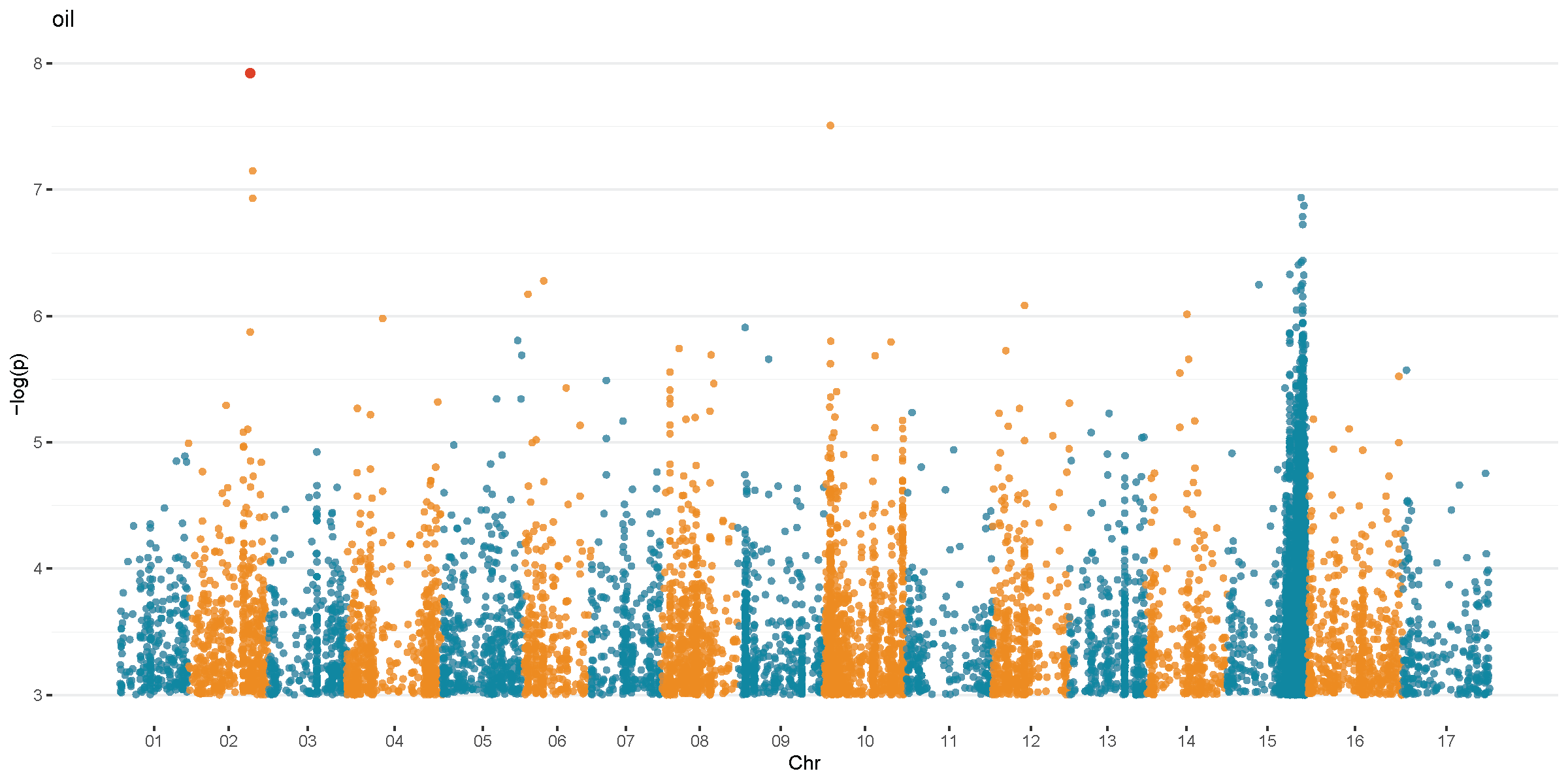

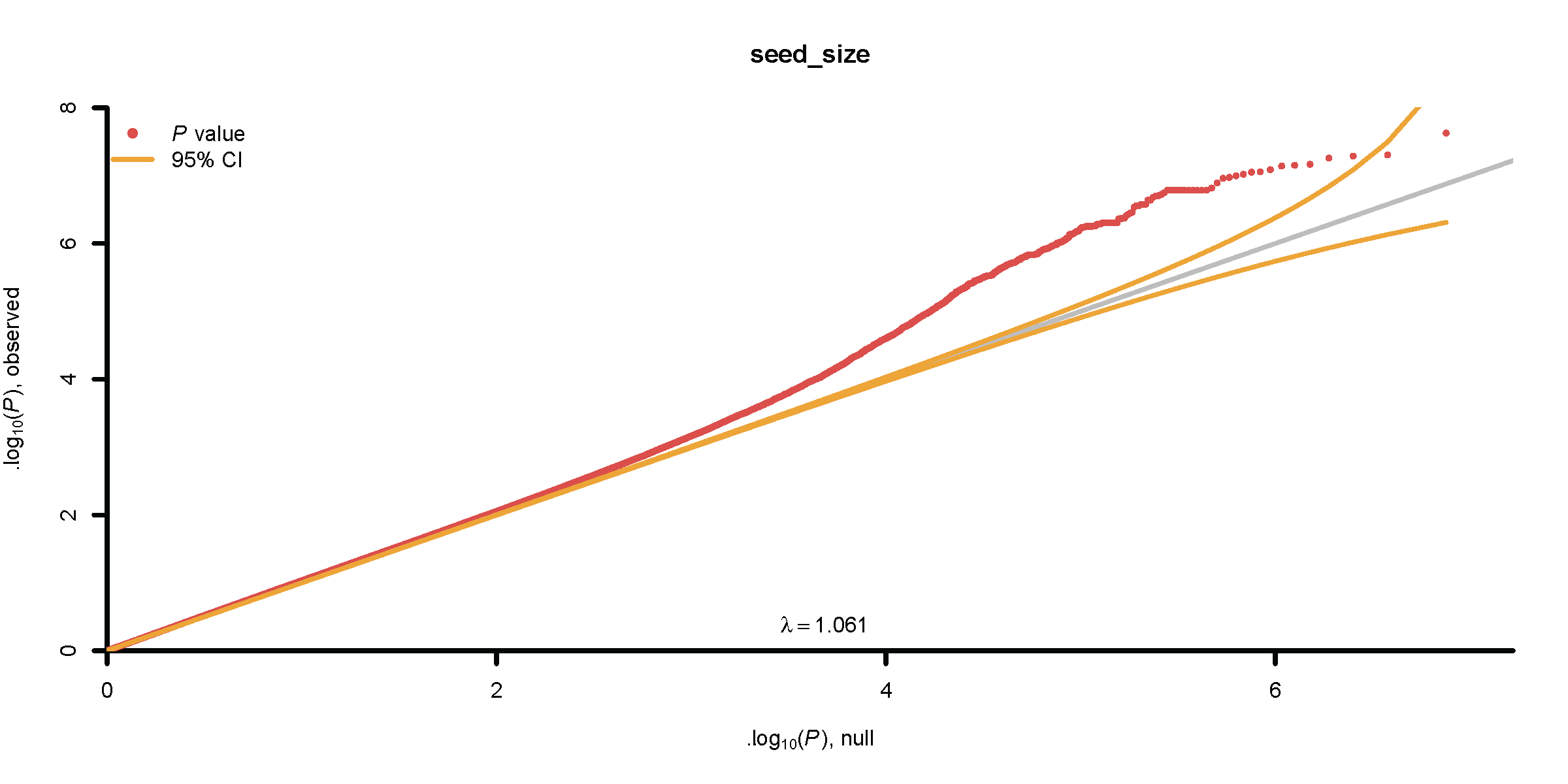

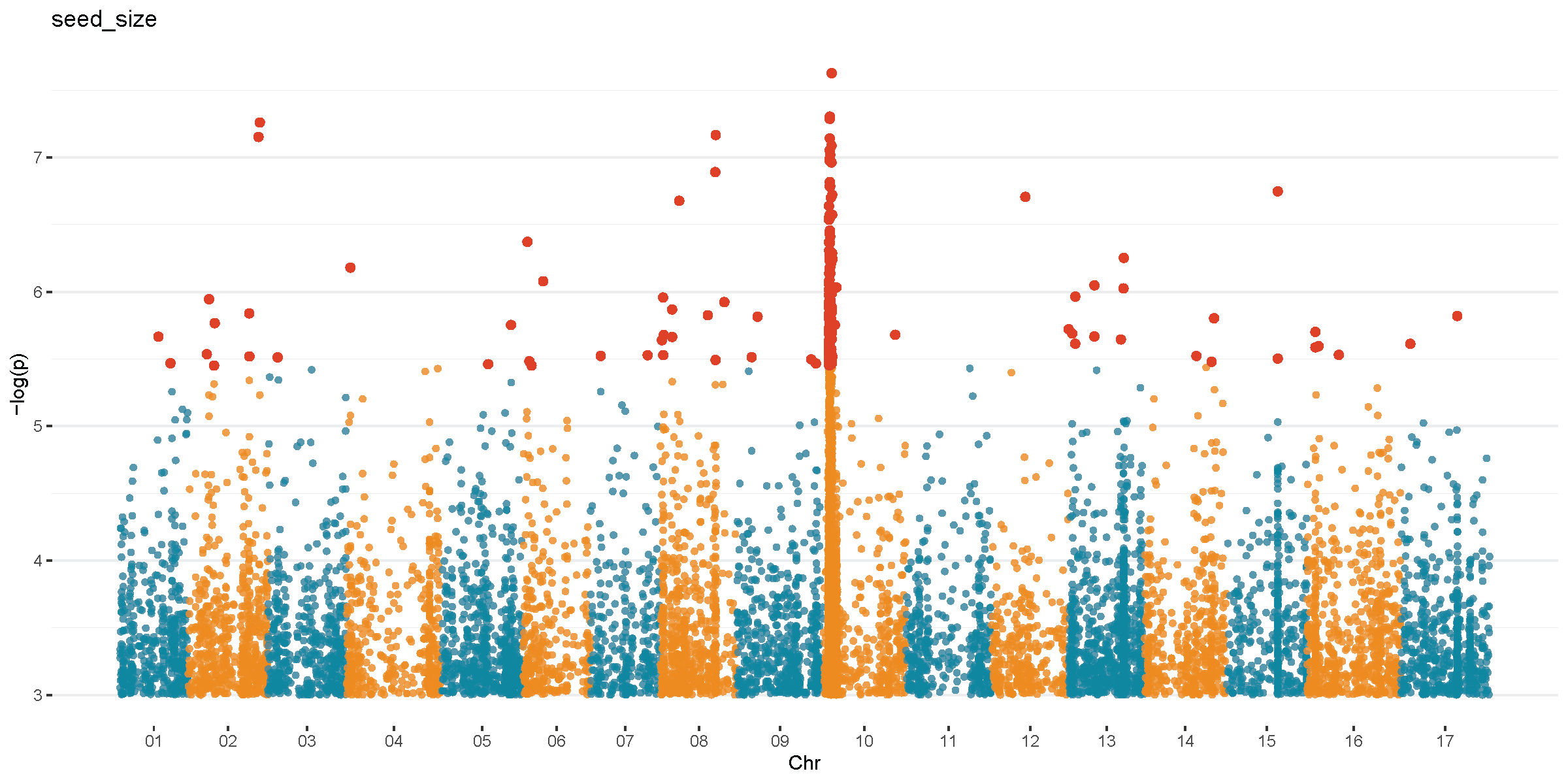

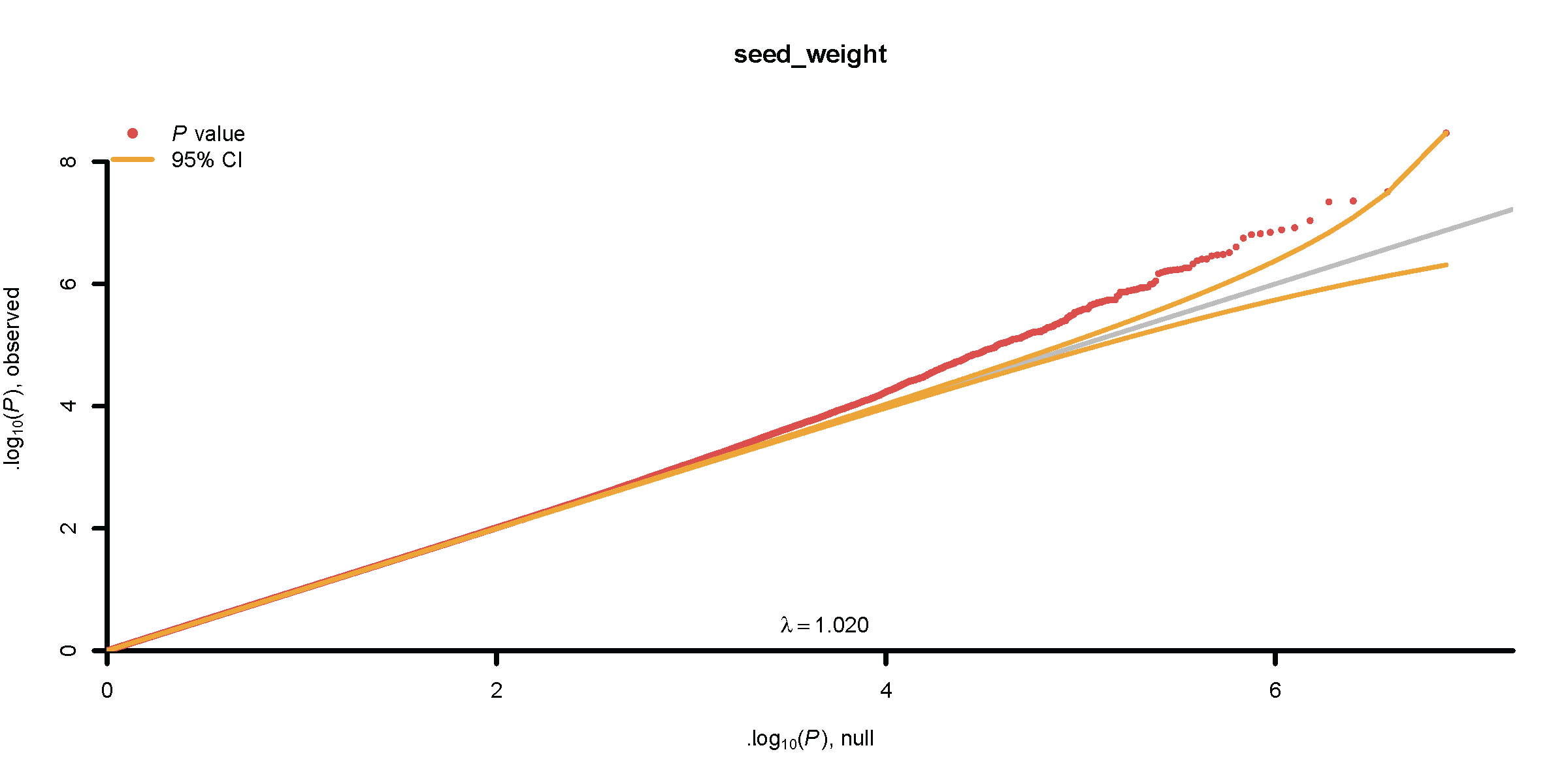

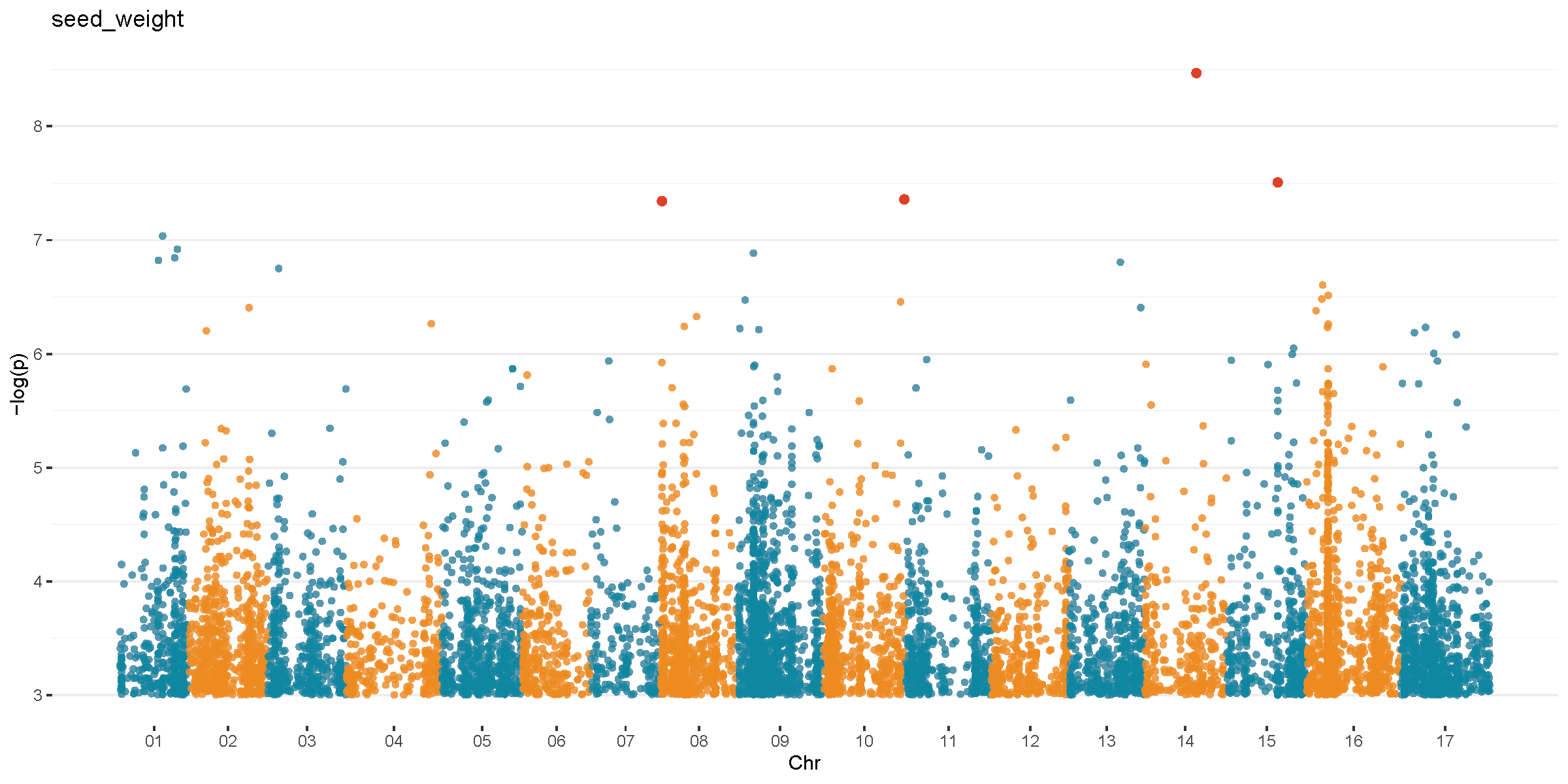

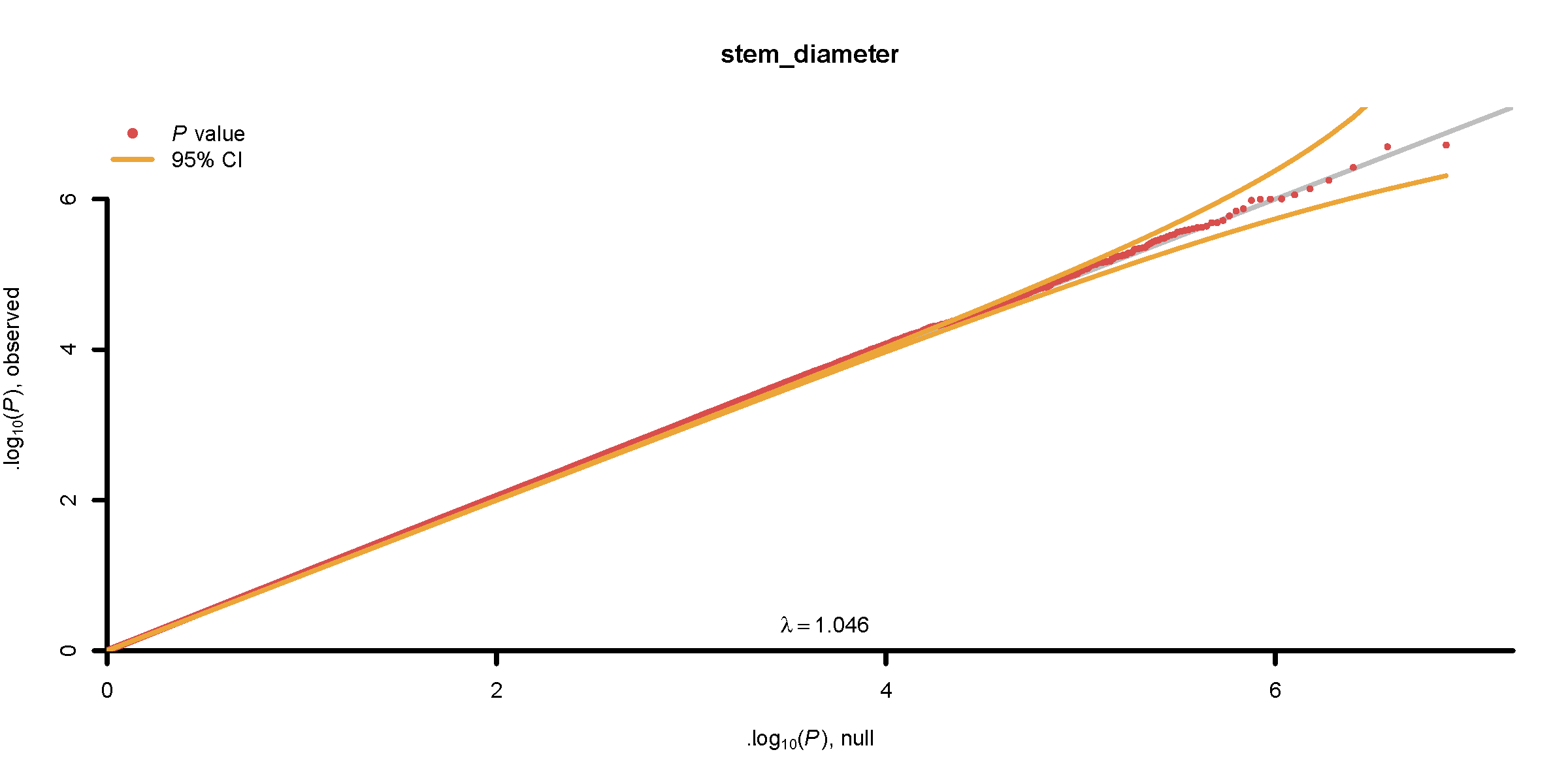

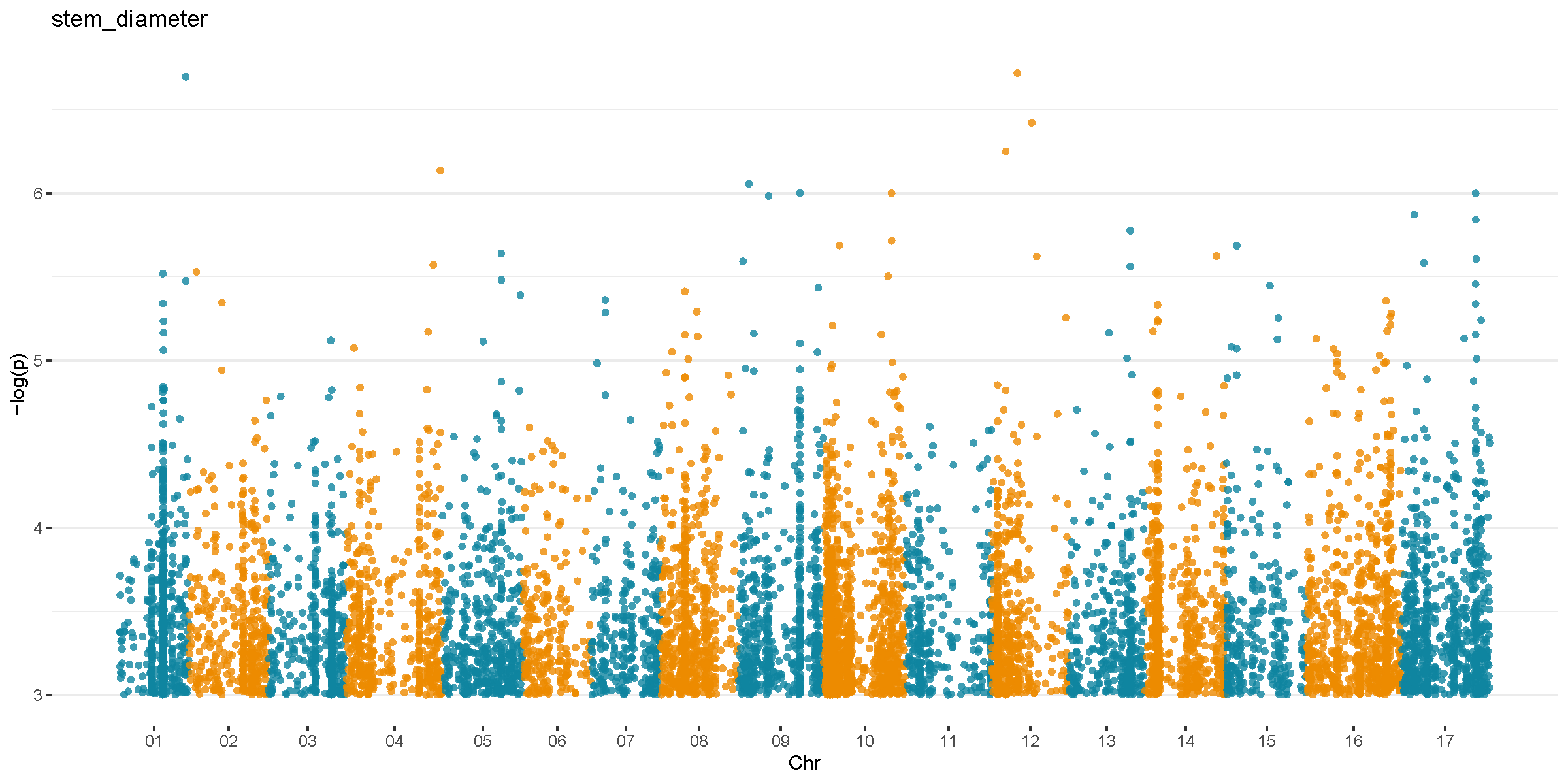

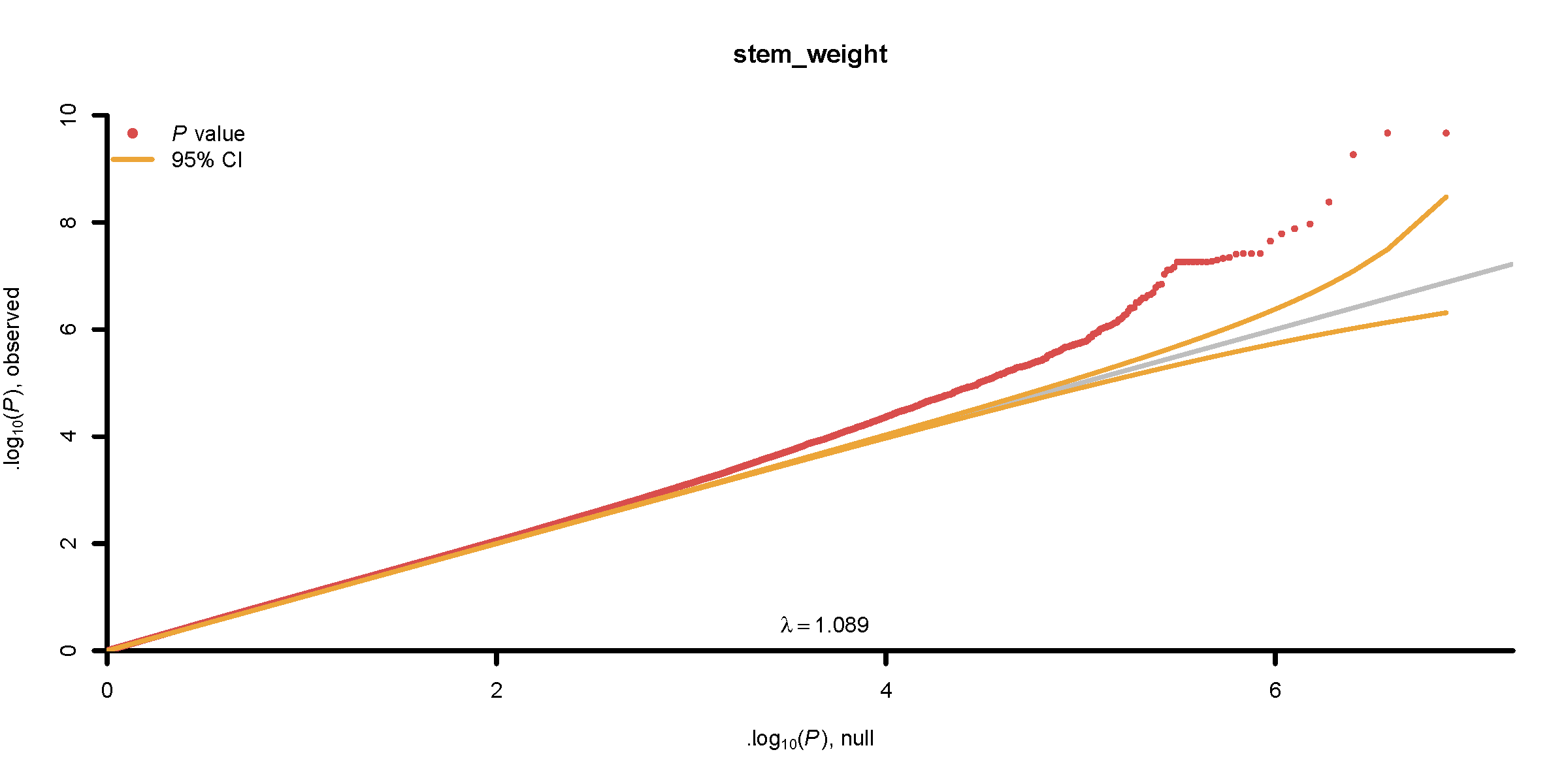

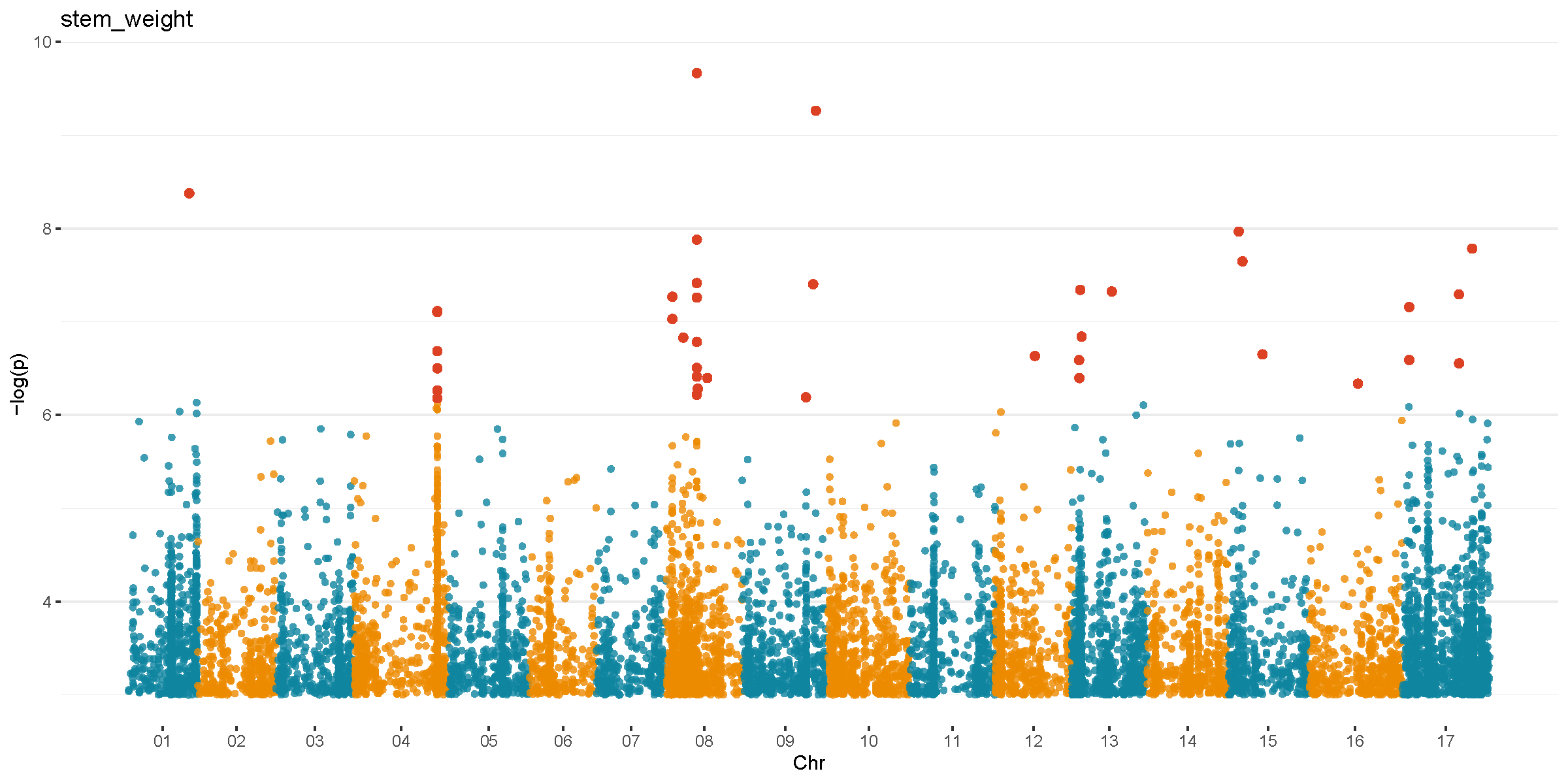

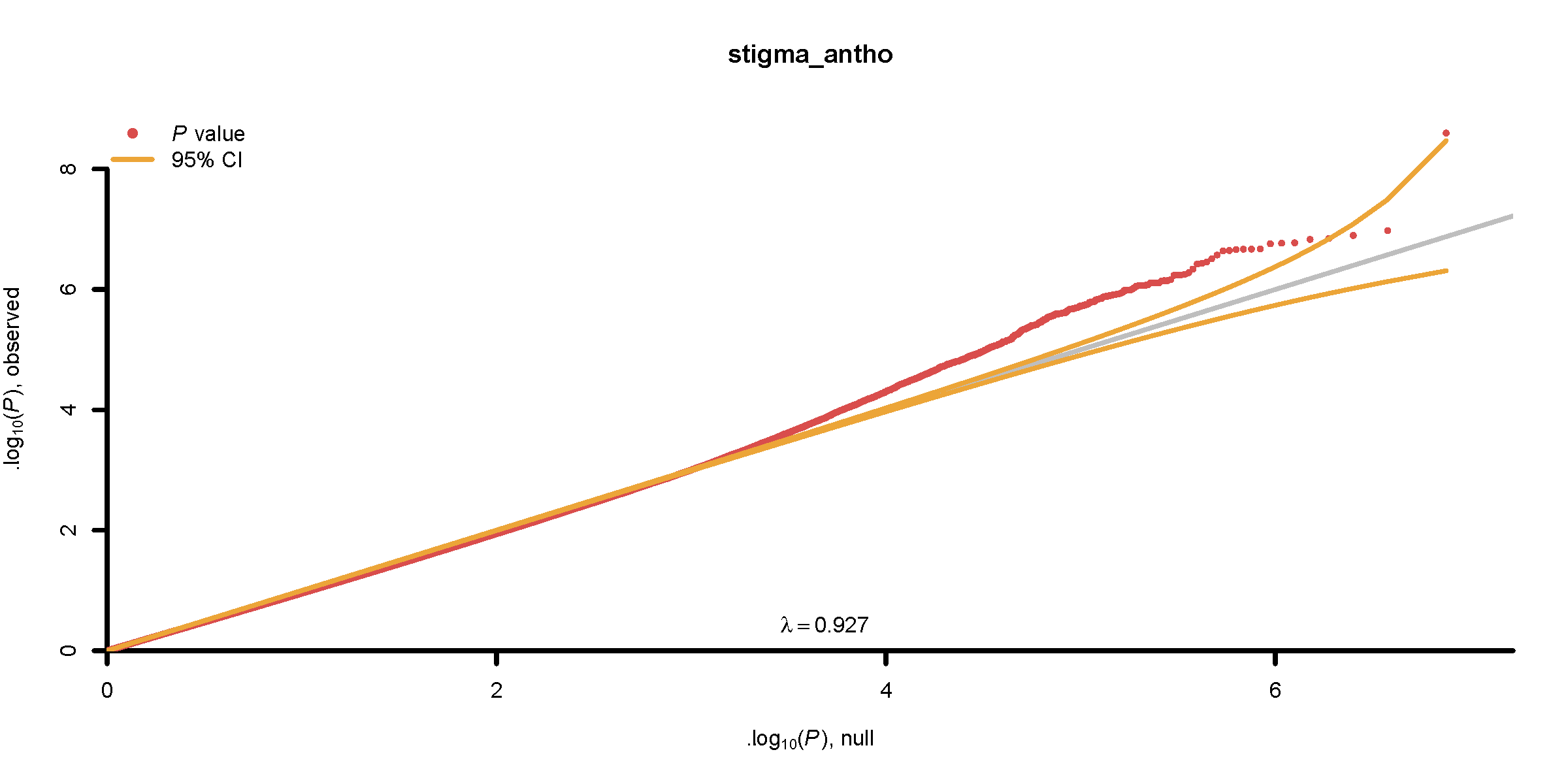

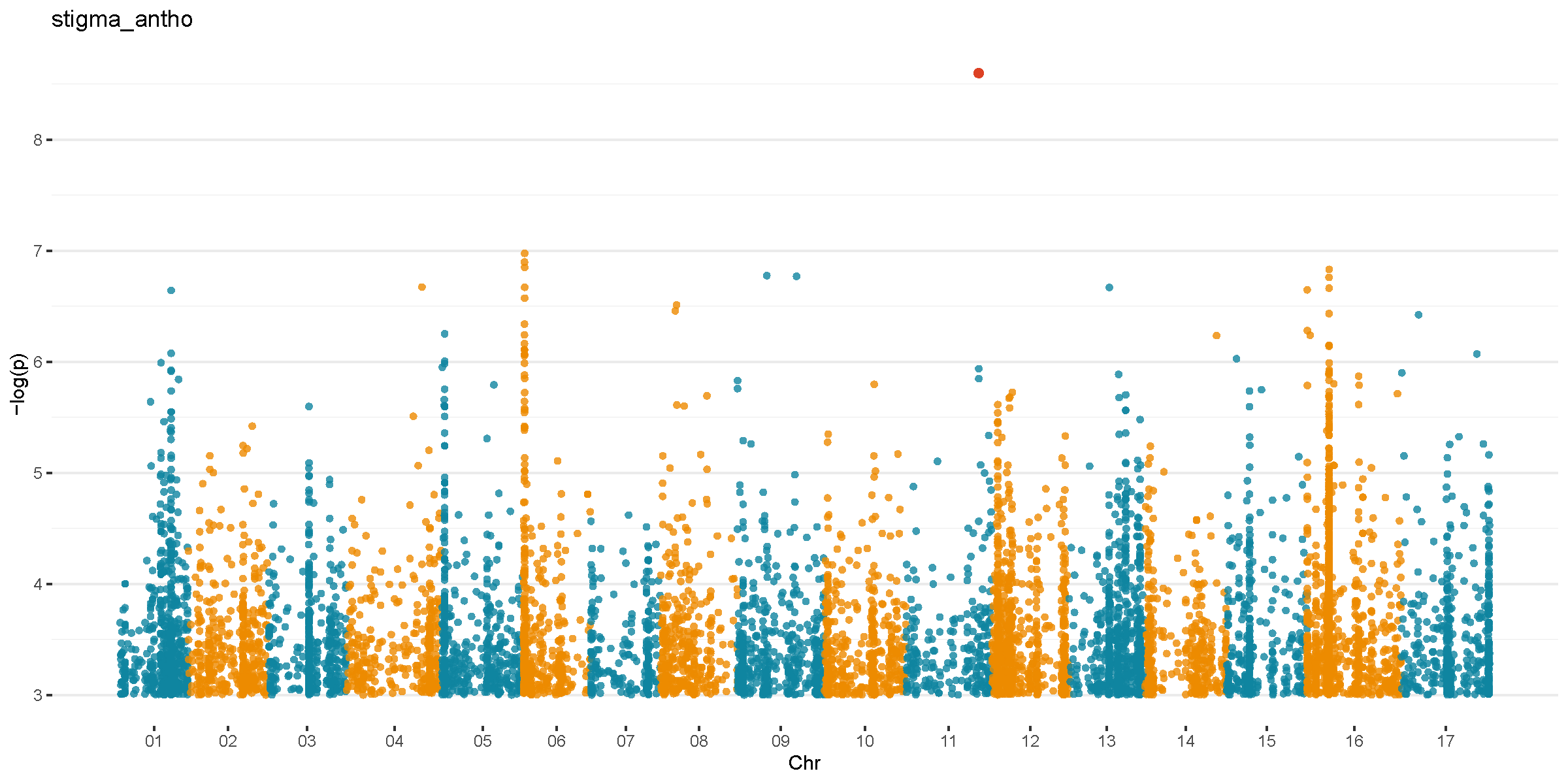


Supplementary Figure S3. Genome-wide association analyses with presence/absence variation. For each trait, panel A represents a quantile-quantile plot of p-values for tested PAV regions using qqPlotFast in ramwas. Values above the diagonal line indicate higher significance than expected based on a normal distribution. Panel B is a manhattan plot of -log10(p-values) for PAV regions. Significance was determined using a q-value correction with a false discovery rate of 0.1. Significant regions are highlighted in red. using a q-value correction with a FDR of 10%. Data collected at two field sites near Ames, IA, USA.

Supplementary Figure S4. Results from linear regression model where X= the percentage of the present allele and Y=predicted phenotype. Fitted linear regression lines for each trait/locality combination.

Supplementary Figure S5. Phenotypic variation (plant height, stem diameter, third-leaf area and total leaf area) in sunflower cultivars under control and drought stress. PSC8: paternal line, XRQ: maternal line, INEDI: F1 hybrid of PSC8 and XRQ. C: control, D: drought. Phenotypic heterosis was found in INEDI particularly in plant height category (asterisk indicates P < 0.05, Wilcoxon rank-sum test in comparison between INEDI and the parental lines).

Supplementary Figure S6. Partial least squares (sPLS) individual plots with confidence ellipse.

Supplementary Figure S7. Trait values for heterotic traits from three common garden locations. The orange bar shows the trait value for HA412HO, the source of the reference genome.

Supplementary Table S1. Genes exhibiting presence absence variation between the parental lines (XRQ and PSC8) and expression complementation in their F_1_ hybrid (INEDI).

1. control_noPSC8_on_XRQ

| **gene ID** | ***Arabidopsis thaliana* gene ID** |
| --- | --- |
| HanXRQChr16g0749691 | AT1G01820.1 |
| HanXRQChr10g0436251 | AT1G04420.1 |
| HanXRQChr03g0126211 | AT1G50140.1 |
| HanXRQChr04g0141691 | AT1G50140.1 |
| HanXRQChr11g0480621 | AT1G66970.1 |
| HanXRQChr10g0449201 | AT2G25180.1 |
| HanXRQChr13g0617081 | AT3G14470.1 |
| HanXRQChr13g0617161 | AT3G14470.1 |
| HanXRQChr16g0740771 | AT3G14790.1 |
| HanXRQChr02g0052871 | AT3G48320.1 |
| HanXRQChr01g0042601 | AT4G16260.1 |
| HanXRQChr03g0121461 | AT4G23160.1 |
| HanXRQChr01g0003811 | NA |
| HanXRQChr01g0014641 | NA |
| HanXRQChr01g0014651 | NA |
| HanXRQChr02g0064201 | NA |
| HanXRQChr04g0141701 | NA |
| HanXRQChr04g0158081 | NA |
| HanXRQChr04g0158091 | NA |
| HanXRQChr04g0179591 | NA |
| HanXRQChr04g0187531 | NA |
| HanXRQChr05g0199651 | NA |
| HanXRQChr05g0229031 | NA |
| HanXRQChr06g0251111 | NA |
| HanXRQChr06g0260701 | NA |
| HanXRQChr06g0262961 | NA |
| HanXRQChr07g0317001 | NA |
| HanXRQChr08g0323981 | NA |
| HanXRQChr08g0327641 | NA |
| HanXRQChr09g0383871 | NA |
| HanXRQChr09g0407951 | NA |
| HanXRQChr10g0431401 | NA |
| HanXRQChr11g0473781 | NA |
| HanXRQChr11g0508551 | NA |
| HanXRQChr13g0575531 | NA |
| HanXRQChr13g0609611 | NA |
| HanXRQChr13g0610801 | NA |
| HanXRQChr13g0617091 | NA |
| HanXRQChr13g0617651 | NA |
| HanXRQChr13g0617751 | NA |
| HanXRQChr16g0755821 | NA |
| HanXRQChr17g0806371 | NA |
| HanXRQChr17g0812381 | NA |

1. drought_noPSC8_on_XRQ

| **gene ID** | ***Arabidopsis thaliana* gene ID** |
| --- | --- |
| HanXRQChr16g0749691 | AT1G01820.1 |
| HanXRQChr01g0002131 | AT1G27530.1 |
| HanXRQChr01g0014651 | NA |
| HanXRQChr04g0179591 | NA |
| HanXRQChr08g0327641 | NA |
| HanXRQChr04g0158091 | NA |
| HanXRQChr11g0473781 | NA |
| HanXRQChr11g0483961 | NA |
| HanXRQChr01g0014641 | NA |
| HanXRQChr04g0158081 | NA |
| HanXRQChr07g0317001 | NA |
| HanXRQChr17g0806371 | NA |

1. control_noXRQ_on_PSC8

| **gene ID** | ***Arabidopsis thaliana* gene ID** |
| --- | --- |
| HanPSC8Chr11g0486671 | AT1G14320.2 |
| HanPSC8Chr14g0614841 | AT1G16130.1 |
| HanPSC8Chr09g0396441 | AT1G23800.1 |
| HanPSC8Chr12g0509901 | AT3G04120.1 |
| HanPSC8Chr01g0023731 | AT3G05000.1 |
| HanPSC8Chr08g0323961 | AT4G15020.1 |
| HanPSC8Chr15g0658571 | AT4G23160.1 |
| HanPSC8Chr04g0176991 | AT5G19350.2 |
| HanPSC8Chr11g0455141 | AT5G62140.1 |
| HanPSC8Chr01g0003721 | NA |
| HanPSC8Chr01g0037761 | NA |
| HanPSC8Chr02g0070261 | NA |
| HanPSC8Chr02g0079911 | NA |
| HanPSC8Chr02g0079921 | NA |
| HanPSC8Chr04g0176981 | NA |
| HanPSC8Chr05g0195611 | NA |
| HanPSC8Chr05g0199131 | NA |
| HanPSC8Chr05g0226071 | NA |
| HanPSC8Chr06g0231491 | NA |
| HanPSC8Chr08g0309931 | NA |
| HanPSC8Chr08g0333631 | NA |
| HanPSC8Chr08g0337111 | NA |
| HanPSC8Chr08g0337121 | NA |
| HanPSC8Chr09g0367021 | NA |
| HanPSC8Chr10g0422541 | NA |
| HanPSC8Chr10g0422551 | NA |
| HanPSC8Chr10g0441851 | NA |
| HanPSC8Chr11g0486661 | NA |
| HanPSC8Chr12g0538071 | NA |
| HanPSC8Chr13g0586921 | NA |
| HanPSC8Chr13g0591581 | NA |
| HanPSC8Chr14g0595091 | NA |
| HanPSC8Chr14g0595821 | NA |
| HanPSC8Chr14g0600911 | NA |
| HanPSC8Chr15g0645241 | NA |
| HanPSC8Chr15g0657011 | NA |
| HanPSC8Chr15g0660271 | NA |
| HanPSC8Chr16g0705961 | NA |
| HanPSC8Chr17g0757391 | NA |
| HanPSC8Chr17g0773091 | NA |
| HanPSC8Chr17g0773121 | NA |
| HanPSC8Chr17g0774531 | NA |
| HanPSC8Chr17g0776851 | NA |

1. drought_noXRQ_on_PSC8

| **gene ID** | ***Arabidopsis thaliana* gene ID** |
| --- | --- |
| HanPSC8Chr11g0486671 | AT1G14320.2 |
| HanPSC8Chr10g0409081 | AT2G24220.1 |
| HanPSC8Chr12g0509901 | AT3G04120.1 |
| HanPSC8Chr01g0023731 | AT3G05000.1 |
| HanPSC8Chr08g0323961 | AT4G15020.1 |
| HanPSC8Chr15g0658571 | AT4G23160.1 |
| HanPSC8Chr04g0174781 | AT5G03040.1 |
| HanPSC8Chr04g0176991 | AT5G19350.2 |
| HanPSC8Chr00c059g0803901 | NA |
| HanPSC8Chr01g0003461 | NA |
| HanPSC8Chr01g0037761 | NA |
| HanPSC8Chr01g0042211 | NA |
| HanPSC8Chr02g0070261 | NA |
| HanPSC8Chr02g0079911 | NA |
| HanPSC8Chr02g0079921 | NA |
| HanPSC8Chr03g0095341 | NA |
| HanPSC8Chr03g0098291 | NA |
| HanPSC8Chr03g0098481 | NA |
| HanPSC8Chr04g0135861 | NA |
| HanPSC8Chr05g0187531 | NA |
| HanPSC8Chr05g0195611 | NA |
| HanPSC8Chr05g0199131 | NA |
| HanPSC8Chr05g0226071 | NA |
| HanPSC8Chr06g0231491 | NA |
| HanPSC8Chr06g0234651 | NA |
| HanPSC8Chr06g0244411 | NA |
| HanPSC8Chr08g0333631 | NA |
| HanPSC8Chr08g0337111 | NA |
| HanPSC8Chr08g0337121 | NA |
| HanPSC8Chr09g0367021 | NA |
| HanPSC8Chr09g0371501 | NA |
| HanPSC8Chr10g0422551 | NA |
| HanPSC8Chr10g0435881 | NA |
| HanPSC8Chr10g0440551 | NA |
| HanPSC8Chr10g0441851 | NA |
| HanPSC8Chr11g0486661 | NA |
| HanPSC8Chr12g0539851 | NA |
| HanPSC8Chr13g0586921 | NA |
| HanPSC8Chr13g0591581 | NA |
| HanPSC8Chr14g0595091 | NA |
| HanPSC8Chr14g0595821 | NA |
| HanPSC8Chr14g0600911 | NA |
| HanPSC8Chr14g0629001 | NA |
| HanPSC8Chr15g0645241 | NA |
| HanPSC8Chr15g0648571 | NA |
| HanPSC8Chr15g0663941 | NA |
| HanPSC8Chr16g0695381 | NA |
| HanPSC8Chr16g0697551 | NA |
| HanPSC8Chr16g0717641 | NA |
| HanPSC8Chr17g0754271 | NA |
| HanPSC8Chr17g0757391 | NA |
| HanPSC8Chr17g0772181 | NA |
| HanPSC8Chr17g0773091 | NA |
| HanPSC8Chr17g0773121 | NA |
| HanPSC8Chr17g0776851 | NA |
| HanPSC8Chr17g0779131 | NA |

Supplementary Table S2. Enrichment of Gene Ontology (GO) terms and Kyoto Encyclopedia of Genes and Genomes (KEGG) pathways.

1. DEG_up_drought

| **Category** | **Enrichment FDR** | **Genes in list** | **Total genes** | **Functional Category** |
| --- | --- | --- | --- | --- |
| Biological process | 4.31E-21 | 335 | 3194 | Response to chemical |
| Biological process | 2.28E-20 | 217 | 1779 | Response to oxygen-containing compound |
| Biological process | 1.63E-18 | 359 | 3648 | Response to stress |
| Biological process | 3.29E-17 | 169 | 1334 | Response to acid chemical |
| Biological process | 3.73E-16 | 234 | 2150 | Response to abiotic stimulus |
| Biological process | 7.05E-15 | 233 | 2194 | Response to organic substance |
| Biological process | 7.05E-15 | 132 | 995 | Response to inorganic substance |
| Biological process | 6.74E-14 | 96 | 640 | Response to abscisic acid |
| Biological process | 6.74E-14 | 117 | 861 | Response to lipid |
| Biological process | 9.09E-14 | 96 | 644 | Response to alcohol |
| Cellular component | 1.06E-14 | 140 | 1087 | Nuclear lumen |
| Cellular component | 2.09E-14 | 173 | 1488 | Nuclear part |
| Cellular component | 6.53E-14 | 157 | 1330 | Membrane-enclosed lumen |
| Cellular component | 6.53E-14 | 157 | 1330 | Organelle lumen |
| Cellular component | 6.53E-14 | 157 | 1330 | Intracellular organelle lumen |
| Cellular component | 4.47E-13 | 236 | 2350 | Cytosol |
| Cellular component | 1.25E-12 | 78 | 501 | Nucleoplasm |
| Cellular component | 1.61E-12 | 66 | 388 | Nucleoplasm part |
| Cellular component | 1.53E-05 | 60 | 497 | Nucleolus |
| Cellular component | 1.53E-05 | 24 | 124 | Nuclear body |
| Molecular function | 9.70E-09 | 319 | 3679 | Cation binding |
| Molecular function | 9.70E-09 | 317 | 3664 | Metal ion binding |
| Molecular function | 1.44E-07 | 343 | 4138 | Nucleic acid binding |
| Molecular function | 1.60E-05 | 222 | 2592 | Drug binding |
| Molecular function | 1.60E-05 | 277 | 3379 | Small molecule binding |
| Molecular function | 3.23E-05 | 274 | 3370 | Anion binding |
| Molecular function | 3.69E-05 | 133 | 1413 | RNA binding |
| Molecular function | 4.77E-05 | 25 | 135 | Unfolded protein binding |
| Molecular function | 0.000111344 | 255 | 3169 | Nucleotide binding |
| Molecular function | 0.000111344 | 204 | 2433 | ATP binding |
| KEGG | 0.000309696 | 183 | 2185 | Metabolic pathways |
| KEGG | 0.000309696 | 31 | 209 | Protein processing in endoplasmic reticulum |
| KEGG | 0.000357523 | 28 | 188 | Spliceosome |
| KEGG | 0.000849332 | 12 | 50 | Valine, leucine and isoleucine degradation |
| KEGG | 0.002428118 | 96 | 1076 | Biosynthesis of secondary metabolites |
| KEGG | 0.002847988 | 10 | 43 | Beta-Alanine metabolism |
| KEGG | 0.002847988 | 16 | 95 | Glycerophospholipid metabolism |
| KEGG | 0.007123497 | 31 | 271 | Plant hormone signal transduction |
| KEGG | 0.009619678 | 11 | 60 | Glycerolipid metabolism |
| KEGG | 0.012850585 | 10 | 54 | Arginine and proline metabolism |

1. DEG_down_drought

| **Category** | **Enrichment FDR** | **Genes in list** | **Total genes** | **Functional Category** |
| --- | --- | --- | --- | --- |
| Biological process | 1.03E-24 | 83 | 275 | Photosynthesis |
| Biological process | 7.60E-21 | 278 | 1963 | Small molecule metabolic process |
| Biological process | 6.04E-17 | 322 | 2538 | Phosphorus metabolic process |
| Biological process | 1.48E-16 | 316 | 2496 | Phosphate-containing compound metabolic process |
| Biological process | 3.28E-16 | 140 | 825 | Small molecule biosynthetic process |
| Biological process | 2.66E-15 | 138 | 829 | Drug metabolic process |
| Biological process | 4.10E-15 | 178 | 1195 | Carbohydrate metabolic process |
| Biological process | 4.61E-15 | 46 | 142 | Photosynthesis, light reaction |
| Biological process | 4.79E-14 | 127 | 765 | Cell wall organization or biogenesis |
| Biological process | 6.22E-14 | 370 | 3194 | Response to chemical |
| Cellular component | 2.44E-82 | 489 | 2582 | Chloroplast |
| Cellular component | 1.51E-79 | 345 | 1487 | Chloroplast part |
| Cellular component | 4.75E-79 | 490 | 2653 | Plastid |
| Cellular component | 7.55E-79 | 345 | 1499 | Plastid part |
| Cellular component | 1.30E-59 | 212 | 784 | Chloroplast stroma |
| Cellular component | 5.78E-59 | 212 | 791 | Plastid stroma |
| Cellular component | 4.42E-49 | 547 | 3832 | Cell periphery |
| Cellular component | 5.87E-42 | 176 | 728 | Plastid envelope |
| Cellular component | 1.13E-41 | 173 | 711 | Chloroplast envelope |
| Cellular component | 4.10E-39 | 450 | 3155 | Plasma membrane |
| Molecular function | 1.01E-14 | 475 | 4302 | Transferase activity |
| Molecular function | 1.01E-14 | 392 | 3379 | Small molecule binding |
| Molecular function | 1.01E-14 | 390 | 3370 | Anion binding |
| Molecular function | 1.00E-13 | 366 | 3169 | Nucleotide binding |
| Molecular function | 1.00E-13 | 366 | 3169 | Nucleoside phosphate binding |
| Molecular function | 3.52E-11 | 311 | 2704 | Purine ribonucleoside triphosphate binding |
| Molecular function | 1.77E-10 | 164 | 1216 | Transmembrane transporter activity |
| Molecular function | 2.30E-10 | 296 | 2592 | Drug binding |
| Molecular function | 2.30E-10 | 320 | 2852 | Ribonucleotide binding |
| Molecular function | 2.54E-10 | 108 | 698 | Inorganic molecular entity transmembrane transporter |
| KEGG | 1.64E-37 | 345 | 2185 | Metabolic pathways |
| KEGG | 1.00E-30 | 201 | 1076 | Biosynthesis of secondary metabolites |
| KEGG | 1.01E-07 | 30 | 116 | Glycolysis / Gluconeogenesis |
| KEGG | 2.42E-07 | 49 | 264 | Carbon metabolism |
| KEGG | 2.57E-06 | 44 | 244 | Biosynthesis of amino acids |
| KEGG | 2.80E-06 | 20 | 69 | Carbon fixation in photosynthetic organisms |
| KEGG | 2.92E-06 | 21 | 76 | Photosynthesis |
| KEGG | 4.64E-05 | 18 | 69 | Glycine, serine and threonine metabolism |
| KEGG | 4.92E-05 | 16 | 57 | Pyrimidine metabolism |
| KEGG | 4.92E-05 | 19 | 77 | Glyoxylate and dicarboxylate metabolism |

1. DAS_up_drought

| **Category** | **Enrichment FDR** | **Genes in list** | **Total genes** | **Functional Category** |
| --- | --- | --- | --- | --- |
| Biological process | 7.96E-42 | 91 | 275 | Photosynthesis |
| Biological process | 2.94E-29 | 243 | 1963 | Small molecule metabolic process |
| Biological process | 1.44E-28 | 106 | 519 | Generation of precursor metabolites and energy |
| Biological process | 6.73E-26 | 226 | 1865 | Organonitrogen compound biosynthetic process |
| Biological process | 2.61E-21 | 145 | 1046 | Cellular amide metabolic process |
| Biological process | 1.90E-20 | 46 | 142 | Photosynthesis, light reaction |
| Biological process | 2.80E-20 | 233 | 2150 | Response to abiotic stimulus |
| Biological process | 7.57E-20 | 207 | 1835 | Oxidation-reduction process |
| Biological process | 3.49E-18 | 118 | 829 | Drug metabolic process |
| Biological process | 1.18E-17 | 123 | 898 | Peptide metabolic process |
| Cellular component | 2.47E-167 | 527 | 2582 | Chloroplast |
| Cellular component | 1.40E-166 | 533 | 2653 | Plastid |
| Cellular component | 4.92E-160 | 392 | 1487 | Chloroplast part |
| Cellular component | 1.09E-159 | 393 | 1499 | Plastid part |
| Cellular component | 1.21E-104 | 207 | 603 | Thylakoid |
| Cellular component | 3.41E-96 | 226 | 791 | Plastid stroma |
| Cellular component | 3.41E-96 | 225 | 784 | Chloroplast stroma |
| Cellular component | 2.40E-91 | 180 | 520 | Chloroplast thylakoid |
| Cellular component | 3.10E-91 | 180 | 521 | Plastid thylakoid |
| Cellular component | 1.11E-83 | 267 | 1243 | Organelle envelope |
| Molecular function | 5.23E-16 | 32 | 84 | Protein domain specific binding |
| Molecular function | 5.63E-13 | 95 | 698 | Inorganic molecular entity transmembrane transporter activity |
| Molecular function | 6.66E-13 | 69 | 427 | Structural constituent of ribosome |
| Molecular function | 4.48E-12 | 312 | 3679 | Cation binding |
| Molecular function | 4.48E-12 | 129 | 1137 | Cofactor binding |
| Molecular function | 1.19E-11 | 168 | 1664 | Oxidoreductase activity |
| Molecular function | 1.52E-11 | 308 | 3664 | Metal ion binding |
| Molecular function | 2.58E-11 | 79 | 577 | Structural molecule activity |
| Molecular function | 8.58E-11 | 90 | 718 | Ion transmembrane transporter activity |
| Molecular function | 9.41E-11 | 20 | 51 | ATPase activity, coupled to transmembrane movement of ions |
| KEGG | 4.76E-47 | 299 | 2185 | Metabolic pathways |
| KEGG | 2.40E-22 | 46 | 135 | Oxidative phosphorylation |
| KEGG | 2.53E-20 | 143 | 1076 | Biosynthesis of secondary metabolites |
| KEGG | 3.09E-15 | 54 | 264 | Carbon metabolism |
| KEGG | 1.08E-13 | 57 | 315 | Ribosome |
| KEGG | 3.65E-10 | 23 | 77 | Glyoxylate and dicarboxylate metabolism |
| KEGG | 1.56E-09 | 21 | 69 | Carbon fixation in photosynthetic organisms |
| KEGG | 6.40E-08 | 20 | 76 | Photosynthesis |
| KEGG | 1.62E-06 | 15 | 53 | Porphyrin and chlorophyll metabolism |
| KEGG | 1.81E-06 | 16 | 61 | Citrate cycle (TCA cycle) |

1. DAS_down_drought

| **Category** | **Enrichment FDR** | **Genes in list** | **Total genes** | **Functional Category** |
| --- | --- | --- | --- | --- |
| Biological process | 3.24E-07 | 54 | 1963 | Small molecule metabolic process |
| Biological process | 4.68E-06 | 70 | 3194 | Response to chemical |
| Biological process | 1.99E-05 | 74 | 3648 | Response to stress |
| Biological process | 1.99E-05 | 16 | 275 | Photosynthesis |
| Biological process | 2.88E-05 | 28 | 825 | Small molecule biosynthetic process |
| Biological process | 4.22E-05 | 51 | 2194 | Response to organic substance |
| Biological process | 4.77E-05 | 50 | 2150 | Response to abiotic stimulus |
| Biological process | 0.000114891 | 43 | 1779 | Response to oxygen-containing compound |
| Biological process | 0.00021153 | 29 | 995 | Response to inorganic substance |
| Biological process | 0.000230189 | 22 | 638 | Organic acid biosynthetic process |
| Cellular component | 1.07E-14 | 78 | 2582 | Chloroplast |
| Cellular component | 2.44E-14 | 78 | 2653 | Plastid |
| Cellular component | 9.65E-14 | 55 | 1487 | Chloroplast part |
| Cellular component | 1.02E-13 | 55 | 1499 | Plastid part |
| Cellular component | 2.37E-08 | 25 | 520 | Chloroplast thylakoid |
| Cellular component | 2.37E-08 | 25 | 521 | Plastid thylakoid |
| Cellular component | 8.35E-08 | 30 | 784 | Chloroplast stroma |
| Cellular component | 8.95E-08 | 30 | 791 | Plastid stroma |
| Cellular component | 2.22E-07 | 21 | 429 | Chloroplast thylakoid membrane |
| Cellular component | 2.22E-07 | 25 | 603 | Thylakoid |
| Molecular function | 0.00024989 | 62 | 3169 | Nucleotide binding |
| Molecular function | 0.00024989 | 66 | 3379 | Small molecule binding |
| Molecular function | 0.00024989 | 66 | 3370 | Anion binding |
| Molecular function | 0.00024989 | 69 | 3679 | Cation binding |
| Molecular function | 0.00024989 | 62 | 3169 | Nucleoside phosphate binding |
| Molecular function | 0.000273112 | 57 | 2852 | Ribonucleotide binding |
| Molecular function | 0.000362331 | 57 | 2894 | Carbohydrate derivative binding |
| Molecular function | 0.000518184 | 67 | 3664 | Metal ion binding |
| Molecular function | 0.000546868 | 17 | 465 | Lyase activity |
| Molecular function | 0.000550136 | 55 | 2830 | Purine nucleotide binding |
| KEGG | 2.94E-08 | 57 | 2185 | Metabolic pathways |
| KEGG | 8.82E-07 | 16 | 264 | Carbon metabolism |
| KEGG | 1.48E-05 | 31 | 1076 | Biosynthesis of secondary metabolites |
| KEGG | 0.001675376 | 6 | 76 | Photosynthesis |
| KEGG | 0.001675376 | 6 | 77 | Glyoxylate and dicarboxylate metabolism |
| KEGG | 0.0026664 | 10 | 244 | Biosynthesis of amino acids |
| KEGG | 0.005695124 | 5 | 69 | Carbon fixation in photosynthetic organisms |
| KEGG | 0.017405165 | 4 | 58 | Pentose phosphate pathway |
| KEGG | 0.017405165 | 6 | 135 | Oxidative phosphorylation |
| KEGG | 0.037565541 | 5 | 116 | Glycolysis / Gluconeogenesis |

1. DEG_OD_control

| **Category** | **Enrichment FDR** | **Genes in list** | **Total genes** | **Functional Category** |
| --- | --- | --- | --- | --- |
| Biological process | 1.03E-15 | 96 | 3194 | Response to chemical |
| Biological process | 1.03E-15 | 69 | 1779 | Response to oxygen-containing compound |
| Biological process | 2.97E-12 | 53 | 1334 | Response to acid chemical |
| Biological process | 8.14E-12 | 95 | 3648 | Response to stress |
| Biological process | 1.41E-11 | 22 | 232 | Response to heat |
| Biological process | 8.46E-11 | 67 | 2194 | Response to organic substance |
| Biological process | 7.95E-10 | 64 | 2150 | Response to abiotic stimulus |
| Biological process | 7.95E-10 | 58 | 1835 | Oxidation-reduction process |
| Biological process | 7.04E-09 | 39 | 995 | Response to inorganic substance |
| Biological process | 5.05E-08 | 55 | 1888 | Response to endogenous stimulus |
| Cellular component | 0.00558592 | 72 | 3832 | Cell periphery |
| Cellular component | 0.01816774 | 59 | 3155 | Plasma membrane |
| Cellular component | 0.018202856 | 8 | 178 | Plant-type vacuole |
| Cellular component | 0.018202856 | 25 | 1090 | Endoplasmic reticulum |
| Cellular component | 0.018202856 | 45 | 2350 | Cytosol |
| Cellular component | 0.018202856 | 24 | 1036 | Cell-cell junction |
| Cellular component | 0.018202856 | 24 | 1036 | Plasmodesma |
| Cellular component | 0.018202856 | 19 | 728 | Plastid envelope |
| Cellular component | 0.018202856 | 19 | 711 | Chloroplast envelope |
| Cellular component | 0.018202856 | 24 | 1036 | Cell junction |
| Molecular function | 1.21E-09 | 55 | 1664 | Oxidoreductase activity |
| Molecular function | 1.07E-06 | 13 | 135 | Unfolded protein binding |
| Molecular function | 7.06E-06 | 14 | 201 | Oxidoreductase activity |
| Molecular function | 7.06E-06 | 78 | 3679 | Cation binding |
| Molecular function | 7.06E-06 | 78 | 3664 | Metal ion binding |
| Molecular function | 7.06E-06 | 13 | 169 | Dioxygenase activity |
| Molecular function | 8.74E-06 | 19 | 389 | Oxidoreductase activity |
| Molecular function | 9.01E-06 | 17 | 317 | Monooxygenase activity |
| Molecular function | 3.07E-05 | 19 | 430 | Tetrapyrrole binding |
| Molecular function | 3.45E-05 | 17 | 355 | Iron ion binding |
| KEGG | 1.74E-09 | 41 | 1076 | Biosynthesis of secondary metabolites |
| KEGG | 9.55E-05 | 50 | 2185 | Metabolic pathways |
| KEGG | 0.001759857 | 5 | 44 | Alpha-Linolenic acid metabolism |
| KEGG | 0.001759857 | 10 | 209 | Protein processing in endoplasmic reticulum |
| KEGG | 0.002418499 | 4 | 29 | Carotenoid biosynthesis |
| KEGG | 0.010349228 | 3 | 21 | Flavonoid biosynthesis |
| KEGG | 0.011606797 | 3 | 23 | Glucosinolate biosynthesis |
| KEGG | 0.011702117 | 2 | 7 | Monoterpenoid biosynthesis |
| KEGG | 0.032096607 | 4 | 68 | Cyanoamino acid metabolism |
| KEGG | 0.032791374 | 4 | 71 | 2-Oxocarboxylic acid metabolism |

1. DEG_UD_control

| **Category** | **Enrichment FDR** | **Genes in list** | **Total genes** | **Functional Category** |
| --- | --- | --- | --- | --- |
| Biological process | 0.001913585 | 14 | 1334 | Response to acid chemical |
| Biological process | 0.001913585 | 19 | 2538 | Phosphorus metabolic process |
| Biological process | 0.001913585 | 19 | 2496 | Phosphate-containing compound metabolic process |
| Biological process | 0.001913585 | 18 | 2150 | Response to abiotic stimulus |
| Biological process | 0.001913585 | 16 | 1888 | Response to endogenous stimulus |
| Biological process | 0.001913585 | 16 | 1853 | Response to hormone |
| Biological process | 0.001913585 | 5 | 142 | Photosynthesis, light reaction |
| Biological process | 0.001913585 | 11 | 861 | Response to lipid |
| Biological process | 0.001913585 | 17 | 1779 | Response to oxygen-containing compound |
| Biological process | 0.002400413 | 8 | 494 | Inorganic ion transmembrane transport |
| Cellular component | 5.92E-08 | 12 | 429 | Chloroplast thylakoid membrane |
| Cellular component | 5.92E-08 | 12 | 450 | Photosynthetic membrane |
| Cellular component | 5.92E-08 | 12 | 449 | Thylakoid membrane |
| Cellular component | 5.92E-08 | 12 | 430 | Plastid thylakoid membrane |
| Cellular component | 8.26E-08 | 12 | 473 | Thylakoid part |
| Cellular component | 1.05E-07 | 13 | 603 | Thylakoid |
| Cellular component | 1.50E-07 | 12 | 520 | Chloroplast thylakoid |
| Cellular component | 1.50E-07 | 12 | 521 | Plastid thylakoid |
| Cellular component | 3.26E-06 | 17 | 1487 | Chloroplast part |
| Cellular component | 3.28E-06 | 17 | 1499 | Plastid part |
| Molecular function | 0.000263779 | 11 | 718 | Ion transmembrane transporter activity |
| Molecular function | 0.000263779 | 7 | 252 | Proton transmembrane transporter activity |
| Molecular function | 0.000263779 | 11 | 698 | Inorganic molecular entity transmembrane transporter |
| Molecular function | 0.00058618 | 7 | 298 | Monovalent inorganic cation transmembrane transporter |
| Molecular function | 0.000866623 | 8 | 451 | Inorganic cation transmembrane transporter activity |
| Molecular function | 0.000946047 | 9 | 609 | Active transmembrane transporter activity |
| Molecular function | 0.001173617 | 8 | 494 | Cation transmembrane transporter activity |
| Molecular function | 0.001256124 | 7 | 375 | Secondary active transmembrane transporter activity |
| Molecular function | 0.001411986 | 5 | 167 | Symporter activity |
| Molecular function | 0.001808625 | 2 | 7 | Calcium:cation antiporter activity |
| KEGG | 0.00426061 | 3 | 76 | Photosynthesis |
| KEGG | 0.00426061 | 15 | 2185 | Metabolic pathways |
| KEGG | 0.01169813 | 3 | 131 | MAPK signaling pathway |
| KEGG | 0.01169813 | 4 | 271 | Plant hormone signal transduction |
| KEGG | 0.039592678 | 2 | 95 | Pentose and glucuronate interconversions |

1. DEG_OD_drought

| **Category** | **Enrichment FDR** | **Genes in list** | **Total genes** | **Functional Category** |
| --- | --- | --- | --- | --- |
| Biological process | 0.039034078 | 3 | 381 | Response to water deprivation |
| Biological process | 0.039034078 | 3 | 389 | Response to water |
| Biological process | 0.039034078 | 2 | 81 | Response to high light intensity |
| Biological process | 0.053267527 | 7 | 3648 | Response to stress |
| Biological process | 0.053267527 | 2 | 163 | Response to light intensity |
| Biological process | 0.053267527 | 2 | 137 | Response to chitin |
| Biological process | 0.058392179 | 2 | 185 | Protein folding |
| Biological process | 0.061150583 | 2 | 203 | Response to wounding |
| Biological process | 0.08744812 | 2 | 260 | Response to organonitrogen compound |
| Cellular component | 0.012270827 | 6 | 2350 | Cytosol |
| Molecular function | 0.005443451 | 2 | 42 | Chaperone binding |
| Molecular function | 0.045544374 | 2 | 215 | Ubiquitin protein ligase activity |
| Molecular function | 0.045544374 | 2 | 215 | Ubiquitin-like protein ligase activity |

1. DEG_UD_drought

| **Category** | **Enrichment FDR** | **Genes in list** | **Total genes** | **Functional Category** |
| --- | --- | --- | --- | --- |
| Biological process | 0.019131685 | 2 | 8 | thiamine biosynthetic process |
| Biological process | 0.019131685 | 2 | 8 | thiamine-containing compound biosynthetic process |
| Biological process | 0.029346301 | 2 | 18 | glycogen metabolic process |
| Biological process | 0.029346301 | 2 | 18 | energy reserve metabolic process |
| Biological process | 0.029346301 | 2 | 14 | thiamine metabolic process |
| Biological process | 0.029346301 | 11 | 1344 | response to abiotic stimulus |
| Biological process | 0.029346301 | 2 | 14 | thiamine-containing compound metabolic process |
| Biological process | 0.035058433 | 2 | 21 | sesquiterpenoid biosynthetic process |
| Biological process | 0.059405717 | 2 | 29 | sesquiterpenoid metabolic process |
| Biological process | 0.062098382 | 4 | 230 | response to water deprivation |
| KEGG | 0.002635606 | 2 | 10 | Thiamine metabolism |
| KEGG | 0.009095051 | 3 | 110 | Phenylalanine metabolism |
| KEGG | 0.009095051 | 2 | 41 | beta-Alanine metabolism |
| KEGG | 0.009095051 | 2 | 40 | Nitrogen metabolism |
| KEGG | 0.009095051 | 8 | 962 | Biosynthesis of secondary metabolites |
| KEGG | 0.011615957 | 11 | 1793 | Metabolic pathways |
| KEGG | 0.071559878 | 2 | 148 | Phenylpropanoid biosynthesis |
| KEGG | 0.091954328 | 2 | 185 | Starch and sucrose metabolism |

1. DASU_control

| **Category** | **Enrichment FDR** | **Genes in list** | **Total genes** | **Functional Category** |
| --- | --- | --- | --- | --- |
| Biological process | 4.05E-16 | 82 | 2150 | Response to abiotic stimulus |
| Biological process | 2.34E-15 | 29 | 275 | Photosynthesis |
| Biological process | 8.26E-13 | 71 | 1963 | Small molecule metabolic process |
| Biological process | 8.37E-13 | 68 | 1835 | Oxidation-reduction process |
| Biological process | 2.09E-12 | 41 | 752 | Response to light stimulus |
| Biological process | 4.36E-12 | 41 | 773 | Response to radiation |
| Biological process | 4.31E-08 | 36 | 829 | Drug metabolic process |
| Biological process | 1.11E-07 | 39 | 995 | Response to inorganic substance |
| Biological process | 1.11E-07 | 32 | 702 | Organophosphate metabolic process |
| Biological process | 2.16E-07 | 25 | 460 | Organophosphate biosynthetic process |
| Cellular component | 1.96E-58 | 150 | 2582 | Chloroplast |
| Cellular component | 5.01E-58 | 151 | 2653 | Plastid |
| Cellular component | 3.94E-50 | 109 | 1487 | Chloroplast part |
| Cellular component | 6.54E-50 | 109 | 1499 | Plastid part |
| Cellular component | 9.49E-32 | 65 | 784 | Chloroplast stroma |
| Cellular component | 1.12E-31 | 58 | 603 | Thylakoid |
| Cellular component | 1.15E-31 | 65 | 791 | Plastid stroma |
| Cellular component | 3.10E-28 | 51 | 520 | Chloroplast thylakoid |
| Cellular component | 3.10E-28 | 51 | 521 | Plastid thylakoid |
| Cellular component | 2.61E-25 | 46 | 473 | Thylakoid part |
| Molecular function | 5.26E-08 | 55 | 1664 | Oxidoreductase activity |
| Molecular function | 0.00137885 | 8 | 84 | Protein domain specific binding |
| Molecular function | 0.00137885 | 72 | 3370 | Anion binding |
| Molecular function | 0.002037576 | 71 | 3379 | Small molecule binding |
| Molecular function | 0.002334086 | 36 | 1362 | Transporter activity |
| Molecular function | 0.002334086 | 5 | 30 | Proton-transporting ATP synthase activity, rotational mechanism |
| Molecular function | 0.002502707 | 66 | 3169 | Nucleotide binding |
| Molecular function | 0.002502707 | 11 | 204 | Oxidoreductase activity, acting on CH-OH group of donors |
| Molecular function | 0.002502707 | 10 | 171 | Oxidoreductase activity, acting on the CH-OH group of donors, NAD or NADP as acceptor |
| Molecular function | 0.002502707 | 31 | 1137 | Cofactor binding |
| KEGG | 1.81E-23 | 93 | 2185 | Metabolic pathways |
| KEGG | 8.48E-17 | 55 | 1076 | Biosynthesis of secondary metabolites |
| KEGG | 2.46E-14 | 26 | 264 | Carbon metabolism |
| KEGG | 2.74E-10 | 13 | 77 | Glyoxylate and dicarboxylate metabolism |
| KEGG | 1.48E-08 | 11 | 69 | Carbon fixation in photosynthetic organisms |
| KEGG | 4.68E-07 | 10 | 76 | Photosynthesis |
| KEGG | 2.17E-05 | 10 | 116 | Glycolysis / Gluconeogenesis |
| KEGG | 0.000102083 | 7 | 62 | Fructose and mannose metabolism |
| KEGG | 0.00018417 | 7 | 69 | Glycine, serine and threonine metabolism |
| KEGG | 0.000479779 | 12 | 244 | Biosynthesis of amino acids |

1. DASD_control

| **Category** | **Enrichment FDR** | **Genes in list** | **Total genes** | **Functional Category** |
| --- | --- | --- | --- | --- |
| Biological process | 0.003908516 | 3 | 184 | Purine nucleotide biosynthetic process |
| Biological process | 0.003908516 | 3 | 116 | ATP biosynthetic process |
| Biological process | 0.003908516 | 3 | 169 | Nucleoside monophosphate biosynthetic process |
| Biological process | 0.003908516 | 3 | 202 | Purine nucleoside monophosphate metabolic process |
| Biological process | 0.003908516 | 3 | 144 | Purine nucleoside monophosphate biosynthetic process |
| Biological process | 0.003908516 | 3 | 201 | Nucleoside triphosphate metabolic process |
| Biological process | 0.003908516 | 3 | 138 | Nucleoside triphosphate biosynthetic process |
| Biological process | 0.003908516 | 3 | 184 | Purine nucleoside triphosphate metabolic process |
| Biological process | 0.003908516 | 3 | 124 | Purine nucleoside triphosphate biosynthetic process |
| Biological process | 0.003908516 | 3 | 178 | Purine ribonucleotide biosynthetic process |
| Cellular component | 1.40E-06 | 13 | 2582 | Chloroplast |
| Cellular component | 1.40E-06 | 13 | 2653 | Plastid |
| Cellular component | 2.82E-06 | 10 | 1487 | Chloroplast part |
| Cellular component | 2.82E-06 | 10 | 1499 | Plastid part |
| Cellular component | 1.81E-05 | 7 | 728 | Plastid envelope |
| Cellular component | 1.81E-05 | 7 | 711 | Chloroplast envelope |
| Cellular component | 2.80E-05 | 6 | 520 | Chloroplast thylakoid |
| Cellular component | 2.80E-05 | 6 | 521 | Plastid thylakoid |
| Cellular component | 2.80E-05 | 9 | 1619 | Organelle subcompartment |
| Cellular component | 5.48E-05 | 6 | 603 | Thylakoid |
| Molecular function | 0.016117376 | 2 | 30 | Proton-transporting ATP synthase activity, rotational mechanism |
| Molecular function | 0.023358736 | 2 | 51 | ATPase activity, coupled to transmembrane movement of ions, rotational mechanism |
| Molecular function | 0.029380085 | 2 | 91 | Cation-transporting ATPase activity |
| Molecular function | 0.029380085 | 2 | 91 | Active ion transmembrane transporter activity |
| Molecular function | 0.029380085 | 2 | 91 | ATPase coupled ion transmembrane transporter activity |
| Molecular function | 0.058748241 | 3 | 451 | Inorganic cation transmembrane transporter activity |
| Molecular function | 0.064618555 | 3 | 494 | Cation transmembrane transporter activity |
| Molecular function | 0.074440387 | 2 | 252 | Proton transmembrane transporter activity |
| Molecular function | 0.074440387 | 2 | 237 | Primary active transmembrane transporter activity |
| Molecular function | 0.074440387 | 2 | 236 | P-P-bond-hydrolysis-driven transmembrane transporter activity |

1. DASU_drought

| **Category** | **Enrichment FDR** | **Genes in list** | **Total genes** | **Functional Category** |
| --- | --- | --- | --- | --- |
| Biological process | 6.12E-12 | 33 | 2150 | Response to abiotic stimulus |
| Biological process | 7.11E-07 | 18 | 995 | Response to inorganic substance |
| Biological process | 3.14E-06 | 15 | 752 | Response to light stimulus |
| Biological process | 3.38E-06 | 15 | 773 | Response to radiation |
| Biological process | 1.66E-05 | 29 | 3194 | Response to chemical |
| Biological process | 6.48E-05 | 30 | 3648 | Response to stress |
| Biological process | 6.48E-05 | 12 | 632 | Response to temperature stimulus |
| Biological process | 6.98E-05 | 5 | 58 | de novo protein folding |
| Biological process | 0.000164396 | 3 | 10 | Nonphotochemical quenching |
| Biological process | 0.000164396 | 3 | 10 | Energy quenching |
| Cellular component | 2.64E-08 | 30 | 2582 | Chloroplast |
| Cellular component | 2.64E-08 | 30 | 2653 | Plastid |
| Cellular component | 2.41E-07 | 21 | 1487 | Chloroplast part |
| Cellular component | 2.41E-07 | 21 | 1499 | Plastid part |
| Cellular component | 5.52E-07 | 15 | 791 | Plastid stroma |
| Cellular component | 5.52E-07 | 15 | 784 | Chloroplast stroma |
| Cellular component | 9.45E-07 | 13 | 603 | Thylakoid |
| Cellular component | 1.15E-05 | 23 | 2350 | Cytosol |
| Cellular component | 1.15E-05 | 10 | 429 | Chloroplast thylakoid membrane |
| Cellular component | 1.15E-05 | 10 | 430 | Plastid thylakoid membrane |
| Molecular function | 0.003769393 | 3 | 28 | Water transmembrane transporter activity |
| Molecular function | 0.003769393 | 3 | 28 | Water channel activity |
| Molecular function | 0.003769393 | 5 | 135 | Unfolded protein binding |
| Molecular function | 0.004072491 | 3 | 34 | Chlorophyll binding |
| Molecular function | 0.004072491 | 4 | 84 | Protein domain specific binding |
| Molecular function | 0.005121704 | 3 | 39 | Heat shock protein binding |
| Molecular function | 0.006889286 | 6 | 297 | Isomerase activity |
| Molecular function | 0.014056092 | 14 | 1664 | Oxidoreductase activity |
| Molecular function | 0.018999626 | 3 | 70 | Carboxy-lyase activity |
| Molecular function | 0.046661637 | 6 | 465 | Lyase activity |
| KEGG | 8.44E-08 | 26 | 2185 | Metabolic pathways |
| KEGG | 0.000286659 | 4 | 60 | Glycerolipid metabolism |
| KEGG | 0.000286659 | 13 | 1076 | Biosynthesis of secondary metabolites |
| KEGG | 0.028475376 | 3 | 116 | Glycolysis / Gluconeogenesis |
| KEGG | 0.032357548 | 2 | 57 | Galactose metabolism |
| KEGG | 0.032357548 | 2 | 54 | Arginine and proline metabolism |
| KEGG | 0.032357548 | 4 | 264 | Carbon metabolism |
| KEGG | 0.032357548 | 2 | 53 | Protein export |
| KEGG | 0.037095638 | 2 | 69 | Glycine, serine and threonine metabolism |
| KEGG | 0.037095638 | 2 | 69 | Carbon fixation in photosynthetic organisms |

1. DASD_drought

| **Category** | **Enrichment FDR** | **Genes in list** | **Total genes** | **Functional Category** |
| --- | --- | --- | --- | --- |
| Biological process | 0.022302564 | 2 | 25 | Oxylipin metabolic process |
| Biological process | 0.022302564 | 2 | 24 | Oxylipin biosynthetic process |
| Biological process | 0.022302564 | 9 | 1835 | Oxidation-reduction process |
| Biological process | 0.026557889 | 3 | 142 | Photosynthesis, light reaction |
| Biological process | 0.032699234 | 3 | 184 | Response to reactive oxygen species |
| Biological process | 0.032699234 | 2 | 58 | de novo protein folding |
| Biological process | 0.032699234 | 5 | 773 | Response to radiation |
| Biological process | 0.032699234 | 5 | 752 | Response to light stimulus |
| Biological process | 0.032699234 | 2 | 57 | Photosynthetic electron transport chain |
| Biological process | 0.032699234 | 2 | 48 | de novo posttranslational protein folding |
| Cellular component | 5.80E-05 | 7 | 520 | Chloroplast thylakoid |
| Cellular component | 5.80E-05 | 7 | 521 | Plastid thylakoid |
| Cellular component | 0.000101052 | 7 | 603 | Thylakoid |
| Cellular component | 0.000232714 | 6 | 473 | Thylakoid part |
| Cellular component | 0.000398277 | 9 | 1487 | Chloroplast part |
| Cellular component | 0.000398277 | 9 | 1499 | Plastid part |
| Cellular component | 0.000620424 | 9 | 1619 | Organelle subcompartment |
| Cellular component | 0.000661001 | 9 | 1661 | Mitochondrion |
| Cellular component | 0.000665368 | 11 | 2582 | Chloroplast |
| Cellular component | 0.000665368 | 5 | 429 | Chloroplast thylakoid membrane |
| Molecular function | 0.004625773 | 9 | 1664 | Oxidoreductase activity |
| Molecular function | 0.02543449 | 2 | 42 | Chaperone binding |
| Molecular function | 0.067334828 | 2 | 98 | Carboxylic acid binding |
| Molecular function | 0.067334828 | 2 | 98 | Organic acid binding |
| Molecular function | 0.075346315 | 3 | 354 | Identical protein binding |
| Molecular function | 0.083110684 | 2 | 135 | Unfolded protein binding |
| KEGG | 0.001876849 | 4 | 264 | Carbon metabolism |
| KEGG | 0.002801541 | 9 | 2185 | Metabolic pathways |
| KEGG | 0.005117563 | 2 | 58 | Pentose phosphate pathway |
| KEGG | 0.005358802 | 2 | 76 | Photosynthesis |
| KEGG | 0.005358802 | 2 | 77 | Glyoxylate and dicarboxylate metabolism |
| KEGG | 0.008507145 | 2 | 116 | Glycolysis / Gluconeogenesis |
| KEGG | 0.008507145 | 5 | 1076 | Biosynthesis of secondary metabolites |

Supplementary Table S3. Overview of RNA-Seq data.

| 12S01 (10a= most stressed, 70=least stressed) | | | | |  |
| --- | --- | --- | --- | --- | --- |
| Sample | Raw Reads | Clean Reads | Mapped Reads | Nongenic Reads | GC % |
| INEDI_10a | 25467845 | 21851744 | 17557842 | 4293902 | 47 |
| INEDI_10b | 18745873 | 14237417 | 11626791 | 2611626 | 45 |
| INEDI_25 | 12245712 | 9533515 | 7579965 | 1953550 | 45 |
| INEDI_40 | 24969947 | 21206170 | 16687524 | 4518646 | 46 |
| INEDI_55 | 13417734 | 11531499 | 9180635 | 2350864 | 45 |
| INEDI_70 | 24157929 | 21352401 | 16880707 | 4471694 | 44 |
| PSC8_10a | 21563688 | 19670091 | 15662382 | 4007709 | 46 |
| PSC8_10b | 7452197 | 5958949 | 4795057 | 1163892 | 47 |
| PSC8_25 | 13117623 | 11866851 | 9470043 | 2396808 | 47 |
| PSC8_40 | 19656732 | 17418737 | 13782924 | 3635813 | 46 |
| PSC8_55 | 26345576 | 23793813 | 18800433 | 4993380 | 48 |
| PSC8_70 | 19485524 | 17846300 | 14243565 | 3602735 | 45 |
| XRQ_10a | 17421743 | 14882723 | 12317179 | 2565544 | 46 |
| XRQ_10b | 19798337 | 16981525 | 14350064 | 2631461 | 48 |
| XRQ_25 | 21078054 | 19122979 | 15820250 | 3302729 | 48 |
| XRQ_40 | 20638592 | 18258431 | 14987413 | 3271018 | 47 |
| XRQ_55 | 15748828 | 13295194 | 10838353 | 2456841 | 45 |
| XRQ_70 | 16641025 | 14588361 | 11939560 | 2648801 | 46 |
|  |  |  |  |  |  |
|  |  |  |  |  |  |
|  |  |  |  |  |  |
| 13HP02 (C=control, D=drought) | | | | |  |
| Sample | Raw Reads | Clean Reads | Mapped Reads | Nongenic Reads | GC % |
| INEDIC1 | 35214987 | 32643270 | 4574833 | 28068437 | 43 |
| INEDIC2 | 55246982 | 52107634 | 6972732 | 45134902 | 42 |
| INEDIC3 | 47569854 | 42308797 | 5923981 | 36384816 | 42 |
| INEDID1 | 98265134 | 93623299 | 10075850 | 83547449 | 42 |
| INEDID2 | 165452728 | 149787483 | 12733234 | 137054249 | 42 |
| INEDID3 | 75482195 | 67830933 | 8451795 | 59379138 | 44 |
| PSC8C1 | 114137816 | 106459079 | 13250012 | 93209067 | 42 |
| PSC8C2 | 67127947 | 59695564 | 6321842 | 53373822 | 42 |
| PSC8C3 | 154743845 | 145255701 | 16822845 | 128442856 | 42 |
| PSC8D1 | 97875712 | 92372605 | 11569949 | 80802656 | 43 |
| PSC8D2 | 78245977 | 64790215 | 6852297 | 58937981 | 42 |
| PSC8D3 | 121544261 | 109502562 | 13936449 | 95566113 | 44 |
| XRQC1 | 78471635 | 74372567 | 9265728 | 65106839 | 42 |
| XRQC2 | 69655717 | 63201953 | 10251421 | 52950532 | 42 |
| XRQC3 | 136936682 | 129801274 | 16660961 | 113140313 | 43 |
| XRQD1 | 120002874 | 111630924 | 10992063 | 100638861 | 42 |
| XRQD2 | 49788414 | 45436728 | 5777141 | 39659587 | 41 |
| XRQD3 | 109576887­­­­­­ | 103193952 | 9940778 | 93253174 | 42 |
